# Chiral phosphine-catalyzed tunable cycloaddition reactions of allenoates with benzofuranone-derived olefins for a highly regio-, diastereo- and enantioselective synthesis of spiro-benzofuranones†
†Electronic supplementary information (ESI) available: Experimental procedures, characterization data of new compounds. CCDC 961159, 967550 and 1010550. For ESI and crystallographic data in CIF or other electronic format see DOI: 10.1039/c5sc03135d


**DOI:** 10.1039/c5sc03135d

**Published:** 2015-09-15

**Authors:** De Wang, Guo-Peng Wang, Yao-Liang Sun, Shou-Fei Zhu, Yin Wei, Qi-Lin Zhou, Min Shi

**Affiliations:** a State Key Laboratory of Organometallic Chemistry , Shanghai Institute of Organic Chemistry , Chinese Academy of Sciences , 345 Lingling Road , Shanghai 200032 , P. R. China . Email: mshi@mail.sioc.ac.cn; b State Key Laboratory and Institute of Element-Organic Chemistry , Collaborative Innovation Center of Chemical Science and Engineering (Tianjin) , Nankai University , Tianjin 300071 , China . Email: qlzhou@nankai.edu.cn

## Abstract

The first regioselective catalytic asymmetric [3 + 2] cycloaddition of benzofuranone-derived olefins with allenoates and substituted allenoates has been developed in the presence of (*R*)-SITCP, affording different functionalized 3-spirocyclopentene benzofuran-2-ones in good yields with high enantioselectivities under mild conditions. The substrate scope has also been examined. The regioselective outcomes for this phosphine-catalyzed [3 + 2] cycloaddition reaction can be rationalized using DFT calculations.

## Abstract 2

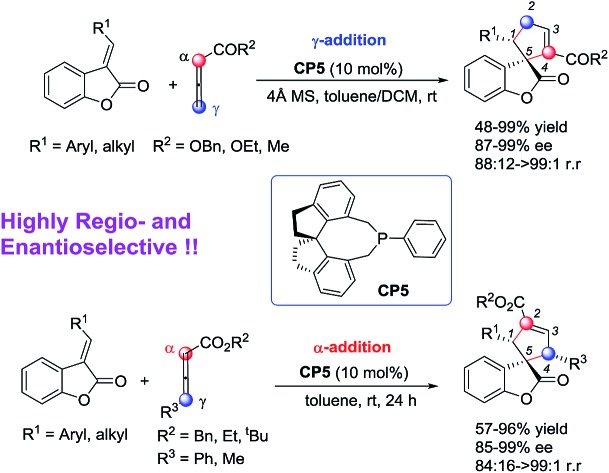
The first regioselective catalytic asymmetric [3 + 2] cycloaddition of benzofuranone-derived olefins with allenoates and substituted allenoates has been developed in the presence of (*R*)-SITCP.

## 

Phosphine-catalyzed [3 + 2] cycloaddition of electron-deficient olefins with allenoates, which provides alternative access to a variety of useful carbocycles, was first reported by Zhang and Lu in 1995.^1^,^2^ Pioneering work on the catalytic asymmetric Lu's [3 + 2] cycloaddition of allenoates with olefins was disclosed by Zhang in 1997.^3^ No further progress was made on the development of this enantioselective [3 + 2] cyclization for about a decade after Zhang's promising results, until Fu and co-workers recently developed a series of axially chiral binaphthyl frameworks containing phosphines that catalyzed the asymmetric cycloaddition of allenoates with electron-deficient olefins, affording the corresponding cycloadducts in good yields with excellent diastereo- and enantioselectivities.^4^ Moreover, Marinetti and co-workers have also discovered that chiral phosphines based on a planar chiral 2-phospha[3]ferrocenophane scaffold were efficient catalysts for this type of asymmetric reaction as well.^5^ A variety of multifunctional chiral phosphines derived from natural amino acids have also emerged as powerful catalysts to promote the [3 + 2] cycloaddition of allenoates with electron-deficient olefins or imines, affording a variety of cyclopentene or pyrrolidine derivatives in good yields with high diastereo- and enantioselectivities under mild conditions.^6^ For example, Miller and co-workers achieved the enantioselective cyclization of allenoates and enones using phosphines containing α-amino acids.6*a* Jacobsen and co-workers utilized phosphine–thiourea catalysts for enantioselective annulations of allenes and imines.6*b* Zhao6*c* and Lu6d–s developed a series of multifunctional phosphine catalysts based on different types of amino acids, and applied these functional phosphine-containing catalysts to different types of cycloadditions. Recently, Kwon's group developed a new class of rigid chiral bicyclic phosphines and applied them to the asymmetric synthesis of multi-substituted pyrrolines.6*t* In addition, some commercially available bidentate chiral phosphine-promoted [3 + 2] cycloadditions have also been reported.^7^

The phosphine-catalyzed [3 + 2] cycloaddition of electron-deficient olefins with allenoates was commonly considered to start from the formation of the corresponding zwitterionic intermediate **I** between PR_3_ and the allenoate. The nature of this zwitterion shown in Scheme 1 may be depicted in two ways, which include anion localization at the α-carbon or γ-carbon, thus two regioisomers derived from the α-addition and γ-addition could be produced at the same time (Scheme 1). Therefore, the selective synthesis of highly regio-, diastereo- and enantioselective products becomes a big challenge. Previous reports mainly focus on how to obtain a single highly regioselective product, however, few people have made efforts to obtain both the α-addition and γ-addition isomers in a controllable way with high regio-, diastereo- and enantioselective values, not to mention the mechanistic study of the regioselectivity.^8^

**Scheme 1:**
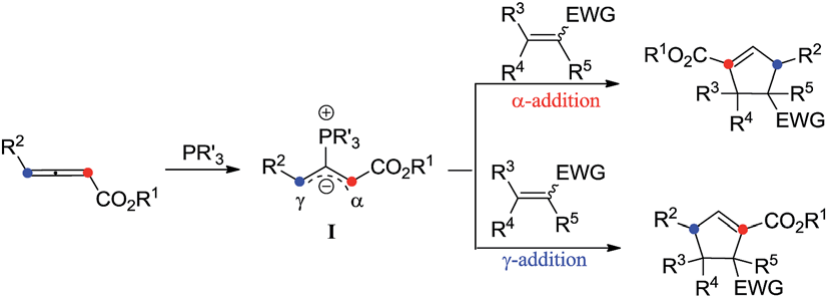
Model of the phosphine catalyzed [3 + 2] cycloaddition.

Benzofuranones as one of the important building blocks exist in a variety of natural products^9^ and potential medicines.^10^ The enantioselective synthesis of chiral benzofuranones remains a considerable challenge,^11^ especially in the field of construction of a chiral spiro-quaternary center at the C3 position of benzofuranones.^12^ As part of our ongoing investigation on phosphine-catalyzed asymmetric cycloaddition,^13^ we wish to report a spiro phosphine (*R*)-SITCP^14^ catalyzed asymmetric [3 + 2] cycloaddition of allenic esters with benzofuranones, furnishing the spiro cycloadducts in good yields with excellent regio-, diastereo- and enantioselectivities, by adjusting the substituents of the allenic esters to obtain both the α-addition and γ-addition products, and using rational DFT calculations to reveal the reason for the regioselectivity. This asymmetric [3 + 2] cycloaddition catalyzed by a chiral phosphine features the simultaneous formation of spiro-quaternary and tertiary stereocenters (two or three chiral centers) in a single step (Scheme 2). In addition, this type of reaction is also suitable for substrates such as arylideneoxindole and alkylidene azlactone, which makes this type of reaction have promising applications.

**Scheme 2:**
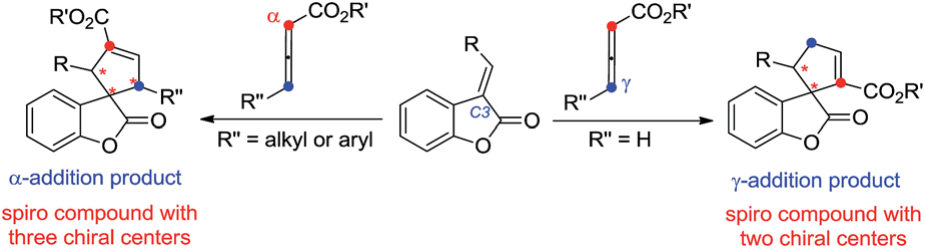
Asymmetric approaches of α- and γ-addition product.

We initially screened a variety of chiral phosphines **CP1–CP8** using (*E*)-3-(2-bromobenzylidene)benzofuran-2(3*H*)-one **1a** and benzyl 2,3-butadienoate **2a** as the model substrates in toluene. The results are summarized in Table 1. We found the γ-addition product **3a** as the main product and the α-addition product **3a′** as the minor product, which were obtained in 26–92% total yields, with the regioselectivity ratios (r.r.) of **3a** : **3a′** from 86 : 14 to 95 : 5, and excellent diastereoselectivities (the minor diastereomer almost could not be detected by ^1^H NMR); the ee value of the main product **3a** is obtained from 8% to 88% (Table 1, entries 1–8). The catalyst **CP5** gave the highest yield, regio- and enantioselectivity compared to other catalysts (Table 1, entry 5). Having identified the best catalyst in this reaction, we next attempted to further optimize the reaction conditions by screening of the solvent and reaction temperature (Table 1, entries 8–14). The reaction outcomes revealed that using 10 mol% of **CP5** as the catalyst and carrying out the reaction in dichloromethane (DCM) and toluene as the mixing solvents (1 : 1) with 4 Å MS (30 mg) as the additive affords **3a** at room temperature for 12 h in 78% yield with >19 : 1 r.r. and 99% ee value, which served as the best reaction conditions for this reaction (Scheme 3, eqn(1)). Using γ-phenyl allenoate **4a** as the Michael acceptor, the reaction proceeded smoothly to give the α-addition product as the major product in 96% yield, with >19 : 1 r.r. and 95% ee value in toluene, however, the reaction proceeded in DCM, diminishing the yield, r.r. and ee value significantly (Scheme 3, eqn (2)).
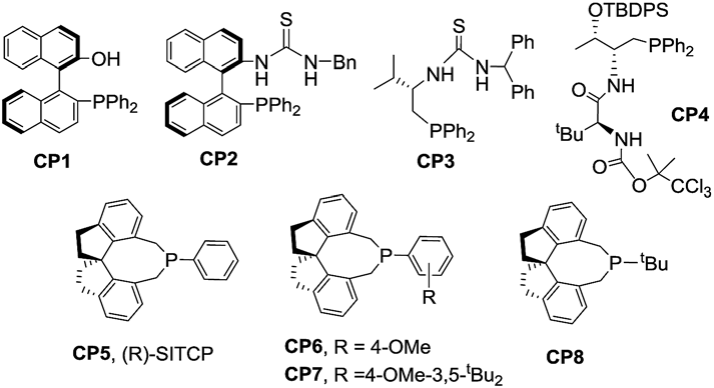


**Table 1:** Optimization of the reaction conditions of α-addition

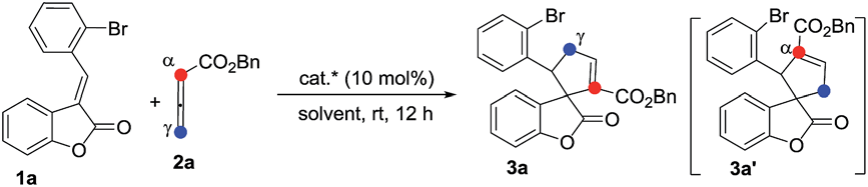						
Entry^*a*^	Cat.*	Solvent	*T* (°C)	Yield^*b*^ (%)	r.r.^*c*^ (**3a** : **3a′**)	ee^*c*^ (%)
1	**CP1**	Toluene	25	37	90 : 10	8
2	**CP2**	Toluene	25	32	88 : 12	20
3	**CP3**	Toluene	25	26	86 : 14	14
4^*d*^	**CP4**	Toluene	25	—	—	—
5	**CP5**	Toluene	25	92	96 : 4	88
6	**CP6**	Toluene	25	72	95 : 5	83
7	**CP7**	Toluene	25	74	94 : 6	88
8	**CP8**	Toluene	25	Trace	92 : 8	13
9	**CP5**	DCM	25	58	>19 : 1	>99
10	**CP5**	THF	25	47	94 : 6	93
11	**CP5**	CH_3_CN	25	22	72 : 28	94
12	**CP5**	Toluene/DCM^*e*^	25	85	>19 : 1	91
13	**CP5**	Toluene/DCM^*f*^	25	64	>19 : 1	98
14	**CP5**	Toluene/DCM^*g*^	25	78	>19 : 1	99
15	**CP5**	Toluene/DCM^*g*^	0	53	>19 : 1	99

^*a*^All reactions were carried out with **1a** (0.1 mmol), **2a** (0.15 mmol), and catalyst (10 mol%) in solvent (1.0 mL).

^*b*^Isolated yield.

^*c*^Determined using ^1^H NMR of the crude product; determined using HPLC.

^*d*^Disordered.

^*e*^Toluene/DCM = 4 : 1.

^*f*^Toluene/DCM = 1 : 1.

^*g*^Toluene/DCM = 1 : 1, 4 Å MS (30 mg) was added as the additive.

**Scheme 3:**
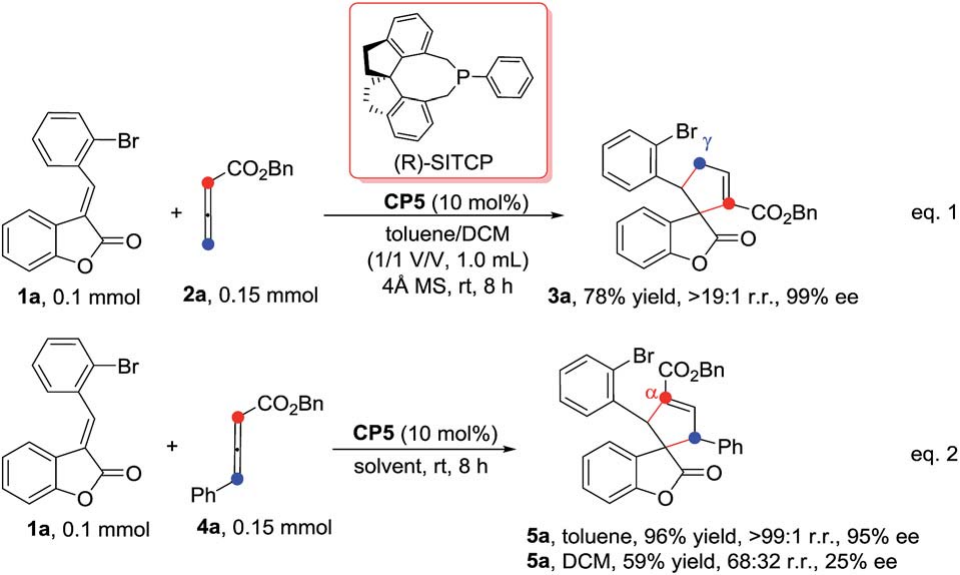
Optimal conditions of α- and γ-addition.

Having identified the optimal reaction conditions, the generality of this (*R*)-SITCP (**CP5**) catalyzed asymmetric γ-addition [3 + 2] cycloaddition was examined using a variety of aryl or alkyl-substituted benzofuranones **1** and allenic esters **2**. The results are summarized in Table 2. Whether R^1^ is an electron-rich or -deficient aromatic ring, the reactions proceeded smoothly to give the corresponding spiro-cycloadducts **3b–3j** in moderate to good yields with 87–96% ee values and 88 : 12 to >99 : 1 r.r. (Table 2, entries 1–9). In the case of 4-CF_3_C_6_H_4_ benzofuranone **1e**, the regioselectivity ratio decreased to 88 : 12 (Table 2, entry 4). Using 4-CNC_6_H_4_ benzofuranone **1g** as the substrate, the corresponding adduct was obtained in 57% yield along with a relatively lower ee value (87% ee) (Table 2, entry 6). When R^1^ is a heteroaromatic group (R^1^ = 2-furyl, 2-thienyl) or a sterically hindered 1-naphthyl moiety, the reactions also proceed efficiently to afford the corresponding products **3k–3m** in 48–99% yields with 93–99% ee values and good regioselectivities (Table 2, entries 10–12). Changing R^1^ from the aromatic group to an aliphatic group provided the corresponding product **3n** in 68% yield with 95% ee and a 98 : 2 regioselectivity ratio (Table 2, entry 13). Other electron deficient allenes such as ethyl-2,3-butadienoate and penta-3,4-dien-2-one are also suitable for this asymmetric [3 + 2] cycloaddition, giving the corresponding products in 94% and 83% yields with 99% and 96% ee values as well as excellent regioselectivities, respectively (Table 2, entries 14 and 15). The absolute configuration of **3m** has been assigned using X-ray diffraction as 1*S*, 5*R*. The ORTEP drawing and the CIF data are summarized in the ESI.† 19

**Table 2:** Scope of the asymmetric [3 + 2] cycloaddition to afford cycloadducts **3b–3q**

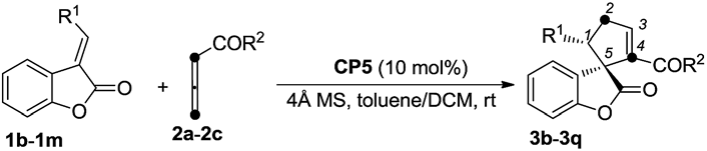					
Entry^*a*^	**1** (R^1^)	**2** (R^2^)	Yield^*b*^ (%)	r.r.^*c*^	ee (%)^*d*^
1	**1b** (4-BrC_6_H_4_)	**2a** (OBn)	**3b**: 92	>99 : 1	95
2	**1c** (4-CH_3_C_6_H_4_)	**2a** (OBn)	**3c**: 76	92 : 8	91
3	**1d** (4-CH_3_OC_6_H_4_)	**2a** (OBn)	**3d**: 72	98 : 2	96
4	**1e** (4-CF_3_C_6_H_4_)	**2a** (OBn)	**3e**: 87	88 : 12	91
5	**1f** (4-FC_6_H_4_)	**2a** (OBn)	**3f**: 67	98 : 2	94
6	**1g** (4-CNC_6_H_4_)	**2a** (OBn)	**3g**: 57	92 : 8	87
7	**1h** (3,4-Cl_2_C_6_H_3_)	**2a** (OBn)	**3h**: 82	92 : 8	90
8	**1i** (C_6_H_5_)	**2a** (OBn)	**3i**: 79	98 : 2	94
9	**1j** (4-PhC_6_H_4_)	**2a** (OBn)	**3j**: 76	>99 : 1	96
10	**1k** (2-furyl)	**2a** (OBn)	**3k**: 48	95 : 5	96
11	**1l** (2-thienyl)	**2a** (OBn)	**3l**: 67	90 : 10	93
12^*e*^	**1m** (1-naphthyl)	**2a** (OBn)	**3m**: 99	97 : 3	99
13	**1n** (cyclohexyl)^*f*^	**2a** (OBn)	**3n**: 68	98 : 2	95
14	**1a** (2-BrC_6_H_4_)	**2b** (OEt)	**3o**: 94	>99 : 1	99
15	**1a** (2-BrC_6_H_4_)	**2c** (Me)	**3p**: 83	>99 : 1	96

^*a*^The reactions were carried out with **1** (0.1 mmol), **2a** (0.15 mmol), **CP5** (0.01 mmol) and 4 Å MS (30 mg) in DCM (0.5 mL) and toluene (0.5 mL) at rt for 12 h. Unless otherwise mentioned, the compounds **1** were *E*-isomers.

^*b*^Isolated yield using column chromatography.

^*c*^Regioselectivity ratios determined using crude ^1^H NMR spectroscopy; r.r. = regioselectivity ratio.

^*d*^Determined using chiral HPLC analysis.

^*e*^The absolute configuration of **3m** has been determined using X-ray diffraction as (1*S*, 5*R*).

^*f*^Compound **1n** was the mixture of *Z* and *E* isomers, *Z*/*E* = 1/1 based on ^1^H NMR analysis.

We next attempted to examine the asymmetric α-addition [3 + 2] cycloaddition reactions of the benzofuranones **1** and the γ-substituted allenoates **4** (Table 3). As for substrate **1b**, product **5b** was obtained in 91% yield, along with 84 : 16 r.r. and an 85% ee value (Table 3, entry 2). For these substrates with electron-rich substituents on their aromatic rings, spiro-cycloadducts **5c–5d** were obtained in relatively moderate yields but with high ee values and regioselectivities (Table 3, entries 3–4). The substrates **1e–1m** with various electron-poor substituents on their aromatic rings were more suitable for this reaction, affording the corresponding cycloadducts in good yields with 91%–99% ee values and 92 : 8 to >99 : 1 regioselectivity ratios (Table 3, entries 5–12). The aliphatic group is also suitable for this reaction (Table 3, entry 13). Some other allenic esters such as ethyl-, *tert*-butyl 4-phenylbuta-2,3-dienoates or benzyl penta-2,3-dienoate are also suitable for this asymmetric [3 + 2] cycloaddition, giving the corresponding products in 67–83% yields with 90–97% ee values and 95 : 5 to >99 : 1 regioselectivities (Table 3, entries 14–16). The absolute configuration of **5j** has been assigned using X-ray diffraction as 1*R*, 4*R*, 5*R*. The ORTEP drawing and the CIF data are summarized in the ESI.† 19

**Table 3:** Scope of the asymmetric [3 + 2] cycloaddition to afford cycloadducts **5b–5q**

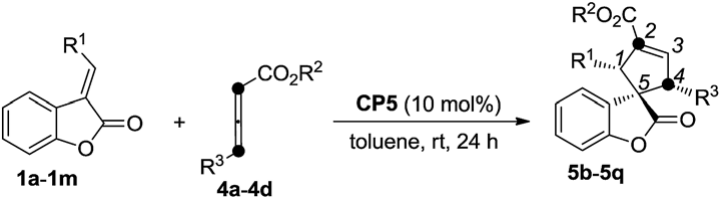					
Entry^*a*^	**1** (R^1^)	**4** (R^2^/R^3^)	Yield^*b*^ (%)	r.r.^*c*^	ee^*d*^ (%)
1	**1a** (2-BrC_6_H_4_)	**4a** (Bn/Ph)	**5a**: 96	>99 : 1	95
2	**1b** (4-BrC_6_H_4_)	**4a** (Bn/Ph)	**5b**: 91	84 : 16	85
3	**1c** (4-CH_3_C_6_H_4_)	**4a** (Bn/Ph)	**5c**: 72	92 : 8	99
4	**1d** (4-CH_3_OC_6_H_4_)	**4a** (Bn/Ph)	**5d**: 68	98 : 2	96
5	**1e** (4-F_3_C_6_H_4_)	**4a** (Bn/Ph)	**5e**: 92	99 : 1	92
6	**1f** (4-FC_6_H_4_)	**4a** (Bn/Ph)	**5f**: 78	95 : 5	99
7	**1g** (4-CNC_6_H_4_)	**4a** (Bn/Ph)	**5g**: 75	>99 : 1	99
8	**1h** (3,4-Cl_2_C_6_H_3_)	**4a** (Bn/Ph)	**5h**: 82	>92 : 8	99
9	**1i** (C_6_H_5_)	**4a** (Bn/Ph)	**5i**: 86	>99 : 1	99
10^*e*^	**1j** (4-PhC_6_H_4_)	**4a** (Bn/Ph)	**5j**: 83	>99 : 1	99
11	**1k** (2-furyl)	**4a** (Bn/Ph)	**5k**: 77	>99 : 1	99
12	**1m** (1-naphthyl)	**4a** (Bt/Ph)	**5l**: 73	>99 : 1	90
13	**1n** (cyclohexyl)^*f*^	**4a** (Bt/Ph)	**5m**: 92	>99 : 1	99
14	**1c** (4-CH_3_C_6_H_4_)	**4b** (Et/Ph)	**5n**: 67	95 : 5	90
15	**1c** (4-CH_3_C_6_H_4_)	**4c** (^*t*^Bu/Ph)	**5o**: 83	>99 : 1	97
16	**1c** (4-CH_3_C_6_H_4_)	**4d** (Bn/Me)	**5p**: 62	95 : 5	94

^*a*^The reactions were carried out with **1a** (0.1 mmol), **2a** (0.12 mmol), and **CP5** (0.01 mmol) in toluene (1.0 mL) at rt for 24 h. Unless otherwise mentioned, the compounds **1** were *E*-isomers.

^*b*^Isolated yield using column chromatography.

^*c*^Regioselectivity ratios determined using crude ^1^H NMR spectroscopy; r.r. = regioselectivity ratios.

^*d*^Determined using chiral HPLC analysis.

^*e*^The absolute configuration of **5j** has been determined using X-ray diffraction as (1*R*, 4*R*, 5*R*).

^*f*^Compound **1n** was a mixture of *Z* and *E* isomers, *Z*/*E* = 1/1 based on ^1^H NMR analysis.

It is noteworthy that this catalytic system can also be applied in the regioselective construction of spiroindolines5h,8a,^15^ in good yields, with high ee values and high regioselectivities (Scheme 4, eqn (1) and eqn (2)). The γ-addition [3 + 2] cycloadducts **7a** and **7b** were obtained in 78% and 98% yields, 96% and 98% ee values and 95 : 5 and >99 : 1 r.r., respectively. The α-addition [3 + 2] cycloadduct **8a** was formed in 89% yield, 99% ee value and 95 : 5 r.r. The enantioselective approach for the construction of spirocyclic oxindolic cyclopentanes based on a phosphine-mediated γ-addition has been reported by Marinetti's group.5*h* Furthermore, the preparations of carbocyclic amino acids have received great attention in medicinal chemistry recently due to their unique biological activities.13e,^16^ As illustrated in Scheme 4 (eqn (3)), the spiro-cycloadduct **10a** was obtained in 87% yield with a >99% ee value and a high regioselectivity using alkylidene azlactone **9a** (1.0 mmol) and the substituted allenoate **4a** (1.5 mmol) as the substrates. The reactions of other substrates with different aromatic rings also proceeded smoothly, affording the corresponding cycloadducts **10b–10f** in good yields with high ee values (>99% ee) and excellent regioselectivities. The ring-opened α-amino acid product **11** was easily obtained *via* treatment with 6 M HCl in high yield without the ee value diminishing (Scheme 4, eqn(3)).

**Scheme 4:**
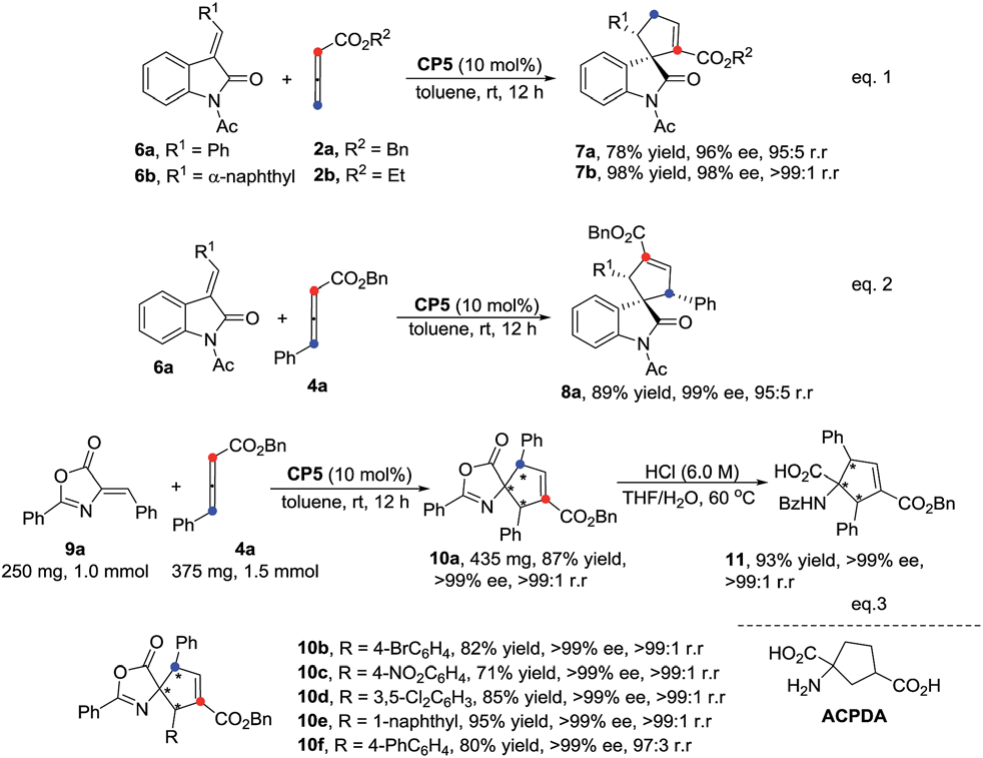
Further applications and transformations.

The plausible mechanisms for this phosphine-catalyzed [3 + 2] cycloaddition have been proposed in Scheme 5 on the basis of our experiments and previous literature.^1^,^2^ The reaction starts from the formation of a zwitterionic intermediate **A** between the allenoate (**2** or **4**) and phosphine. Intermediate **A** acts as a 1,3-dipole and undergoes a [3 + 2] cycloaddition with benzofuranone **1** to give a phosphrous ylide **B***via* γ-addition or **D***via* α-addition. For allenoate **2** (R^3^ = H), γ-addition is the main pathway. In contrast, allenoate **4** (R^3^ = aryl or alkyl group) mainly undergoes α-addition. Then, an intramolecular^1^,^2^ proton transfer is speculated to convert the phosphorus ylide **B** or **D** to another zwitterionic intermediate **C** or **E**, which, upon elimination of the phosphine catalyst, gives rise to the final cycloadduct **3** or **5**.

**Scheme 5:**
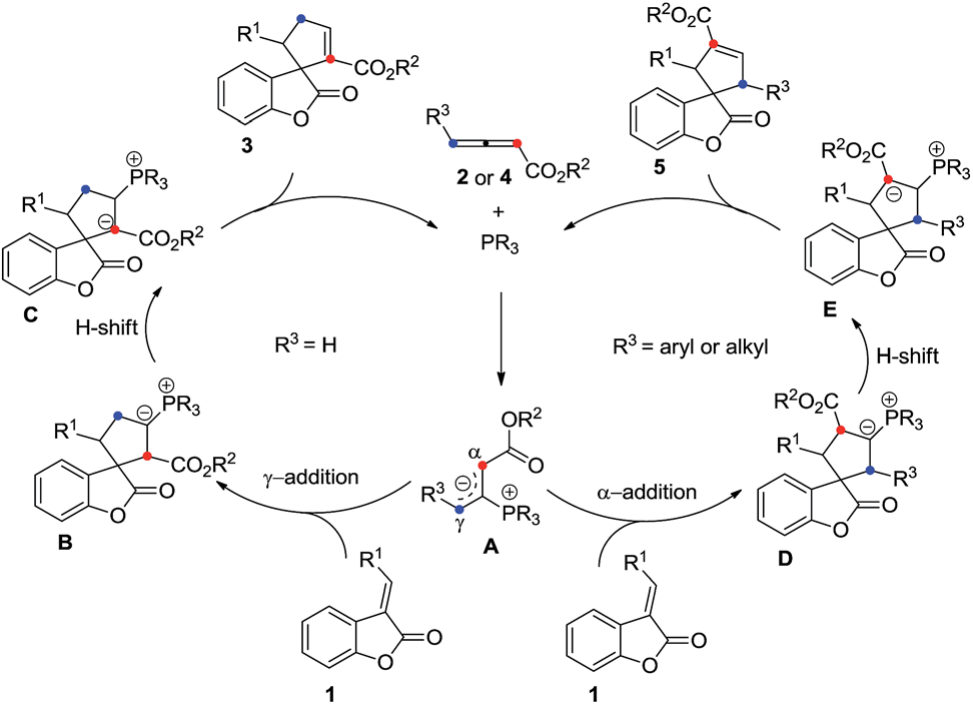
Plausible mechanism for the phosphine-catalyzed [3 + 2] cycloaddition.

The possible transition state of this asymmetric [3 + 2] cycloaddition is illustrated in Scheme 6 and may account for the stereochemical outcomes. The zwitterionic intermediate2s,^17^ derived from the chiral phosphine and allenoate could approach the benzofuranone **1** through either the *Re* face or *Si* face. Presumably, due to steric reasons, the zwitterionic intermediate (R^3^ = H) is more favored to attack the benzofuranone **1** from the *Si* face to give the corresponding product (Scheme 6, left), however, the zwitterionic intermediate (R^3^ = Ph or Me) is more favored to attack the benzofuranone **1** from the *Re* face to afford the corresponding product (Scheme 6, right).

**Scheme 6:**
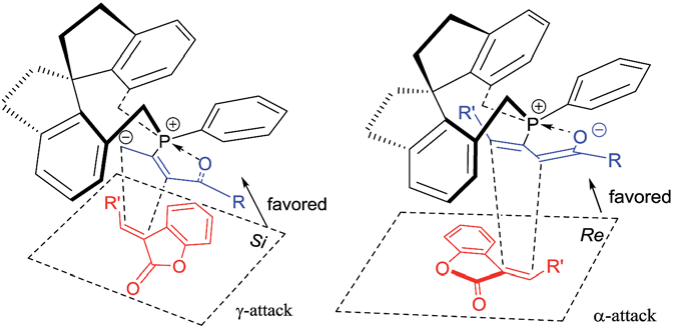
Plausible transition states of the γ-addition and α-addition.

In order to understand the regiochemical outcome of this reaction, we have done theoretical investigations on this [3 + 2] cycloaddition. All calculations have been performed at the mPW1K/6-31G(d) level with the Gaussian 09 program (see the ESI†). The calculation results indicated that the cycloaddition process is stepwise, which agrees with the previous theoretical studies by Yu’s group.^17^ For allenoate **2** (R^3^ = H), two intermediates, γ-**INT1** and γ-**INT2**, in the γ-addition mode are thermodynamically more favorable than those intermediates in the α-addition mode, which may account for why the γ-addition adducts were experimentally obtained as the major products. In contrast, using allenoate **4** (R^3^ = Ph) as a substrate, the energies of the intermediates γ-**INT1′** and γ-**INT2′** in the γ-addition mode are higher than those of α-**INT1′** and α-**INT2′** in the α-addition mode, probably due to the steric hindrance between the R^3^ substituents and benzofuranone in the intermediates γ-**INT1′** and γ-**INT2′**. Thus, the α-addition mode is more favorable in this case (see Schemes 7 and 8). All of these DFT calculations have been summarized in the ESI.†

**Scheme 7:**
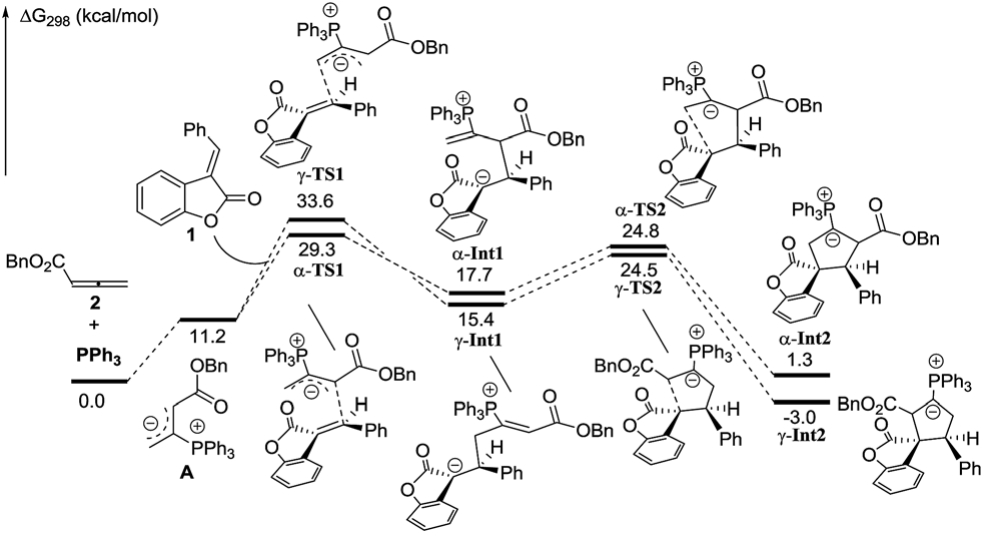
Theoretical investigations of the phosphine-catalyzed [3 + 2] cycloaddition of **1** and **2**.

**Scheme 8:**
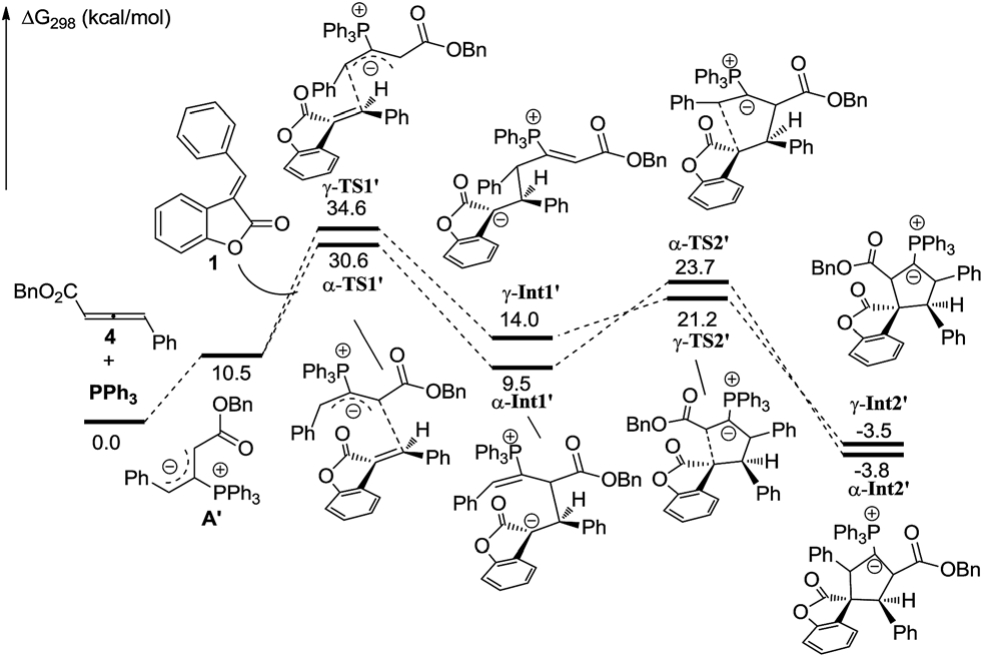
Theoretical investigations of the phosphine-catalyzed [3 + 2] cycloaddition of **1** and **4**.

In summary, we have reported the first example of the successful asymmetric and regioselective construction of 3,3’-spirocyclopentenebenzofuranones catalyzed by a chiral phosphine (*R*-SITCP) by employing benzofuranone and two types of allenic esters. Under the present catalytic system, γ-addition products and α-addition products can be obtained in 48–99% yields with 87–99% ee values and 88 : 12 to >19 : 1 regioselectivity ratios and in 62–96% yields with 85–99% ee values and 84 : 16 to >19 : 1 regioselectivity ratios, respectively. Moreover, this catalytic asymmetric [3 + 2] system can be also applied in the regioselective construction of spiro-oxindoles **7** and **8** as well as spiro-azlactone **10** which can be easily transformed to aspartic acid analogues.^18^ The DFT studies disclosed the origins of the regioselective outcomes for this phosphine-catalyzed [3 + 2] reaction. Further application of this type of reaction for the synthesis of more natural and natural-like spiro-compounds is ongoing.

## Supplementary Material

Supplementary information

Crystal structure data
