# Supplementary material for: Chiral phosphine-catalyzed tunable cycloaddition reactions of allenoates with benzofuranone-derived olefins for a highly regio-, diastereo- and enantioselective synthesis of spiro-benzofuranones
Source: Chem Sci. 2015 Sep 15;6(12):7319–25. doi: 10.1039/c5sc03135d (PMC5950759; doi:10.1039/c5sc03135d)
Supplement: Supplementary file 1 [file SC-006-C5SC03135D-s001.pdf]

Electronic Supplementary Material (ESI) for Chemical Science.
This journal is © The Royal Society of Chemistry 2015

Supporting information

Chiral Phosphine-Catalyzed Tunable Cycloaddition Reactions of Allenoates with Benzofuranone Derived Olefins for Highly Regio-, Diastereo- and Enantioselective Synthesis of Spiro-Benzofuranones

De Wang,[a] Guo-Peng Wang,[b] Yao-Liang Sun,[a] Shou-Fei Zhu,[b] Yin Wei,[a*] Qi-Lin Zhou[b*] and Min Shi,[a*]

[a] *State Key Laboratory of Organometallic Chemistry, Shanghai Institute of Organic Chemistry, Chinese Academy of Sciences, 354 Fenglin Lu, Shanghai 200032, China.*
Mshi@mail.sioc.ac.cn
[b] *State Key Laboratory and Institute of Elemento-Organic Chemistry, Collaborative Innovation Center of Chemical Science and Engineering (Tianjin), Nankai University, Tianjin 300071, China*
qlzhou@nankai.edu.cn

Contents:

1. General methods S2
2. General procedure for the synthesis of **3** S3
3. General procedure for the synthesis of **11** S3
4. Screen of chiral phosphines and optimization of the reaction conditions S4
5. Characterization and spectra charts containing HPLC traces for products **3** S5
6. Characterization and spectra charts containing HPLC traces for products **5** S44
7. Characterization and spectra charts for **7-8** and **10** S80
8. X-ray data of products **3m**, **5j** and **7b** S104
9. Theoretical investigations and computational details S106
10. Archive entries S109
11. References S135

1. General Methods: ^{1}H and ^{13}C NMR spectra were recorded at 400 and 100 MHz or 300 and 75 MHz by VARIAN, respectively. Low- and high-resolution mass spectra were recorded by EI or ESI method. The used organic solvents were dried by standard methods if it was necessary. Optical rotations were determined at 589 nm (sodium D line) by using a Perkin-Elmer-341 MC digital polarimeter; $[\alpha]_D$-values are given in unit of 10 deg^{-1} cm^2 g^{-1}. Chiral HPLC was performed on a SHIMADZU SPD-10A *vp* series with chiral columns (Chiralpak AD-H, OD-H and IC-H columns 4.6 x 250 mm, (Daicel Chemical Ind., Ltd.)). Commercially obtained reagents were used without further purification. All these reactions were monitored by TLC with silica-gel-coated plates. Flash column chromatography was carried out by using silica gel at increased pressure.

CP1-CP2,[1] **CP3-CP4**,[2] **CP5-CP8**,[3] were prepared according to the previously reported procedures.

All allenoates[4] and compounds **1a-1m**,[5] compound **6**,[6] compound **9**,[7] were prepared according to the previously reported procedures.

All the α- or γ-attack racemic products were carried out with triphenylphosphine (20 mol%) in toluene.

2. General procedure for the phosphine-catalyzed [3+2] annulation of benzofuranone with electron-deficient allenoate.

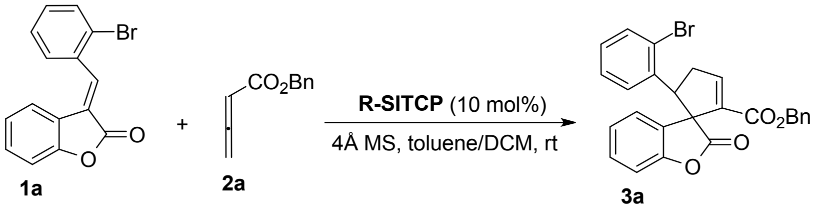

Benzofuranone **1a** (0.1 mmol), (R)-SITCP (0.01 mmol), 4Å MS (30 mg), anhydrous DCM (0.5 mL) and anhydrous toluene (0.5 mL) were added into a Schlenk tube, then allenoate **2a** (0.15 mmol) was added very slowly. The reaction mixture was stirred at room temperature for 12 h (TLC monitored) under argon atmosphere. The reaction mixture was then concentrated on a rotary evaporator under reduce pressure and the residue was subjected to purification by column chromatography (PE/AcOEt = 15/1~10/1) to afford the corresponding product **3a**.

3. General procedure for the synthesis of 11

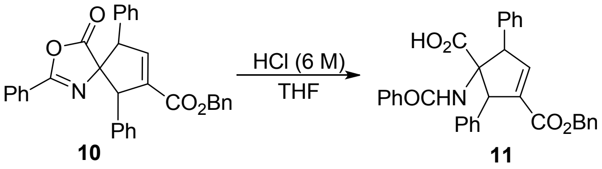

Compound **10** (100 mg, 0.2 mmol) was dissolved in THF (3.0 mL), then 6 M HCl (2 mL) was added. The solution was heated at 60 ^{o}C for 4 h. After cooling to room temperature, the aqueous solution was extracted with DCM and the combined organic layers were dried over Na_2SO_4. Then the crude product was purified by flash silica gel chromatography (DCM/EtOH = 20/1) to afford the product **11** as a white solid in 93% yield.

4. Table SI-1. Optimization of reaction conditions

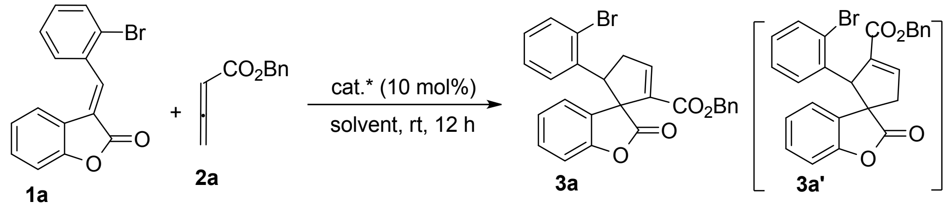

entry[a]	cat.*	solvent	T (°C)	yield[b] (%)	r.r.[c] (**3a**:**3a'**)	ee[c] (%)
1	**CP1**	toluene	25	37	90:10	8
2	**CP2**	toluene	25	32	88:12	20
3	**CP3**	toluene	25	26	86:14	14
4[d]	**CP4**	toluene	25	-	-	-
5	**CP5**	toluene	25	92	96:4	88
6	**CP6**	toluene	25	72	95:5	83
7	**CP7**	toluene	25	74	94:6	88
8	**CP8**	toluene	25	trace	92:8	13
9	**CP5**	DCM	25	58	>19:1	>99
10	**CP5**	THF	25	47	94:6	93
11	**CP5**	CH_3CN	25	22	72:28	94
12	**CP5**	toluene/DCM[e]	25	85	>19:1	91
13	**CP5**	toluene/DCM[f]	25	64	>19:1	98
14	***CP5***	*toluene/DCM[g]*	*25*	*78*	*>19:1*	*99*
15	**CP5**	toluene/DCM[g]	0	53	>19:1	99

[a] All reactions were carried out with **1a** (0.1 mmol), **2a** (0.15 mmol), catalyst (10 mol%) in solvent (1.0 mL); [b] Isolated yield; [c] Determined by ^{1}H NMR of crude product; Determined by HPLC; [d] disordered; [e] toluene/DCM = 4:1; [f] toluene/DCM = 1:1; [g] toluene/DCM = 1:1, 4Å MS (30 mg) was added as additive.

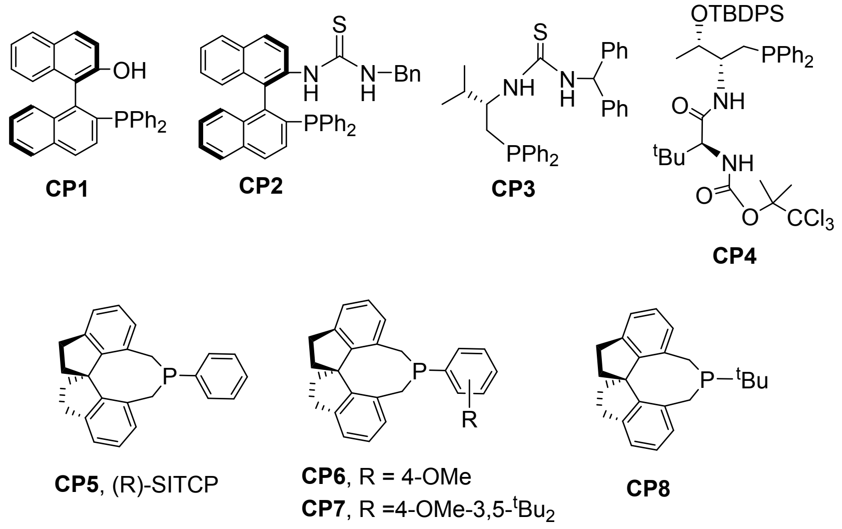

5. Characterization and spectra charts containing HPLC traces for products 3a-q.

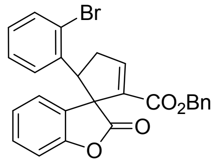

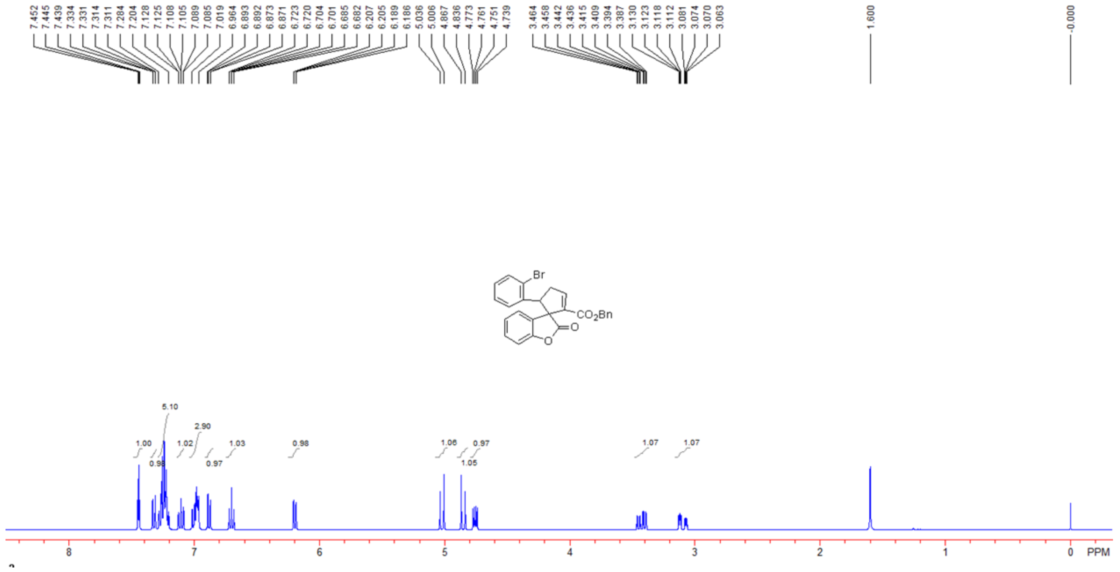

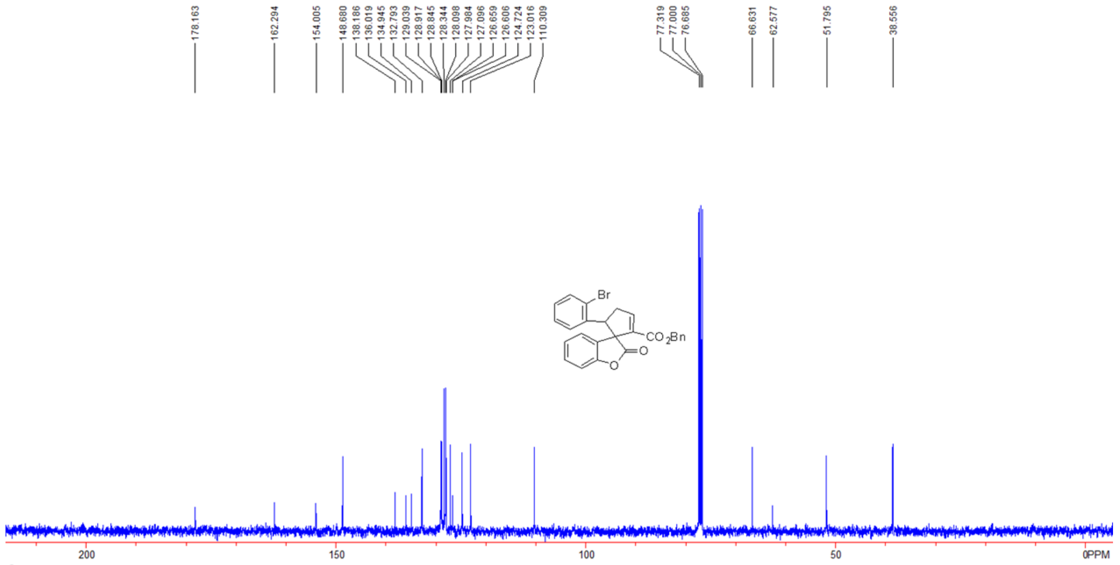

Benzyl 5'-(2-bromophenyl)-2-oxo-2H-spiro[benzofuran-3,1'-cyclopent[2]ene]-2'-carboxylate (3a)

White solid, 78% yield, 37 mg, Mp: 135-136 ºC. ^{1}H NMR (400 MHz, $CDCl_3$, TMS) δ 7.45 (t, J = 2.4 Hz, 1H), 7.32 (dd, J_1 = 1.2 Hz, J_2 = 8.0 Hz, 1H), 7.28-7.20 (m, 5H), 7.11 (dt, J_1 = 1.2 Hz, J_2 = 8.0 Hz, 1H), 7.02-6.96 (m, 3H), 6.88 (dd, J_1 = 0.8 Hz, J_2 = 8.0 Hz, 1H), 6.70 (dt, J_1 = 1.2 Hz, J_2 = 7.6 Hz, 1H), 6.20 (dd, J_1 = 1.2 Hz, J_2 = 7.6 Hz, 1H), 5.02 (d, J = 12.4 Hz, 1H), 4.85 (d, J = 12.4 Hz, 1H), 4.76 (dd, J_1 = 4.8 Hz, J_2 = 8.8 Hz, 1H), 3.43 (ddd, J_1 = 2.4 Hz, J_2 = 8.8 Hz, J_3 = 19.4 Hz, 1H), 3.10 (ddd, J_1 = 2.8 Hz, J_2 = 4.8 Hz, J_3 = 19.4 Hz, 1H); ^{13}C NMR (100 MHz, $CDCl_3$) δ 178.2,

162.3, 154.0, 148.7, 138.2, 136.0, 134.9, 132.8, 129.0, 128.9, 128.8, 128.3, 128.1, 128.0, 127.1, 126.7, 126.6, 124.7, 123.0, 110.3, 66.6, 62.6, 51.8, 38.6; IR (neat) ν 3005, 1800, 1716, 1636, 1462, 1275, 1260, 1109, 1070, 749, 668 cm^{-1}; HRMS Calcd. for $C_{26}H_{23}BrNO_4^{+1}$ $(M+NH_4)^+$: 492.0805, found: 492.0821. $[\alpha]^{20}_{D}$ = +114.5 (c 0.8, $CHCl_3$) for 99% ee; Enantiomeric excess was determined by HPLC with a Chiralcel IC-H column, Hexane/^{i}PrOH = 90/10, 0.8 mL/min, 230 nm, t_{minor} = 63.043 min, t_{major} = 55.443 min.

实验时间：2013-04-03, 10:52:57
谱图文件:I:\regio-and enantio\液相\wd-pri-14race-IC-90100.8230.org

实验者:
报告时间：2013-10-12, 15:51:49
积分方法:面积归一法

使用仪器类型:气相色谱　　检测器:FID　　进样器:分流

柱温:程序升温

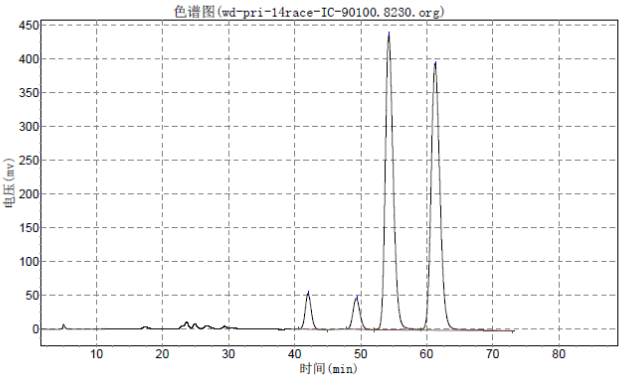

分析结果表

峰号	峰名	保留时间	峰高	峰面积	含量
1		42.057	53578.434	3480172.250	4.3206
2		49.457	46564.242	3572802.500	4.4356
3		54.257	432401.406	36702992.000	45.5660
4		61.323	393983.469	36793140.000	45.6779
总计			926527.551	80549106.750	100.0000

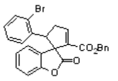

IC-H, n-Hexane:^{i}PrOH=90:10, 0.8 ml/min, 230nm

实验时间：2013-04-03, 12:22:50
谱图文件：I:\regio-and enantio\液相\wd-pri-14asy-IC-90100.8230.org

实验者：
报告时间：2013-10-12, 16:04:29
积分方法：面积归一法

使用仪器类型：气相色谱　　检测器：FID　　进样器：分流

柱温：程序升温

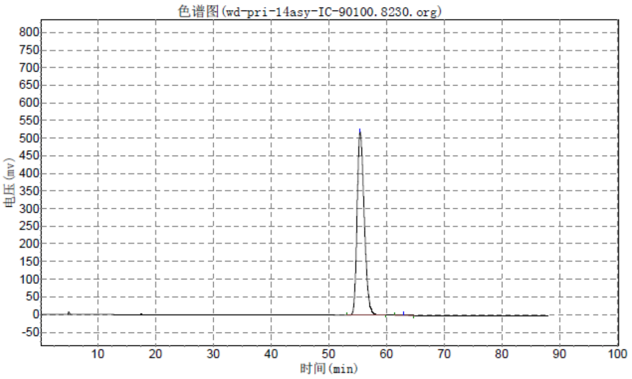

分析结果表

峰号	峰名	保留时间	峰高	峰面积	含量
1		55.443	519847.531	44619340.000	99.7685
2		63.043	1184.511	103519.328	0.2315
总计			521032.042	44722859.328	100.0000

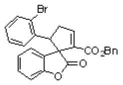

IC-H, n-Hexane:iPrOH=90:10, 0.8 ml/min, 230nm

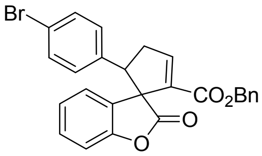

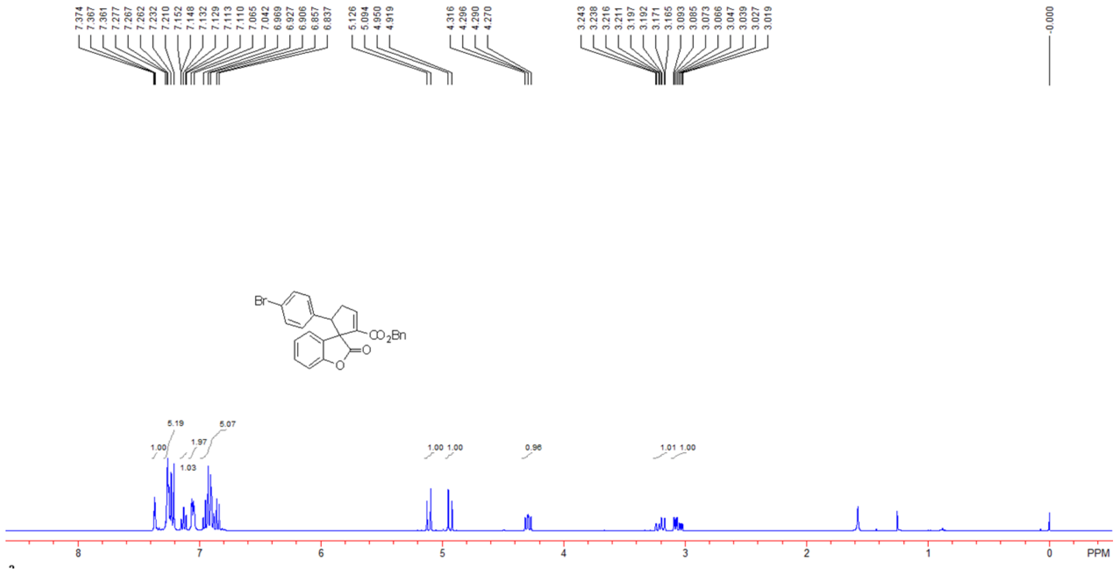

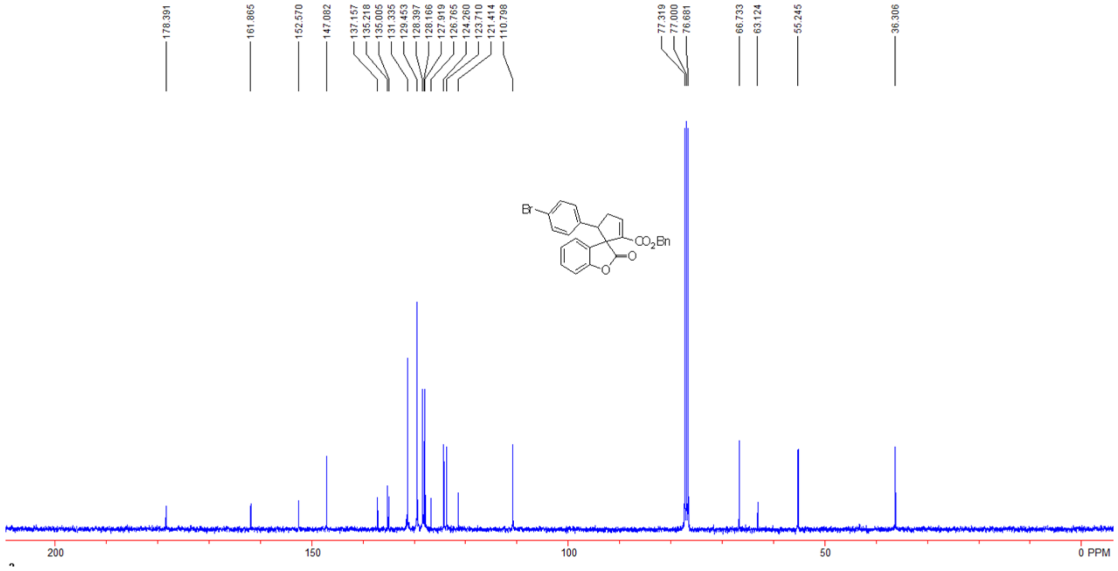

Benzyl 5'-(4-bromophenyl)-2-oxo-2H-spiro[benzofuran-3,1'-cyclopent[2]ene]-2'-carboxylate (3b)

A pale yellow solid, 92% yield, 43 mg, Mp: 77-79 ºC. ^{1}H NMR (400 MHz, $CDCl_3$, TMS) δ 7.37 (t, J = 2.4 Hz, 1H), 7.28-7.21 (m, 5H), 7.13 (dt, J_1 = 1.2 Hz, J_2 = 7.6 Hz, 1H), 7.07-7.04 (m, 2H), 6.97-6.84 (m, 5H), 5.11 (d, J = 12.4 Hz, 1H), 4.93 (d, J = 12.4 Hz, 1H), 4.29 (dd, J_1 = 8.0 Hz, J_2 = 10.4 Hz, 1H), 3.20 (ddd, J_1 = 2.0 Hz, J_2 = 10.4 Hz, J_3 = 18.4 Hz, 1H), 3.06 (ddd, J_1 = 3.2 Hz, J_2 = 8.0 Hz, J_3 = 18.4 Hz, 1H); ^{13}C NMR (100 MHz, $CDCl_3$) δ 178.4, 161.9, 152.6, 147.1, 137.2, 135.2, 135.0, 131.3, 129.5, 128.4, 128.2, 127.9, 126.8, 124.3, 123.7, 121.4, 110.8, 66.7, 63.1, 55.2, 36.3; IR (neat) ν 3005, 1800, 1712, 1617, 1462, 1275, 1260, 1071, 1009, 750, 697 cm^{-1}; HRMS Calcd. for $C_{26}H_{23}BrNO_4^{+1}$ $(M+NH_4)^+$: 492.0805, found: 492.0811. $[\alpha]^{20}_D$ = +110.9 (c 0.7, $CHCl_3$) for 95% ee; Enantiomeric excess was determined by HPLC with a Chiralcel IC-H column, Hexane/^{i}PrOH = 90/10, 0.8 mL/min, 230 nm, t_{minor} = 46.643 min, t_{major} = 48.822 min.

实验时间：2013-04-10, 01:06:57
谱图文件:I:\regio-and enantio\液相\wd-pri17-IC-80200.7230.org

实验者：
报告时间：2013-10-24, 20:35:21
积分方法:面积归一法

使用仪器类型:气相色谱　　检测器:FID　　进样器:分流

柱温:程序升温

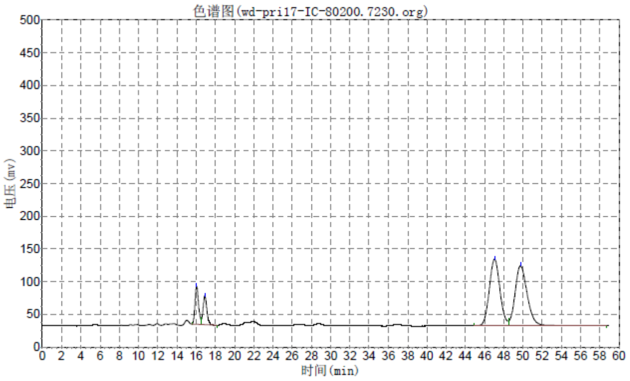

分析结果表

峰号	峰名	保留时间	峰高	峰面积	含量
1		16.057	59879.938	1636372.000	8.6658
2		16.923	45502.516	1332646.500	7.0573
3		47.090	101489.938	7840130.000	41.5191
4		49.757	91248.836	8074030.500	42.7578
总计			298121.227	18883179.000	100.0000

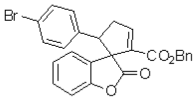

IC-H, Hexane:^{i}PrOH=80:20, 0.7 ml/min, 230

实验时间：2013-04-10, 00:06:47
谱图文件:I:\regio-and enantio\液相\wd-pri18-IC-80200.7230.org

实验者：
报告时间：2013-10-24, 20:37:06
积分方法:面积归一法

使用仪器类型:气相色谱　　检测器:FID　　进样器:分流

柱温:程序升温

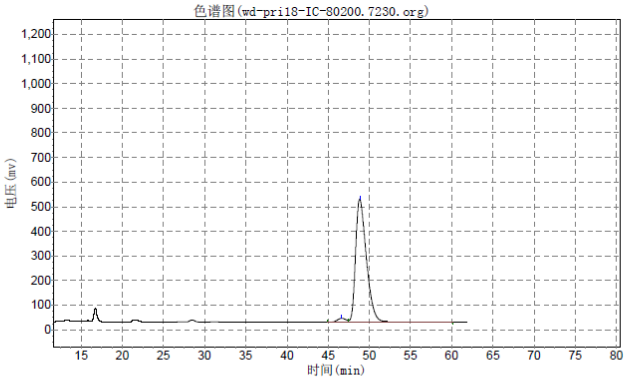

分析结果表

峰号	峰名	保留时间	峰高	峰面积	含量
1		46.643	16236.725	1184863.375	2.5356
2		48.822	500965.688	45543992.000	97.4644
总计			517202.412	46728855.375	100.0000

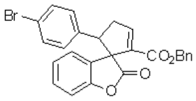

IC-H, Hexane:^{i}PrOH=80:20, 0.7 ml/min, 230

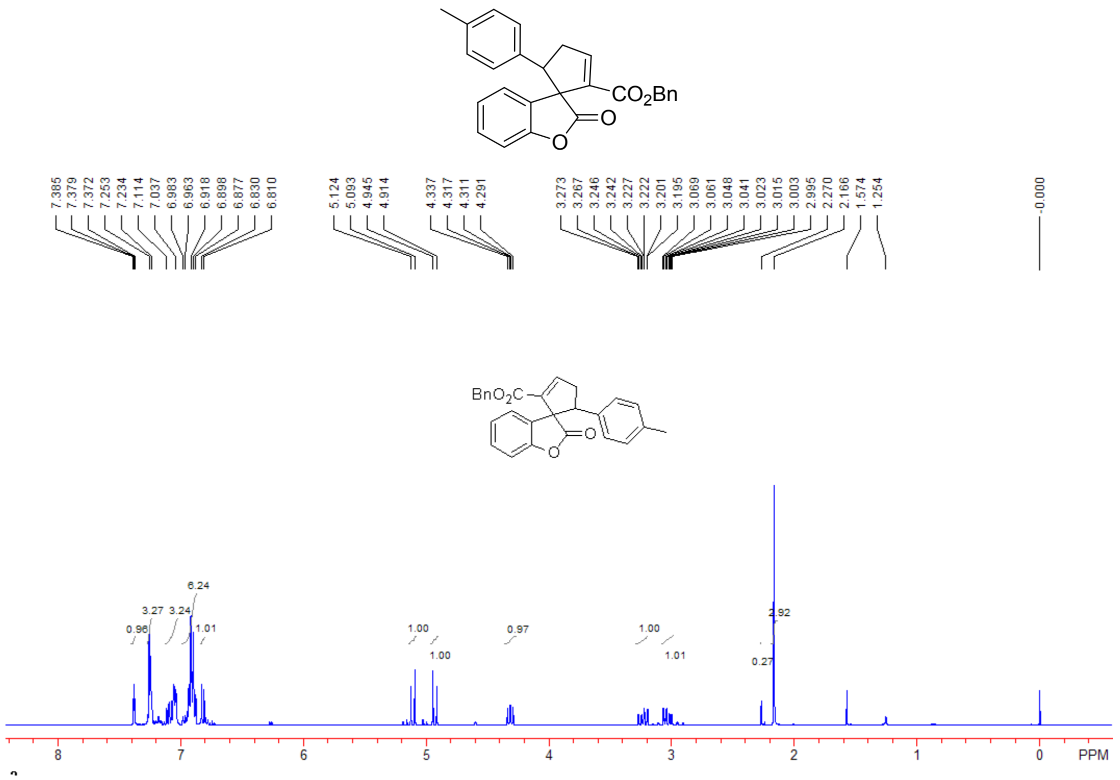

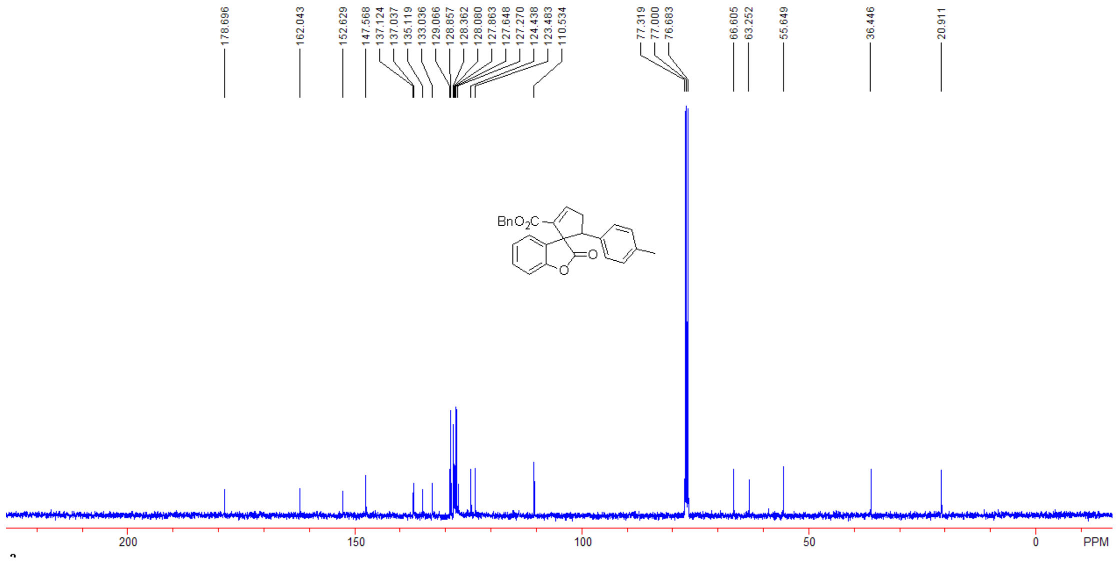

Benzyl 2-oxo-5'-(p-tolyl)-2H-spiro[benzofuran-3,1'-cyclopent[2]ene]-2'-carboxylate (3c)

Colorless solid, 76% yield, 31 mg, Mp: 91-92 ºC. ^{1}H NMR (400 MHz, $CDCl_3$, TMS) δ 7.38 (t, J = 2.4 Hz, 1H), 7.25-7.23 (m, 3H), 7.11-7.04 (m, 3H), 6.98-6.88 (m, 6H), 6.82 (d, J = 8.0 Hz, 1H), 5.11 (d, J = 12.4 Hz, 1H), 4.93 (d, J = 12.4 Hz, 1H), 4.31 (dd, J_1 = 8.0 Hz, J_2 = 10.4 Hz, 1H), 3.23 (ddd, J_1 = 2.4 Hz, J_2 = 10.4 Hz, J_3 = 18.0 Hz, 1H), 3.03 (ddd, J_1 = 3.2 Hz, J_2 = 8.0 Hz, J_3 = 18.0 Hz, 1H), 2.17 (s, 3H); ^{13}C NMR (100 MHz, $CDCl_3$) δ 178.7, 162.0, 152.6, 147.6, 137.1, 137.0, 135.1, 133.0, 129.1, 128.9, 128.4, 128.1, 127.9, 127.6, 127.3, 124.4, 123.5, 110.5, 66.6, 63.3, 55.6, 36.4, 20.9; IR (neat) ν 1797, 1711, 1461, 1230, 1110, 1070, 997, 750 cm^{-1}; HRMS

Calcd. for $C_{27}H_{26}NO_4^{+1}$ $(M+NH_4)^+$: 428.1856, found: 428.1865. $[\alpha]^{20}_D$ = +92.4 (c 0.9, $CHCl_3$) for 91% ee; Enantiomeric excess was determined by HPLC with a Chiralcel IC-H column, Hexane/^{i}PrOH = 80/20, 0.7 mL/min, 230 nm, t_{minor} = 53.148 min, t_{major} = 46.343 min.

N2000 数据工作站 1

实验时间：2013-03-30, 15:26:28
谱图文件:I:\regio-and enantio\液相\wd-pri-12race-IC-80200.7230.org

实验者：
报告时间：2013-10-12, 16:09:57
积分方法:面积归一法

使用仪器类型:气相色谱 检测器:FID 进样器:分流

柱温:程序升温

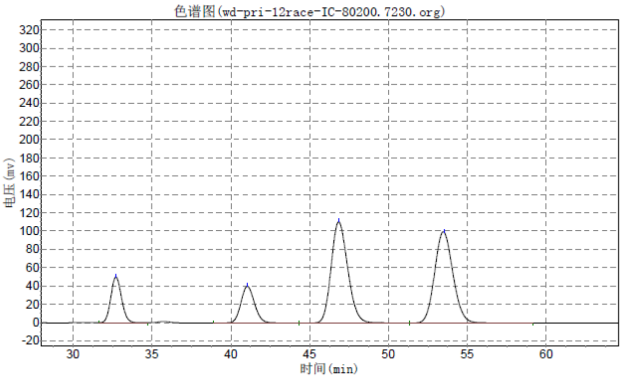

分析结果表

峰号	峰名	保留时间	峰高	峰面积	含量
1		32.732	49940.094	2399427.500	11.4394
2		41.065	39984.367	2397035.750	11.4280
3		46.865	110459.273	8115709.000	38.6922
4		53.532	99797.805	8062894.000	38.4404
总计			300181.539	20975066.250	100.0000

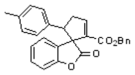

IC-H, n-Hexane:^{i}PrOH=80:20, 0.7 ml/min, 230nm

实验时间：2013-03-30, 16:30:26
谱图文件:I:\regio-and enantio\液相\wd-pri-13asy-IC-80200.7230.org

实验者：
报告时间：2013-10-12, 16:15:45
积分方法:面积归一法

使用仪器类型:气相色谱　　检测器:FID　　进样器:分流

柱温:程序升温

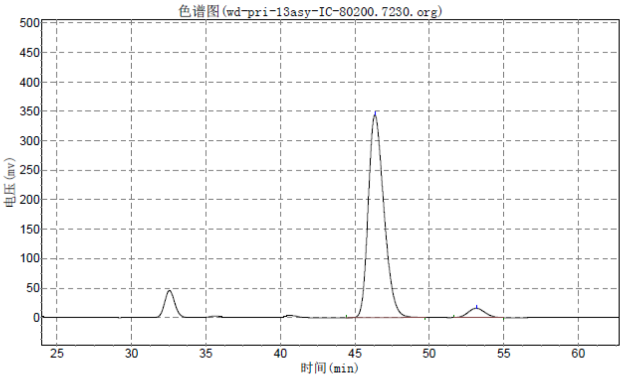

分析结果表

峰号	峰名	保留时间	峰高	峰面积	含量
1		46.343	344439.531	25131220.000	95.3503
2		53.148	15696.317	1225505.125	4.6497
总计			360135.849	26356725.125	100.0000

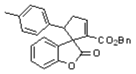

IC-H, *n*-Hexane:^{i}PrOH=80:20, 0.7 ml/min, 230nm

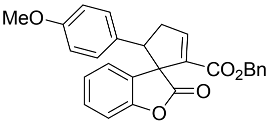

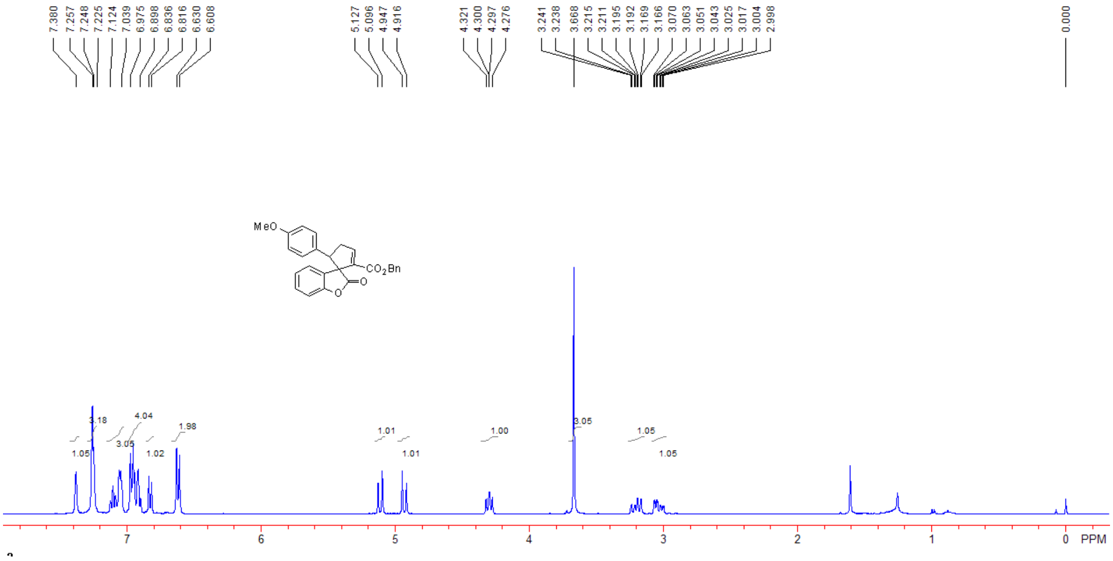

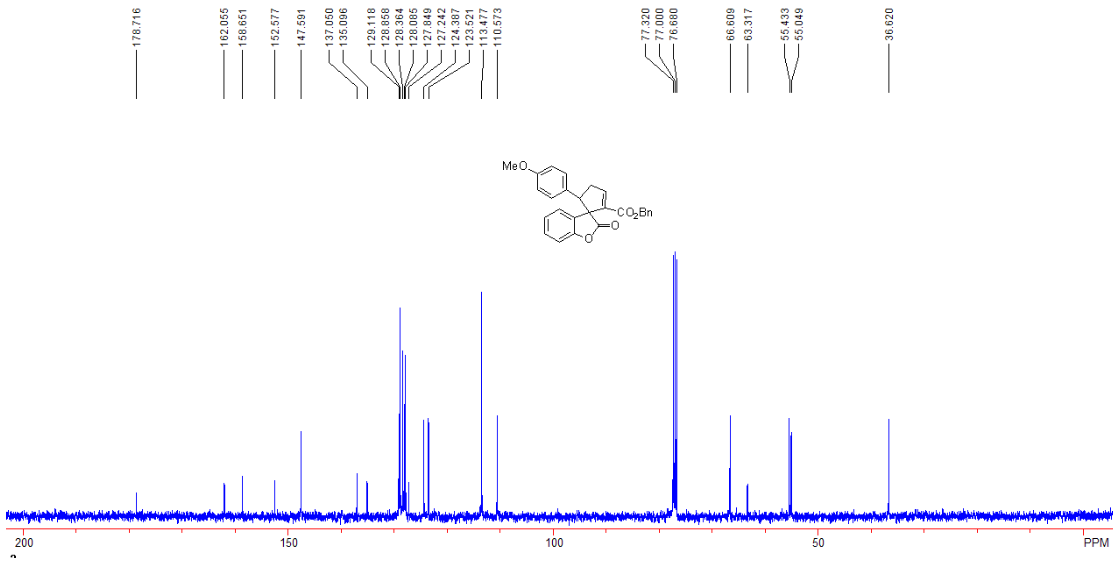

Benzyl 5'-(4-methoxyphenyl)-2-oxo-2H-spiro[benzofuran-3,1'-cyclopent[2]ene]-2'-carboxylate (3d)

A White solid, 72% yield, 30 mg, Mp: 73-75 ºC. ^{1}H NMR (400 MHz, $CDCl_3$, TMS) δ 7.38 (s, 1H), 7.26-7.23 (m, 3H), 7.12-7.04 (m, 3H), 6.98-6.90 (m, 4H), 6.83 (d, J = 8.0 Hz, 1H), 6.20 (d, J = 8.8 Hz, 2H), 5.11 (d, J = 12.4 Hz, 1H), 4.93 (d, J = 12.4 Hz, 1H), 4.30 (dd, J_1 = 8.4 Hz, J_2 = 9.6 Hz, 1H), 3.67 (s, 3H), 3.20 (ddd, J_1 = 1.2 Hz, J_2 = 9.6 Hz, J_3 = 18.4 Hz, 1H), 3.03 (ddd, J_1 = 2.8 Hz, J_2 = 8.4 Hz, J_3 = 18.4 Hz, 1H); ^{13}C NMR (100 MHz, $CDCl_3$) δ 178.7, 162.1, 158.7, 152.6, 147.6, 137.1, 135.1, 129.1, 128.9, 128.4, 128.1, 127.8, 127.2, 124.4, 123.5, 113.5, 110.6, 66.6, 63.3, 55.4, 55.0, 36.6; IR (neat) ν 2920, 1799, 1712, 1614, 1514, 1462, 1275, 1071, 764, 668 cm^{-1}; HRMS Calcd. for $C_{27}H_{26}NO_5^{+1}$ $(M+NH_4)^+$: 444.1805, found: 444.1804. $[\alpha]^{20}_D$ = +133.9 (c 0.8, $CHCl_3$) for 95% ee; Enantiomeric excess was determined by HPLC with a Chiralcel IC-H column, Hexane/^{i}PrOH = 80/20, 0.7 mL/min, 230 nm, t_{minor} = 41.242 min, t_{major} = 55.935 min.

N2000 数据工作站 1

实验时间：2013-04-25, 12:58:27
谱图文件:I:\regio-and enantio\液相\wd-pri21race-PC2-80200.7230.org

实验者:
报告时间：2013-10-14, 19:48:06
积分方法:面积归一法

使用仪器类型:气相色谱　　检测器:FID　　进样器:分流

柱温:程序升温

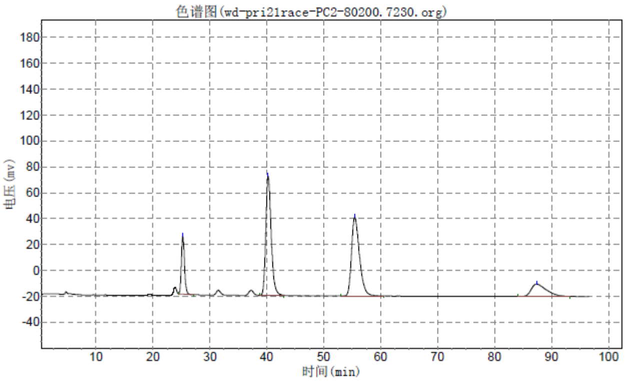

分析结果表

峰号	峰名	保留时间	峰高	峰面积	含量
1		25.238	44753.168	1702337.125	10.9893
2		40.242	91942.773	6079348.500	39.2449
3		55.445	60868.711	5934740.500	38.3114
4		87.365	9565.261	1774374.250	11.4544
总计			207129.913	15490800.375	100.0000

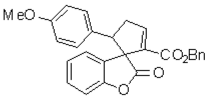

PC-2, *n*-Hexane:^{i}PrOH = 80:20, 0.7 ml/min, 230nm

N2000 数据工作站 1

实验时间：2013-04-25, 14:41:12
谱图文件:I:\regio-and enantio\液相\wd-pri21asy-PC2-80200.7230.org

实验者:
报告时间：2013-10-14, 19:46:28
积分方法:面积归一法

使用仪器类型:气相色谱　　检测器:FID　　进样器:分流

柱温:程序升温

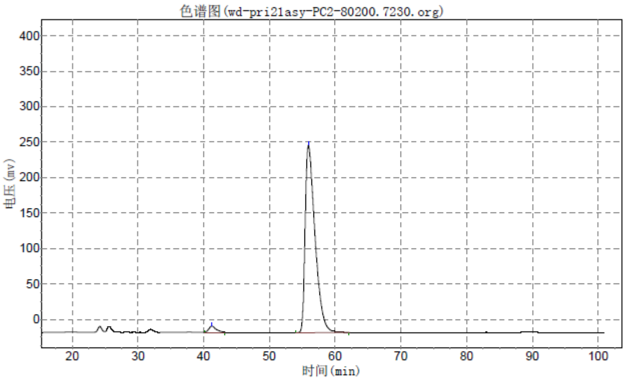

分析结果表

峰号	峰名	保留时间	峰高	峰面积	含量
1		41.242	8698.754	654230.938	2.3223
2		55.935	264208.000	27517668.000	97.6777
总计			272906.754	28171898.938	100.0000

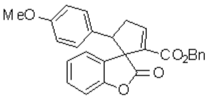

PC-2, *n*-Hexane:^{i}PrOH = 80:20, 0.7 ml/min, 230nm

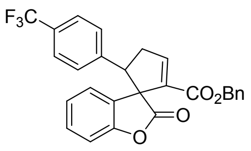
F3C
CO2Bn
O
O

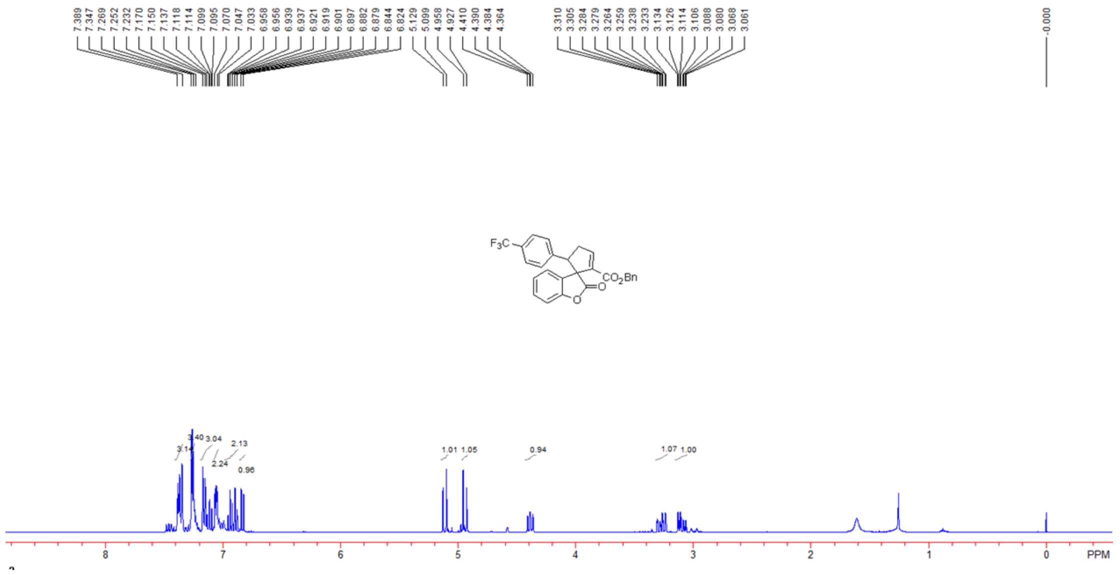
7.389
7.347
7.269
7.252
7.232
7.170
7.150
7.137
7.118
7.114
7.099
7.095
7.070
7.047
7.033
6.958
6.956
6.939
6.937
6.921
6.919
6.901
6.897
6.882
6.879
6.844
6.824
5.129
5.099
4.958
4.927
4.410
4.390
4.384
4.364
3.310
3.305
3.284
3.279
3.264
3.259
3.238
3.233
3.134
3.126
3.114
3.106
3.088
3.080
3.068
3.061
-0.000
3.14
3.40
3.04
2.24
2.13
0.96
1.01
1.05
0.94
1.07
1.00
8
7
6
5
4
3
2
1
0
PPM

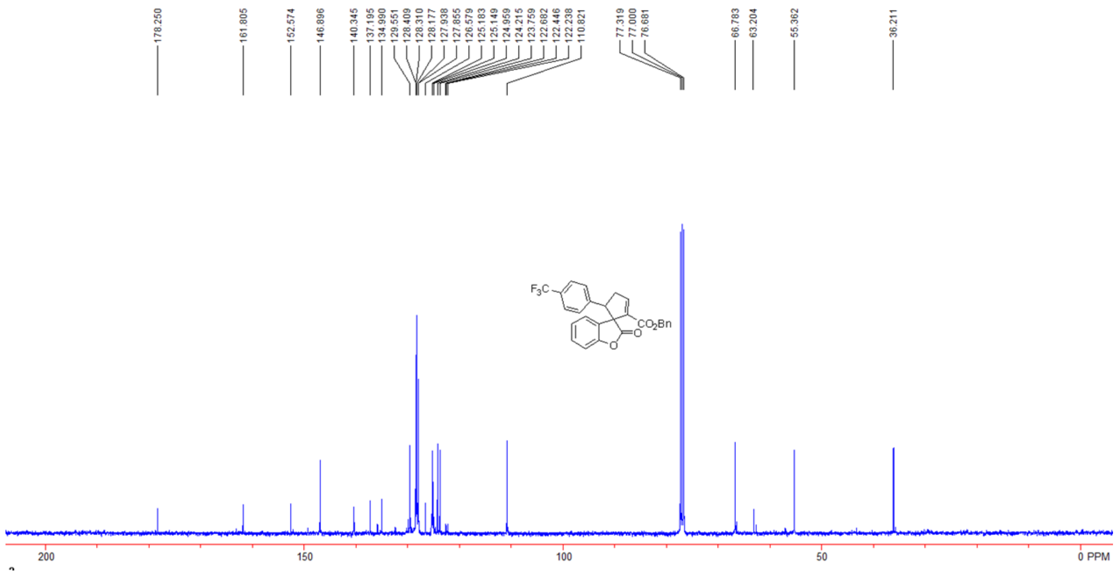
178.250
161.805
152.574
146.896
140.345
137.195
134.990
129.551
128.409
128.310
128.177
127.938
127.855
126.579
125.183
125.149
124.959
124.215
123.759
122.682
122.446
122.238
110.821
77.319
77.000
76.681
66.783
63.204
55.362
36.211
200
150
100
50
0 PPM

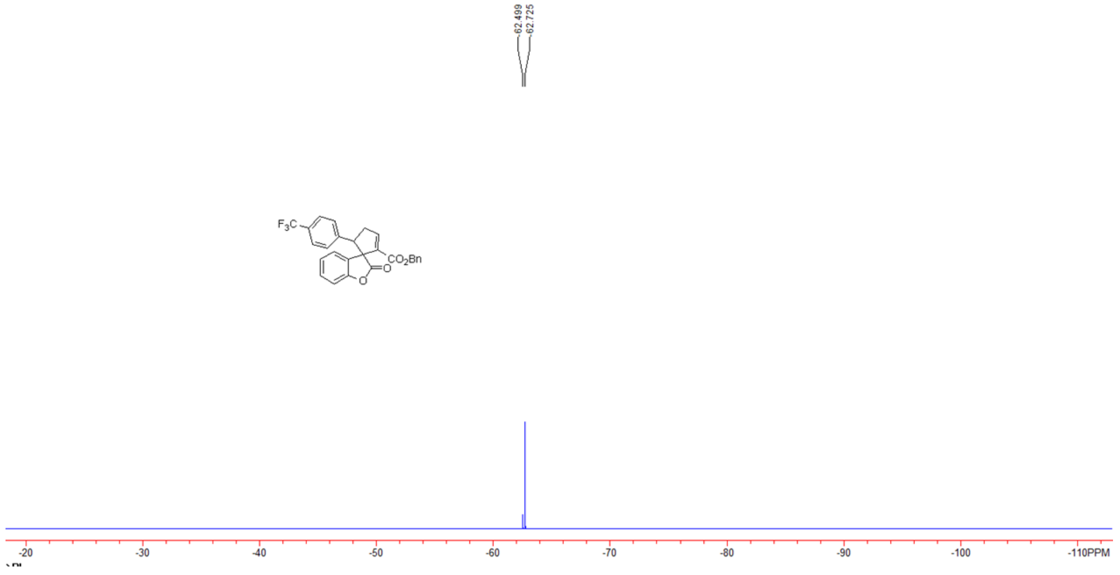
-62.499
-62.725
-20
-30
-40
-50
-60
-70
-80
-90
-100
-110PPM

Benzyl 2-oxo-5'-(4-(trifluoromethyl)phenyl)-2H-spiro[benzofuran-3,1'-cyclopent[2]ene]-2'-carboxylate (3e)

A pale yellow solid, 87% yield, 40 mg, Mp: 47-49 ºC. ^{1}H NMR (400 MHz, $CDCl_3$, TMS) δ 7.39-7.35 (m, 3H), 7.27-7.23 (m, 3H), 7.17-7.10 (m, 3H), 7.07-7.03 (m, 2H), 6.96-6.88 (m, 2H), 6.83 (d, J = 8.0 Hz, 1H), 5.11 (d, J = 12.4 Hz, 1H), 4.94 (d, J = 12.4 Hz, 1H), 4.39 (dd, J_1 = 8.0 Hz, J_2 = 10.4 Hz, 1H), 3.27 (ddd, J_1 = 2.0 Hz, J_2 = 10.4 Hz, J_3 = 18.4 Hz, 1H), 3.10 (ddd, J_1 = 3.2 Hz, J_2 = 8.0 Hz, J_3 = 18.4 Hz, 1H); ^{13}C NMR (100 MHz, $CDCl_3$) δ 178.3, 161.8, 152.6, 146.9, 140.3, 137.2, 135.0, 129.6, 128.4, 128.3, 128.2, 127.9, 127.8, 126.6, 125.2 (q, J = 3.4 Hz), 124.2, 123.8, 123.6 (q, J = 272.1 Hz), 122.6 (q, J = 23.6 Hz), 110.8, 66.8, 63.2, 55.4, 36.2; ^{19}F NMR (376 MHz, $CDCl_3$) δ -62.5 (minor), -62.7 (major); IR (neat) ν 2912, 1800, 1715, 1618, 1463, 1324, 1260, 1112, 1070, 750 cm^{-1}; HRMS Calcd. for $C_{27}H_{23}F_3NO_4^{+1}$ $(M+NH_4)^+$: 482.1574, found: 482.1564. $[\alpha]^{20}_D$ = +106.3 (c 1.1, $CHCl_3$) for 91% ee; Enantiomeric excess was determined by HPLC with a Chiralcel IC-H column, Hexane/^{i}PrOH = 90/10, 0.8 mL/min, 230 nm, t_{minor} = 35.468 min, t_{major} = 47.097 min.

N2000 数据工作站 1

实验时间：2013-05-23, 11:07:31
谱图文件:I:\regio-and enantio\液相\wd-pri-23rac-CF3-IC-90100.8230.org

实验者:
报告时间：2013-10-12, 16:21:18
积分方法:面积归一法

使用仪器类型:气相色谱　检测器:FID　进样器:分流

柱温:程序升温

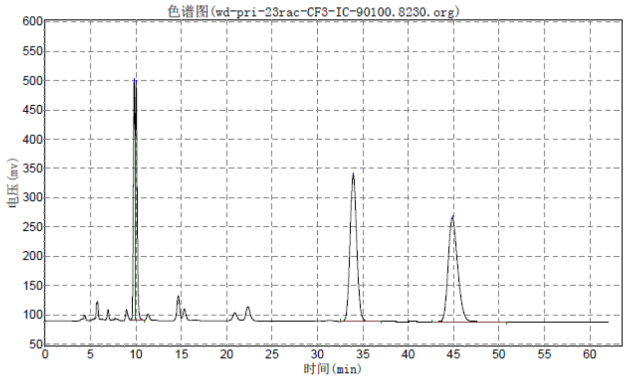

分析结果表

峰号	峰名	保留时间	峰高	峰面积	含量
1		9.798	407965.281	5095636.000	13.8949
2		10.032	408113.938	5641317.000	15.3828
3		33.982	251085.219	12986384.000	35.4115
4		44.915	177367.641	12949443.000	35.3108
总计			1244532.078	36672780.000	100.0000

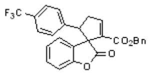

IC-H, *n*-Hexane: PrOH=90:10, 0.8 ml/min, 230nm

N2000 数据工作站 1

实验时间：2013-05-23, 12:45:09
谱图文件:I:\regio-and enantio\液相\wd-pri-23asy-CF3-IC-90100.8230.org

实验者：
报告时间：2013-10-12, 16:24:48
积分方法:面积归一法

使用仪器类型:气相色谱 检测器:FID 进样器:分流

柱温:程序升温

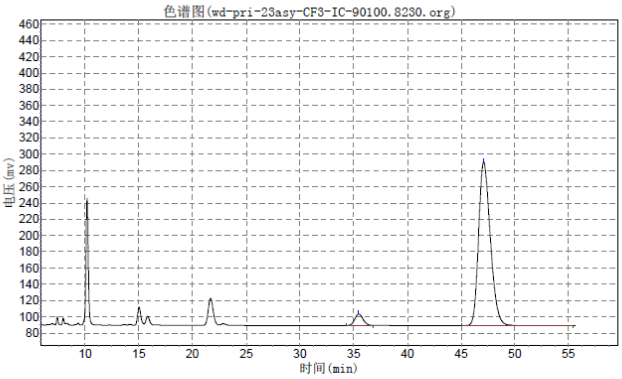

分析结果表

峰号	峰名	保留时间	峰高	峰面积	含量
1		35.468	13991.789	740431.000	4.6368
2		47.097	200852.609	15228287.000	95.3632
总计			214844.398	15968718.000	100.0000

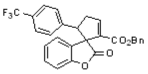

IC-H, *n*-Hexane: PrOH=90:10, 0.8 ml/min, 230nm

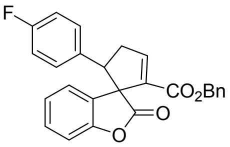

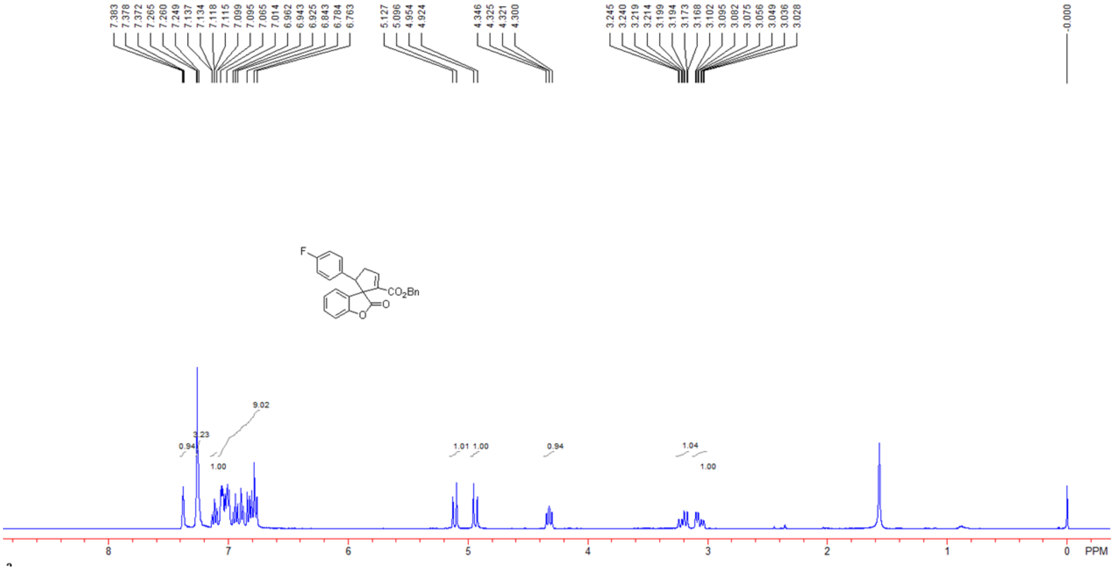

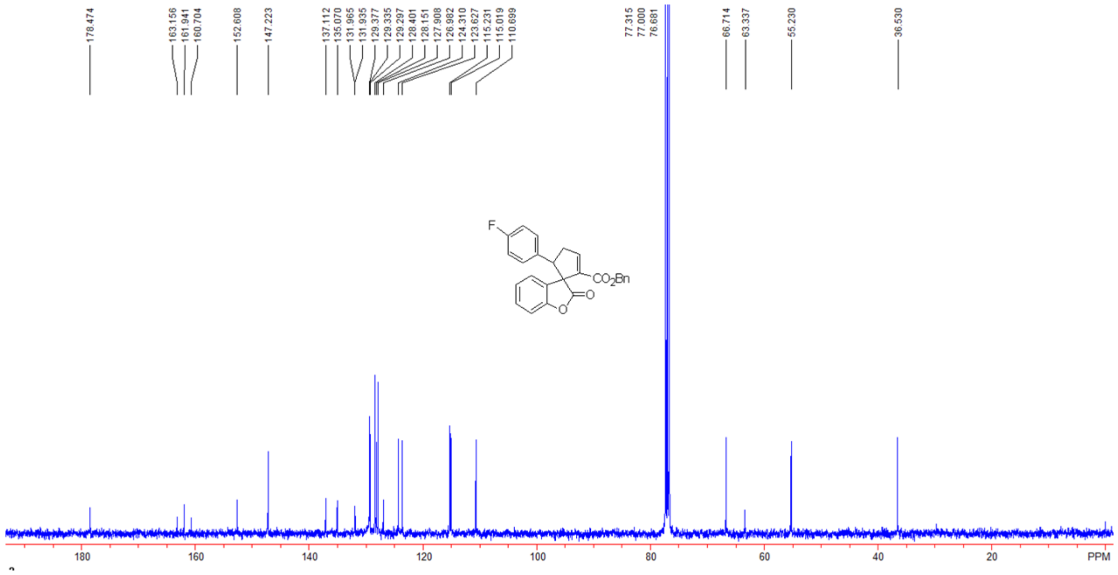

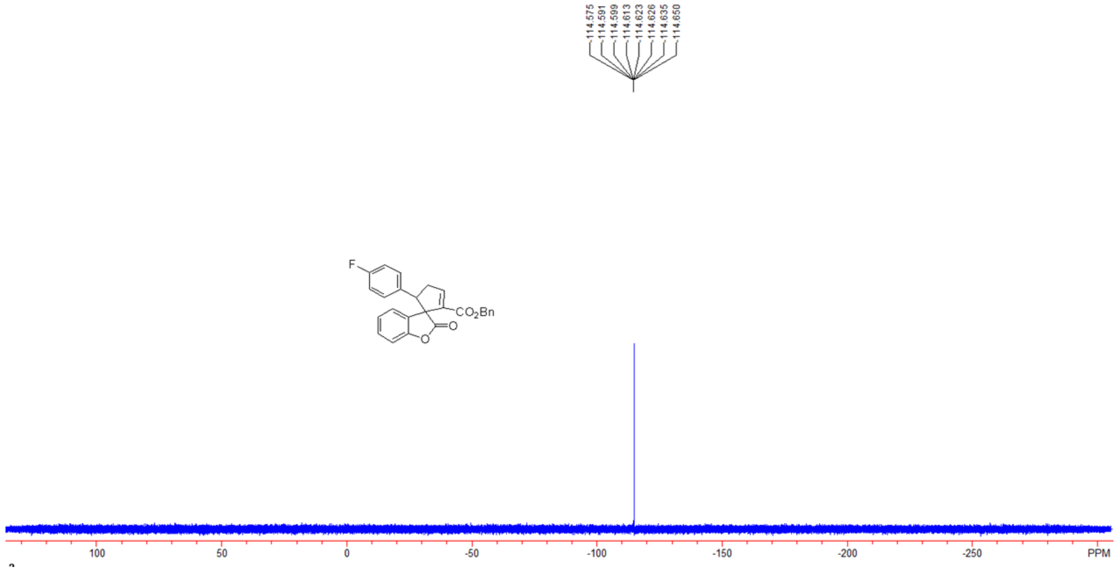

Benzyl 5'-(4-fluorophenyl)-2-oxo-2H-spiro[benzofuran-3,1'-cyclopent[2]ene]-2'-carboxylate (3f)

A pale yellow solid, 67% yield, 27 mg, Mp: 101-103 ºC. ^{1}H NMR (400 MHz, $CDCl_3$, TMS) δ 7.38 (t, J = 2.4 Hz, 1H), 7.27-7.25 (m, 3H), 7.12 (dt, J_1 = 1.2 Hz, J_2 = 7.6 Hz, 1H), 7.07-6.76 (m, 9H), 5.11 (d, J = 12.0 Hz, 1H), 4.94 (d, J = 12.0 Hz, 1H), 4.32 (dd, J_1 = 8.4 Hz, J_2 = 10.0 Hz, 1H), 3.21 (ddd, J_1 = 2.0 Hz, J_2 = 10.0 Hz, J_3 = 18.4 Hz, 1H), 3.07 (ddd, J_1 = 2.8 Hz, J_2 = 8.4 Hz, J_3 = 18.4 Hz, 1H); ^{13}C NMR (100 MHz, $CDCl_3$) δ 178.5, 161.94, 161.93 (d, J = 245.2 Hz), 152.6, 147.2, 137.1, 135.1, 132.0 (d, J = 3.0 Hz), 129.4 (d, J = 4.2 Hz), 129.3, 128.4, 128.2, 127.9, 127.0, 124.3, 123.6, 115.1 (d, J = 21.2 Hz), 110.7, 66.7, 63.3, 55.2, 36.5; ^{19}F NMR (376 MHz, $CDCl_3$) δ -114.58- -114.65 (m); IR (neat) ν 2922, 1799, 1712, 1617, 1510, 1462, 1228, 1133, 1071, 751 cm^{-1}; HRMS Calcd. for $C_{26}H_{23}FNO_4^{+1}$ (M+NH$_4$)$^+$: 432.1606, found: 432.1602. $[\alpha]^{20}_D$ = +102.9 (c 0.5, $CHCl_3$) for 94% ee; Enantiomeric excess was determined by HPLC with a Chiralcel PC-2

column, Hexane/^{i}PrOH = 90/10, 0.5 mL/min, 214 nm, t_{minor} = 69.688 min, t_{major} = 102.107 min.

实验时间：2013-08-15, 18:42:15
谱图文件:I:\regio-and enantio\液相\wd-18-93-rerace-PC-2-9010-0.5-214.org

实验者：
报告时间：2013-10-12, 16:31:40
积分方法:面积归一法

使用仪器类型:气相色谱 检测器:FID 进样器:分流

柱温:程序升温

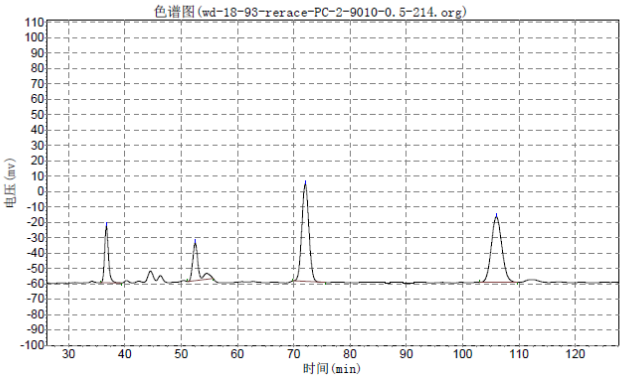

分析结果表

峰号	峰名	保留时间	峰高	峰面积	含量
1		36.677	37111.750	1738130.000	11.9254
2		52.453	24526.076	1750638.500	12.0113
3		72.035	63482.227	5524407.500	37.9034
4		106.012	42762.984	5561802.000	38.1599
总计			167883.037	14574978.000	100.0000

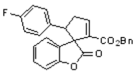

PC-2, *n*-Hexane:^{i}PrOH=90:10, 0.5 ml/min, 214nm

实验时间：2013-08-15, 16:30:42
谱图文件:I:\regio-and enantio\液相\wd-18-93-asy-PC-2-9010-0.5-214.org

实验者：
报告时间：2013-10-12, 16:36:22
积分方法:面积归一法

使用仪器类型:气相色谱 检测器:FID 进样器:分流

柱温:程序升温

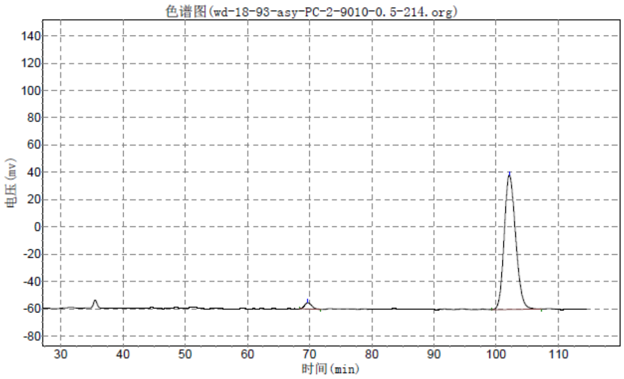

分析结果表

峰号	峰名	保留时间	峰高	峰面积	含量
1		69.688	4609.492	365659.938	2.8276
2		102.107	98526.297	12565955.000	97.1724
总计			103135.789	12931614.938	100.0000

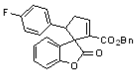

PC-2, *n*-Hexane:^{i}PrOH=90:10, 0.5 ml/min, 214nm

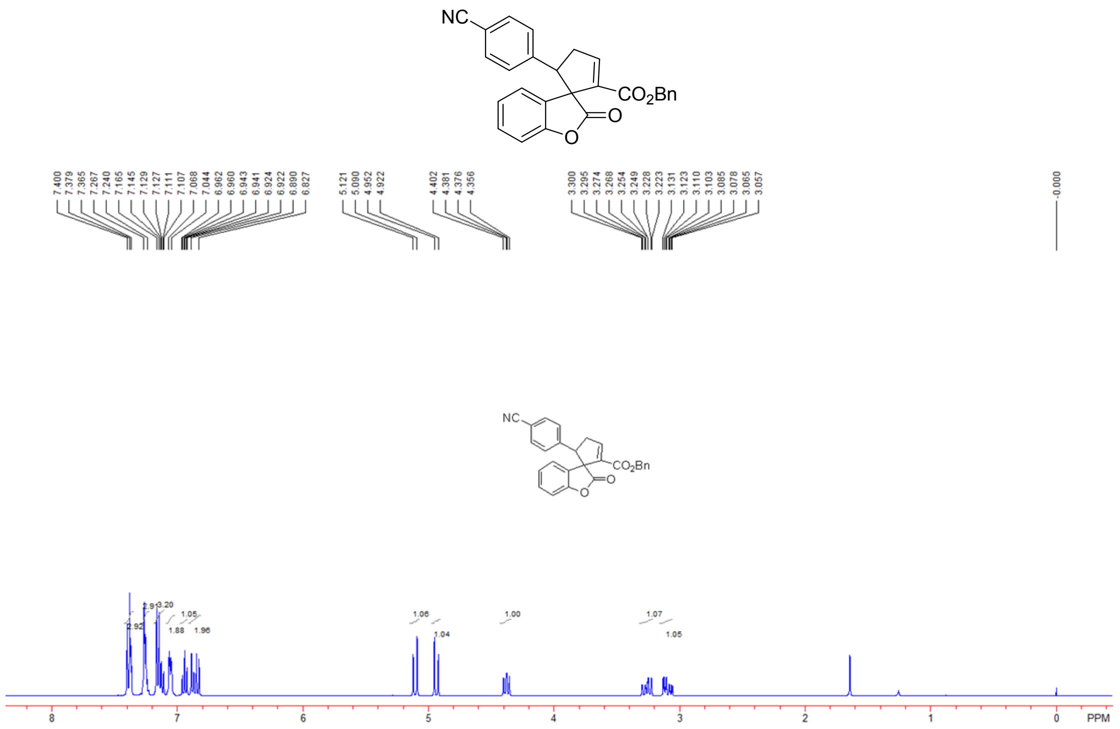

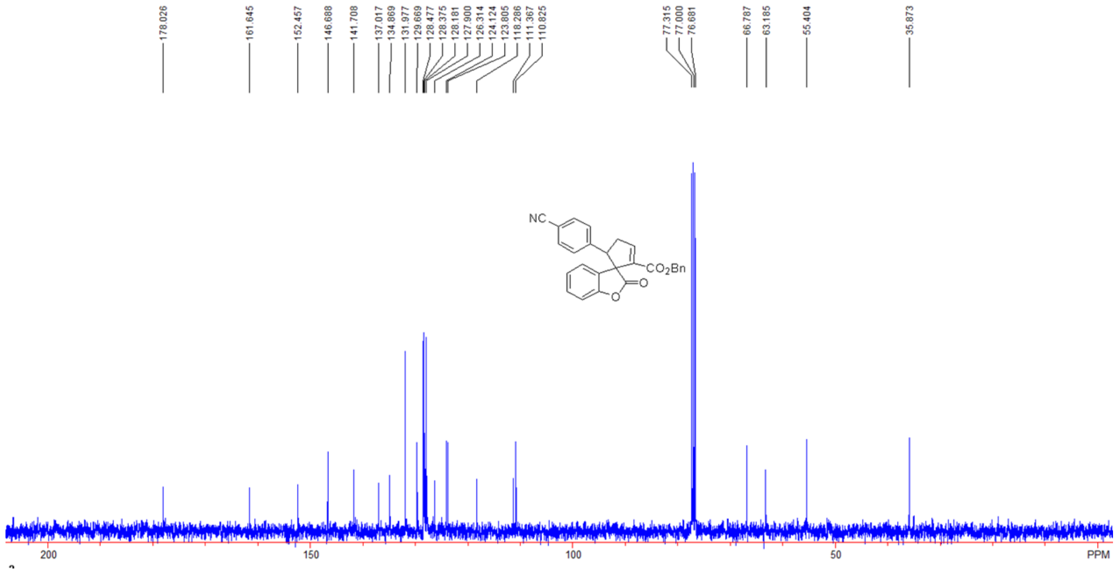

Benzyl 5'-(4-cyanophenyl)-2-oxo-2H-spiro[benzofuran-3,1'-cyclopent[2]ene]-2'-carboxylate (3g)

A pale yellow solid, 57% yield, 24 mg, Mp: 169-170 ºC. ^{1}H NMR (400 MHz, $CDCl_3$, TMS) δ 7.40-7.37 (m, 3H), 7.27-7.24 (m, 3H), 7.17-7.11 (m, 3H), 7.07-7.04 (m, 2H), 6.94 (dt, J_1 = 0.8 Hz, J_2 = 7.6 Hz, 1H), 6.89-6.83 (m, 2H), 5.11 (d, J = 12.4 Hz, 1H), 4.94 (d, J = 12.4 Hz, 1H), 4.38 (dd, J_1 = 8.4 Hz, J_2 = 10.4 Hz, 1H), 3.26 (ddd, J_1 = 2.0 Hz, J_2 = 10.4 Hz, J_3 = 18.4 Hz, 1H), 3.09 (ddd, J_1 = 3.2 Hz, J_2 = 8.4 Hz, J_3 = 18.4 Hz, 1H); ^{13}C NMR (100 MHz, $CDCl_3$) δ 178.0, 161.6, 152.5, 146.7, 141.7, 137.0, 134.9, 132.0, 129.7, 128.5, 128.4, 128.2, 127.9, 126.3, 124.1, 123.8,

118.3, 111.4, 110.8, 66.8, 63.2, 55.4, 35.9; IR (neat) ν 3005, 1798, 1709, 1462, 1275, 1260, 1071, 876, 749 cm^{-1}; HRMS Calcd. for $C_{27}H_{23}N_2O_4^{+1}$ $(M+NH_4)^+$: 439.1652, found: 439.1654. $[\alpha]^{20}_D$ = +107.8 (c 0.3, $CHCl_3$) for 87% ee; Enantiomeric excess was determined by HPLC with a Chiralcel IC-H column, Hexane/^{i}PrOH = 70/30, 0.7 mL/min, 214 nm, t_{minor} = 38.430 min, t_{major} = 55.355 min.

N2000 数据工作站 1

实验时间：2013-07-01, 22:23:13
谱图文件:I:\regio-and enantio\液相\wd-pri-27rac-CN-IC-70300.7214.org

实验者：
报告时间：2013-10-12, 16:45:16
积分方法:面积归一法

使用仪器类型:气相色谱 检测器:FID 进样器:分流

柱温:程序升温

色谱图(wd-pri-27rac-CN-IC-70300.7214.org)

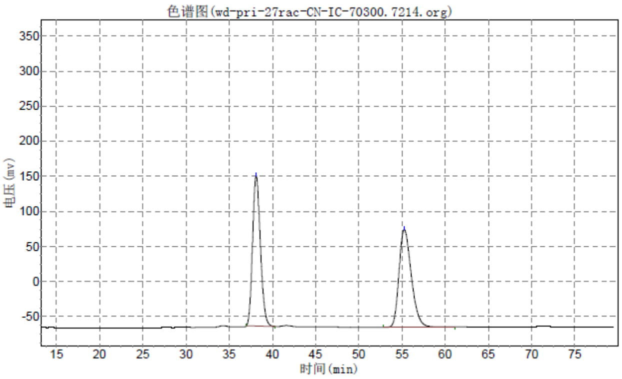

分析结果表

峰号	峰名	保留时间	峰高	峰面积	含量
1		38.115	213695.422	13483629.000	49.6716
2		55.252	139058.906	13661917.000	50.3284
总计			352754.328	27145546.000	100.0000

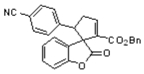

IC-H, n-Hexane: PrOH=70:30, 0.7 ml/min, 214nm

实验时间：2013-07-01, 23:37:15
谱图文件:I:\regio-and enantio\液相\wd-pri-27asy-CN-IC-70300.7214.org

实验者：
报告时间：2013-10-12, 16:48:10
积分方法:面积归一法

使用仪器类型:气相色谱 检测器:FID 进样器:分流

柱温:程序升温

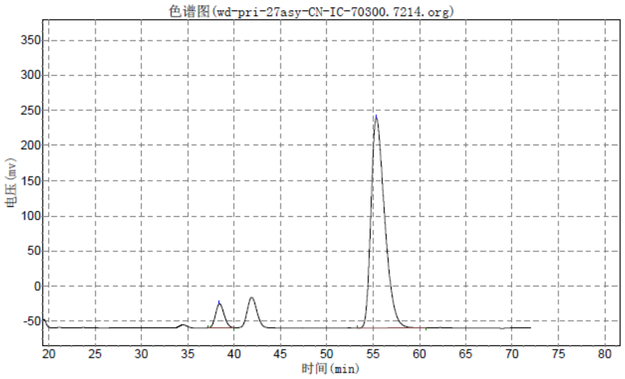

分析结果表

峰号	峰名	保留时间	峰高	峰面积	含量
1		38.430	34108.629	2173086.500	6.6163
2		55.355	298809.656	30671550.000	93.3837
总计			332918.285	32844636.500	100.0000

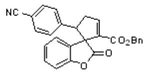

IC-H, *n*-Hexane: PrOH=70:30, 0.7 ml/min, 214nm

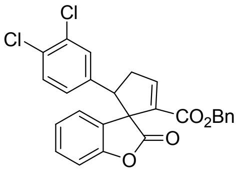

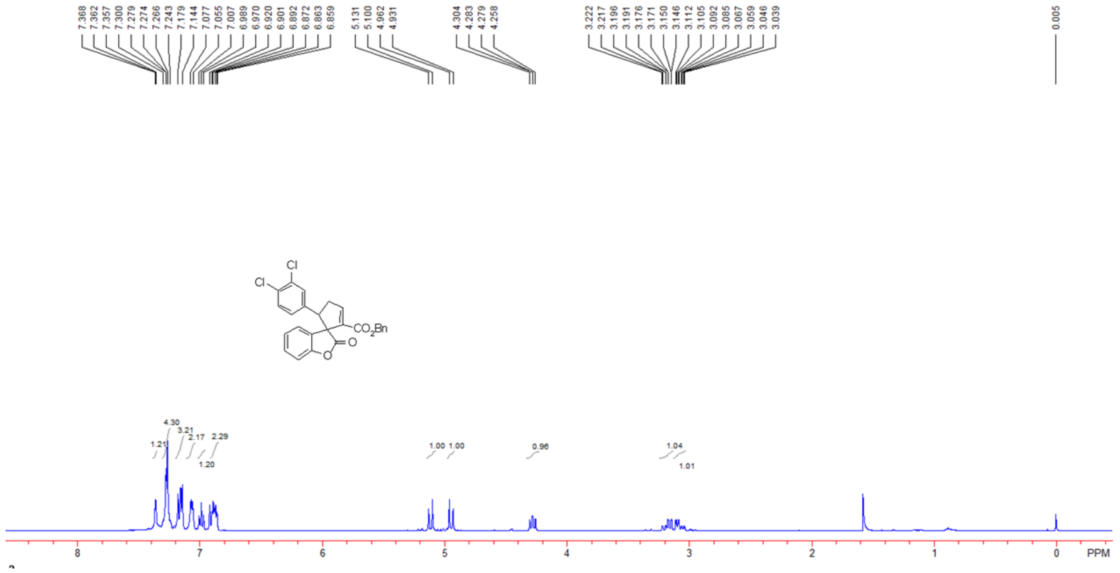

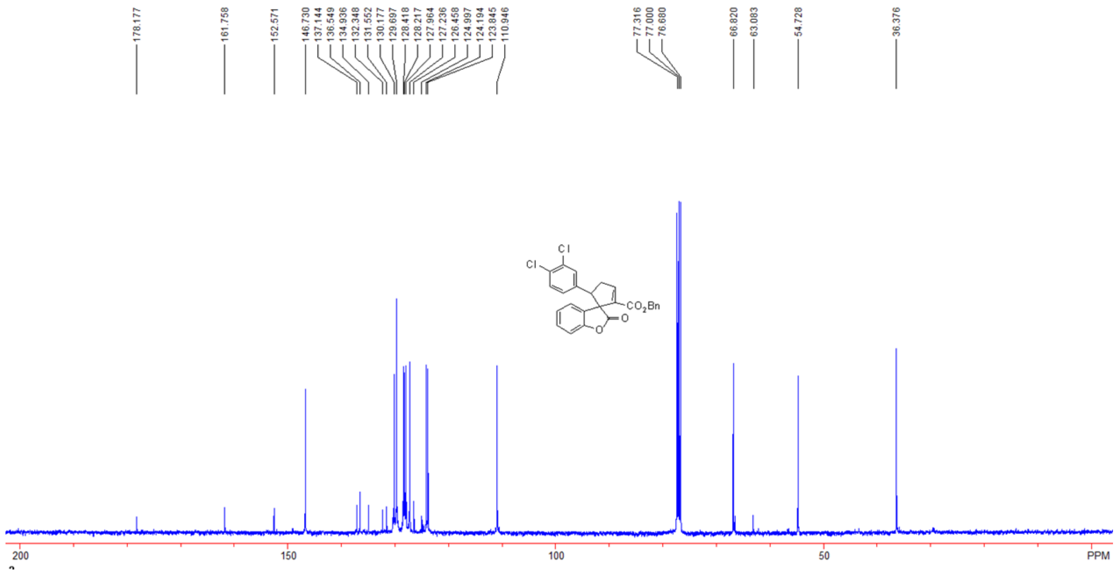

Benzyl 5'-(3,4-dichlorophenyl)-2-oxo-2H-spiro[benzofuran-3,1'-cyclopent[2]ene]-2'-carboxylate (3h)

A pale yellow solid, 82% yield, 38 mg, Mp: 61-62 ºC. ^{1}H NMR (400 MHz, $CDCl_3$, TMS) δ 7.36 (t, J = 2.0 Hz, 1H), 7.30-7.24 (m, 4H), 7.18-7.14 (m, 3H), 7.08-7.06 (m, 2H), 6.99 (t, J = 7.6 Hz, 1H), 6.92-6.86 (m, 2H), 5.12 (d, J = 12.4 Hz, 1H), 4.95 (d, J = 12.4 Hz, 1H), 4.28 (dd, J_1 = 8.4 Hz, J_2 = 10.0 Hz, 1H), 3.18 (ddd, J_1 = 2.0 Hz, J_2 = 10.0 Hz, J_3 = 18.4 Hz, 1H), 3.08 (ddd, J_1 = 2.8 Hz, J_2 = 8.4 Hz, J_3 = 18.4 Hz, 1H); ^{13}C NMR (100 MHz, $CDCl_3$) δ 178.2, 161.8, 152.6, 146.7, 137.1, 136.5, 134.9, 132.3, 131.6, 130.2, 129.7, 128.4, 128.2, 128.0, 127.2, 126.5, 125.0, 124.2, 123.8, 110.9, 66.8, 63.1, 54.7, 36.4; IR (neat) ν 3005, 1800, 1716, 1462, 1275, 1260, 1111, 1071, 749, 668 cm^{-1}; HRMS Calcd. for $C_{26}H_{22}Cl_2NO_4^{+1}$ $(M+NH_4)^+$: 482.0920, found: 482.0913. $[\alpha]^{20}_D$ = +120.7 (c 0.5, $CHCl_3$) for 90% ee; Enantiomeric excess was determined by HPLC with a Chiralcel PC-2 column, Hexane/^{i}PrOH = 90/10, 0.7 mL/min, 214 nm, t_{minor} = 45.960 min, t_{major} = 64.750 min.

实验时间：2013-08-16, 11:52:30
谱图文件:I:\regio-and enantio\液相\wd-18-92-race-PC-2-9010-0.7-214.org

实验者:
报告时间：2013-10-12, 16:50:47
积分方法:面积归一法

使用仪器类型:气相色谱 检测器:FID 进样器:分流

柱温:程序升温

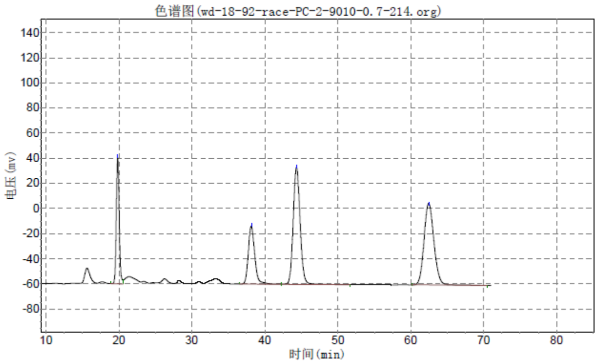

分析结果表

峰号	峰名	保留时间	峰高	峰面积	含量
1		19.848	100063.008	2803035.250	15.5285
2		38.182	46226.281	2899649.250	16.0637
3		44.382	93151.672	6207101.000	34.3866
4		62.582	64546.492	6141131.500	34.0212
总计			303987.453	18050917.000	100.0000

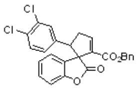

PC-2, *n*-Hexane:^{i}PrOH=90:10, 0.7 ml/min, 214nm

N2000 数据工作站 1

实验时间：2013-08-16, 13:40:44
谱图文件:I:\regio-and enantio\液相\wd-18-92-asy-PC-2-9010-0.7-214.org

实验者:
报告时间：2013-10-12, 16:54:37
积分方法:面积归一法

使用仪器类型:气相色谱 检测器:FID 进样器:分流

柱温:程序升温

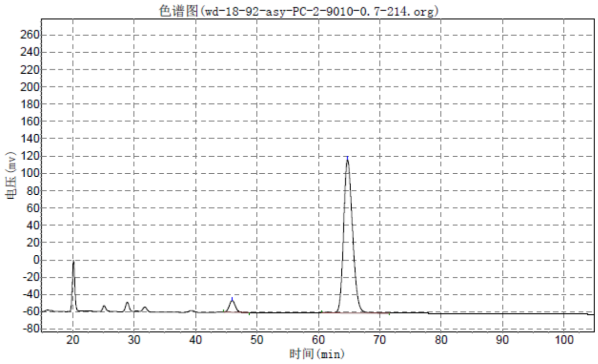

分析结果表

峰号	峰名	保留时间	峰高	峰面积	含量
1		45.960	13650.747	929350.438	4.9507
2		64.750	177145.219	17842802.000	95.0493
总计			190795.966	18772152.438	100.0000

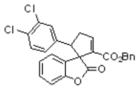

PC-2, *n*-Hexane:^{i}PrOH=90:10, 0.7 ml/min, 214nm

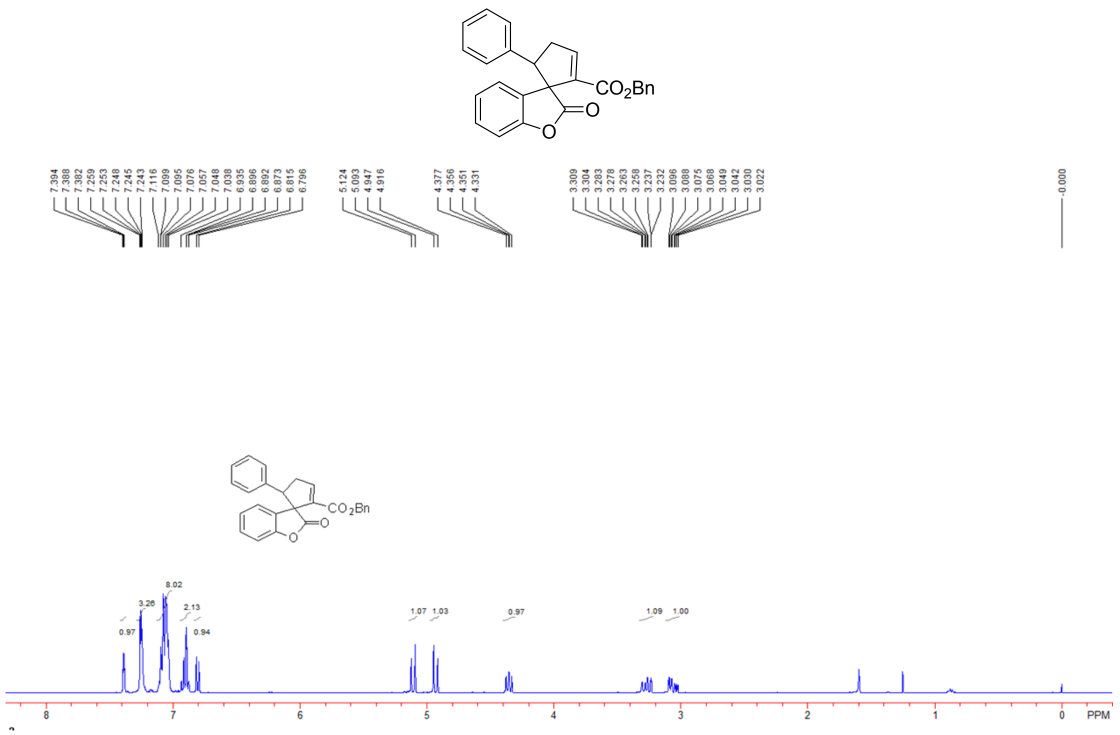

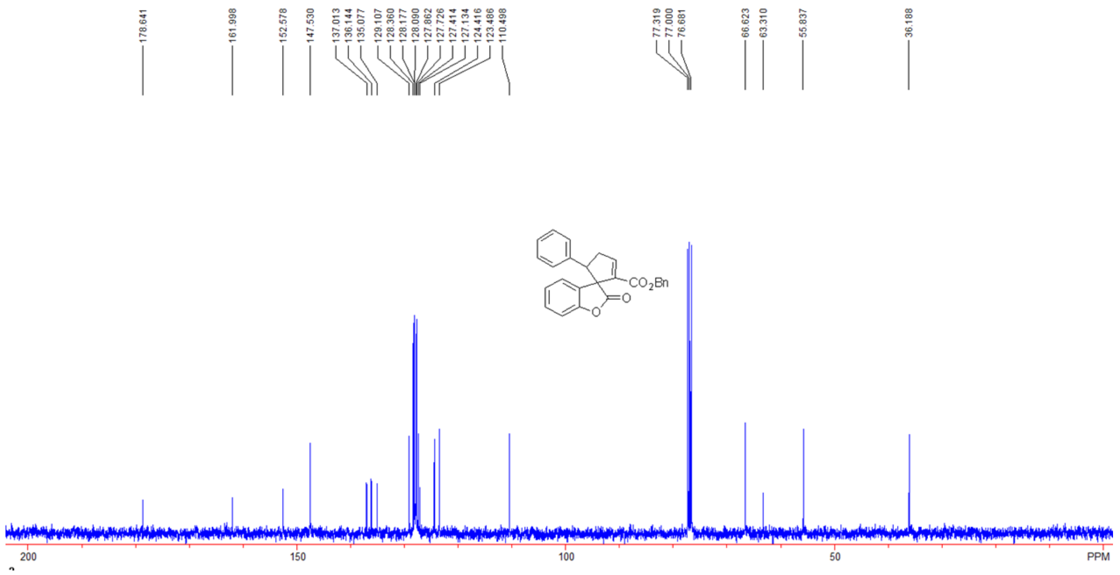

Benzyl 2-oxo-5'-phenyl-2H-spiro[benzofuran-3,1'-cyclopent[2]ene]-2'-carboxylate (3i)

A white solid, 79% yield, 31 mg, Mp: 128-130 ºC. ^{1}H NMR (400 MHz, $CDCl_3$, TMS) δ 7.39 (t, J = 2.4 Hz, 1H), 7.26-7.24 (m, 3H), 7.12-7.04 (m, 8H), 6.94-6.87 (m, 2H), 6.81 (d, J = 7.6 Hz, 1H), 5.11 (d, J = 12.4 Hz, 1H), 4.93 (d, J = 12.4 Hz, 1H), 4.35 (dd, J_1 = 8.4 Hz, J_2 = 10.4 Hz, 1H), 3.27 (ddd, J_1 = 2.0 Hz, J_2 = 10.4 Hz, J_3 = 18.4 Hz, 1H), 3.06 (ddd, J_1 = 3.2 Hz, J_2 = 8.4 Hz, J_3 = 18.4 Hz, 1H); ^{13}C NMR (100 MHz, $CDCl_3$) δ 178.6, 162.0, 152.6, 147.5, 137.0, 136.1, 135.1, 129.1, 128.4, 128.2, 128.1, 127.9, 127.7, 127.4, 127.1, 124.4, 123.5, 110.5, 66.6, 63.3, 55.8, 36.2; IR (neat) ν 2920, 1799, 1712, 1617, 1462, 1243, 1110, 1070, 876, 752 cm^{-1}; HRMS Calcd. for $C_{26}H_{24}NO_4^{+1}$ $(M+NH_4)^+$: 414.1700, found: 414.1696. $[\alpha]^{20}_D$ = +124.1 (c 0.4, $CHCl_3$) for 94% ee;

Enantiomeric excess was determined by HPLC with a Chiralcel IC-H column, Hexane/^{i}PrOH = 80/20, 0.6 mL/min, 214 nm, t_{minor} = 40.527 min, t_{major} = 35.810 min.

N2000 数据工作站 1

实验时间：2013-06-27, 12:53:44
谱图文件:I:\regio-and enantio\液相\wd-18-83A-rac-IC80200.6214.org

实验者：
报告时间：2013-10-14, 21:12:56
积分方法:面积归一法

使用仪器类型:气相色谱 检测器:FID 进样器:分流

柱温:程序升温

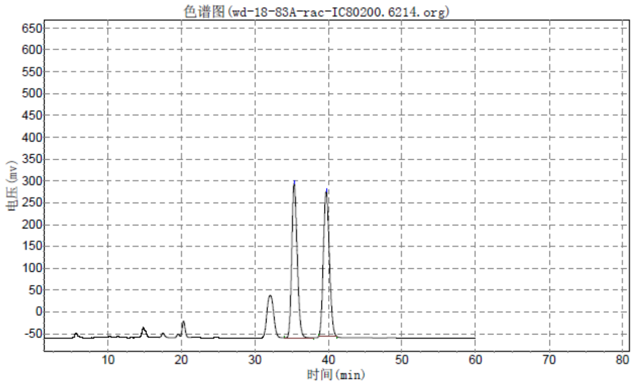

分析结果表

峰号	峰名	保留时间	峰高	峰面积	含量
1		35.332	353855.031	18270412.000	49.3306
2		39.677	331736.969	18766266.000	50.6694
总计			685592.000	37036678.000	100.0000

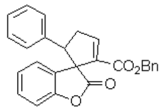

IC-H, *n*-Hexane:^{i}PrOH = 80:20, 0.6 ml/min, 214 nm

实验时间：2013-06-27, 14:22:29
谱图文件:I:\regio-and enantio\液相\wd-18-83B-asy-IC80200.6214.org

实验者：
报告时间：2013-10-14, 21:16:22
积分方法:面积归一法

使用仪器类型:气相色谱 检测器:FID 进样器:分流

柱温:程序升温

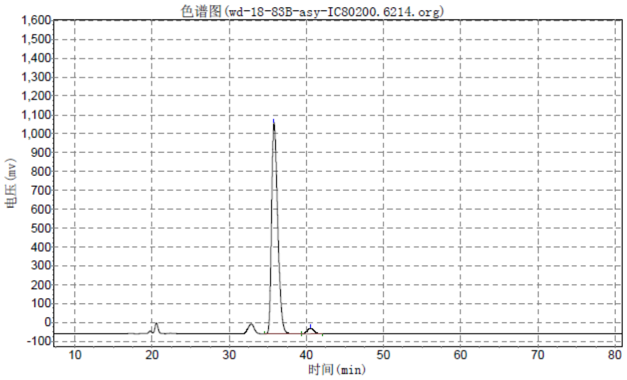

分析结果表

峰号	峰名	保留时间	峰高	峰面积	含量
1		35.810	1119109.500	61844436.000	97.1555
2		40.527	30458.652	1810696.000	2.8445
总计			1149568.152	63655132.000	100.0000

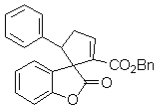

IC-H, *n*-Hexane:^{i}PrOH = 80:20, 0.6 ml/min, 214 nm

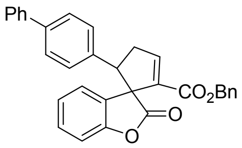

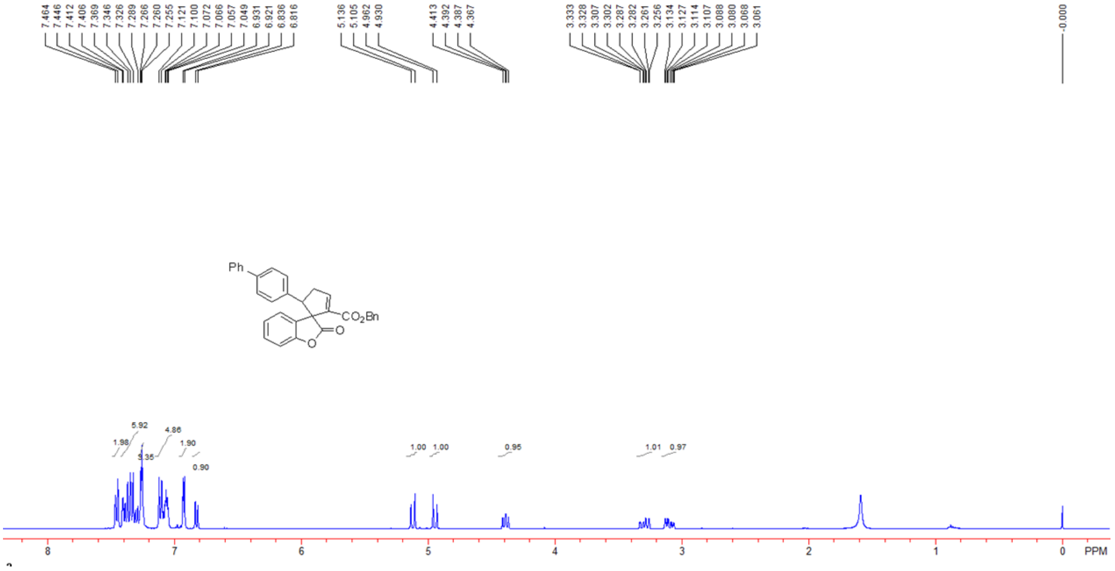

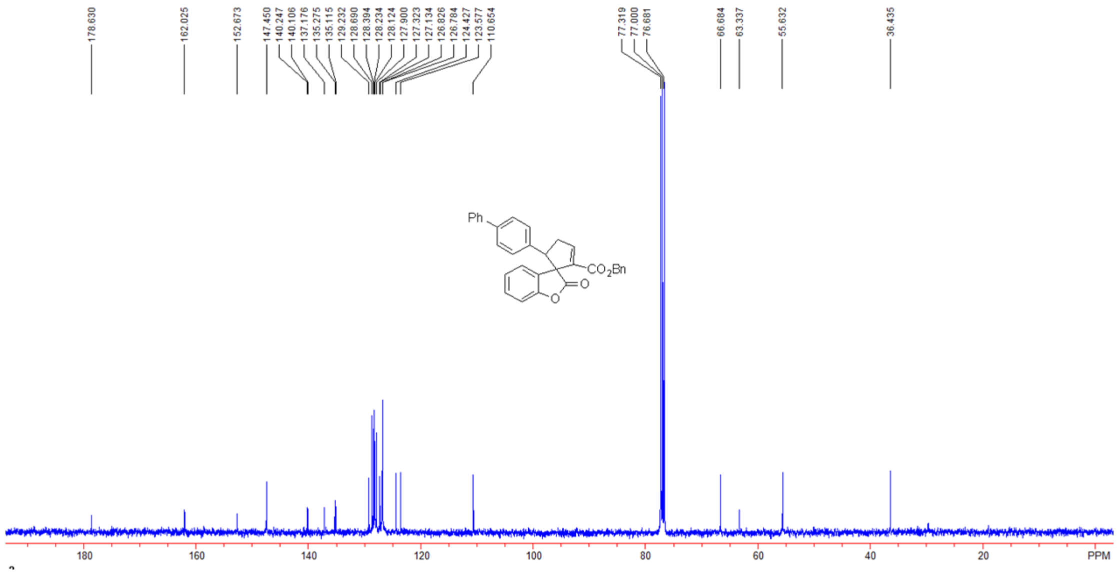

Benzyl 5'-([1,1'-biphenyl]-4-yl)-2-oxo-2H-spiro[benzofuran-3,1'-cyclopent[2]ene]-2'-carboxylate (3j)

A pale yellow solid, 76% yield, 35 mg, Mp: 98-100 ºC. ^1H NMR (400 MHz, $CDCl_3$, TMS) δ 7.46 (d, J = 7.2 Hz, 1H), 7.41-7.29 (m, 6H), 7.27-7.26 (m, 3H), 7.12-7.05 (m, 5H), 6.93 (d, J = 4.0 Hz, 2H), 6.83 (d, J = 8.0 Hz, 1H), 5.12 (d, J = 12.4 Hz, 1H), 4.95 (d, J = 12.4 Hz, 1H), 4.39 (dd, J_1 = 8.0 Hz, J_2 = 10.4 Hz, 1H), 3.29 (ddd, J_1 = 2.0 Hz, J_2 = 10.4 Hz, J_3 = 18.4 Hz, 1H), 3.10 (ddd, J_1 = 2.8 Hz, J_2 = 8.0 Hz, J_3 = 18.4 Hz, 1H); ^{13}C NMR (100 MHz, $CDCl_3$) δ 178.6, 162.0, 152.7, 147.5, 140.2, 140.1, 137.2, 135.3, 135.1, 129.2, 128.7, 128.4, 128.2, 128.1, 127.9, 127.3, 127.1, 126.83, 126.78, 124.4, 123.6, 110.7, 66.7, 63.3, 55.6, 36.4; IR (neat) ν 3007, 1800, 1712, 1462, 1275, 1260, 1071, 749, 695 cm^{-1}; HRMS Calcd. for $C_{32}H_{28}NO_4^{+1}$ $(M+NH_4)^+$: 490.2013, found: 490.2035. $[\alpha]^{20}_D$ = +54.9 (c 1.2, $CHCl_3$) for 95% ee; Enantiomeric excess was determined by HPLC with a Chiralcel PC-2 column, Hexane/^{i}PrOH = 60/40, 0.5 mL/min, 230 nm, t_{minor} = 35.240 min, t_{major} = 44.685 min.

实验时间：2013-09-04, 13:51:54
谱图文件:I:\regio-and enantio\液相\wd-19-63-race-PC-2-6040-0.5-230.org

实验者：
报告时间：2013-10-16, 21:47:50
积分方法:面积归一法

使用仪器类型:气相色谱 检测器:FID 进样器:分流

柱温:程序升温

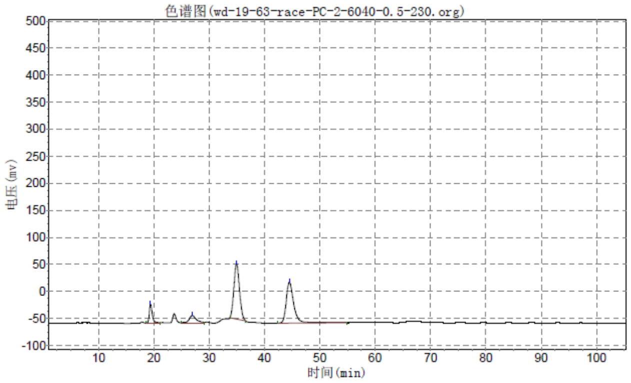

分析结果表

峰号	峰名	保留时间	峰高	峰面积	含量
1		19.422	35835.672	1464538.125	8.4069
2		26.943	14847.680	1570299.625	9.0140
3		34.948	102218.094	7160324.500	41.1023
4		44.487	76039.305	7225583.000	41.4769
总计			228940.750	17420745.250	100.0000

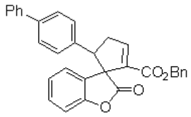

PC-2, *n*-Hexane:^{i}PrOH = 60:40, 0.5 ml/min, 230nm

实验时间：2013-09-04, 11:53:23
谱图文件:I:\regio-and enantio\液相\wd-19-63-asy-PC-2-6040-0.5-230.org

实验者：
报告时间：2013-10-16, 21:52:44
积分方法:面积归一法

使用仪器类型:气相色谱 检测器:FID 进样器:分流

柱温:程序升温

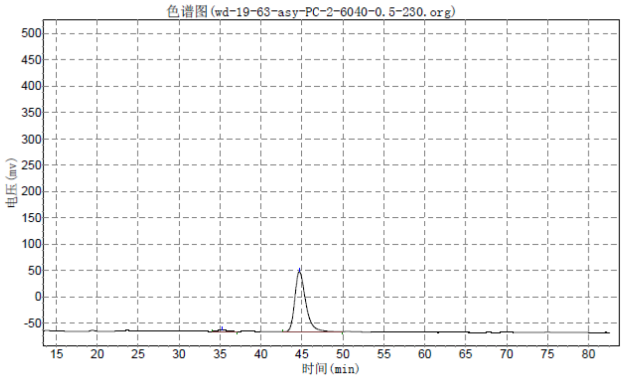

分析结果表

峰号	峰名	保留时间	峰高	峰面积	含量
1		35.240	3603.758	256653.281	2.3811
2		44.685	114280.500	10522072.000	97.6189
总计			117884.258	10778725.281	100.0000

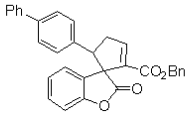

PC-2, *n*-Hexane:^{i}PrOH = 60:40, 0.5 ml/min, 230nm

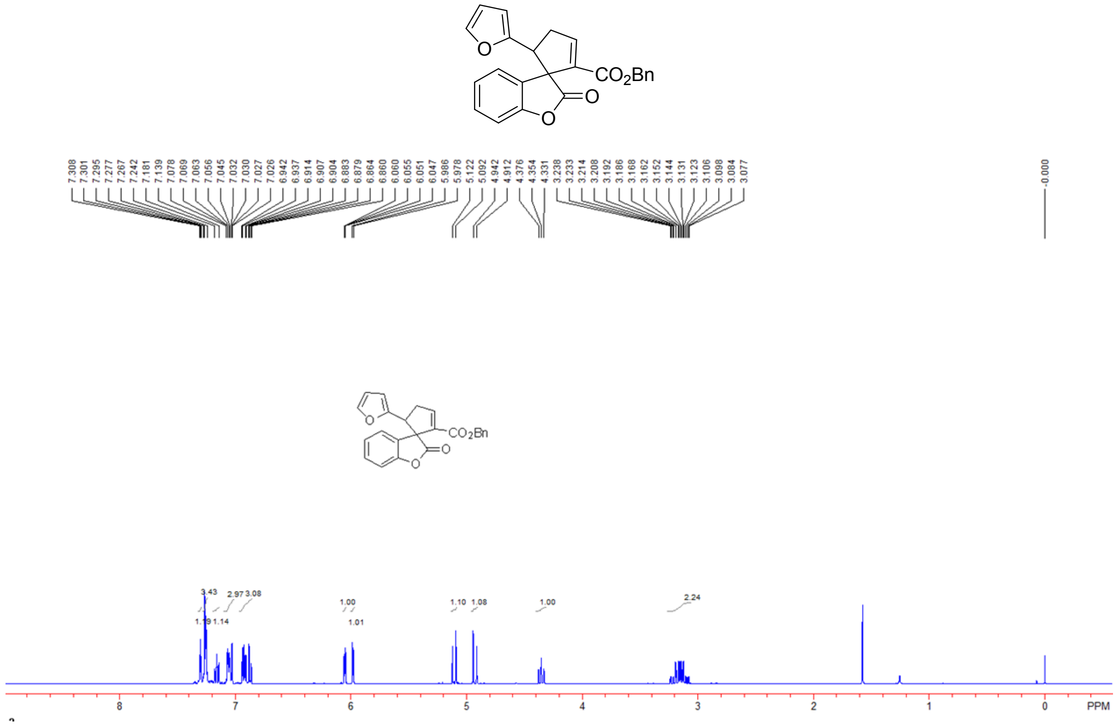

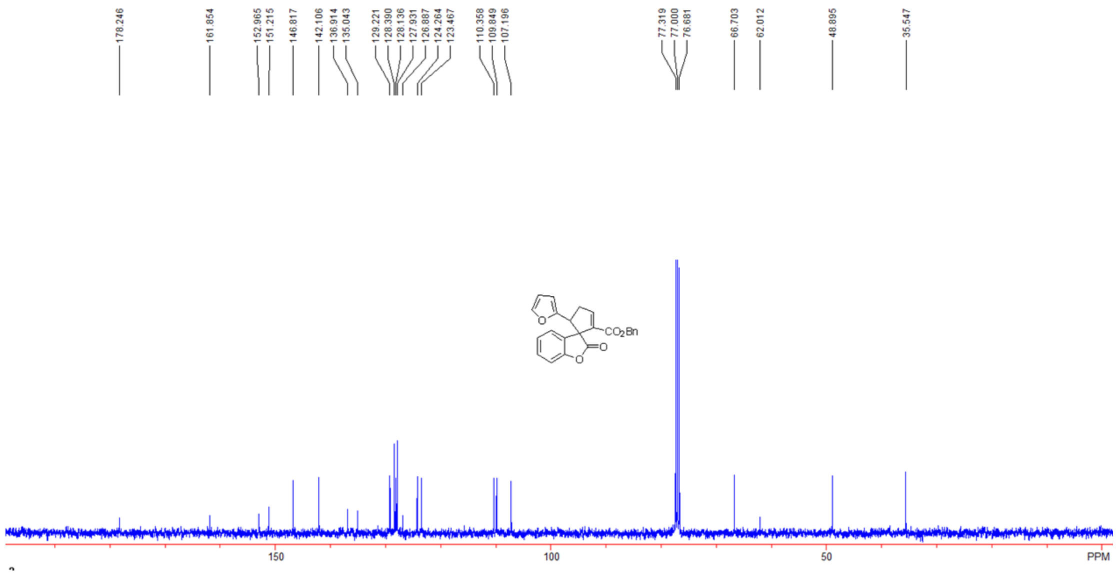

Benzyl 5'-(furan-2-yl)-2-oxo-2H-spiro[benzofuran-3,1'-cyclopent[2]ene]-2'-carboxylate (3k)

A yellow oil, 67% yield, 26 mg. ^{1}H NMR (400 MHz, $CDCl_3$, TMS) δ 7.30 (t, J = 2.8 Hz, 1H), 7.28-7.24 (m, 3H), 7.18-7.14 (m, 1H), 7.08-7.03 (m, 3H), 6.94-6.86 (m, 3H), 6.05 (dd, J_1 = 2.0 Hz, J_2 = 3.2 Hz, 1H), 5.98 (d, J = 3.2 Hz, 1H), 5.11 (d, J = 12.0 Hz, 1H), 4.93 (d, J = 12.0 Hz, 1H), 4.35 (t, J = 8.8 Hz, 1H), 3.24-3.08 (m, 2H); ^{13}C NMR (100 MHz, $CDCl_3$) δ 178.2, 161.9, 153.0, 151.2, 146.8, 142.1, 136.9, 135.0, 129.2, 128.4, 128.1, 127.9, 126.9, 124.3, 123.5, 110.4, 109.8, 107.2, 66.7, 62.0, 48.9, 35.5; IR (neat) ν 2914, 1800, 1709, 1617, 1461, 1338, 1229, 1131, 1070, 1016, 876 cm^{-1}; HRMS Calcd. for $C_{24}H_{22}NO_5^{+1}$ $(M+NH_4)^+$: 404.1492, found: 404.1489. $[\alpha]^{20}_D$ = +123.7 (c 0.3, $CHCl_3$) for 96% ee; Enantiomeric excess was determined by HPLC with a

Chiralcel PC-2 column, Hexane/^{i}PrOH = 90/10, 0.7 mL/min, 214 nm, t_{minor} = 40.662 min, t_{major} = 47.713 min.

N2000 数据工作站 1

实验时间：2013-08-21,14:18:11
谱图文件:I:\regio-and enantio\液相\wd-19-43-race-re-PC-2-9010-0.7-214.org

实验者:
报告时间：2013-10-14,20:22:05
积分方法:面积归一法

使用仪器类型:气相色谱 检测器:FID 进样器:分流

柱温:程序升温

色谱图(wd-19-43-race-re-PC-2-9010-0.7-214.org)

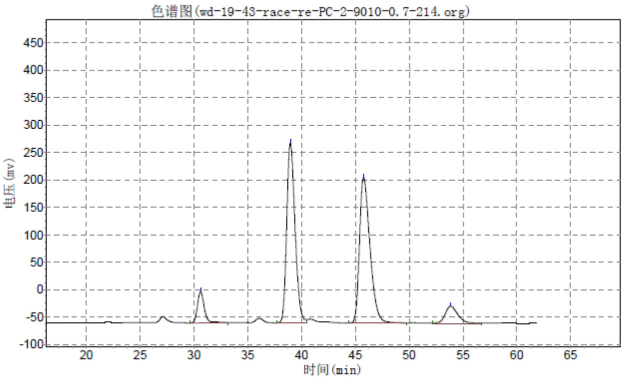

分析结果表

峰号	峰名	保留时间	峰高	峰面积	含量
1		30.613	57202.328	2500266.000	6.1452
2		38.960	328637.313	17636716.000	43.3481
3		45.770	266144.188	18006906.000	44.2579
4		53.852	31079.986	2542374.250	6.2487
总计			683063.814	40686262.250	100.0000

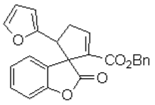

PC-2, *n*-Hexane:^{i}PrOH = 90:10, 0.7 ml/min, 214 nm

实验时间：2013-08-21, 15:30:10
谱图文件:I:\regio-and enantio\液相\wd-19-43-asy-re-PC-2-9010-0.7-214.org

实验者：
报告时间：2013-10-14, 20:27:20
积分方法:面积归一法

使用仪器类型:气相色谱 检测器:FID 进样器:分流

柱温:程序升温

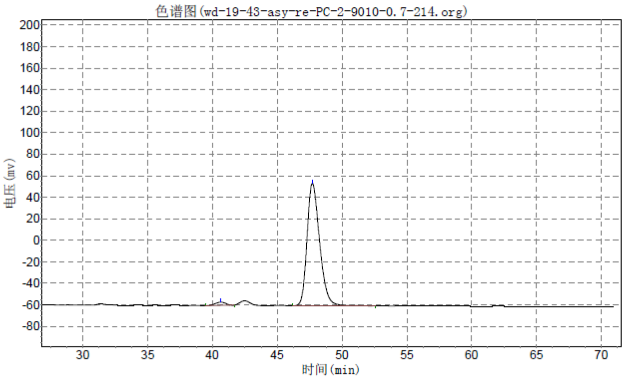

分析结果表

峰号	峰名	保留时间	峰高	峰面积	含量
1		40.662	3031.085	168679.797	2.1534
2		47.713	113973.914	7664514.000	97.8466
总计			117005.000	7833193.797	100.0000

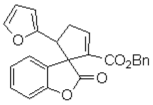

PC-2, *n*-Hexane:^{i}PrOH = 90:10, 0.7 ml/min, 214 nm

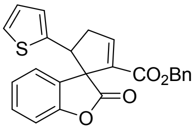

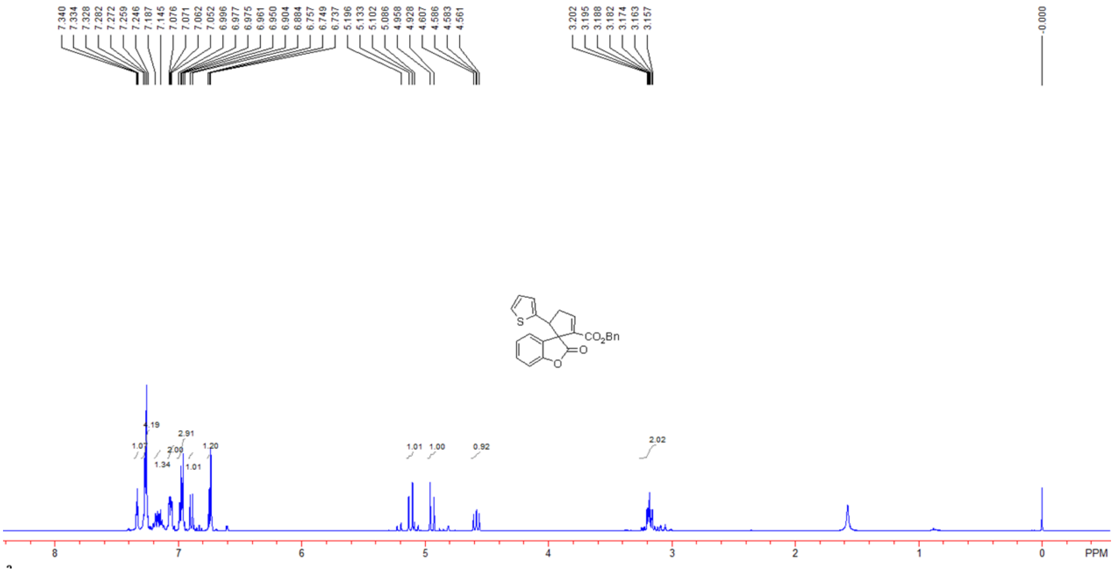

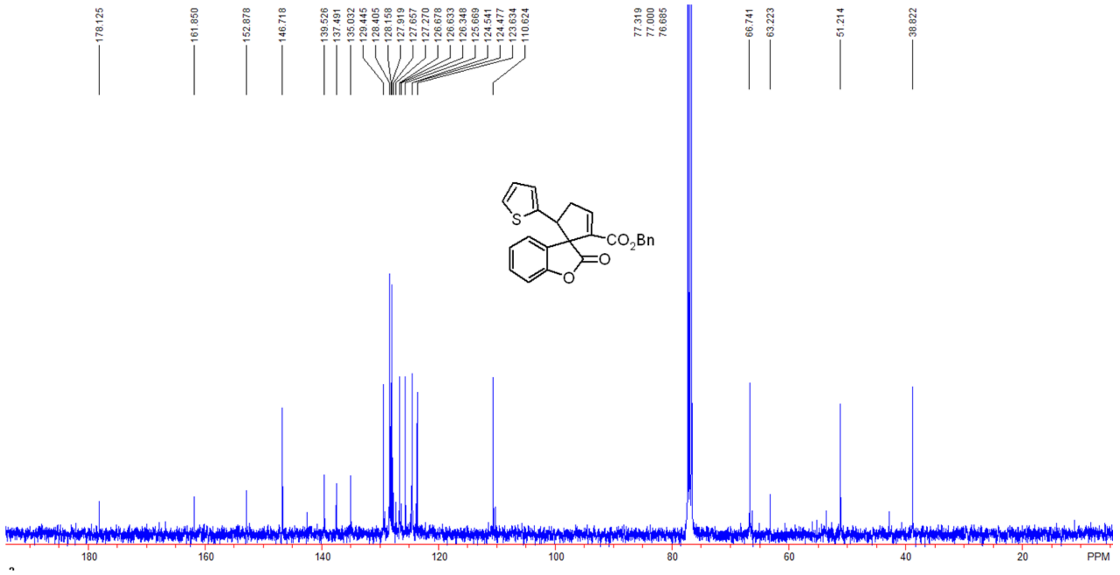

Benzyl 2-oxo-5'-(thiophen-2-yl)-2H-spiro[benzofuran-3,1'-cyclopent[2]ene]-2'-carboxylate (3l)

A yellow oil, 67% yield, 26 mg. ^{1}H NMR (400 MHz, $CDCl_3$, TMS) δ 7.33 (t, J = 2.4 Hz, 1H), 7.28-7.25 (m, 4H), 7.19-7.15 (m, 1H), 7.08-7.05 (m, 2H), 7.00-6.95 (m, 3H), 6.89 (d, J = 8.0 Hz, 1H), 6.76-6.74 (m, 1H), 5.12 (d, J = 12.4 Hz, 1H), 4.94 (d, J = 12.4 Hz, 1H), 4.58 (dd, J_1 = 8.4 Hz, J_2 = 10.0 Hz, 1H), 3.20-3.16 (m, 2H); ^{13}C NMR (100 MHz, $CDCl_3$) δ 178.1, 161.9, 152.9, 146.7, 139.5, 137.5, 135.0, 129.4, 128.4, 128.2, 127.9, 127.7, 127.3, 126.7, 126.6, 126.3, 125.7, 124.54, 124.48, 123.6, 110.6, 66.7, 63.2, 51.2, 38.8; IR (neat) ν 2926, 1797, 1712, 1617, 1461, 1335, 1233, 1069, 999, 875 cm^{-1}; HRMS Calcd. for $C_{24}H_{22}NO_4S^{+1}$ $(M+NH_4)^+$: 420.1264, found: 420.1260. $[\alpha]^{20}_{D}$ = +93.8 (c 1.2, $CHCl_3$) for 93% ee, 90:10 r.r.; Enantiomeric excess was determined by HPLC with a Chiralcel PC-2 column, Hexane/^{i}PrOH = 90/10, 0.7 mL/min, 214 nm, t_{minor} = 44.468 min, t_{major} = 65.975 min.

N2000 数据工作站 1

实验时间：2013-08-17, 12:23:27
谱图文件:I:\regio-and enantio\液相\wd-18-94-race-PC-2-9010-0.7-214.org

实验者:
报告时间：2013-10-14, 19:56:41
积分方法:面积归一法

使用仪器类型:气相色谱 检测器:FID 进样器:分流

柱温:程序升温

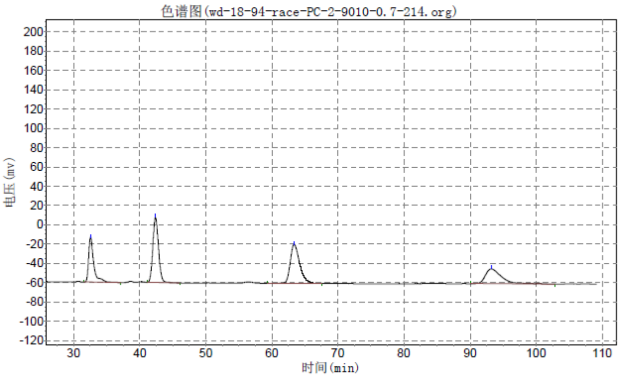

分析结果表

峰号	峰名	保留时间	峰高	峰面积	含量
1		32.598	46729.355	2588256.500	19.1204
2		42.398	67486.227	4099569.500	30.2850
3		63.398	40533.648	4094117.250	30.2447
4		93.265	15505.283	2754684.000	20.3499
总计			170254.514	13536627.250	100.0000

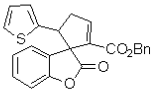

PC-2, *n*-Hexane:^{i}PrOH = 90:10, 0.7 ml/min, 214 nm

N2000 数据工作站 1

实验时间：2013-08-17, 16:01:22
谱图文件:I:\regio-and enantio\液相\wd-18-94-asy-PC-2-9010-0.7-214.org

实验者:
报告时间：2013-10-14, 20:10:29
积分方法:面积归一法

使用仪器类型:气相色谱 检测器:FID 进样器:分流

柱温:程序升温

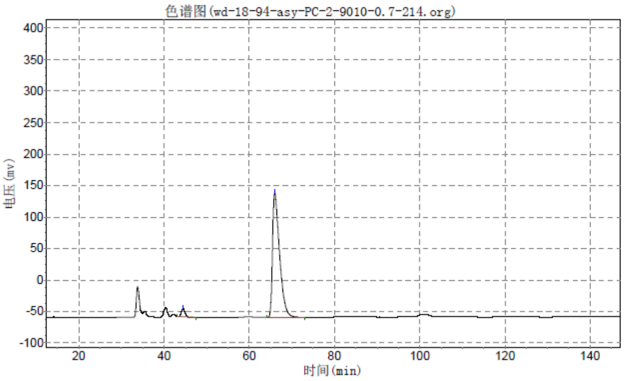

分析结果表

峰号	峰名	保留时间	峰高	峰面积	含量
1		44.468	13111.495	743387.688	3.1879
2		65.975	197719.797	22575420.000	96.8121
总计			210831.292	23318807.688	100.0000

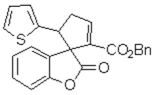

PC-2, *n*-Hexane:^{i}PrOH = 90:10, 0.7 ml/min, 214 nm

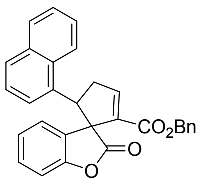

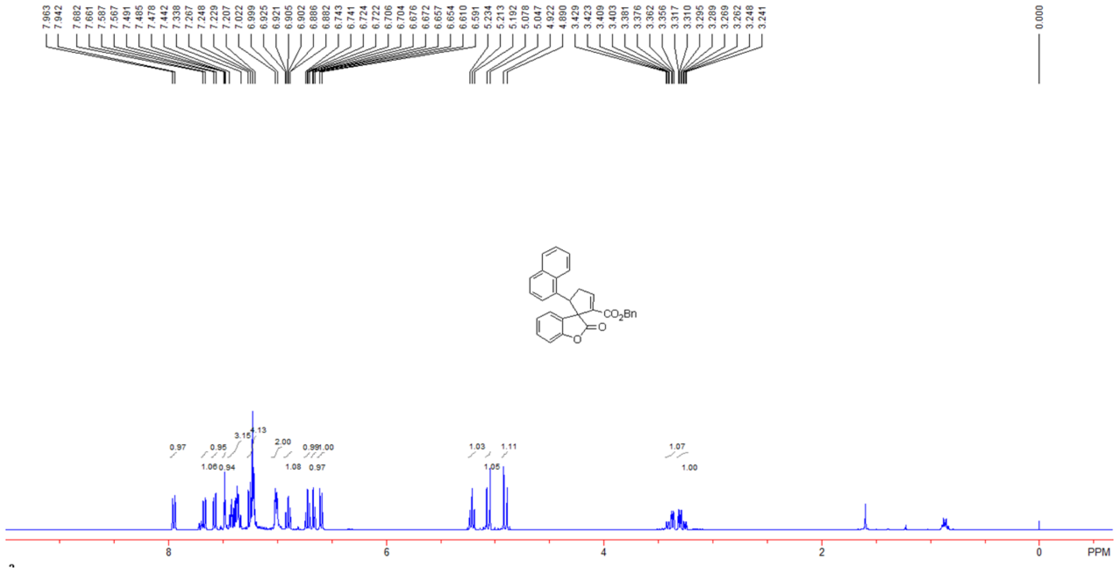

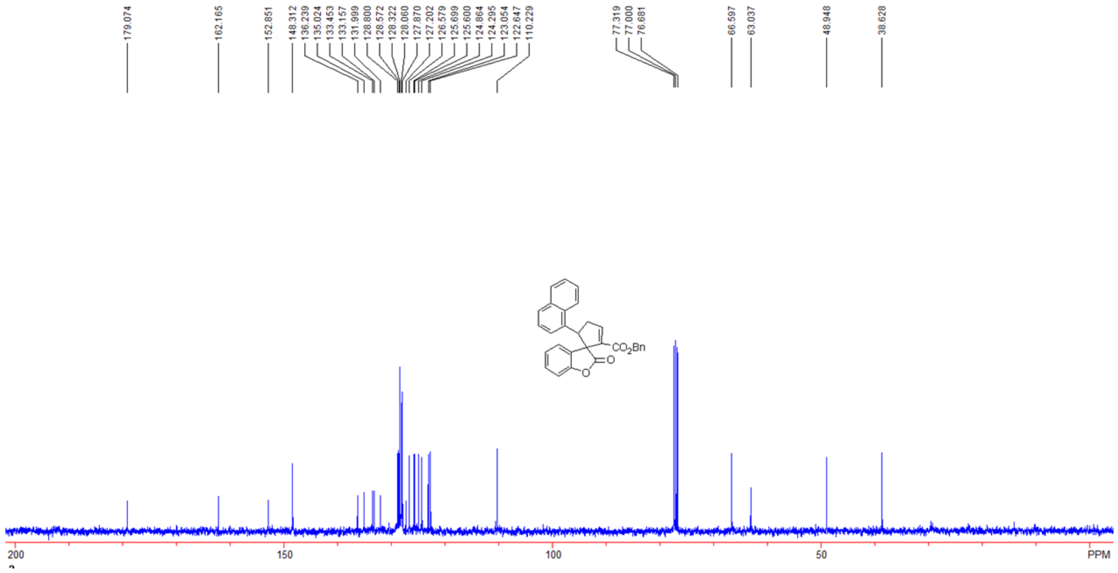

Benzyl 5'-(naphthalen-1-yl)-2-oxo-2H-spiro[benzofuran-3,1'-cyclopent[2]ene]-2'-carboxylate (3m)

A colorless solid, 99% yield, 45 mg, Mp: 123-124 ºC. ^{1}H NMR (400 MHz, $CDCl_3$, TMS) δ 7.95 (d, J = 8.4 Hz, 1H), 7.68-7.66 (m, 1H), 7.58 (d, J = 8.0 Hz, 1H), 7.49 (t, J = 2.4 Hz, 1H), 7.44-7.34 (m, 3H), 7.27-7.21 (m, 4H), 7.02-7.00 (m, 2H), 6.90 (dt, J_1 = 1.6 Hz, J_2 = 8.0 Hz, 1H), 6.72 (dt, J_1 = 0.8 Hz, J_2 = 7.6 Hz, 1H), 6.66 (dd, J_1 = 1.6 Hz, J_2 = 7.6 Hz, 1H), 6.60 (d, J = 7.6 Hz, 1H), 5.21 (t, J = 8.4 Hz, 1H), 5.06 (d, J = 12.4 Hz, 1H), 4.91 (d, J = 12.4 Hz, 1H), 3.39 (ddd, J_1 = 2.4 Hz, J_2 = 8.0 Hz, J_3 = 19.0 Hz, 1H), 3.28 (ddd, J_1 = 2.8 Hz, J_2 = 8.4 Hz, J_3 = 19.0 Hz, 1H); ^{13}C

NMR (100 MHz, $CDCl_3$) δ 179.1, 162.2, 152.9, 148.3, 136.2, 135.0, 133.5, 133.2, 132.0, 128.8, 128.6, 128.3, 128.1, 127.9, 127.2, 126.6, 125.7, 125.6, 124.9, 124.3, 123.1, 122.6, 110.2, 66.6, 63.0, 48.9, 38.6; IR (neat) ν 2926, 1795, 1709, 1617, 1461, 1259, 1108, 1071, 750, 695 cm^{-1}; HRMS Calcd. for $C_{30}H_{26}NO_4^{+1}$ $(M+NH_4)^+$: 464.1856, found: 464.1863. $[\alpha]^{20}_D$ = +356.1 (c 0.4, $CHCl_3$) for >99% ee; Enantiomeric excess was determined by HPLC with a Chiralcel AD-H column, Hexane/^{i}PrOH = 90/10, 0.6 mL/min, 214 nm, t_{minor} = 32.215 min, t_{major} = 40.858 min.

N2000 数据工作站 1

实验时间: 2013-09-02, 10:48:25
谱图文件:I:\regio-and enantio\液相\wd-19-20-rac-AD-9010-0.6-214.org
实验者:
报告时间: 2013-10-14, 20:44:56
积分方法:面积归一法

使用仪器类型:气相色谱 检测器:FID 进样器:分流

柱温:程序升温

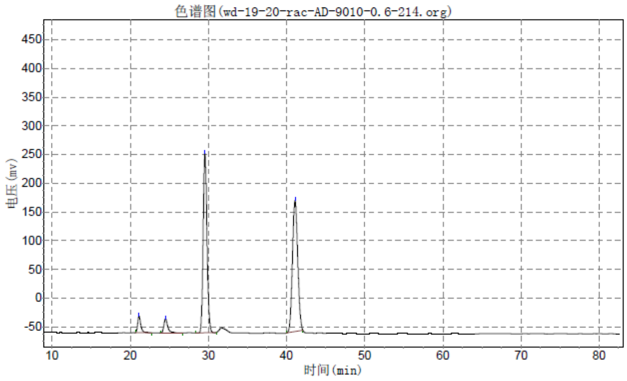

分析结果表

峰号	峰名	保留时间	峰高	峰面积	含量
1		21.123	29052.352	827859.813	3.6058
2		24.513	24717.545	836967.375	3.6454
3		29.557	311891.094	10617242.000	46.2436
4		41.078	228114.016	10677297.000	46.5052
总计			593775.006	22959366.188	100.0000

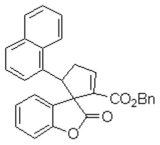

AD-H, *n*-Hexane:^{i}PrOH = 90:10, 0.6 ml/min, 214 nm

实验时间：2013-09-02, 12:45:13
谱图文件:I:\6.regio-and enantio\液相\wd-19-20-asy-re-AD-9010-0.6-214.org

实验者：
报告时间：2013-12-02, 20:42:29
积分方法:面积归一法

使用仪器类型:气相色谱 检测器:FID 进样器:分流

柱温:程序升温

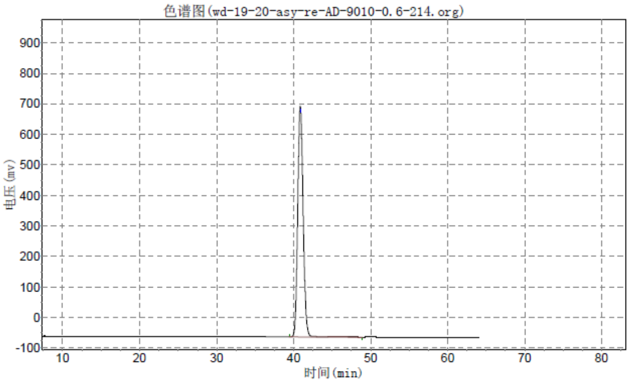

分析结果表

峰号	峰名	保留时间	峰高	峰面积	含量
1		40.932	750652.625	39056824.000	100.0000
总计			750652.625	39056824.000	100.0000

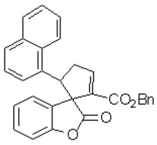

AD-H, Hexane:^{i}PrOH = 90:10, 0.6 ml/min, 214 nm.

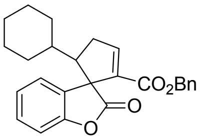

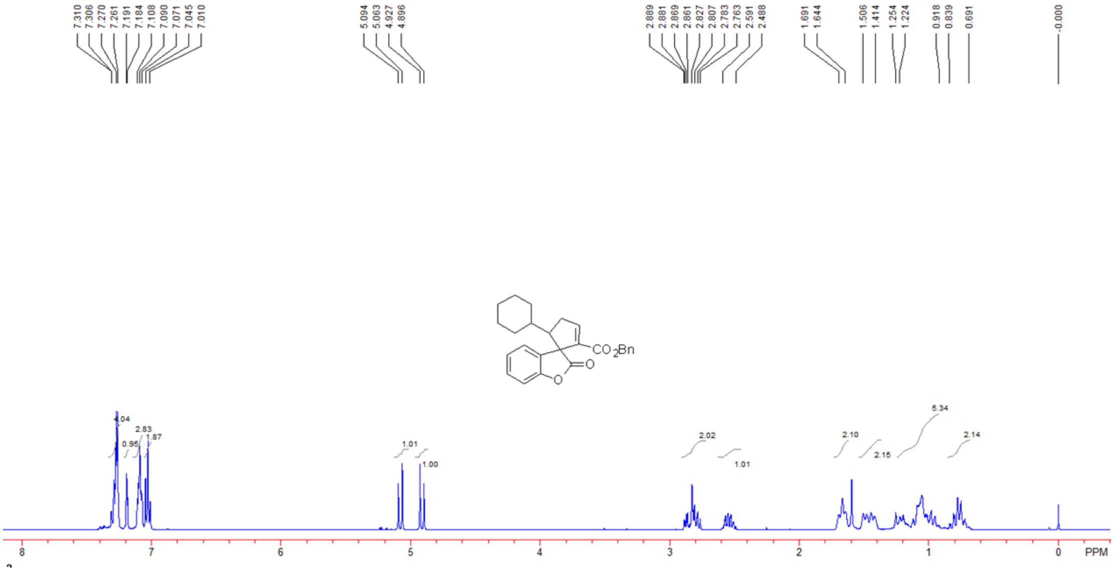

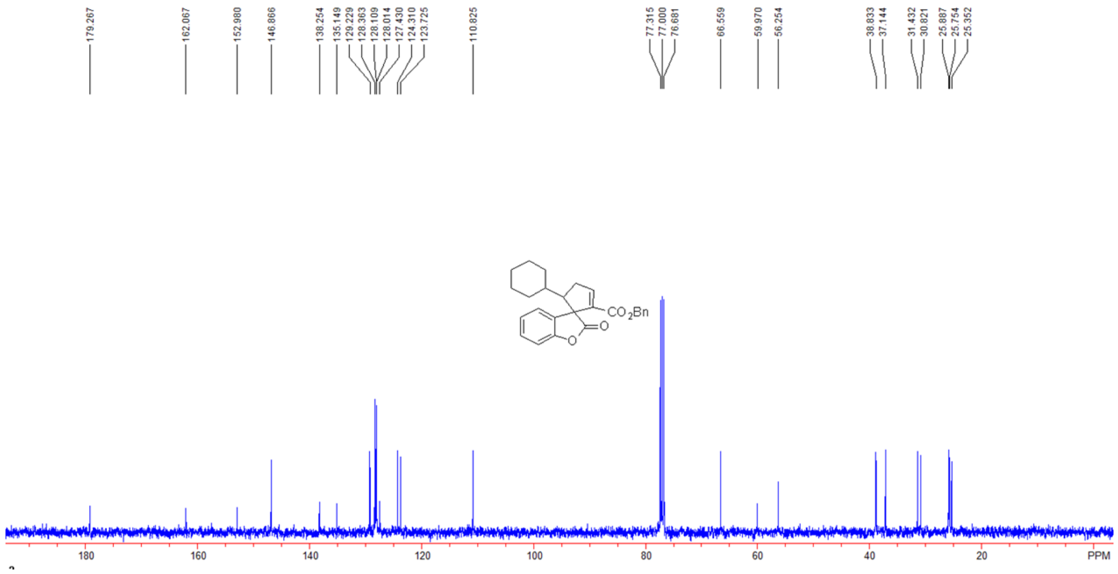

Benzyl 5'-cyclohexyl-2-oxo-2H-spiro[benzofuran-3,1'-cyclopent[2]ene]-2'-carboxylate (3n)

A pale yellow oil, 68% yield, 27 mg. ^{1}H NMR (400 MHz, $CDCl_3$, TMS) δ 7.31-7.26 (m, 4H), 7.19-7.18 (m, 1H), 7.11-7.07 (m, 3H), 7.05-7.01 (m, 2H), 5.08 (d, J = 12.4 Hz, 1H), 4.91 (d, J = 12.4 Hz, 1H), 2.89-2.76 (m, 2H), 2.59-2.49 (m, 1H), 1.69-1.64 (m, 2H), 1.51-1.41 (m, 2H), 1.25-0.92 (m, 5H), 0.84-0.69 (m, 2H); ^{13}C NMR (100 MHz, $CDCl_3$) δ 179.3, 162.1, 153.0, 146.9, 138.3, 135.1, 129.2, 128.4, 128.1, 128.0, 127.4, 124.3, 123.7, 110.8, 66.6, 60.0, 56.3, 38.8, 37.1, 31.4, 30.8, 25.9, 25.8, 25.4; IR (neat) ν 2925, 1800, 1712, 1632, 1461, 1328, 1233, 1068, 996, 752 cm^{-1}; HRMS Calcd. for $C_{26}H_{30}NO_4^{+1}$ $(M+NH_4)^+$: 420.2169, found: 420.2168. $[\alpha]^{20}_{D}$ = +115.6 (c 0.6, $CHCl_3$) for 95% ee; Enantiomeric excess was determined by HPLC with a Chiralcel IC-H column, Hexane/^{i}PrOH = 95/5, 0.7 mL/min, 214 nm, t_{minor} = 139.188 min, t_{major} = 70.937 min.

实验时间：2013-09-18, 14:30:56
谱图文件:I:\regio-and enantio\液相\wd-19-76-race-re-IC-955-0.7-214.org
实验者：
报告时间：2013-10-16, 22:01:24
积分方法:面积归一法

使用仪器类型:气相色谱 检测器:FID 进样器:分流

柱温:程序升温

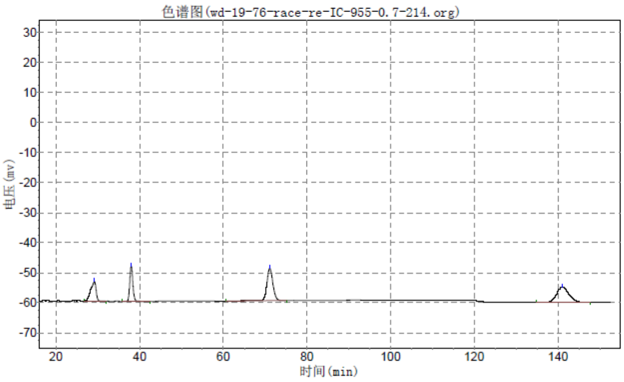

分析结果表

峰号	峰名	保留时间	峰高	峰面积	含量
1		29.160	6620.237	599422.688	18.1927
2		37.980	11780.327	602567.625	18.2882
3		71.103	10727.334	1047130.250	31.7808
4		141.045	5033.070	1045729.500	31.7383
总计			34160.968	3294850.063	100.0000

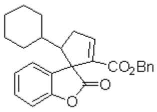

IC-H, *n*-Hexane:^{i}PrOH = 95:5, 0.7 ml/min, 214nm

实验时间：2013-09-18, 11:57:06
谱图文件:I:\regio-and enantio\液相\wd-19-76-asy-IC-955-0.7-214.org
实验者：
报告时间：2013-10-16, 22:03:05
积分方法:面积归一法

使用仪器类型:气相色谱 检测器:FID 进样器:分流

柱温:程序升温

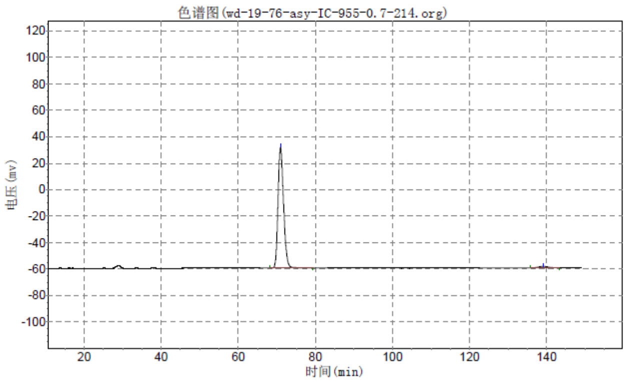

分析结果表

峰号	峰名	保留时间	峰高	峰面积	含量
1		70.937	91221.828	9047222.000	97.5337
2		139.188	1232.433	228778.109	2.4663
总计			92454.261	9276000.109	100.0000

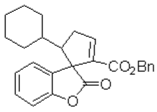

IC-H, *n*-Hexane:^{i}PrOH = 95:5, 0.7 ml/min, 214nm

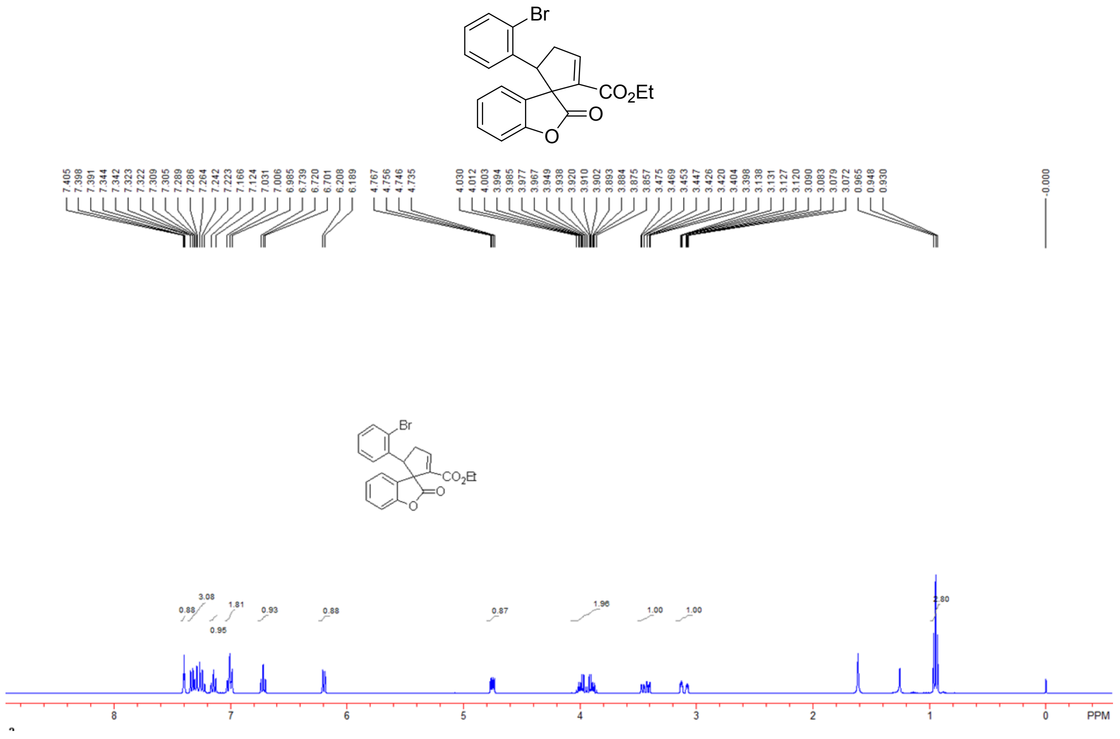

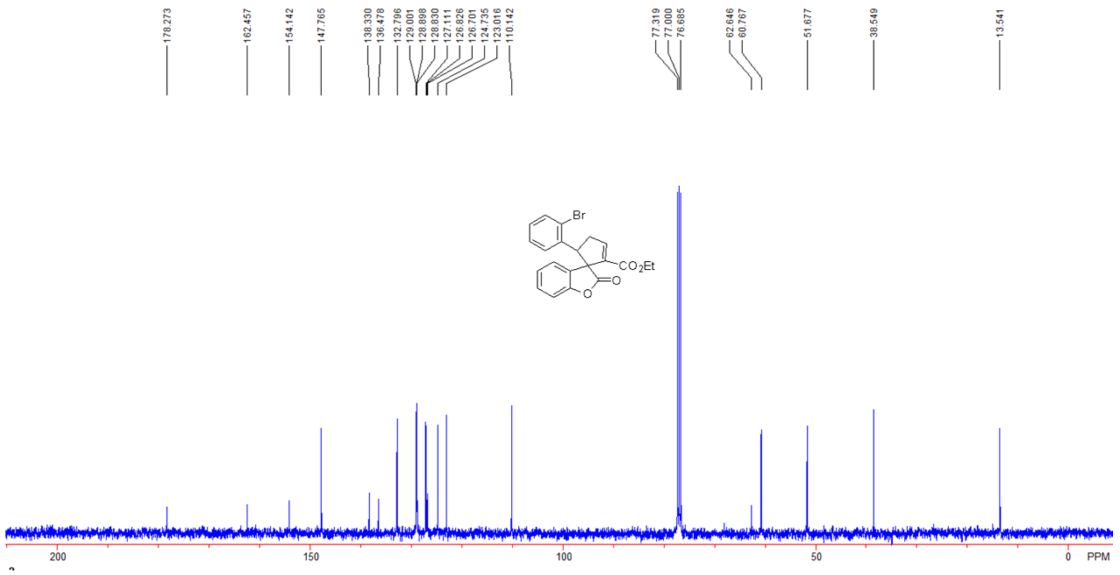

Ethyl 5'-(2-bromophenyl)-2-oxo-2H-spiro[benzofuran-3,1'-cyclopent[2]ene]-2'-carboxylate (3o)

A pale yellow solid, 94% yield, 39 mg, Mp: 179-180 ºC. ^{1}H NMR (400 MHz, $CDCl_3$, TMS) δ 7.40 (t, J = 2.8 Hz, 1H), 7.34-7.22 (m, 3H), 7.17-7.12 (m, 1H), 7.03-6.99 (m, 2H), 6.72 (t, J = 7.6 Hz, 1H), 6.20 (d, J = 7.6 Hz, 1H), 4.75 (dd, J_1 = 4.4 Hz, J_2 = 8.4 Hz, 1H), 4.03-3.86 (m, 2H), 3.44 (ddd, J_1 = 2.4 Hz, J_2 = 8.4 Hz, J_3 = 19.2 Hz, 1H), 3.11 (ddd, J_1 = 2.8 Hz, J_2 = 4.4 Hz, J_3 = 19.2 Hz, 1H), 0.93 (t, J = 7.2 Hz, 1H); ^{13}C NMR (100 MHz, $CDCl_3$) δ 178.3, 162.5, 154.1, 147.8, 138.3, 136.5, 132.8, 129.0, 128.9, 128.8, 127.1, 126.8, 126.7, 124.7, 123.0, 110.1, 62.6, 60.8, 51.7, 38.5, 13.5; IR (neat) ν 2921, 1801, 1714, 1462, 1275, 1260, 1070, 750 cm^{-1}; HRMS Calcd.

for $C_{21}H_{21}BrNO_4^{+1}$ $(M+NH_4)^+$: 430.0648, found: 430.0641. $[\alpha]^{20}_D$ = +116.0 (c 1.0, $CHCl_3$) for 99% ee; Enantiomeric excess was determined by HPLC with a Chiralcel AD-H column, Hexane/^{i}PrOH = 80/20, 0.5 mL/min, 214 nm, t_{minor} = 28.642 min, t_{major} = 25.942 min.

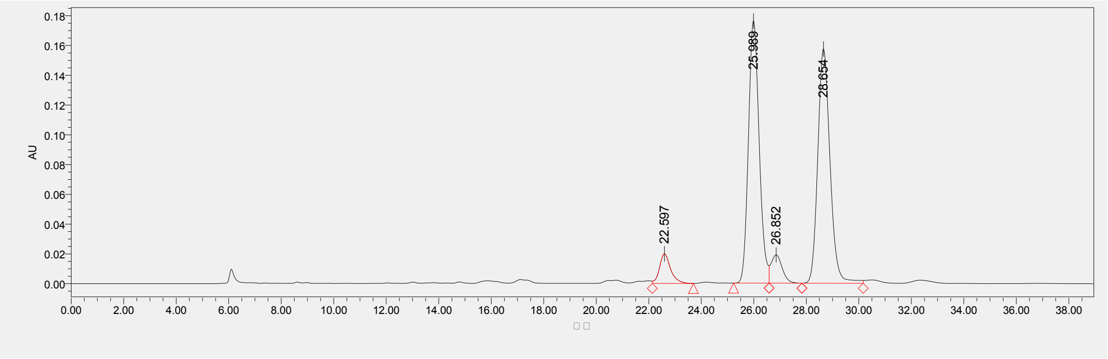

NO	R. Time	Peak Area	Precent	Peak Height
1	22.597	583755	5.18	19892
2	25.989	5021751	44.54	176248
3	26.852	559529	4.96	19009
4	28.654	5110892	45.33	157384

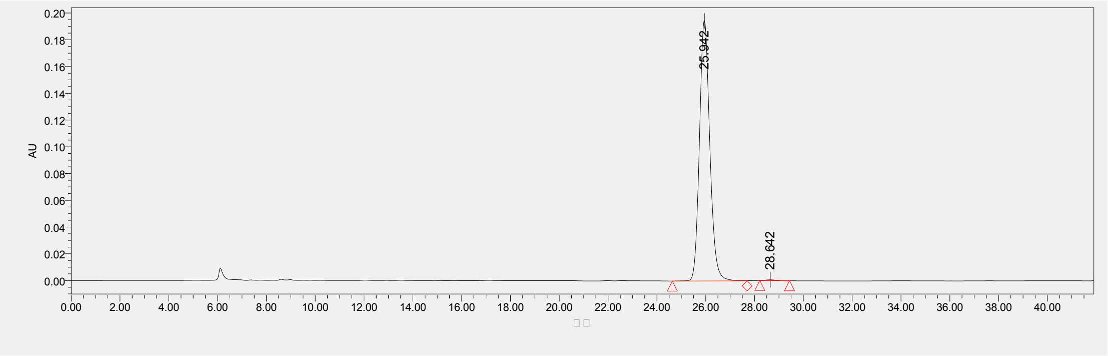

NO	R. Time	Peak Area	Precent	Peak Height
1	25.942	5741557	99.73	194648
2	28.642	6080678	0.27	532

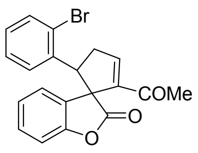

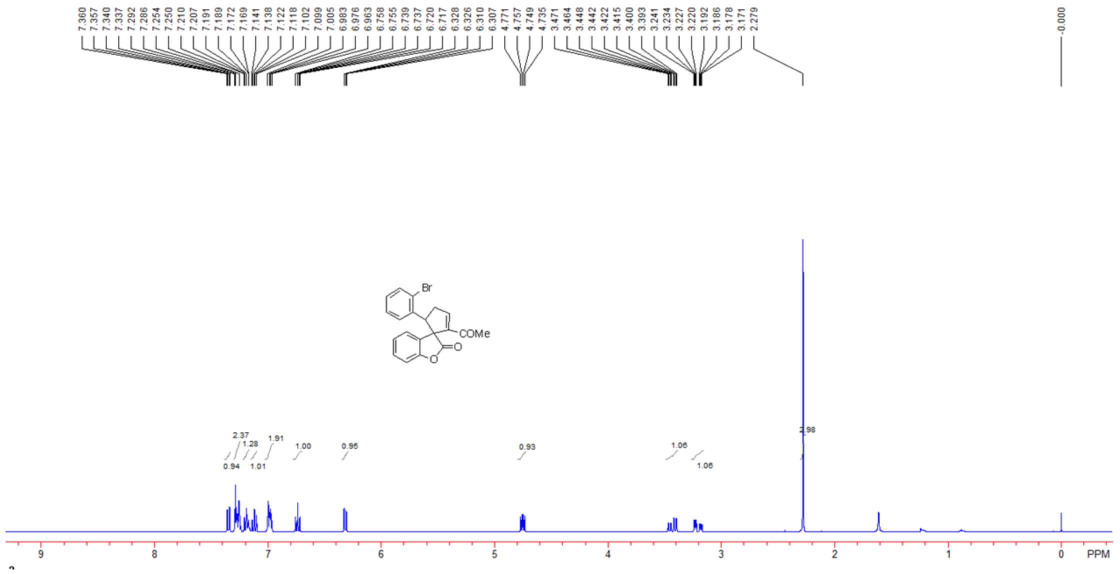

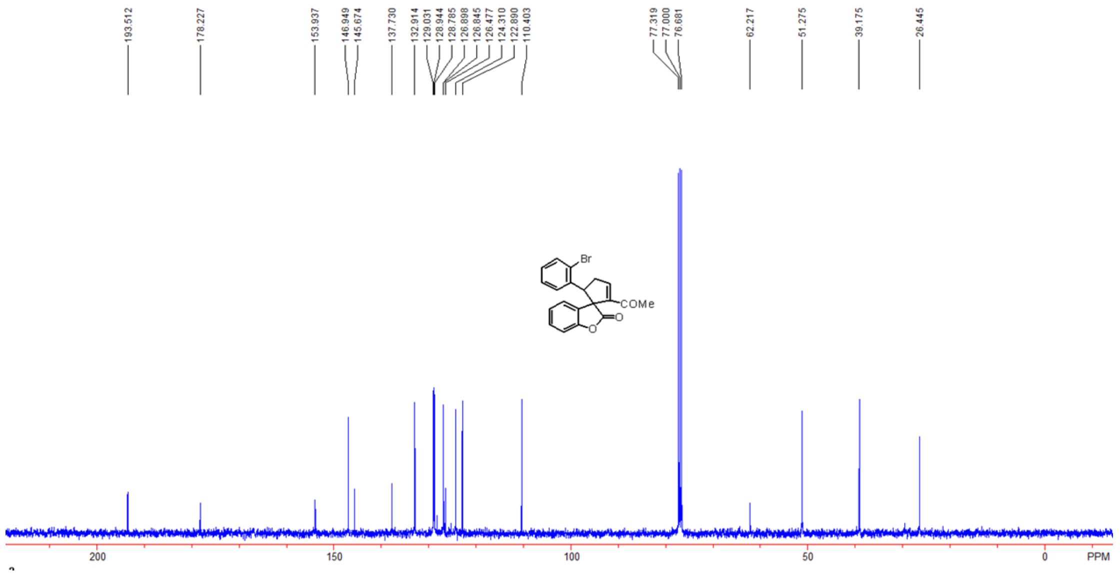

2'-Acetyl-5'-(2-bromophenyl)-2H-spiro[benzofuran-3,1'-cyclopent[2]en]-2-one (3p)

A white solid, 83% yield, 32 mg, Mp: 184-185 ºC. ^{1}H NMR (400 MHz, $CDCl_3$, TMS) δ 7.35 (dd, J_1 = 1.2 Hz, J_2 = 8.0 Hz, 1H), 7.29-7.25 (m, 2H), 7.19 (dt, J_1 = 1.2 Hz, J_2 = 7.6 Hz, 1H), 7.12 (dt, J_1 =1.2 Hz, J_2 = 7.6 Hz, 1H), 7.01-6.96 (m, 2H), 6.74 (dt, J_1 =1.2 Hz, J_2 = 7.6 Hz, 1H), 6.32 (dd, J_1 =1.2 Hz, J_2 = 7.2 Hz, 1H), 4.75 (dd, J_1 = 5.6 Hz, J_2 = 8.8 Hz, 1H), 3.43 (ddd, J_1 = 2.8 Hz, J_2 = 8.8 Hz, J_3 = 19.4 Hz, 1H), 3.21 (ddd, J_1 = 2.8 Hz, J_2 = 5.6 Hz, J_3 = 19.4 Hz, 1H), 2.28 (s, 3H); ^{13}C NMR (100 MHz, $CDCl_3$) δ 193.5, 178.2, 153.9, 146.9, 145.7, 137.7, 132.9, 129.0, 128.9, 128.8, 126.9, 126.8, 126.5, 124.3, 122.9, 110.4, 62.2, 51.3, 39.2, 26.4; IR (neat) ν 2920, 1796, 1667, 1617, 1462, 1275, 1129, 1068, 874, 676 cm^{-1}; HRMS Calcd. for $C_{20}H_{19}BrNO_3^{+1}$ (M+NH$_4$)$^+$: 400.0543, found: 400.0533. $[\alpha]^{20}_D$ = +146.4 (c 0.5, $CHCl_3$) for 96% ee; Enantiomeric excess was determined by HPLC with a Chiralcel AD-H column, Hexane/^{i}PrOH = 80/20, 0.5 mL/min, 214 nm, t_{minor} = 21.832 min, t_{major} = 17.582 min.

实验时间：2013-08-28, 15:47:59
谱图文件:I:\regio-and enantio\液相\wd-19-53-rac-re-AD-8020-0.5-214.org

实验者：
报告时间：2013-10-16, 22:09:45
积分方法:面积归一法

使用仪器类型:气相色谱 检测器:FID 进样器:分流

柱温:程序升温

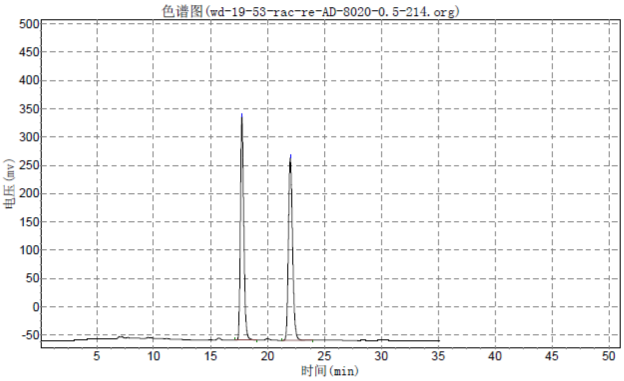

分析结果表

峰号	峰名	保留时间	峰高	峰面积	含量
1		17.757	394504.844	8118074.500	49.8750
2		22.015	321902.563	8158781.000	50.1250
总计			716407.406	16276855.500	100.0000

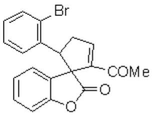

AD-H, *n*-Hexane:^{i}PrOH = 80:20, 0.5 ml/min, 214nm

N2000 数据工作站 1

实验时间：2013-08-28, 16:26:51
谱图文件:I:\regio-and enantio\液相\wd-19-53-asy-re-AD-8020-0.5-214.org

实验者：
报告时间：2013-10-16, 22:14:06
积分方法:面积归一法

使用仪器类型:气相色谱 检测器:FID 进样器:分流

柱温:程序升温

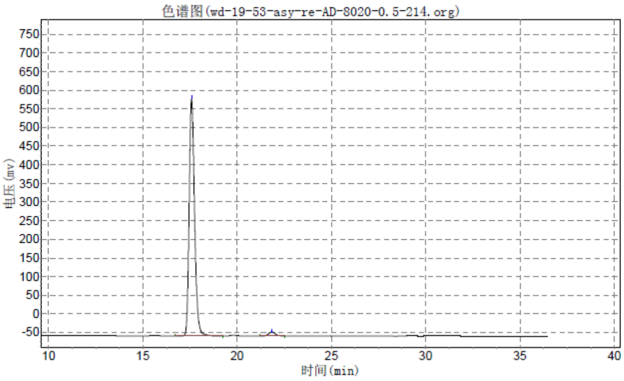

分析结果表

峰号	峰名	保留时间	峰高	峰面积	含量
1		17.582	635650.063	12633042.000	98.0432
2		21.830	10567.569	252136.063	1.9568
总计			646217.632	12885178.063	100.0000

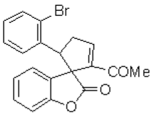

AD-H, *n*-Hexane:^{i}PrOH = 80:20, 0.5 ml/min, 214nm

6. Characterization and spectra charts containing HPLC traces for 5a-5p

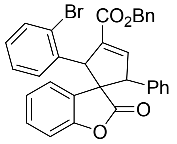

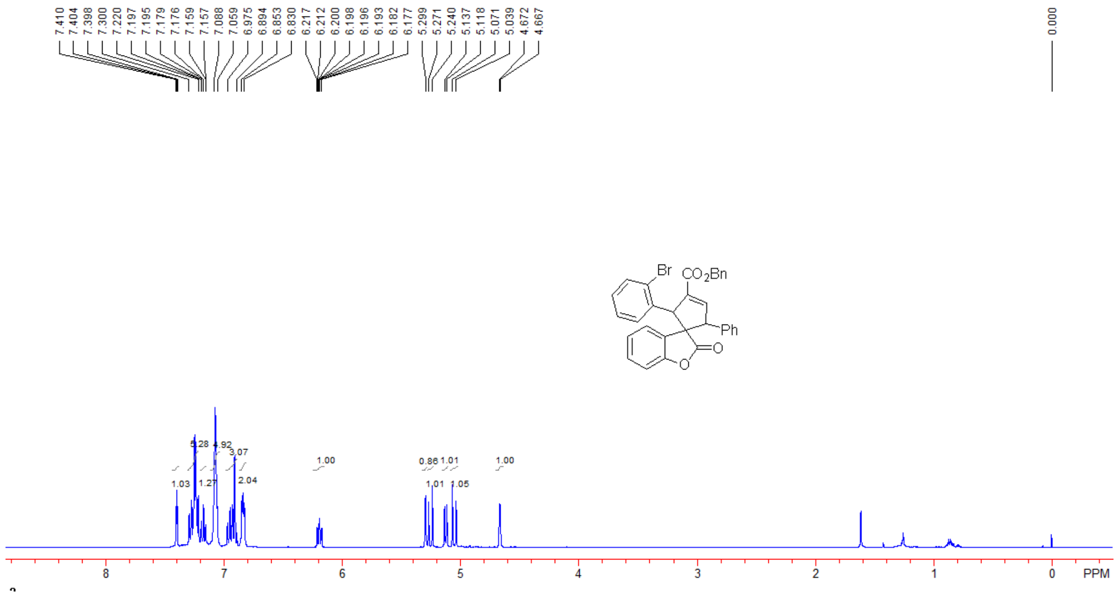

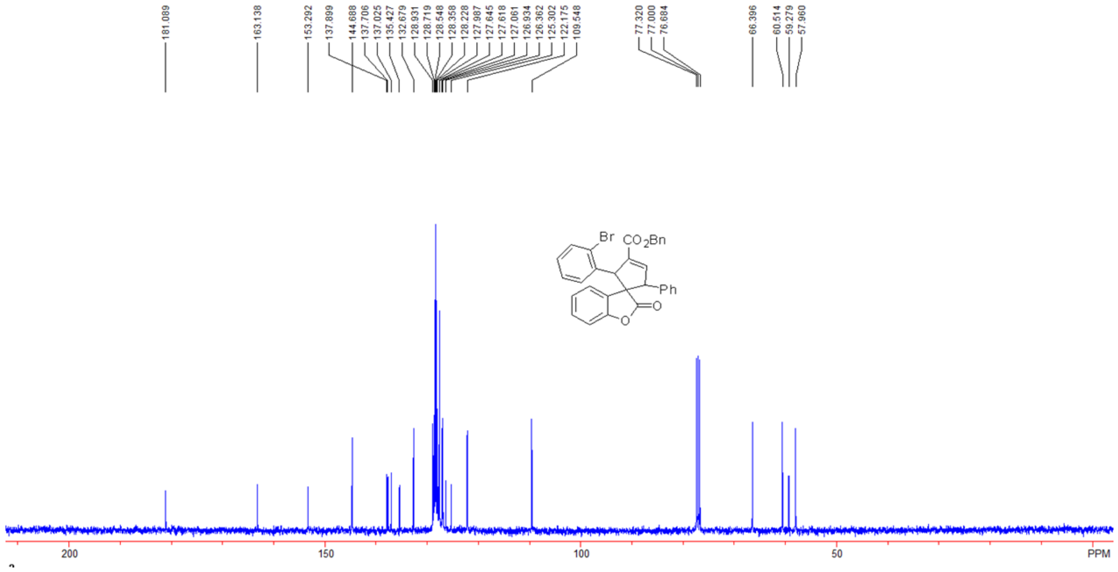

Benzyl 2'-(2-bromophenyl)-2-oxo-5'-phenyl-2H-spiro[benzofuran-3,1'-cyclopent[3]ene]-3'-carboxylate (5a)

A pale yellow solid, 96% yield, 52 mg, Mp: 67-68 ºC. ^{1}H NMR (400 MHz, $CDCl_3$, TMS) δ 7.40 (t, J = 2.4 Hz, 1H), 7.30-7.22 (m, 5H), 7.18 (dt, J_1 = 0.8 Hz, J_2 = 7.6 Hz, 1H), 7.09-7.06 (m, 5H), 6.98-6.89 (m, 3H), 6.85-6.83 (m, 2H), 6.22-6.18 (m, 1H), 5.30 (s, 1H), 5.26 (d, J = 12.4 Hz, 1H), 5.13 (d, J = 7.6 Hz, 1H), 5.06 (d, J = 12.4 Hz, 1H), 4.67 (d, J = 2.0 Hz, 1H); ^{13}C NMR (100 MHz, $CDCl_3$) δ 181.1, 163.1, 153.3, 144.7, 137.9, 137.7, 137.0, 135.4, 132.7, 128.9, 128.7, 128.5, 128.4, 128.2, 128.0, 127.65, 127.62, 127.1, 126.9, 126.4, 125.3, 122.2, 109.5, 66.4, 60.5, 59.3,

58.0; IR (neat) ν 3062, 1794, 1716, 1617, 1462, 1228, 1075, 966, 750, 696 cm^{-1}; HRMS Calcd. for $C_{32}H_{27}BrNO_4^{+1}$ $(M+NH_4)^+$: 568.1118, found: 568.1109. $[\alpha]^{20}_D$ = -83.1 (c 4.5, $CHCl_3$) for 95% ee; Enantiomeric excess was determined by HPLC with a Chiralcel IC-H column, Hexane/^{i}PrOH = 90/10, 0.5 mL/min, 214 nm, t_{minor} = 48.333 min, t_{major} = 31.928 min.

N2000 数据工作站 1

实验时间：2013-05-29, 10:44:31
谱图文件:I:\regio-and enantio\液相\wd-18-34-rac-IC-90100.5214.org

实验者：
报告时间：2013-10-21, 22:31:19
积分方法:面积归一法

使用仪器类型:气相色谱 检测器:FID 进样器:分流

柱温:程序升温

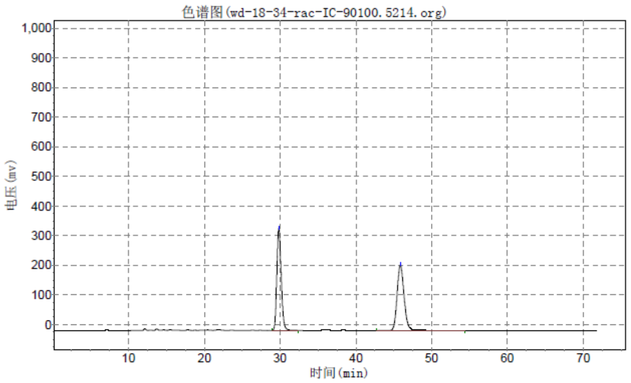

分析结果表

峰号	峰名	保留时间	峰高	峰面积	含量
1		29.857	343962.156	13845126.000	49.4353
2		45.857	219354.813	14161422.000	50.5647
总计			563316.969	28006548.000	100.0000

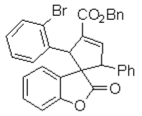

IC-H, *n*-Hexane:^{i}PrOH = 90:10, 0.5 ml/min, 214 nm

实验时间：2013-08-29, 17:39:16
谱图文件:I:\regio-and enantio\液相\wd-18-34-asy-re-IC-9010-0.5-214.org
实验者：
报告时间：2013-10-21, 22:34:43
积分方法:面积归一法

使用仪器类型:气相色谱 检测器:FID 进样器:分流

柱温:程序升温

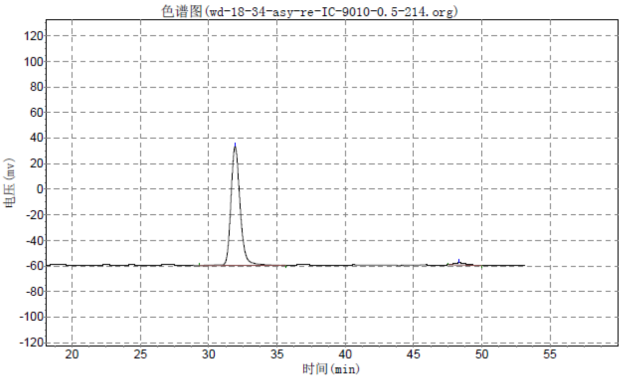

分析结果表

峰号	峰名	保留时间	峰高	峰面积	含量
1		31.928	93077.141	4356865.500	97.4257
2		48.333	1841.073	115122.773	2.5743
总计			94918.213	4471988.273	100.0000

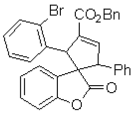

IC-H, *n*-Hexane:^{i}PrOH = 90:10, 0.5 ml/min, 214 nm

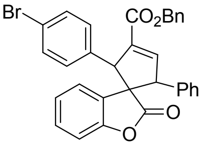

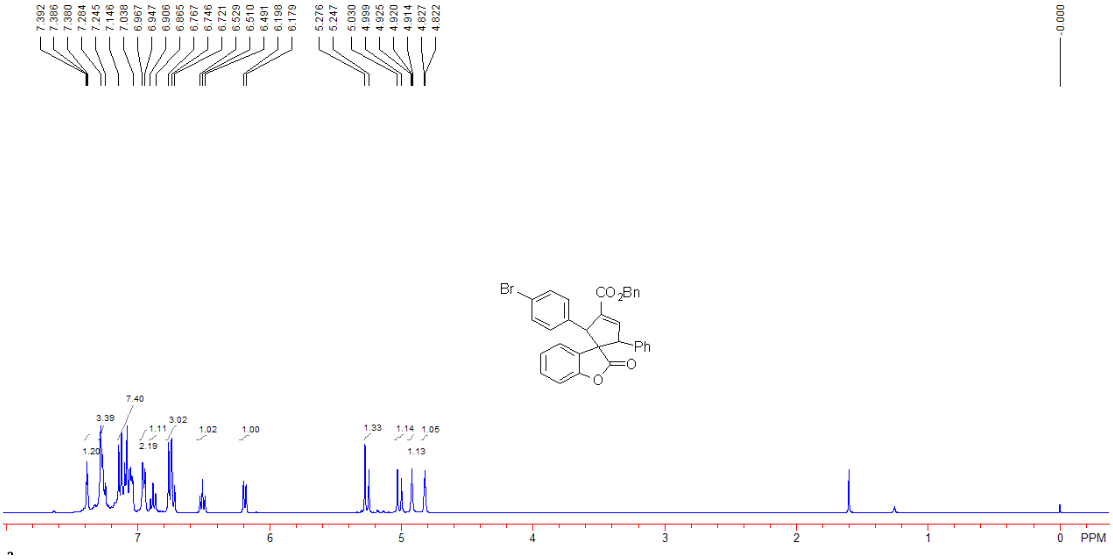

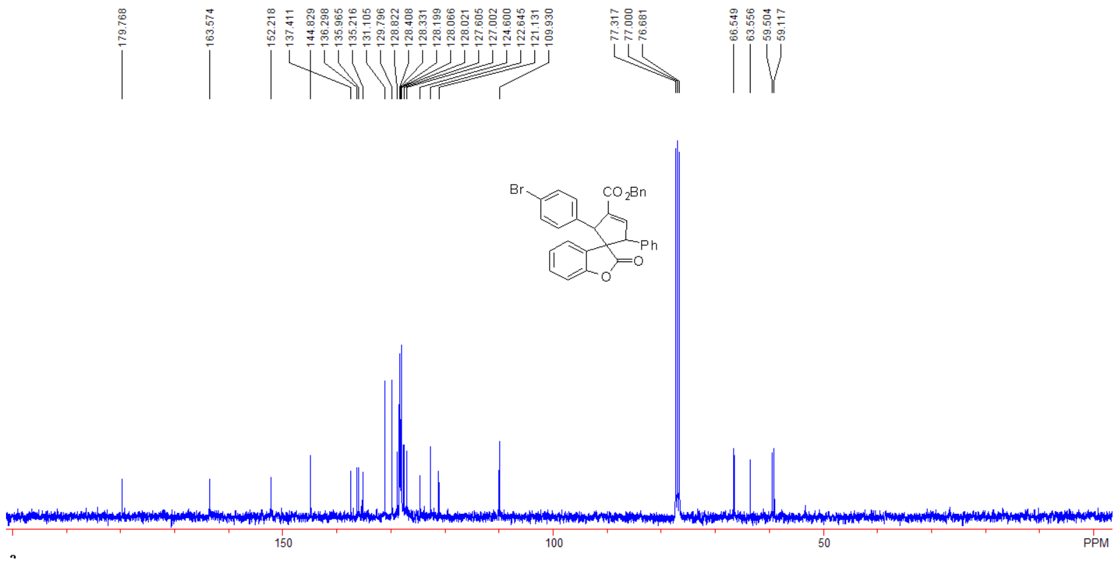

Benzyl 2'-(4-bromophenyl)-2-oxo-5'-phenyl-2H-spiro[benzofuran-3,1'-cyclopent[3]ene]-3'-carboxylate (5b)

A pale yellow solid, 91% yield, 49 mg, Mp: 68-70 ºC. ^{1}H NMR (400 MHz, $CDCl_3$, TMS) δ 7.39 (t, J = 2.4 Hz, 1H), 7.28-7.25 (m, 3H), 7.15-7.04 (m, 7H), 6.97-6.95 (m, 2H), 6.91-6.87 (m, 1H), 6.77-6.72 (m, 3H), 6.51 (t, J = 7.6 Hz, 1H), 6.19 (d, J = 7.2 Hz, 1H), 5.26 (d, J = 12.4 Hz, 1H), 5.01 (d, J = 12.4 Hz, 1H), 4.92 (t, J = 2.0 Hz, 1H), 4.82 (t, J = 2.0 Hz, 1H); ^{13}C NMR (100 MHz, $CDCl_3$) δ 179.8, 163.6, 152.2, 144.8, 137.4, 136.3, 136.0, 135.2, 131.1, 129.8, 128.8, 128.4, 128.3, 128.2, 128.1, 128.0, 127.6, 127.0, 124.6, 122.6, 121.1, 109.9, 66.5, 63.6, 59.5, 59.1; IR (neat) ν 3025, 1796, 1716, 1617, 1462, 1229, 1131, 1073, 1010, 751 cm^{-1}; HRMS Calcd. for $C_{32}H_{27}BrNO_4^{+1}$ $(M+NH_4)^+$: 568.1118, found: 568.1107. $[\alpha]^{20}_D$ = -96.3 (c 1.2, $CHCl_3$) for 85% ee; Enantiomeric excess was determined by HPLC with a Chiralcel AD-H column, Hexane/^{i}PrOH = 90/10, 0.6 mL/min, 214 nm, t_{minor} = 28.120 min, t_{major} = 19.770 min.

实验时间：2013-08-22, 14:46:53
谱图文件:I:\regio-and enantio\液相\wd-18-62-race-AD-9010-0.6-214.org

实验者：
报告时间：2013-11-05, 9:25:20
积分方法:面积归一法

使用仪器类型:气相色谱 检测器:FID 进样器:分流

柱温:程序升温

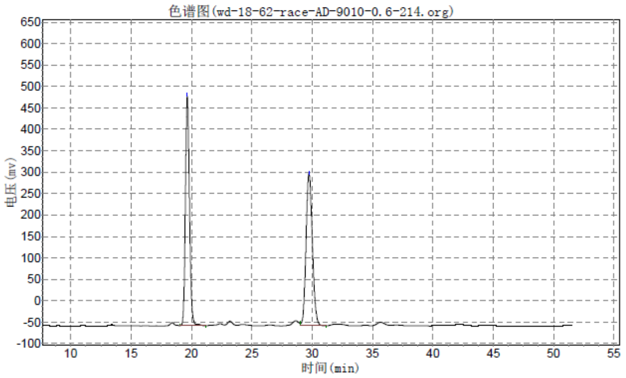

分析结果表

峰号	峰名	保留时间	峰高	峰面积	含量
1		19.665	533911.438	12859218.000	49.8389
2		29.765	355548.500	12942371.000	50.1611
总计			889459.938	25801589.000	100.0000

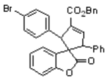

AD-H, Hexane:^{i}PrOH = 90:10, 0.6 ml/min, 214 nm

实验时间：2013-08-22, 16:20:37
谱图文件:I:\regio-and enantio\液相\wd-19-44-asy-AD-9010-0.6-214.org

实验者：
报告时间：2013-11-05, 9:24:19
积分方法:面积归一法

使用仪器类型:气相色谱 检测器:FID 进样器:分流

柱温:程序升温

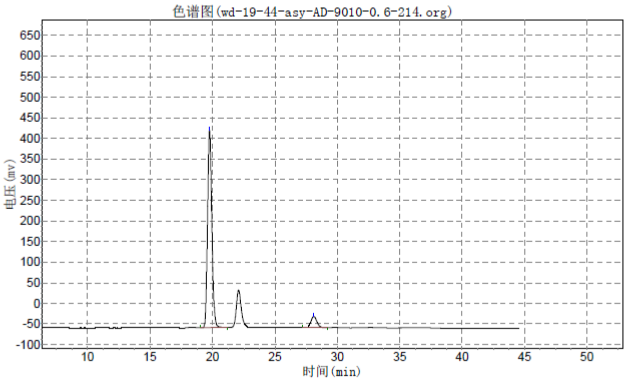

分析结果表

峰号	峰名	保留时间	峰高	峰面积	含量
1		19.770	479001.156	11472860.000	92.4778
2		28.120	27652.422	933209.250	7.5222
总计			506653.578	12406069.250	100.0000

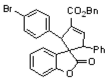

AD-H, Hexane:^{i}PrOH = 90:10, 0.6 ml/min, 214 nm

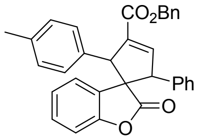

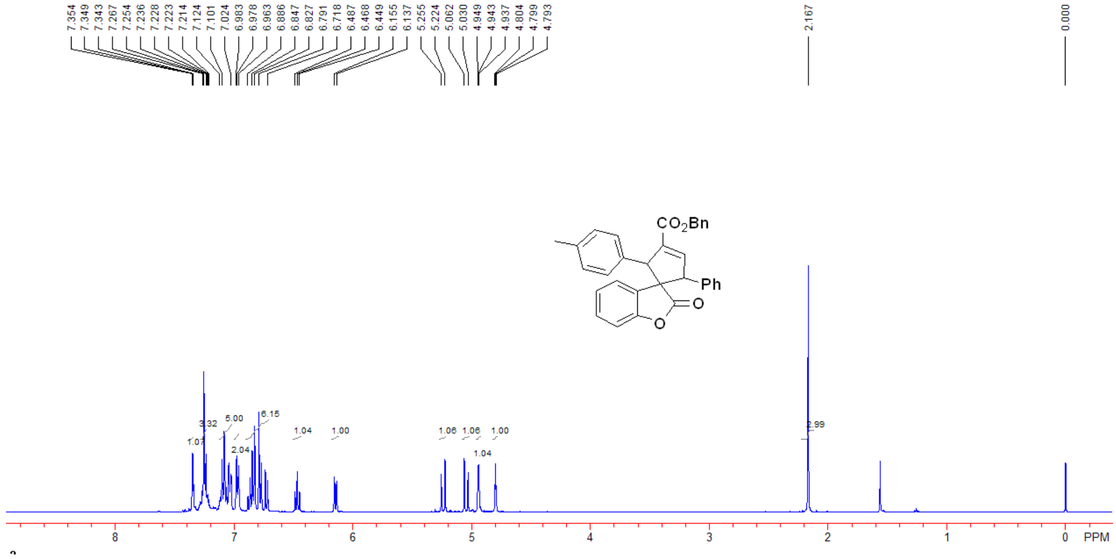

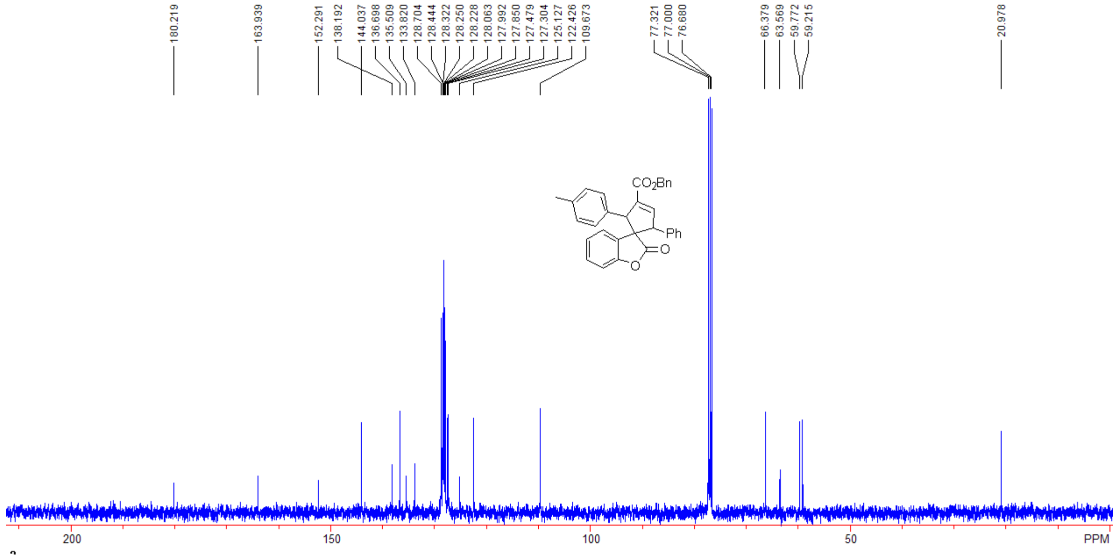

Benzyl 2-oxo-5'-phenyl-2'-(p-tolyl)-2H-spiro[benzofuran-3,1'-cyclopent[3]ene]-3'-carboxylate (5c)

A yellow solid, 72% yield, 28 mg, Mp: 56-58 ºC. ^{1}H NMR (400 MHz, $CDCl_3$, TMS) δ 7.35 (t, J = 2.4 Hz, 1H), 7.27-7.21 (m, 3H), 7.12-7.02 (m, 5H), 6.98-6.96 (m, 2H), 6.89-6.72 (m, 6H), 6.47 (t, J = 7.6 Hz, 1H), 6.15 (d, J = 7.2 Hz, 1H), 5.24 (d, J = 12.8 Hz, 1H), 5.05 (d, J = 12.8 Hz, 1H), 4.94 (t, J = 2.4 Hz, 1H), 4.80 (t, J = 2.4 Hz, 1H), 2.17 (s, 3H); ^{13}C NMR (100 MHz, $CDCl_3$) δ 180.2, 163.9, 152.3, 144.0, 138.2, 136.7, 135.5, 133.8, 128.7, 128.4, 128.32, 128.25, 128.2, 128.1, 128.0, 127.9, 127.5, 127.3, 125.1, 122.4, 109.7, 66.4, 63.6, 59.8, 59.2, 21.0; IR (neat) ν 2922, 1796, 1716, 1615, 1462, 1229, 1073, 964, 750 cm^{-1}; HRMS Calcd. for $C_{33}H_{30}NO_4^{+1}$ $(M+NH_4)^+$:

504.2169, found: 504.2167. $[\alpha]^{20}_{D}$ = -26.1 (c 1.2, $CHCl_3$) for 98% ee; Enantiomeric excess was determined by HPLC with a Chiralcel AD-H column, Hexane/^{i}PrOH = 90/10, 0.6 mL/min, 214 nm, t_{minor} = 21.765 min, t_{major} = 16.378 min.

N2000 数据工作站 1

实验时间：2013-05-14, 13:12:18
谱图文件:I:\regio-and enantio\液相\wd-18-20-rac-re-90100.6214.org

实验者：
报告时间：2013-10-21, 22:20:37
积分方法:面积归一法

使用仪器类型:气相色谱 检测器:FID 进样器:分流

柱温:程序升温

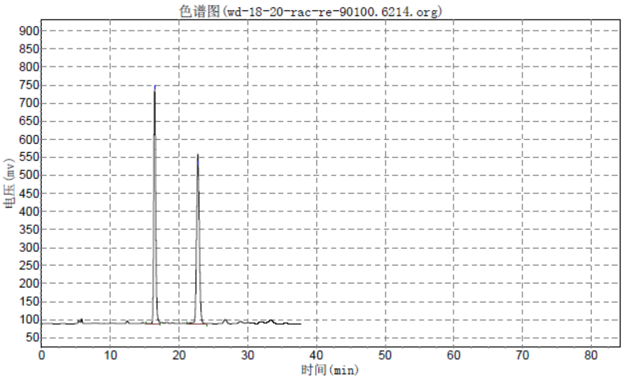

分析结果表

峰号	峰名	保留时间	峰高	峰面积	含量
1		16.465	657060.375	12942486.000	49.6584
2		22.798	461128.813	13120544.000	50.3416
总计			1118189.188	26063030.000	100.0000

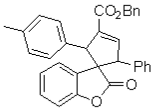

AD-H, *n*-Hexane:^{i}PrOH = 90:10, 0.6 ml/min, 214 nm

实验时间：2013-05-14, 12:34:08
谱图文件:I:\regio-and enantio\液相\wd-18-20-asy-re-90100.6214.org

实验者:
报告时间：2013-10-21, 22:27:45
积分方法:面积归一法

使用仪器类型:气相色谱　　检测器:FID　　进样器:分流

柱温:程序升温

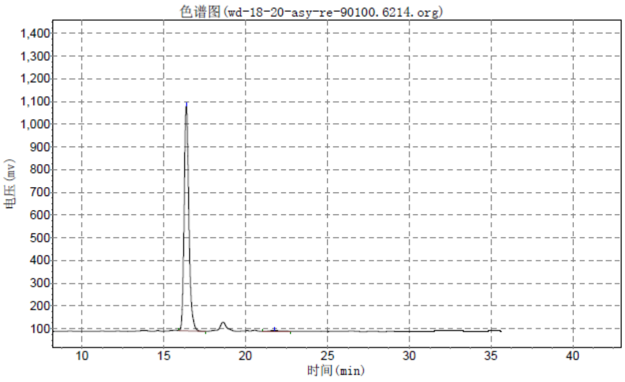

分析结果表

峰号	峰名	保留时间	峰高	峰面积	含量
1		16.378	990828.188	19891498.000	99.1750
2		21.765	3950.094	165461.094	0.8250
总计			994778.281	20056959.094	100.0000

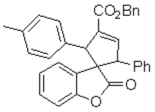

AD-H, *n*-Hexane:^{i}PrOH = 90:10, 0.6 ml/min, 214 nm

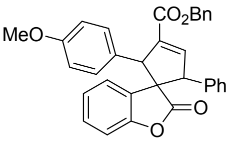

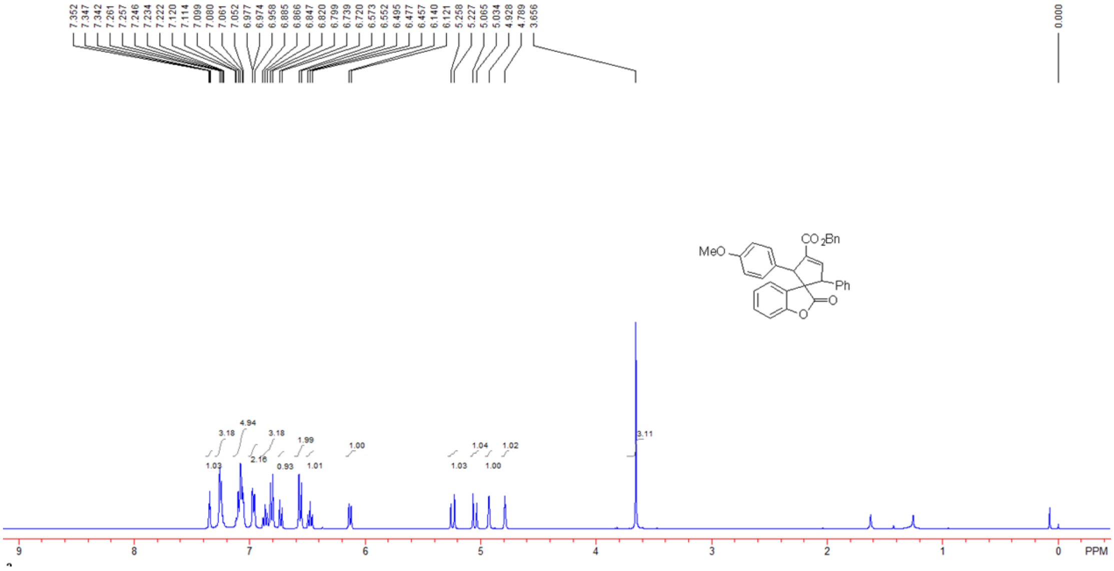

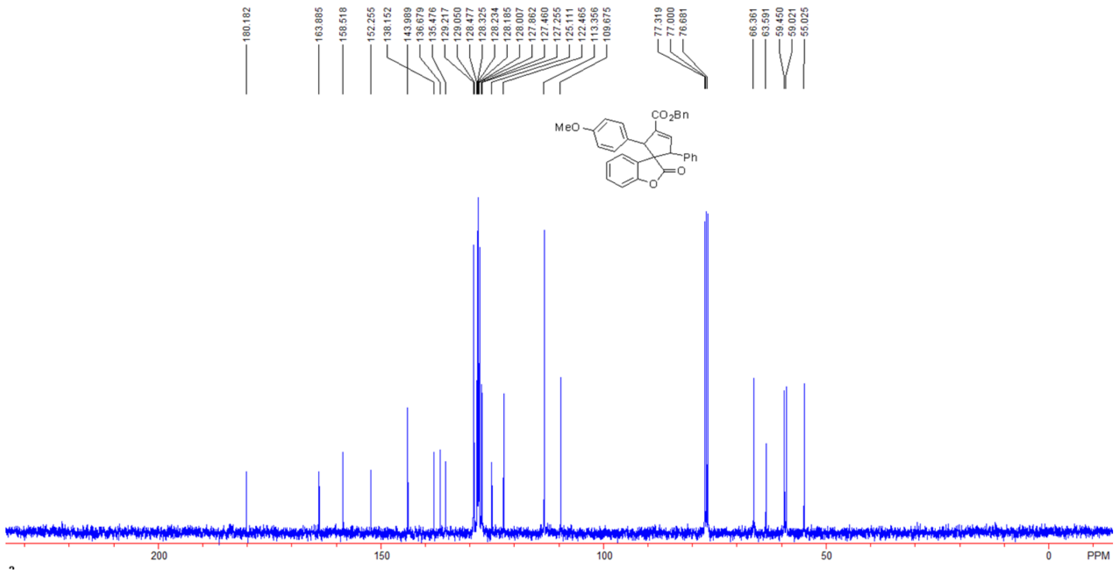

Benzyl 2'-(4-methoxyphenyl)-2-oxo-5'-phenyl-2H-spiro[benzofuran-3,1'-cyclopent[3]ene]-3'-carboxylate (5d)

A white solid, 68% yield, 34 mg, Mp: 58-60 ºC. ^{1}H NMR (400 MHz, $CDCl_3$, TMS) δ 7.35 (t, J = 2.0 Hz, 1H), 7.26-7.22 (m, 3H), 7.12-7.05 (m, 5H), 6.98-6.96 (m, 2H), 6.89-6.80 (m, 3H), 6.73 (d, J = 7.6 Hz, 1H), 6.56 (d, J = 8.4 Hz, 2H), 6.48 (t, J = 8.0 Hz, 1H), 6.13 (d, J = 7.6 Hz, 1H), 5.24 (d, J = 12.4 Hz, 1H), 5.05 (d, J = 12.4 Hz, 1H), 4.93 (s, 1H), 4.79 (s, 1H), 3.66 (s, 3H); ^{13}C NMR (100 MHz, $CDCl_3$) δ 180.2, 163.9, 158.5, 152.3, 144.0, 138.2, 136.7, 135.5, 129.2, 129.1, 128.5, 128.3, 128.23, 128.19, 128.0, 127.9, 127.5, 127.3, 125.1, 122.5, 113.4, 109.7, 66.4, 63.6, 59.5, 59.0, 55.0; IR (neat) ν 2921, 1797, 1719, 1513, 1462, 1275, 1260, 1074, 750 cm^{-1}; HRMS Calcd. for $C_{33}H_{30}NO_5^{+1}$ $(M+NH_4)^+$: 520.2118, found: 520.2110. $[\alpha]^{20}_D$ = -66.4 (c 1.0, $CHCl_3$) for 99% ee; Enantiomeric excess was determined by HPLC with a Chiralcel IC-H column, Hexane/^{i}PrOH = 90/10, 0.65 mL/min, 214 nm, t_{minor} = 27.195 min, t_{major} = 31.582 min.

实验时间：2013-05-29, 15:05:52
谱图文件:I:\regio-and enantio\液相\wd-18-35-rac-IC-90100.65214.org

实验者：
报告时间：2013-10-21, 22:37:20
积分方法:面积归一法

使用仪器类型:气相色谱　　检测器:FID　　进样器:分流

柱温:程序升温

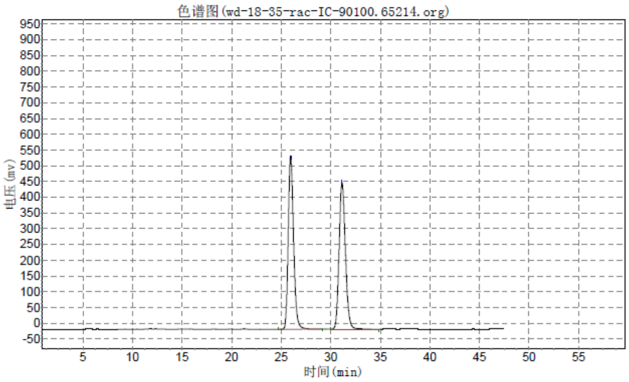

分析结果表

峰号	峰名	保留时间	峰高	峰面积	含量
1		25.998	548807.688	20971918.000	50.3040
2		31.132	460579.938	20718456.000	49.6960
总计			1009387.625	41690374.000	100.0000

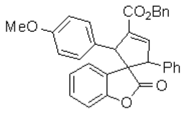

IC-H, *n*-Hexane:^{i}PrOH = 90:10, 0.65 ml/min, 214 nm

N2000 数据工作站 1

实验时间：2013-05-29, 15:52:32
谱图文件:I:\regio-and enantio\液相\wd-18-35-asy-IC-90100.65214.org

实验者：
报告时间：2013-10-21, 22:46:29
积分方法:面积归一法

使用仪器类型:气相色谱　　检测器:FID　　进样器:分流

柱温:程序升温

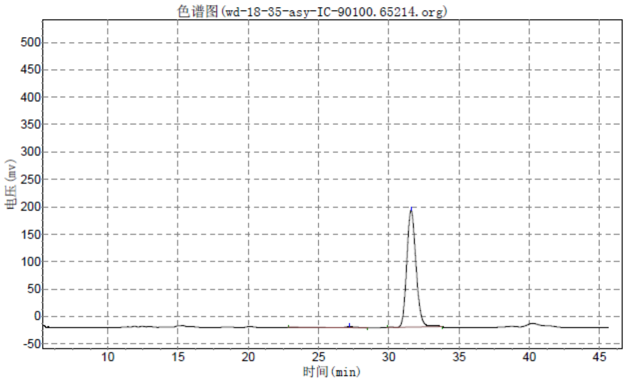

分析结果表

峰号	峰名	保留时间	峰高	峰面积	含量
1		27.195	1476.739	813.278	0.0083
2		31.582	213319.000	9763855.000	99.9917
总计			214795.739	9764668.278	100.0000

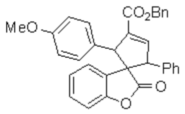

IC-H, *n*-Hexane:^{i}PrOH = 90:10, 0.65 ml/min, 214 nm

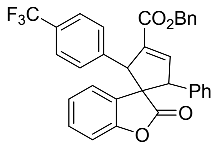
F3C
CO2Bn
Ph
O
O

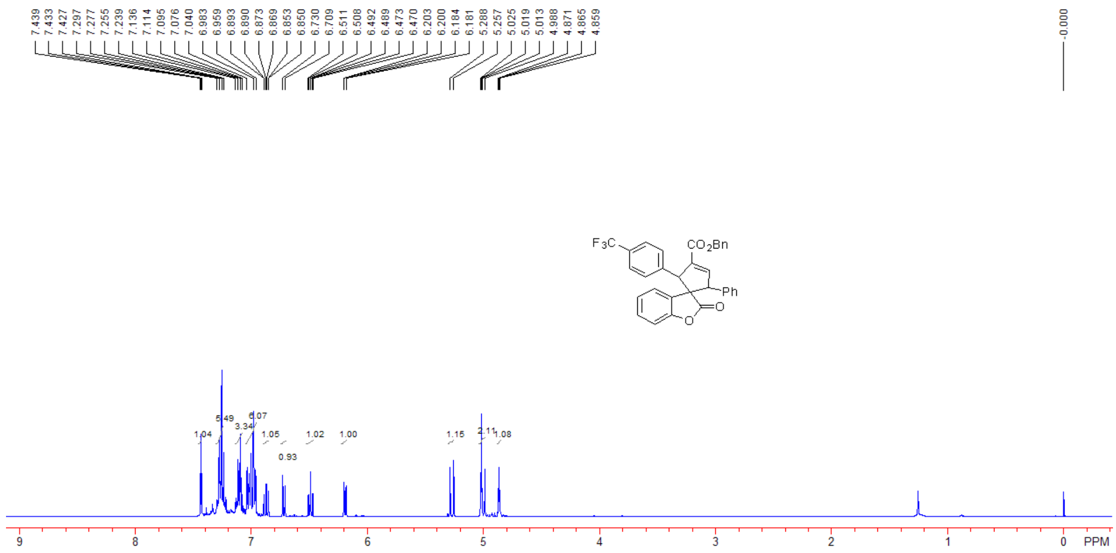
7.439 7.433 7.427 7.297 7.277 7.255 7.239 7.136 7.114 7.095 7.076 7.040 6.983 6.959 6.893 6.890 6.873 6.869 6.853 6.850 6.730 6.709 6.511 6.508 6.492 6.489 6.473 6.470 6.203 6.200 6.184 6.181 5.288 5.257 5.025 5.019 5.013 4.988 4.871 4.865 4.859
-0.000
1.04 5.49 3.34 6.07 1.05 0.93 1.02 1.00 1.15 2.11 1.08
9 8 7 6 5 4 3 2 1 0 PPM

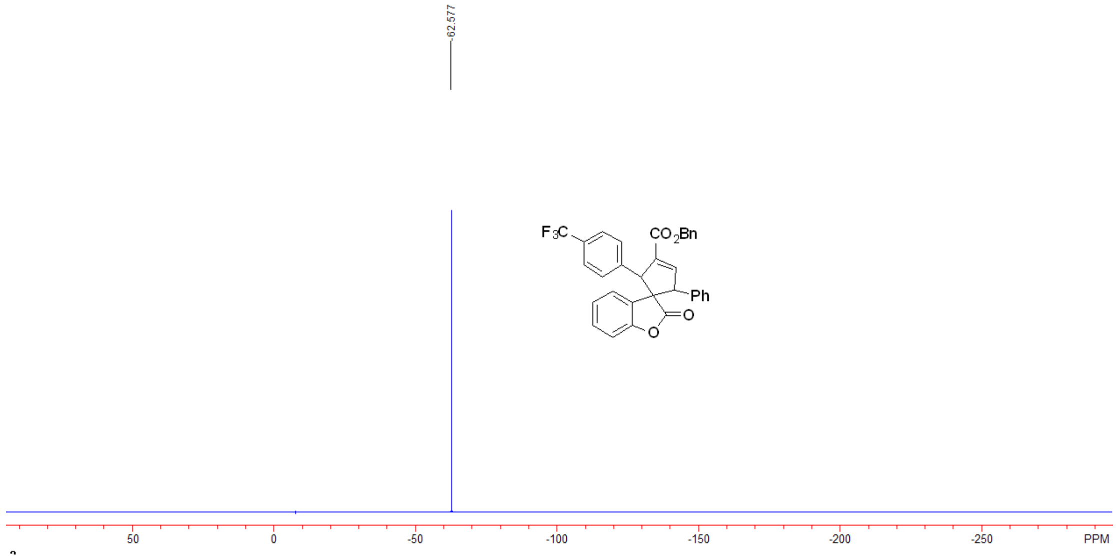
-62.577
50 0 -50 -100 -150 -200 -250 PPM

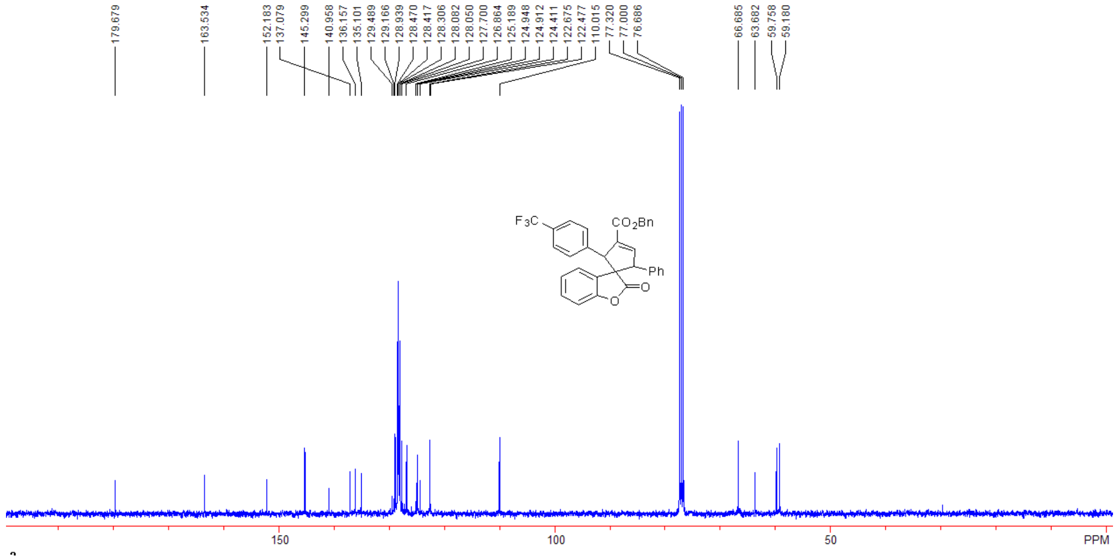
179.679 163.534 152.183 137.079 145.299 140.958 136.157 135.101 129.489 129.166 128.939 128.470 128.417 128.306 128.082 128.050 127.700 126.864 125.189 124.948 124.912 124.411 122.675 122.477 110.015 77.320 77.000 76.686 66.685 63.682 59.758 59.180
150 100 50 PPM

Benzyl 2-oxo-5'-phenyl-2'-(4-(trifluoromethyl)phenyl)-2H-spiro[benzofuran-3,1'-cyclopent[3]ene]-3'-carboxylate (5e)

A pale yellow solid, 92% yield, 49 mg, Mp: 53-54 ºC. ^{1}H NMR (400 MHz, $CDCl_3$, TMS) δ 7.43 (t, J = 2.4 Hz, 1H), 7.30-7.24 (m, 5H), 7.14-7.08 (m, 3H), 7.04-6.96 (m, 6H), 6.87 (dt, J_1 = 1.2 Hz, J_2 = 8.0 Hz, 1H), 6.73-6.71 (m, 1H), 6.49 (dt, J_1 = 1.2 Hz, J_2 = 7.6 Hz, 1H), 6.19 (dd, J_1 = 1.2 Hz, J_2 = 7.6 Hz, 1H), 5.27 (d, J = 12.4 Hz, 1H), 5.03-4.99 (m, 2H), 4.87 (t, J = 2.4 Hz, 1H); ^{13}C NMR (100 MHz, $CDCl_3$) δ 179.7, 163.5, 152.2, 145.3, 141.0, 137.1, 136.2, 135.1, 129.3 (q, J = 32.3 Hz), 128.9, 128.5, 128.4, 128.3, 128.1 (q, J = 3.2 Hz), 127.7, 126.9, 124.9 (q, J = 3.6 Hz), 123.8 (q, J = 271.2 Hz), 122.7, 110.0, 66.7, 63.7, 59.8, 59.2; ^{19}F NMR (376 MHz, $CDCl_3$) δ -62.58 (s); IR (neat) ν 3032, 1800, 1734, 1617, 1462, 1325, 1166, 1120, 1069, 751 cm^{-1}; HRMS Calcd. for $C_{33}H_{27}F_3NO_4^{+1}$ $(M+NH_4)^+$: 558.1887, found: 558.1873. $[\alpha]^{20}_D$ = -25.1 (c 0.9, $CHCl_3$) for 92% ee; Enantiomeric excess was determined by HPLC with a Chiralcel AD-H column, Hexane/^{i}PrOH = 80/20, 0.5 mL/min, 214 nm, t_{minor} = 16.552 min, t_{major} = 13.116 min.

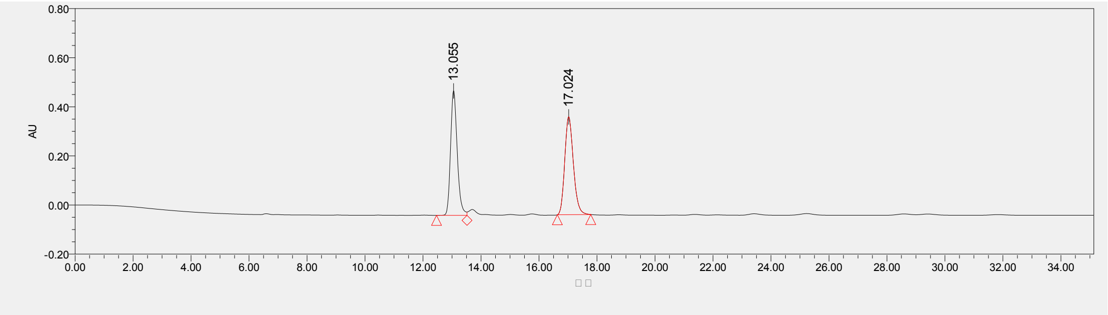

NO	R. Time	Peak Area	Precent	Peak Height
1	13.055	8086139	49.54	508061
2	17.042	8236122	50.46	398900

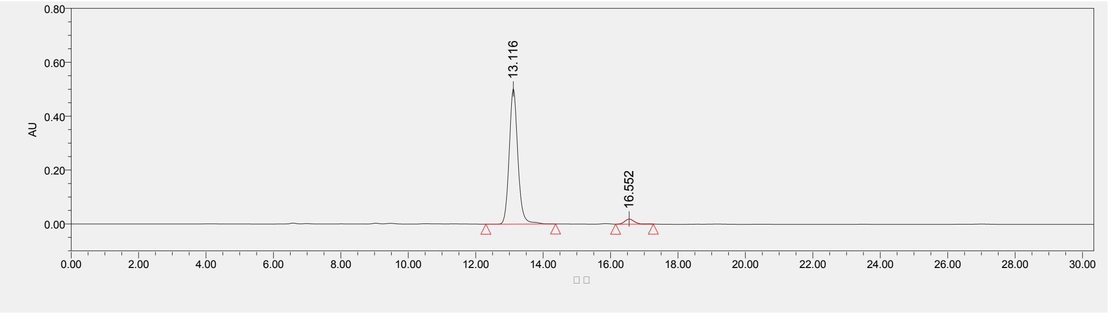

NO	R. Time	Peak Area	Precent	Peak Height
1	13.116	8912648	96.08	501668
2	16.552	363758	3.92	18661

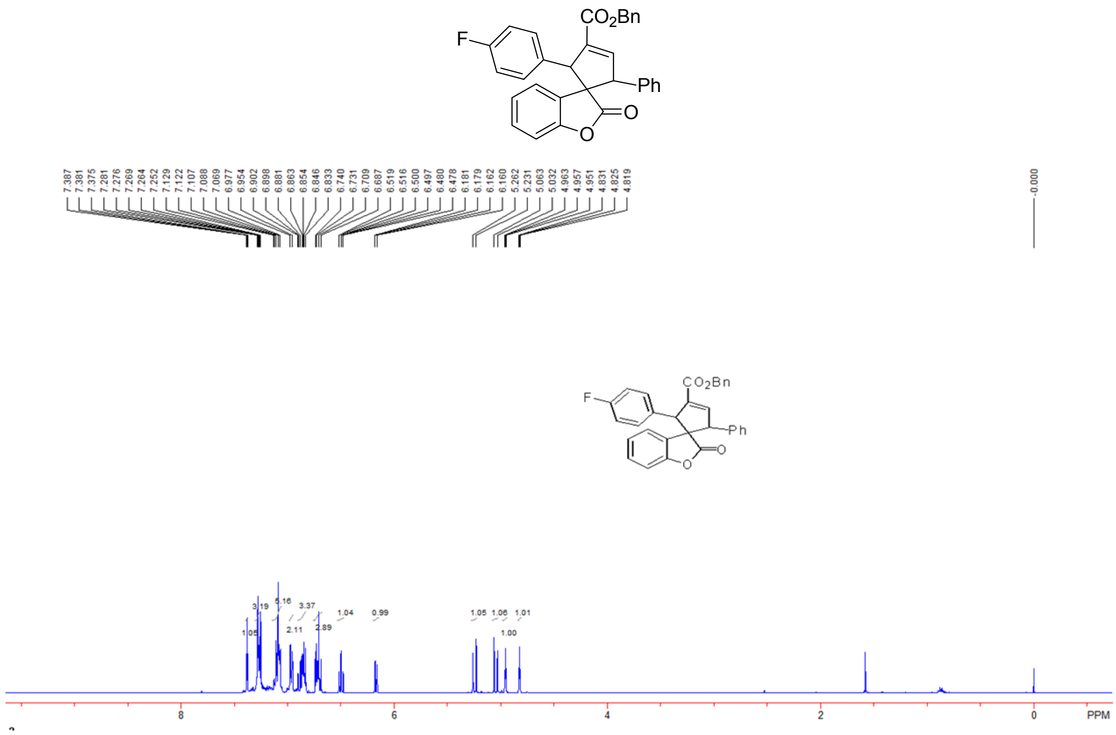
CO2Bn
F
Ph
O
O
7.387 7.381 7.375 7.281 7.276 7.269 7.264 7.252 7.129 7.122 7.107 7.088 7.069 6.977 6.954 6.902 6.898 6.881 6.863 6.854 6.846 6.833 6.740 6.731 6.709 6.687 6.519 6.516 6.500 6.497 6.480 6.478 6.181 6.179 6.162 6.160 5.262 5.231 5.063 5.032 4.963 4.957 4.951 4.831 4.825 4.819
-0.000
1.05 3.19 5.16 3.37 2.11 2.89 1.04 0.99 1.05 1.06 1.01 1.00
8
6
4
2
0
PPM

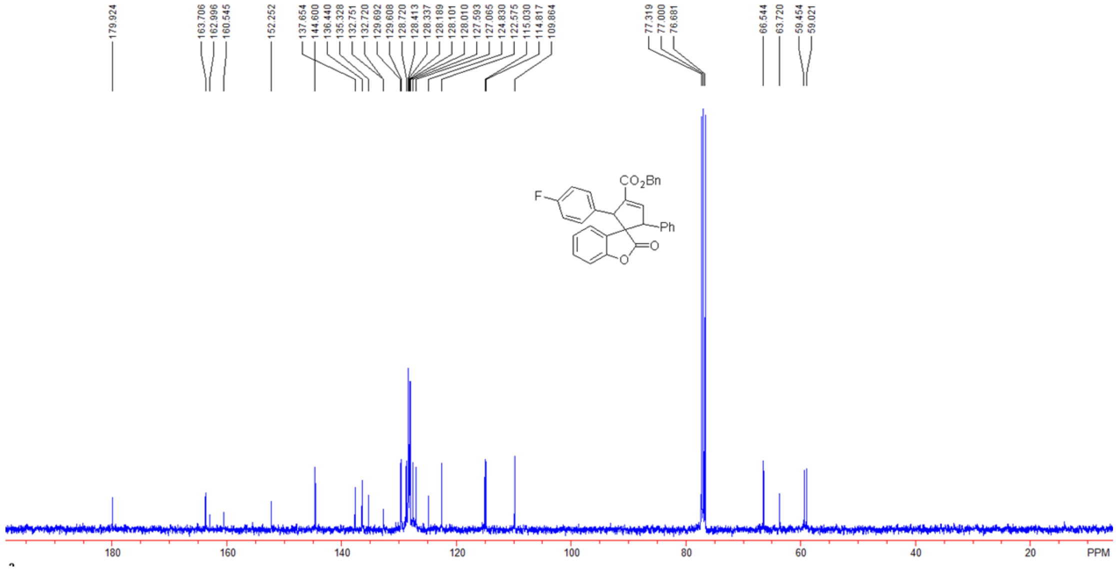
179.924
163.706 162.996 160.545
152.252
137.654 144.600 136.440 135.328 132.751 132.720 129.692 129.608 128.720 128.413 128.337 128.189 128.101 128.010 127.593 127.065 124.830 122.575 115.030 114.817 109.864
77.319 77.000 76.681
66.544 63.720 59.454 59.021
CO2Bn
F
Ph
O
O
180
160
140
120
100
80
60
40
20
PPM

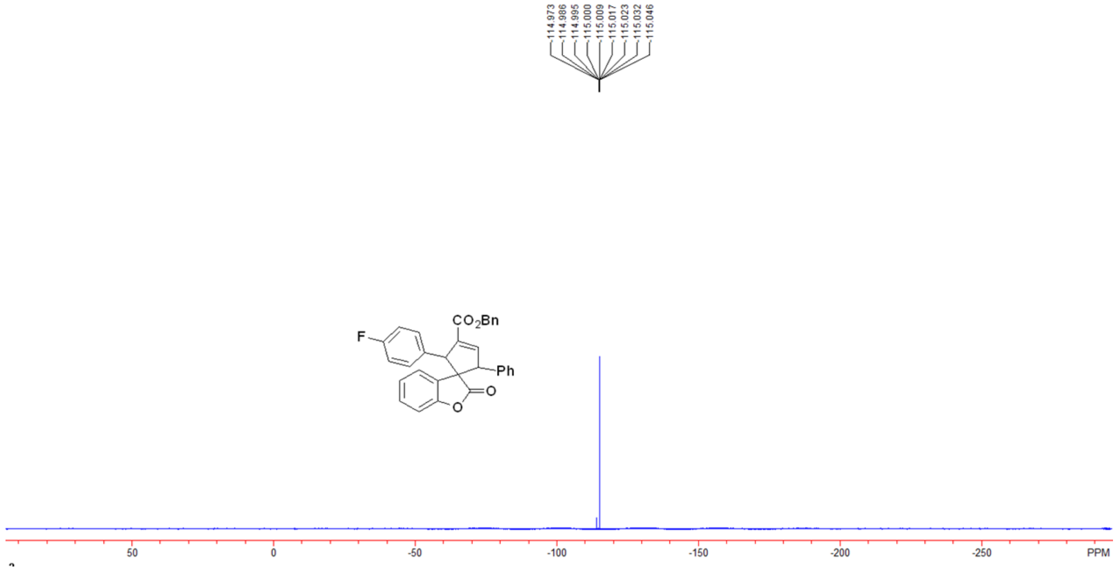
-114.973 -114.986 -114.995 -115.000 -115.009 -115.017 -115.023 -115.032 -115.046
CO2Bn
F
Ph
O
O
50
0
-50
-100
-150
-200
-250
PPM

Benzyl 2'-(4-fluorophenyl)-2-oxo-5'-phenyl-2H-spiro[benzofuran-3,1'-cyclopent[3]ene]-3'-carboxylate (5f)

A pale yellow solid, 78% yield, 38 mg, Mp: 60-61 ºC. ^{1}H NMR (400 MHz, $CDCl_3$, TMS) δ 7.38 (t, J = 2.4 Hz, 1H), 7.28-7.25 (m, 3H), 7.13-7.07 (m, 5H), 6.98-6.95 (m, 2H), 6.90-6.83 (m, 3H), 6.74-6.69 (m, 3H), 6.50 (dt, J_1 = 1.2 Hz, J_2 = 7.6 Hz, 1H), 6.17 (dd, J_1 = 0.8 Hz, J_2 = 7.6 Hz, 1H), 5.25 (d, J = 12.4 Hz, 1H), 5.05 (d, J = 12.4 Hz, 1H), 4.96 (t, J = 2.4 Hz, 1H), 4.83 (t, J = 2.4 Hz, 1H); ^{13}C NMR (100 MHz, $CDCl_3$) δ 179.9, 163.7, 161.8 (d, J = 245.1 Hz), 152.3, 144.6, 137.7, 136.4, 135.3, 132.7 (d, J = 3.1 Hz), 129.7 (d, J = 8.4 Hz), 128.7, 128.4, 128.3, 128.2, 128.1, 128.0, 127.6, 127.1, 124.8, 122.6, 114.9 (d, J = 21.3 Hz), 109.9, 66.5, 63.7, 59.5, 59.0; ^{19}F NMR (376 MHz, $CDCl_3$) δ -114.97~-115.05 (m); IR (neat) ν 2915, 1796, 1716, 1617, 1508, 1461, 1225, 1073, 965, 750, 696 cm^{-1}; HRMS Calcd. for $C_{32}H_{27}FNO_4^{+1}$ $(M+NH_4)^+$: 508.1919, found: 508.1911. $[\alpha]^{20}_D$ = -36.5 (c 0.8, $CHCl_3$) for 97% ee; Enantiomeric excess was determined by HPLC with a Chiralcel AD-H column, Hexane/^{i}PrOH = 90/10, 0.5 mL/min, 214 nm, t_{minor} = 26.629 min, t_{major} = 16.367 min.

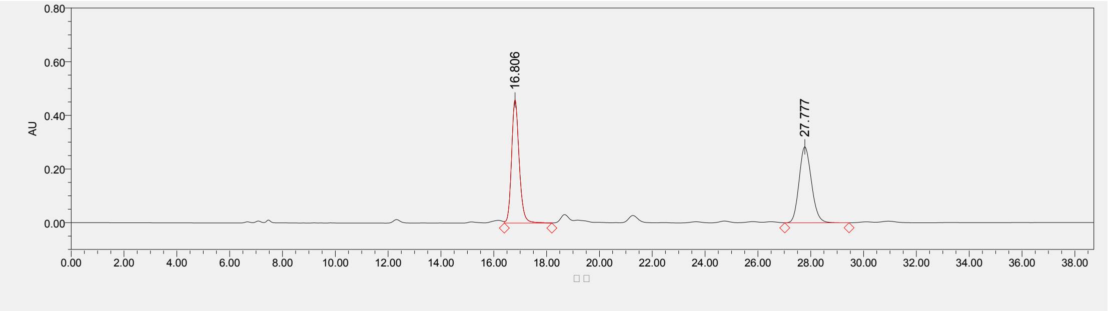

NO	R. Time	Peak Area	Precent	Peak Height
1	16.806	9192003	49.81	459594
2	27.777	9262713	50.19	283434

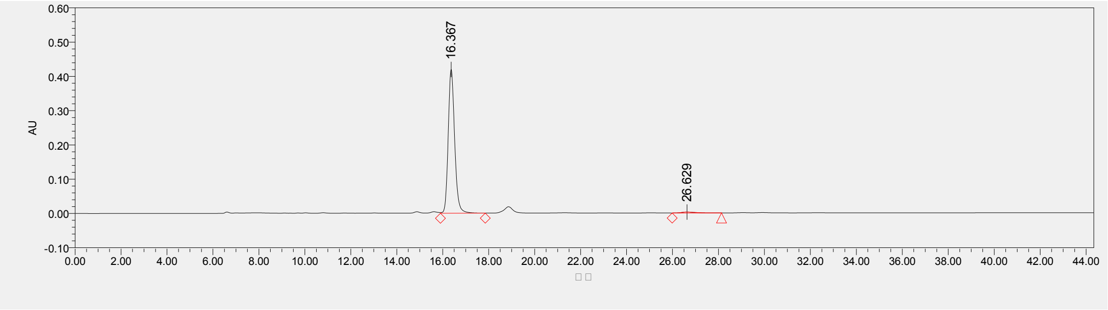

NO	R. Time	Peak Area	Precent	Peak Height
1	16.367	8163954	98.64	419385
2	26.629	112587	1.36	2825

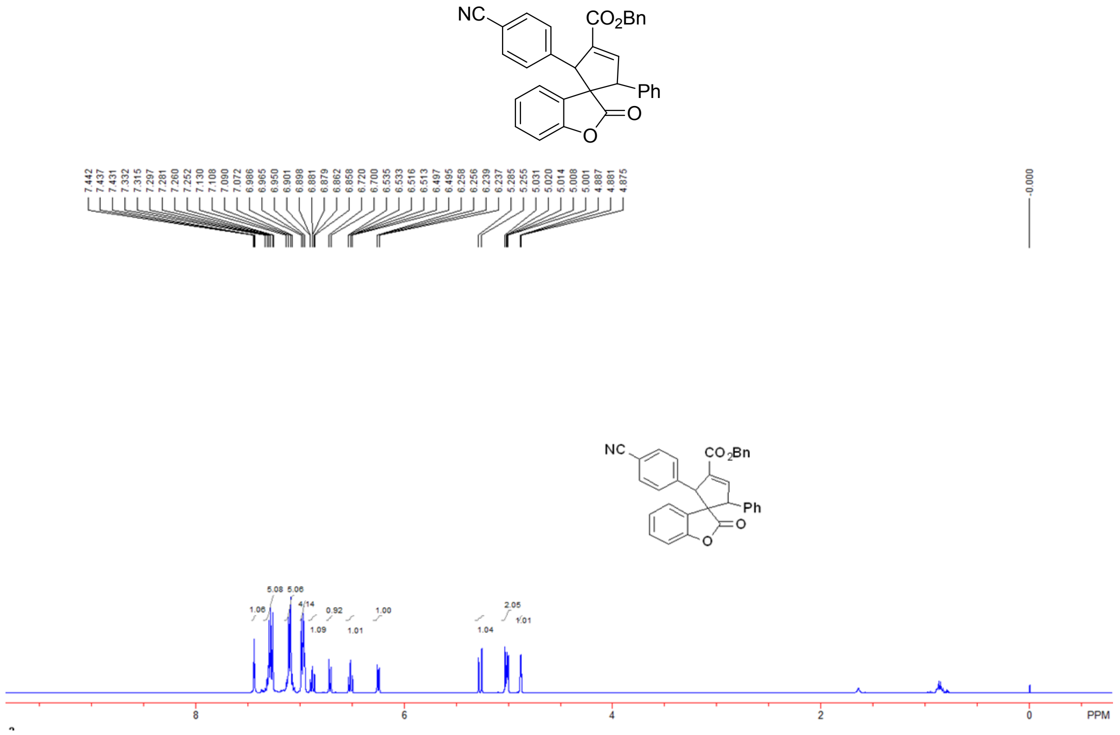

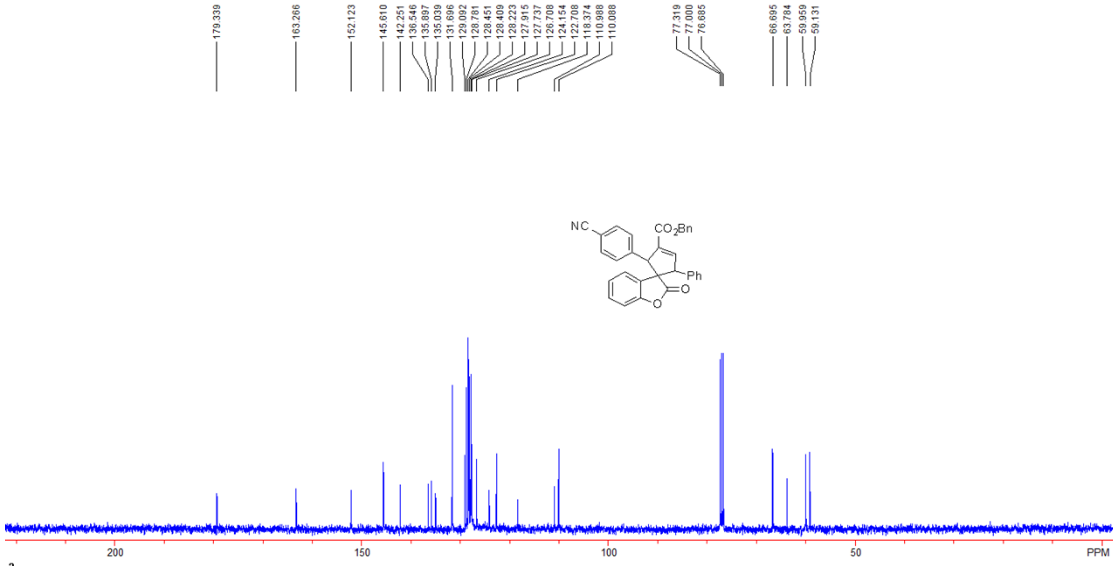

Benzyl 2'-(4-cyanophenyl)-2-oxo-5'-phenyl-2H-spiro[benzofuran-3,1'-cyclopent[3]ene]-3'-carboxylate (5g)

A pale yellow solid, 75% yield, 37 mg, Mp: 160-161 ºC. ^{1}H NMR (400 MHz, $CDCl_3$, TMS) δ 7.44 (t, J = 2.4 Hz, 1H), 7.33-7.25 (m, 5H), 7.13-7.07 (m, 5H), 6.99-6.95 (m, 4H), 6.90-6.83 (m, 3H), 6.88 (dt, J = 1.2 Hz, J = 8.0 Hz, 1H), 6.71 (d, J = 8.0 Hz, 1H), 6.51 (dt, J_1 = 0.8 Hz, J_2 = 7.6 Hz, 1H), 6.25 (dd, J_1 = 0.8 Hz, J_2 = 7.6 Hz, 1H), 5.27 (d, J = 12.0 Hz, 1H), 5.02 (d, J = 12.0 Hz, 1H), 5.01 (t, J = 2.4 Hz, 1H), 4.88 (t, J = 2.4 Hz, 1H); ^{13}C NMR (100 MHz, $CDCl_3$) δ 179.3, 163.3, 152.1, 145.6, 142.3, 136.5, 135.9, 135.0, 131.7, 129.1, 128.8, 128.5, 128.4, 128.2, 127.9, 127.7, 126.7, 124.2, 122.7, 118.4, 111.0, 110.1, 66.7, 63.8, 60.0, 59.1; IR (neat) ν 3015, 1796,

1716, 1462, 1275, 1261, 1074, 749, 697 cm^{-1}; HRMS Calcd. for $C_{33}H_{27}N_2O_4^{+1}$ $(M+NH_4)^+$: 515.1965, found: 515.1959. $[\alpha]^{20}_{D}$ = -142.6 (c 0.75, $CHCl_3$) for 99% ee; Enantiomeric excess was determined by HPLC with a Chiralcel AD-H column, Hexane/^{i}PrOH = 80/20, 0.6 mL/min, 214 nm, t_{minor} = 25.448 min, t_{major} = 20.886 min.

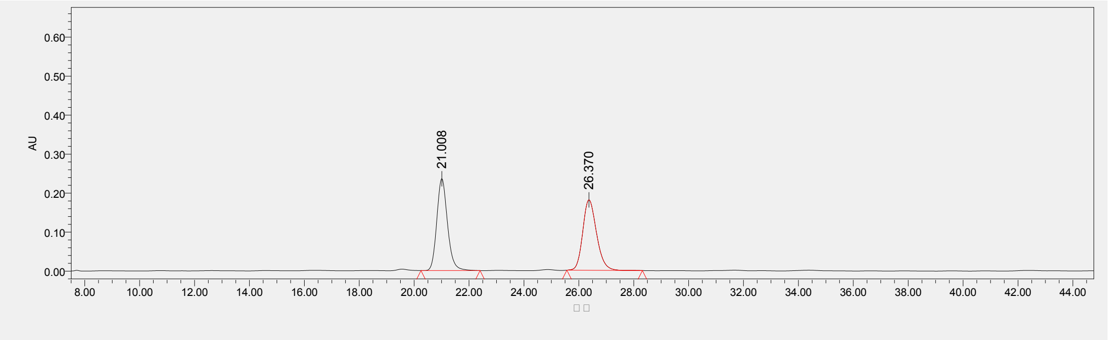

NO	R. Time	Peak Area	Precent	Peak Height
1	21.008	6396516	50.36	235759
2	26.370	6305550	49.64	180561

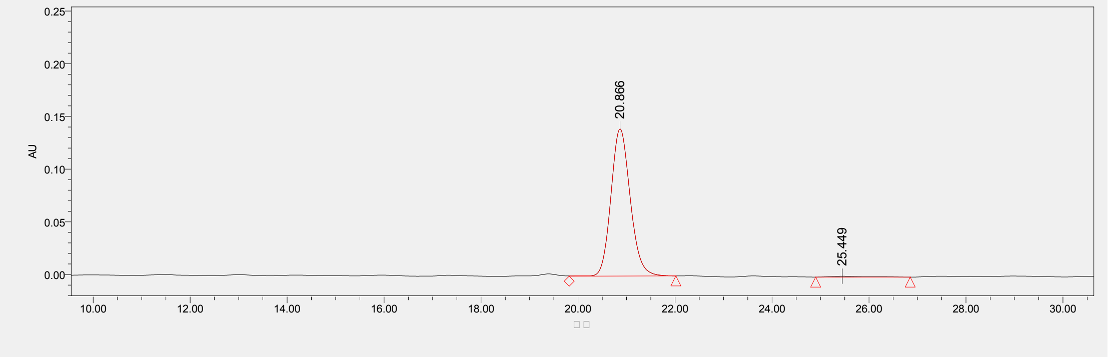

NO	R. Time	Peak Area	Precent	Peak Height
1	20.886	3856858	99.38	139603
2	25.448	24196	0.62	639

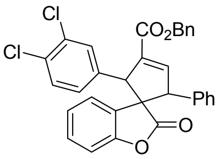

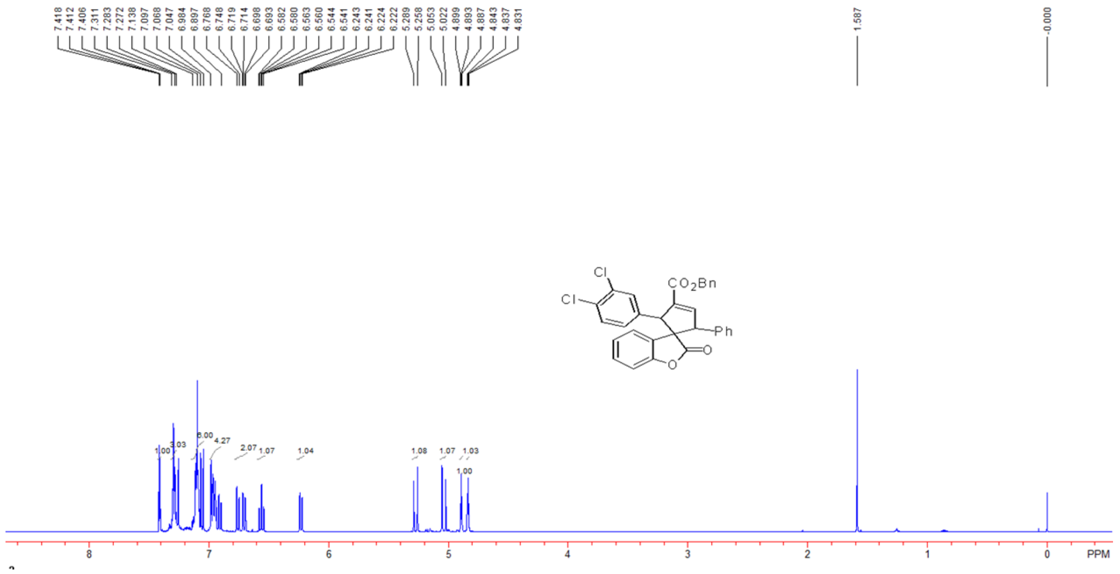

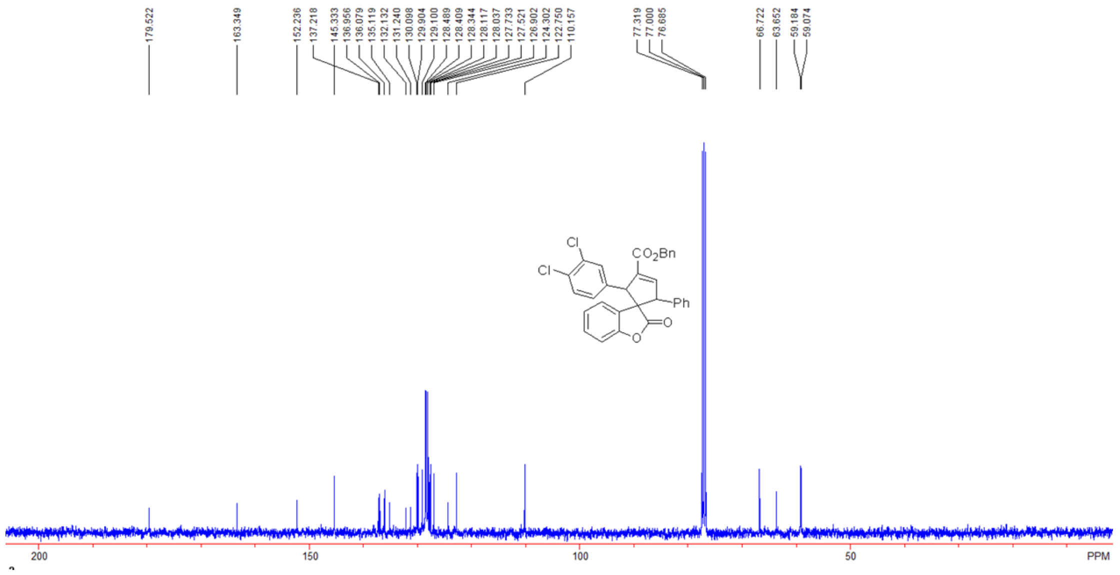

Benzyl 2'-(3,4-dichlorophenyl)-2-oxo-5'-phenyl-2H-spiro[benzofuran-3,1'-cyclopent[3]ene]-3'-carboxylate (5h)

A pale yellow solid, 82% yield, 44 mg, Mp: 63-65 ºC. ^{1}H NMR (400 MHz, $CDCl_3$, TMS) δ 7.41 (t, J = 2.4 Hz, 1H), 7.31-7.27 (m, 3H), 7.14-7.05 (m, 6H), 6.98-6.90 (m, 4H), 6.77-6.69 (m, 2H), 6.56 (dt, J_1 = 1.2 Hz, J_2 = 7.6 Hz, 1H), 6.23 (dd, J_1 = 0.8 Hz, J_2 = 7.6 Hz, 1H), 5.27 (d, J = 12.4 Hz, 1H), 5.04 (d, J = 12.4 Hz, 1H), 4.89 (t, J = 2.4 Hz, 1H), 4.84 (t, J = 2.4 Hz, 1H); ^{13}C NMR (100 MHz, $CDCl_3$) δ 179.5, 163.3, 152.2, 145.3, 137.2, 137.0, 136.1, 135.1, 132.1, 131.2, 130.1, 129.9, 129.1, 128.5, 128.4, 128.3, 128.1, 128.0, 127.7, 127.5, 126.9, 124.3, 122.8, 110.2, 66.7, 63.7, 59.2, 59.1; IR (neat) ν 2923, 1797, 1716, 1617, 1462, 1275, 1260, 1074, 750 cm^{-1}; HRMS Calcd. for $C_{32}H_{26}Cl_2NO_4^{+1}$ $(M+NH_4)^+$: 558.1233, found: 558.1226. $[\alpha]^{20}_D$ = -84.2 (c 2.0, $CHCl_3$) for >99% ee; Enantiomeric excess was determined by HPLC with a Chiralcel AD-H column, Hexane/^{i}PrOH = 90/10, 0.6 mL/min, 214 nm, t_{major} = 14.156 min.

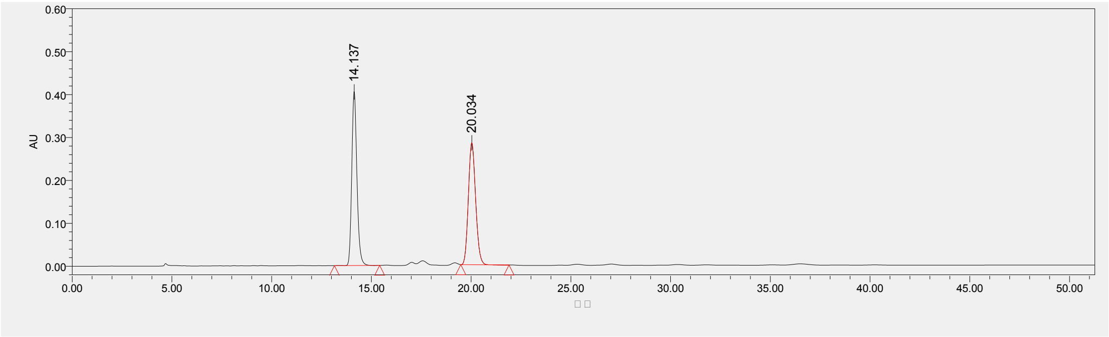

NO	R. Time	Peak Area	Precent	Peak Height
1	14.137	7268695	50.20	405150
2	20.034	7210046	49.80	284032

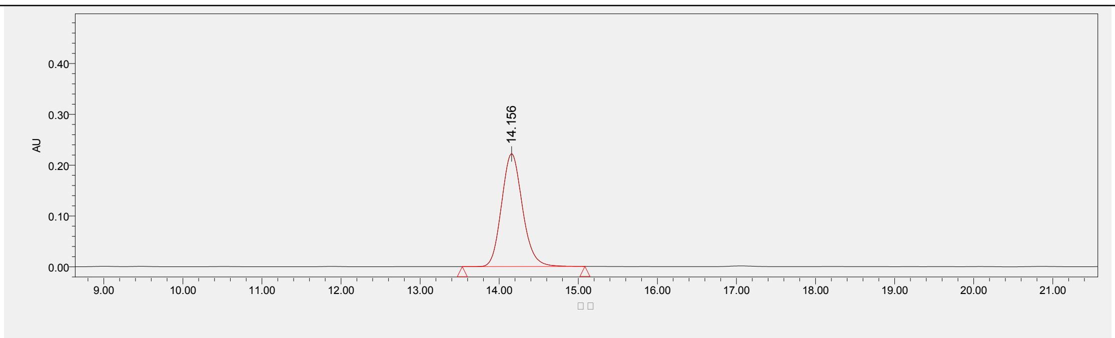

NO	R. Time	Peak Area	Precent	Peak Height
1	14.156	4072800	100.00	222094

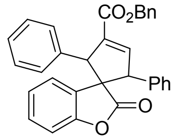

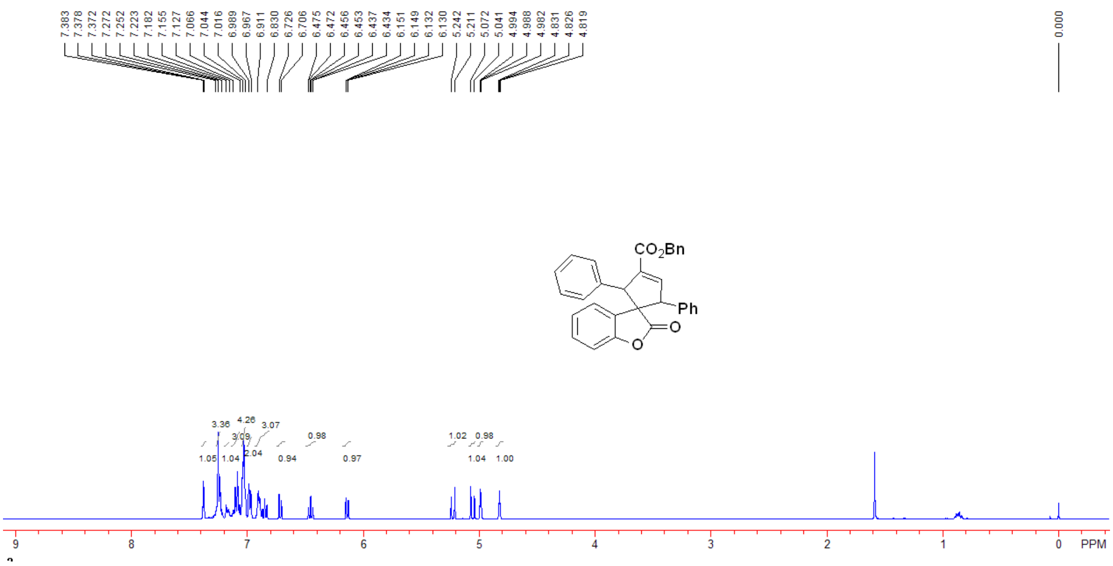

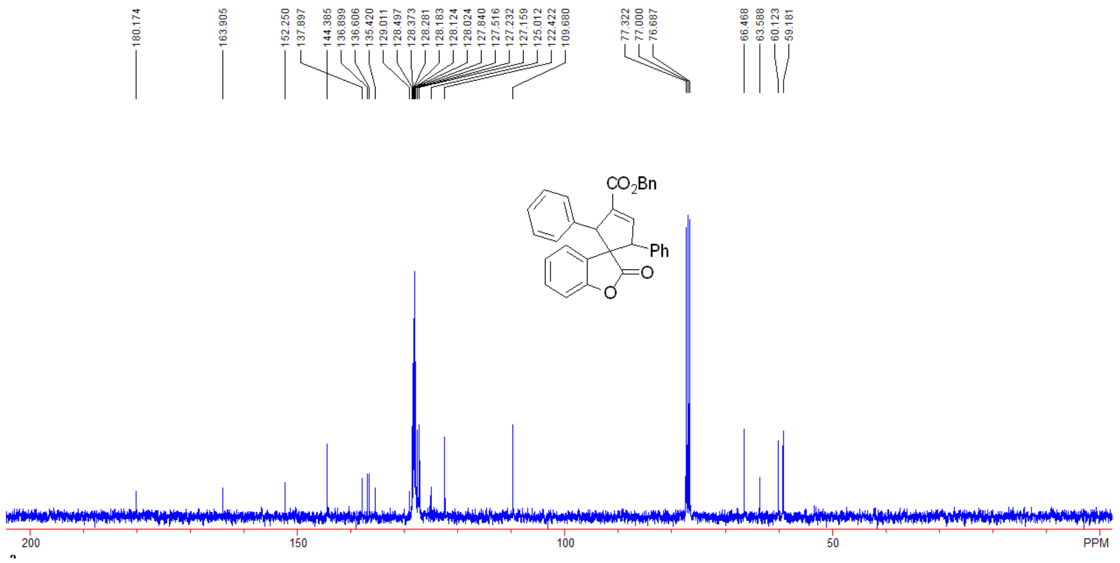

Benzyl 2-oxo-2',5'-diphenyl-2H-spiro[benzofuran-3,1'-cyclopent[3]ene]-3'-carboxylate (5i)

A pale yellow solid, 86% yield, 40 mg, Mp: 56-57 ºC. ^{1}H NMR (400 MHz, $CDCl_3$, TMS) δ 7.38 (t, J = 2.0 Hz, 1H), 7.27-7.22 (m, 3H), 7.18-7.16 (m, 1H), 7.13-7.07 (m, 3H), 7.04-7.02 (m, 4H), 6.99-6.97 (m, 2H), 6.91-6.83 (m, 3H), 6.72 (d, J = 8.0 Hz, 1H), 6.45 (dt, J_1 = 1.2 Hz, J_2 = 7.6 Hz, 1H), 6.14 (dd, J_1 = 0.8 Hz, J_2 = 7.6 Hz, 1H), 5.23 (d, J = 12.4 Hz, 1H), 5.06 (d, J = 12.4 Hz, 1H), 4.99 (t, J = 2.4 Hz, 1H), 4.83 (t, J = 2.4 Hz, 1H); ^{13}C NMR (100 MHz, $CDCl_3$) δ 180.2, 163.9, 152.3, 144.4, 137.9, 136.9, 136.6, 135.4, 129.0, 128.5, 128.4, 128.3, 128.2, 128.1, 128.0, 127.8, 127.5, 127.23, 127.16, 125.0, 122.4, 109.7, 66.5, 63.6, 60.1, 59.2; IR (neat) ν 3005, 1796, 1717, 1462, 1275, 1260, 1229, 1075, 750, 697 cm^{-1}; HRMS Calcd. for $C_{32}H_{28}NO_4^{+1}$ $(M+NH_4)^+$: 490.2013, found: 490.2003. $[\alpha]^{20}_D$ = +10.4 (c 2.0, $CHCl_3$) for 99% ee; Enantiomeric excess was determined by HPLC with a Chiralcel AD-H column, Hexane/^{i}PrOH = 80/20, 0.5 mL/min, 214 nm, t_{minor} = 23.032 min, t_{major} = 16.527 min.

N2000 数据工作站 1

实验时间：2013-07-04, 22:29:56
谱图文件:I:\regio-and enantio\液相\wd-19-6-rac-AD80200.5214.org

实验者:
报告时间：2013-10-24, 21:35:08
积分方法:面积归一法

使用仪器类型:气相色谱　　检测器:FID　　进样器:分流

柱温:程序升温

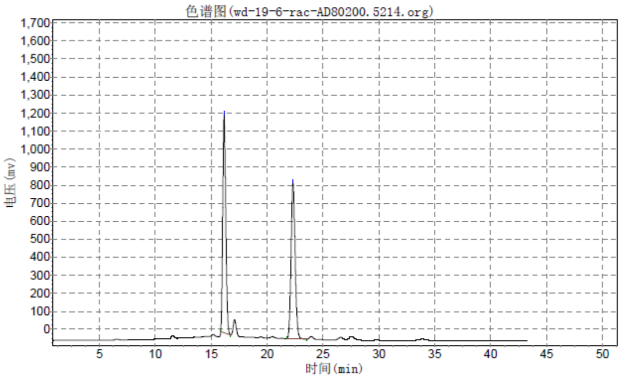

分析结果表

峰号	峰名	保留时间	峰高	峰面积	含量
1		16.140	1208379.375	22688062.000	50.6370
2		22.302	866444.625	22117218.000	49.3630
总计			2074824.000	44805280.000	100.0000

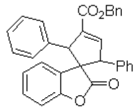

AD-H, Hexane:iPrOH = 80:20, 0.5 ml/min, 214 nm

N2000 数据工作站 1

实验时间：2013-07-05, 00:41:46
谱图文件:I:\regio-and enantio\液相\wd-19-6-asy-re-AD80200.5214.org

实验者:
报告时间：2013-10-24, 21:37:07
积分方法:面积归一法

使用仪器类型:气相色谱　　检测器:FID　　进样器:分流

柱温:程序升温

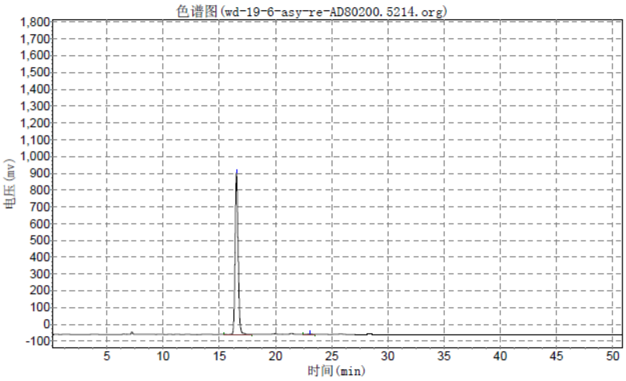

分析结果表

峰号	峰名	保留时间	峰高	峰面积	含量
1		16.527	962201.375	17861908.000	99.2975
2		23.032	4010.189	126360.188	0.7025
总计			966211.564	17988268.188	100.0000

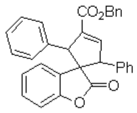

AD-H, Hexane:iPrOH = 80:20, 0.5 ml/min, 214 nm

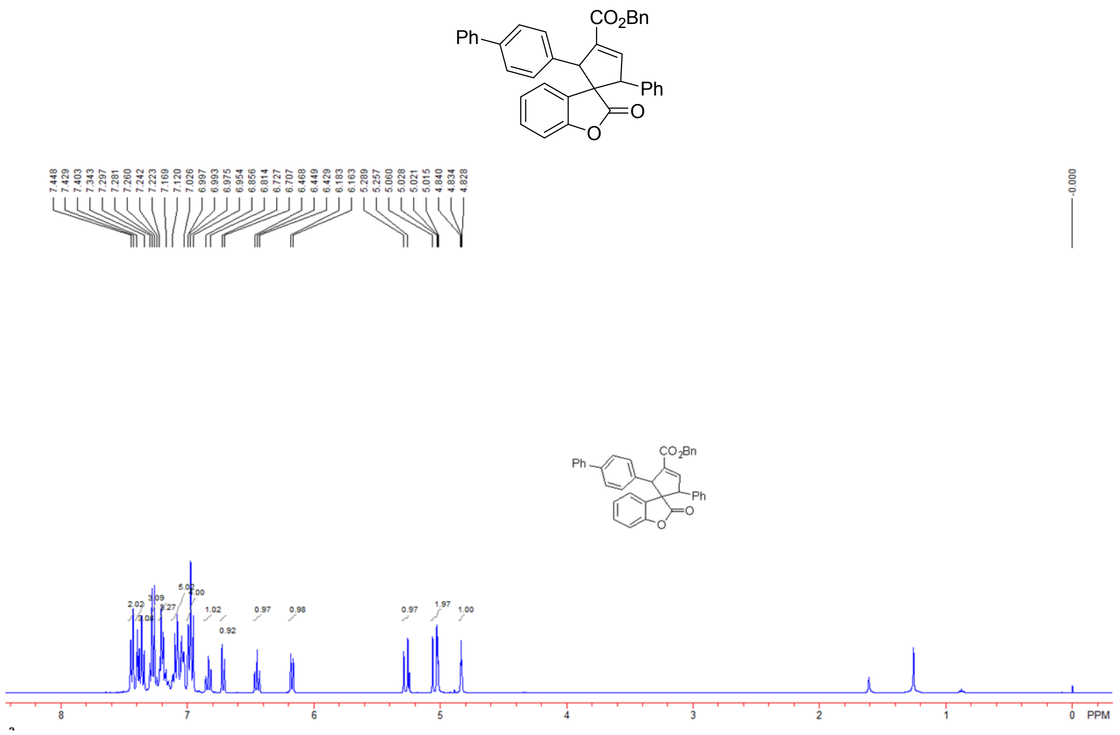

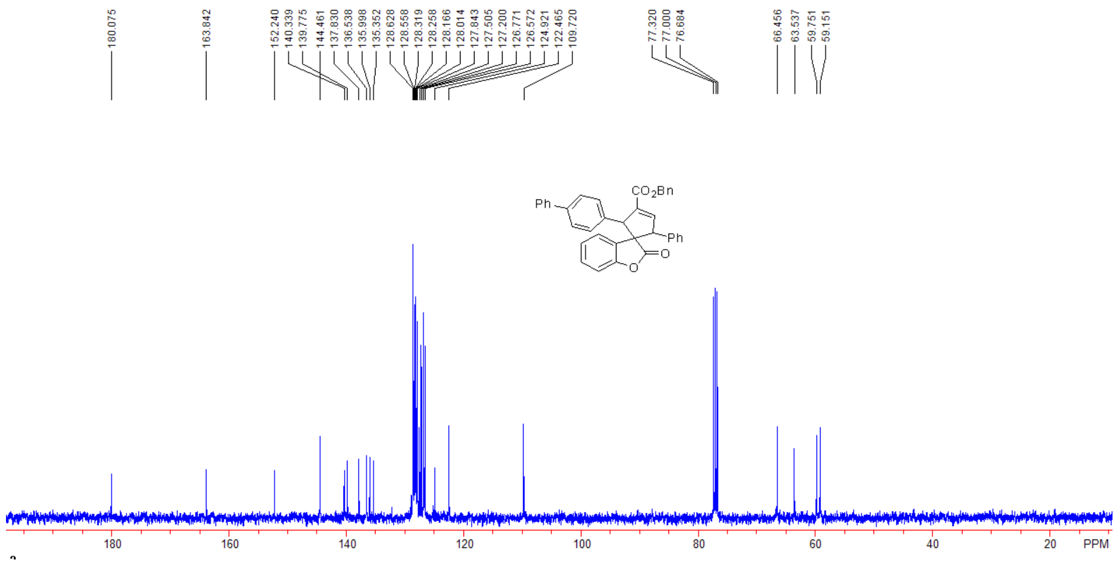

Benzyl 2'-([1,1'-biphenyl]-4-yl)-2-oxo-5'-phenyl-2H-spiro[benzofuran-3,1'-cyclopent[3]ene]-3'-carboxylate (5j)

A colorless solid, 83% yield, 45 mg, Mp: 152-153 ºC. ^{1}H NMR (400 MHz, $CDCl_3$, TMS) δ 7.45-7.43 (m, 2H), 7.40-7.34 (m, 3H), 7.30-7.24 (m, 3H), 7.22-7.17 (m, 3H), 7.12-7.03 (m, 5H), 6.99-6.95 (m, 4H), 6.86-6.81 (m, 1H), 6.72 (d, J = 8.0 Hz, 1H), 6.45 (t, J = 8.0 Hz, 2H), 6.17 (d, J = 8.0 Hz, 1H), 5.27 (d, J = 12.8 Hz, 1H), 5.04 (d, J = 12.8 Hz, 1H), 5.02 (t, J = 2.4 Hz, 1H), 4.83 (t, J = 2.4 Hz, 1H); ^{13}C NMR (100 MHz, $CDCl_3$) δ 180.1, 163.8, 152.2, 144.5, 140.3, 139.8, 137.8, 136.5, 136.0, 135.4, 128.63, 128.56, 128.32, 128.26, 128.2, 128.0, 127.8, 127.5, 127.2, 126.8, 126.6, 124.9, 122.5, 109.7, 66.5, 63.5, 59.8, 59.2; IR (neat) ν 3005, 1796, 1719, 1462, 1275, 1260,

1074, 750, 697 cm^{-1}; HRMS Calcd. for $C_{38}H_{32}NO_4^{+1}$ $(M+NH_4)^+$: 566.2326, found: 566.2317. $[\alpha]^{20}_D$ = -207.4 (c 0.4, $CHCl_3$) for 99% ee; Enantiomeric excess was determined by HPLC with a Chiralcel AD-H column, Hexane/^{i}PrOH = 80/20, 0.6 mL/min, 214 nm, t_{minor} = 21.012 min, t_{major} = 16.547 min.

N2000 数据工作站 1

实验时间：2013-06-28,10:39:07
谱图文件:J:\regio-and enantio\液相\wd-18-74-rac-AD80200.6214.org

实验者：
报告时间：2013-11-04,21:36:55
积分方法:面积归一法

使用仪器类型:气相色谱 检测器:FID 进样器:分流

柱温:程序升温

色谱图(wd-18-74-rac-AD80200.6214.org)

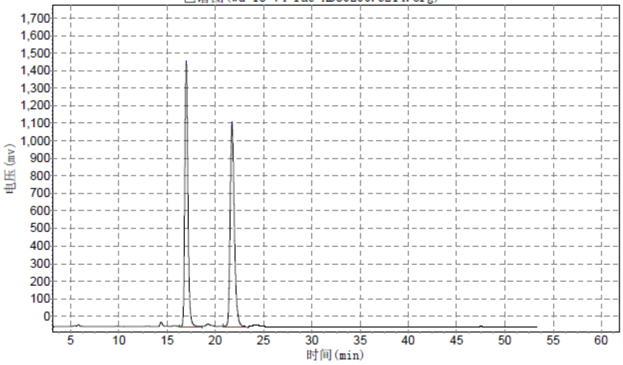

分析结果表

峰号	峰名	保留时间	峰高	峰面积	含量
1		16.990	1494858.375	33909452.000	49.2585
2		21.757	1159717.125	34930396.000	50.7415
总计			2654575.500	68839848.000	100.0000

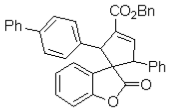

AD-H, Hexane:^{i}PrOH = 80:20, 0.6 ml/min, 214 nm

实验时间：2013-06-28, 16:58:59
谱图文件：J:\regio-and enantio\液相\wd-18-74-asy-AD80200.6214.org
实验者：
报告时间：2013-11-04, 21:41:17
积分方法：面积归一法

使用仪器类型：气相色谱 检测器：FID 进样器：分流

柱温：程序升温

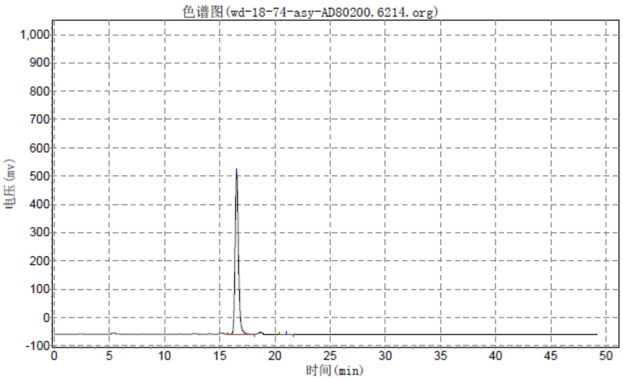

分析结果表

峰号	峰名	保留时间	峰高	峰面积	含量
1		16.547	572555.750	12091761.000	99.8803
2		21.012	544.124	14496.749	0.1197
总计			573099.874	12106257.749	100.0000

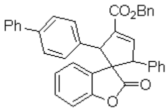

AD-H, Hexane:^{i}PrOH = 80:20, 0.6 ml/min, 214 nm

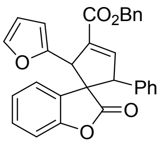

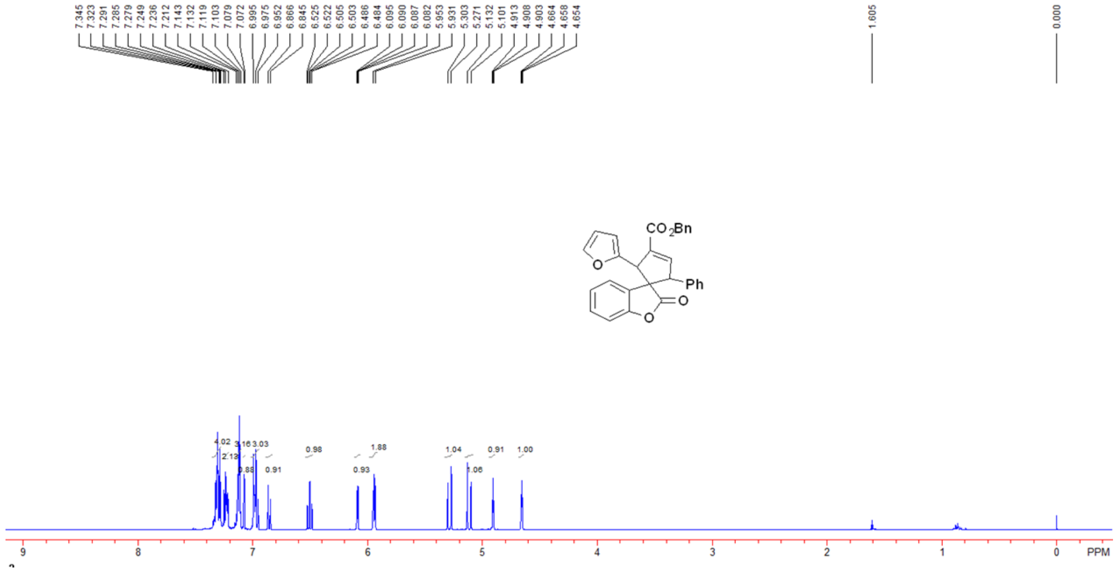

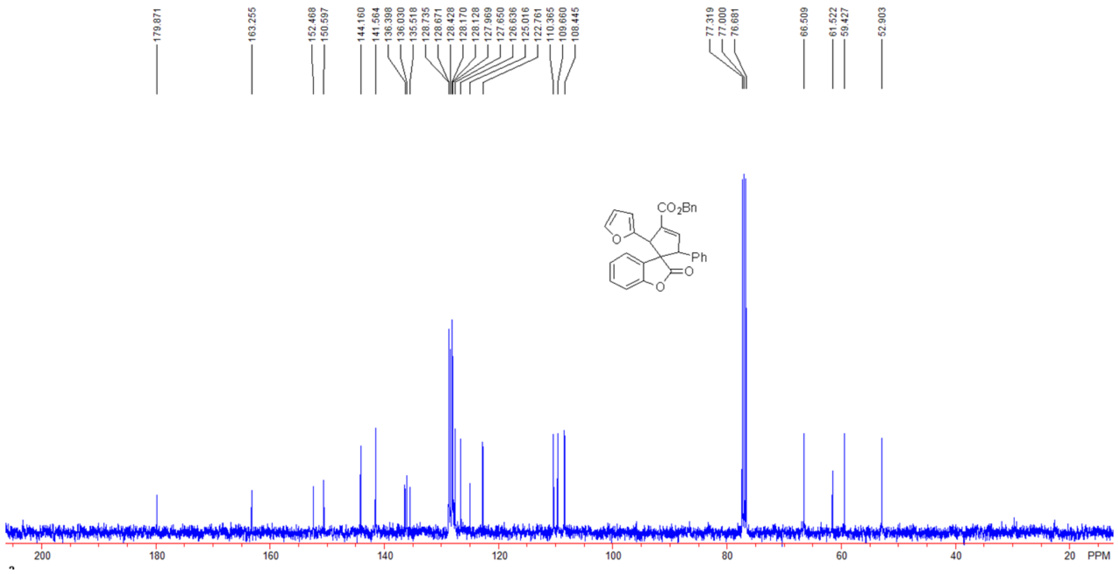

Benzyl 2'-(furan-2-yl)-2-oxo-5'-phenyl-2H-spiro[benzofuran-3,1'-cyclopent[3]ene]-3'-carboxylate (5k)

A pale yellow oil, 77% yield, 35 mg. ^{1}H NMR (400 MHz, $CDCl_3$, TMS) δ 7.35-7.28 (m, 4H), 7.25-7.21 (m, 2H), 7.14-7.10 (m, 3H), 7.08-7.07 (m, 1H), 7.00-6.95 (m, 3H), 6.87-6.85 (m, 1H), 6.50 (dt, J_1 = 0.8 Hz, J_2 = 7.6 Hz, 1H), 6.09 (dd, J_1 = 2.0 Hz, J_2 = 3.2 Hz, 1H), 5.95-5.93 (m, 2H), 5.29 (d, J = 12.4 Hz, 1H), 5.12 (d, J = 12.4 Hz, 1H), 4.91 (t, J = 2.0 Hz, 1H), 4.66 (t, J = 2.0 Hz, 1H); ^{13}C NMR (100 MHz, $CDCl_3$) δ 179.9, 163.3, 152.5, 150.6, 144.2, 141.6, 136.4, 136.0, 135.5, 128.74, 128.67, 128.4, 128.2, 128.1, 128.0, 127.7, 126.6, 125.0, 122.8, 110.4, 109.7, 108.4, 66.5, 61.5, 59.4, 52.9; IR (neat) ν 2922, 1798, 1720, 1462, 1275, 1260, 1132, 764, 698 cm^{-1}; HRMS Calcd. for $C_{30}H_{26}NO_5^{+1}$ $(M+NH_4)^+$: 480.1805, found: 480.1797. $[\alpha]^{20}_D$ = +66.1 (c 1.2, $CHCl_3$) for >99% ee; Enantiomeric excess was determined by HPLC with a Chiralcel AD-H column, Hexane/^{i}PrOH = 90/10, 0.6 mL/min, 214 nm, t_{minor} = 33.963 min, t_{major} = 20.870 min.

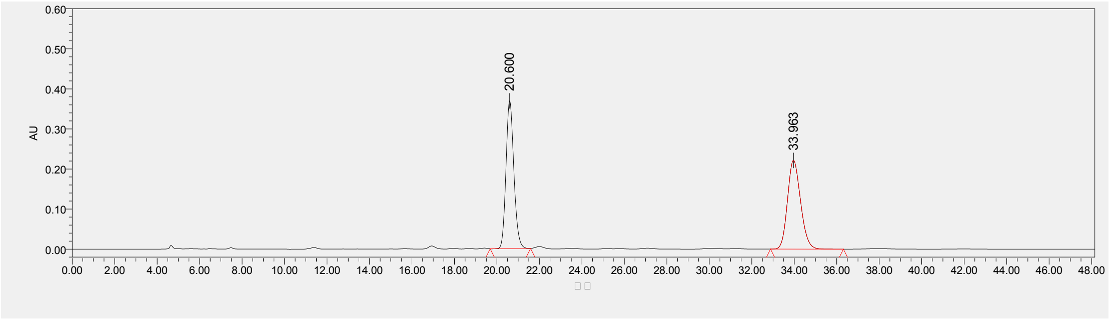

NO	R. Time	Peak Area	Precent	Peak Height
1	20.600	9490410	50.08	368957
2	33.963	9459260	49.92	221471

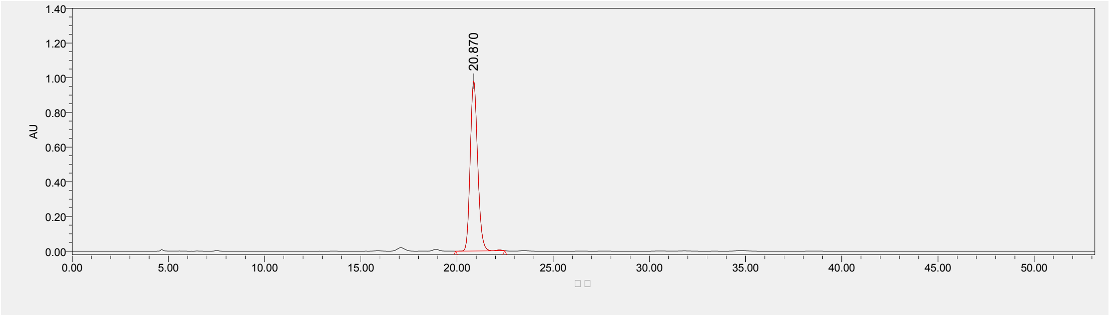

NO	R. Time	Peak Area	Precent	Peak Height
1	20.870	26304210	100.00	979588

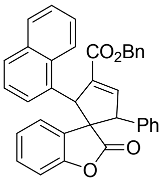

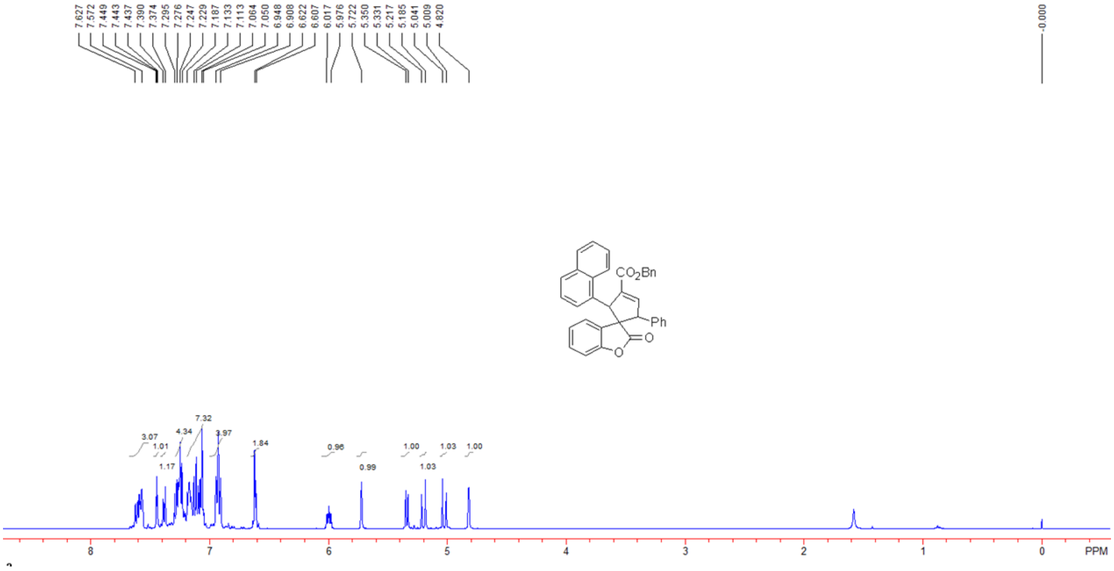

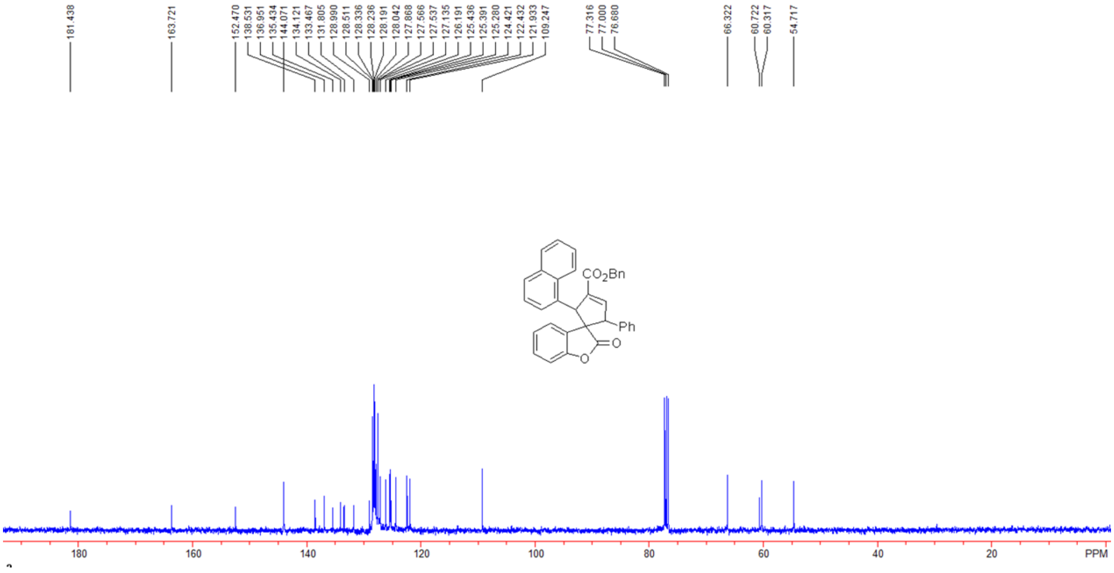

Benzyl 2'-(naphthalen-1-yl)-2-oxo-5'-phenyl-2H-spiro[benzofuran-3,1'-cyclopent[3]ene]-3'-carboxylate (5l)

A pale yellow solid, 73% yield, 38 mg, Mp: 129-130 ºC. ^{1}H NMR (400 MHz, $CDCl_3$, TMS) δ 7.63-7.57 (m, 3H), 7.44 (t, J = 2.4 Hz, 1H), 7.38 (d, J = 6.4 Hz, 1H), 7.30-7.23 (m, 4H), 7.19-7.05 (m, 7H), 6.95-6.91 (m, 4H), 6.62-6.61 (m, 2H), 6.02-5.98 (m, 1H), 5.72 (s, 1H), 5.34 (d, J = 7.6 Hz, 1H), 5.20 (d, J = 12.8 Hz, 1H), 5.03 (d, J = 12.8 Hz, 1H), 4.82 (s, 1H); ^{13}C NMR (100 MHz, $CDCl_3$) δ 181.4, 163.7, 152.5, 144.1, 138.5, 137.0, 135.4, 134.1, 133.5, 131.8, 129.0, 128.5, 128.3, 128.24, 128.19, 128.0, 127.9, 127.6, 127.5, 127.1, 126.2, 125.44, 125.39, 125.3, 124.4, 122.4, 121.9, 109.2, 66.3, 60.7, 60.3, 54.7; IR (neat) ν 2924, 1793, 1719, 1617, 1462, 1229, 1131, 1076, 965, 752 cm^{-1}; HRMS Calcd. for $C_{36}H_{30}NO_4^{+1}$ $(M+NH_4)^+$: 540.2169, found: 540.2158. $[\alpha]^{20}_D$ = +91.7 (c 2.5, $CHCl_3$) for 90% ee; Enantiomeric excess was determined by HPLC with a Chiralcel AD-H column, Hexane/^{i}PrOH = 90/10, 0.65 mL/min, 214 nm, t_{minor} = 17.476 min, t_{major} = 21.164 min.

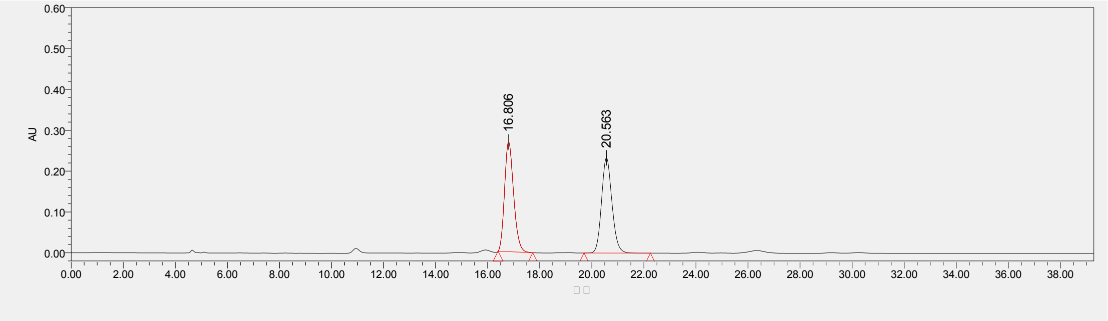

NO	R. Time	Peak Area	Precent	Peak Height
1	16.806	6503290	50.08	268956
2	20.563	6482948	49.92	233296

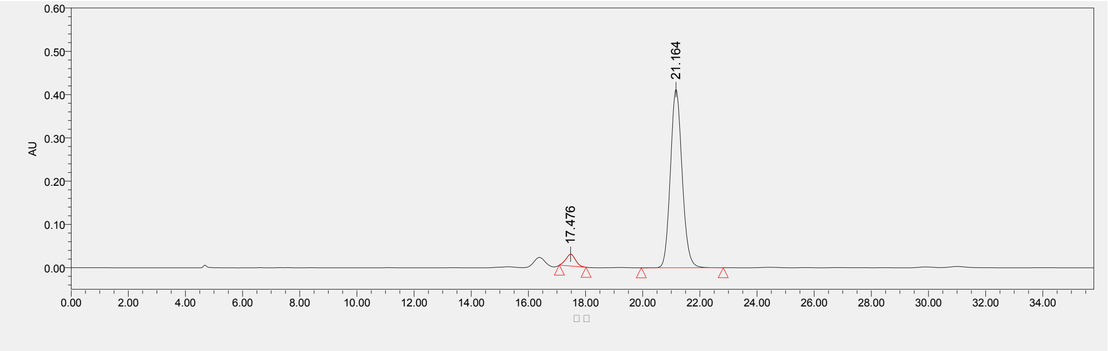

NO	R. Time	Peak Area	Precent	Peak Height
1	17.476	636139	5.14	27175
2	21.164	11734074	94.86	411896

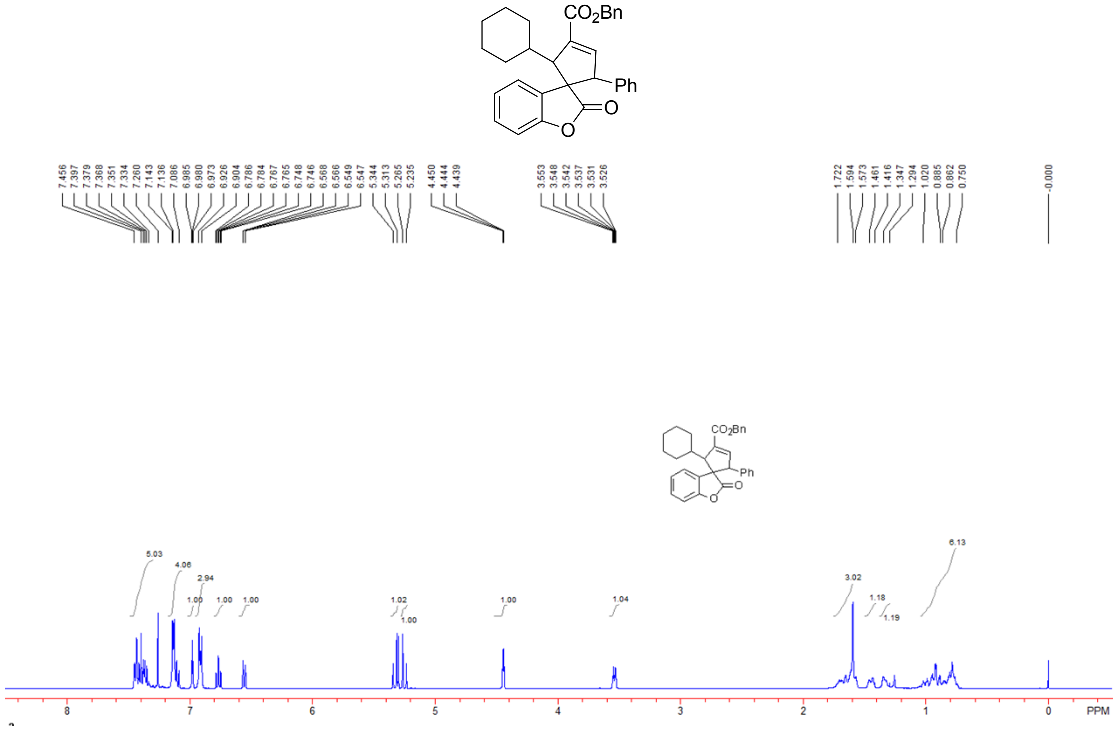

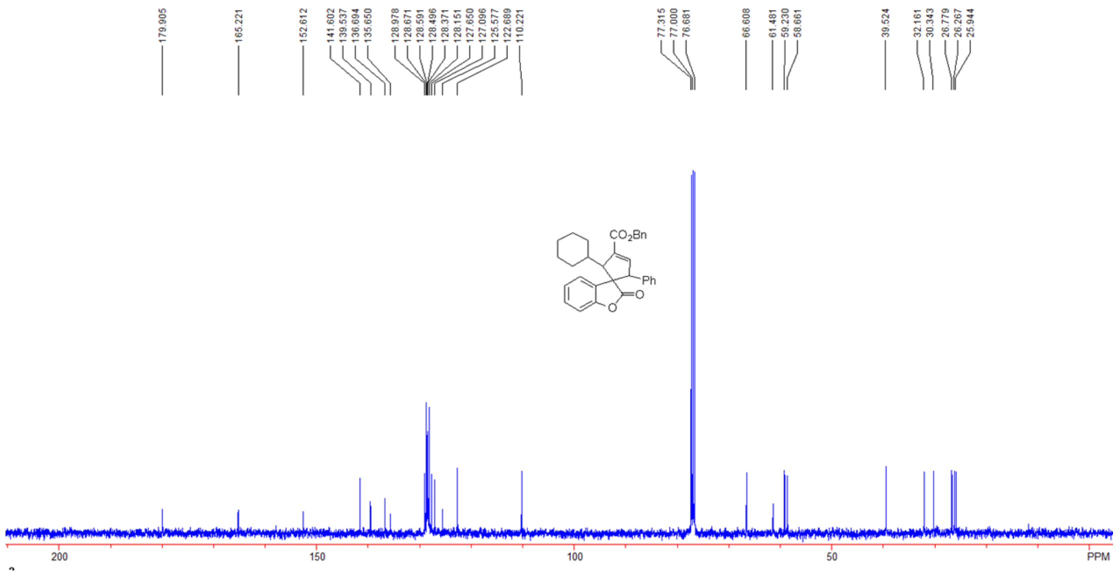

Benzyl 2'-cyclohexyl-2-oxo-5'-phenyl-2H-spiro[benzofuran-3,1'-cyclopent[3]ene]-3'-carboxylate (5m)

A pale yellow oil, 92% yield, 43 mg. ^{1}H NMR (400 MHz, $CDCl_3$, TMS) δ 7.46-7.33 (m, 5H), 7.14-7.09 (m, 4H), 6.98 (t, J = 2.4 Hz, 1H), 6.93-6.90 (m, 3H), 6.77 (dt, J_1 = 0.8 Hz, J_2 = 7.6 Hz, 1H), 6.56 (d, J_1 = 0.8 Hz, J_2 = 7.6 Hz, 1H), 5.33 (d, J = 12.4 Hz, 1H), 5.25 (d, J = 12.4 Hz, 1H), 4.44 (t, J = 2.0 Hz, 1H), 3.54 (dt, J_1 = 2.0 Hz, J_2 = 6.4 Hz, 1H), 1.72-1.57 (m, 3H), 1.46-1.42 (m, 1H), 1.35-1.29 (m, 1H), 1.02-0.75 (m, 6H); ^{13}C NMR (100 MHz, $CDCl_3$) δ 179.9, 165.2, 152.6, 141.6, 139.5, 136.7, 135.7, 129.0, 128.7, 128.6, 128.5, 128.4, 128.2, 127.7, 127.1, 125.6, 122.7, 110.2, 66.6, 61.5, 59.2, 58.7, 39.5, 32.2, 30.3, 26.8, 26.3, 25.9; IR (neat) ν 2924, 1798, 1716,

1614, 1461, 1231, 1090, 1018, 872, 753, 697 cm^{-1}; HRMS Calcd. for $C_{32}H_{34}NO_4^{+1}$ $(M+NH_4)^+$: 496.2482, found: 496.2481. $[\alpha]^{20}_D$ = +80.7 (c 1.8, $CHCl_3$) for >99% ee; Enantiomeric excess was determined by HPLC with a Chiralcel AD-H column, Hexane/^{i}PrOH = 80/20, 0.5 mL/min, 214 nm, t_{minor} = 23.432 min, t_{major} = 27.015 min.

N2000 数据工作站 1

实验时间：2013-09-13, 10:37:09
谱图文件:I:\regio-and enantio\液相\wd-19-77-race-PC2-955-0.6-214.org

实验者：
报告时间：2013-10-22, 21:04:12
积分方法:面积归一法

使用仪器类型:气相色谱 检测器:FID 进样器:分流

柱温:程序升温

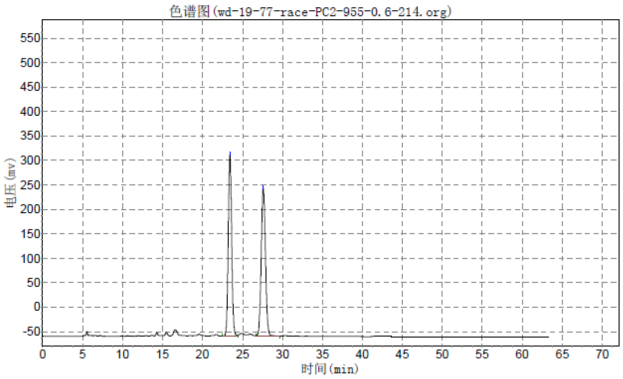

分析结果表

峰号	峰名	保留时间	峰高	峰面积	含量
1		23.432	370534.219	11261866.000	50.0140
2		27.598	302308.250	11255574.000	49.9860
总计			672842.469	22517440.000	100.0000

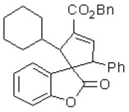

PC-2, *n*-Hexane:^{i}PrOH = 95:5, 0.6 ml/min, 214 nm

实验时间：2013-09-13, 11:40:41
谱图文件:I:\regio-and enantio\液相\wd-19-77-asy-PC2-955-0.6-214.org

实验者：
报告时间：2013-10-22, 21:08:56
积分方法:面积归一法

使用仪器类型:气相色谱 检测器:FID 进样器:分流

柱温:程序升温

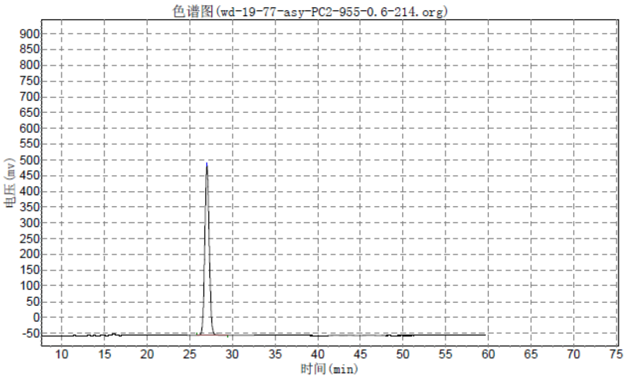

分析结果表

峰号	峰名	保留时间	峰高	峰面积	含量
1		27.015	538275.313	19452646.000	100.0000
总计			538275.313	19452646.000	100.0000

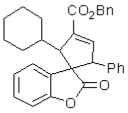

PC-2, *n*-Hexane:^{i}PrOH = 95:5, 0.6 ml/min, 214 nm

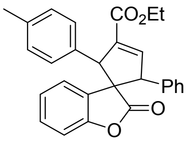

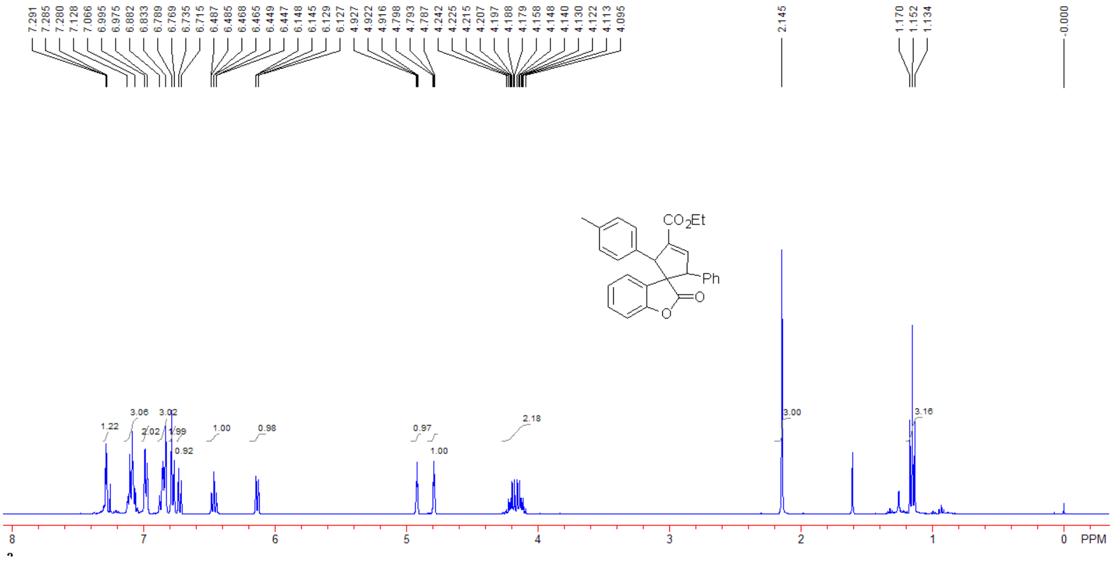

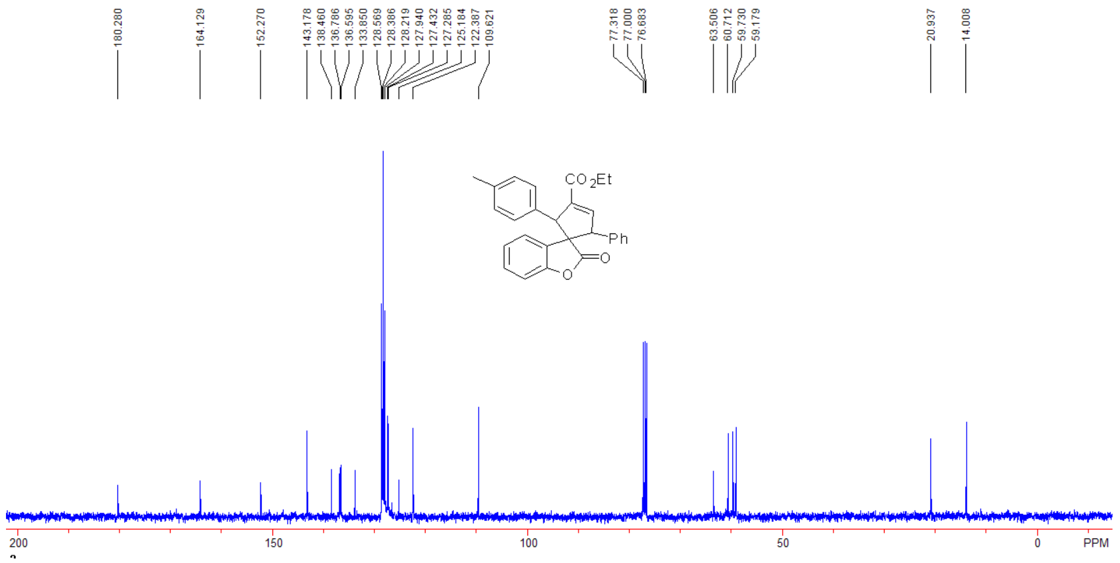

Ethyl 2-oxo-5'-phenyl-2'-(p-tolyl)-2H-spiro[benzofuran-3,1'-cyclopent[3]ene]-3'-carboxylate (5n)

A pale yellow solid, 67% yield, 28 mg, Mp: 78-80 ºC. ^{1}H NMR (400 MHz, $CDCl_3$, TMS) δ 7.29-7.28 (m, 1H), 7.13-7.07 (m, 3H), 7.00-6.98 (m, 2H), 6.88-6.83 (m, 3H), 6.78 (d, J = 8.0 Hz, 2H), 6.73 (d, J = 8.0 Hz, 1H), 6.47 (dt, J_1 = 0.8 Hz, J_2 = 7.2 Hz, 1H), 6.14 (dd, J_1 = 0.8 Hz, J_2 = 7.2 Hz, 1H), 4.92 (t, J = 2.0 Hz, 1H), 4.79 (t, J = 2.0 Hz, 1H), 4.24-4.10 (m, 2H), 2.15 (s, 3H), 1.15 (t, J = 7.2 Hz, 3H); ^{13}C NMR (100 MHz, $CDCl_3$) δ 180.3, 164.1, 152.3, 143.2, 138.5, 136.8, 136.6, 133.9, 128.6, 128.4, 128.2, 127.9, 127.4, 127.3, 125.2, 122.4, 109.6, 63.5, 60.7, 59.7, 59.2, 20.9, 14.0; IR (neat) ν 2987, 1797, 1717, 1462, 1275, 1260, 1074, 750 cm^{-1}; HRMS Calcd. for $C_{28}H_{28}NO_4^{+1}$ $(M+NH_4)^+$: 442.2013, found: 442.2022. $[\alpha]^{20}_D$ = -33.9 (c 0.85, $CHCl_3$) for 90% ee; Enantiomeric excess was determined by HPLC with a Chiralcel PC-2 column, Hexane/^{i}PrOH = 90/10, 0.5 mL/min, 214 nm, t_{minor} = 27.947 min, t_{major} = 24.077 min.

实验时间：2013-08-30, 17:12:30
谱图文件:I:\regio-and enantio\液相\wd-18-63-rac-PC-2-9010-0.5-214.org

实验者：
报告时间：2013-10-22, 21:14:37
积分方法:面积归一法

使用仪器类型:气相色谱　　检测器:FID　　进样器:分流

柱温:程序升温

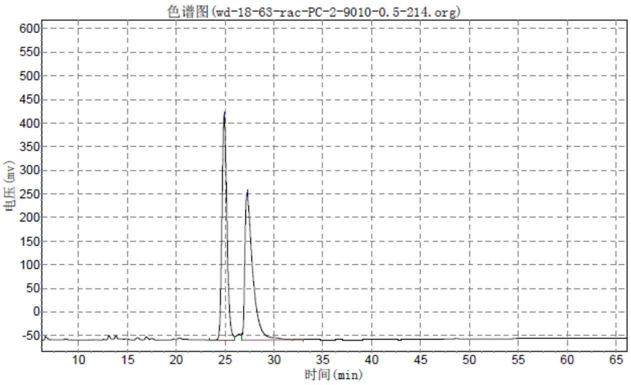

分析结果表

峰号	峰名	保留时间	峰高	峰面积	含量
1		24.907	477019.500	16915916.000	49.1172
2		27.240	311463.469	17524020.000	50.8828
总计			788482.969	34439936.000	100.0000

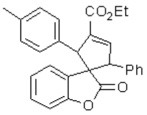

PC-2, *n*-Hexane:^{i}PrOH = 90:10, 0.5 ml/min, 214 nm

N2000 数据工作站 1

实验时间：2013-08-30, 18:37:57
谱图文件:I:\regio-and enantio\液相\wd-18-63-asy-PC-2-9010-0.5-214.org

实验者：
报告时间：2013-10-22, 21:16:12
积分方法:面积归一法

使用仪器类型:气相色谱　　检测器:FID　　进样器:分流

柱温:程序升温

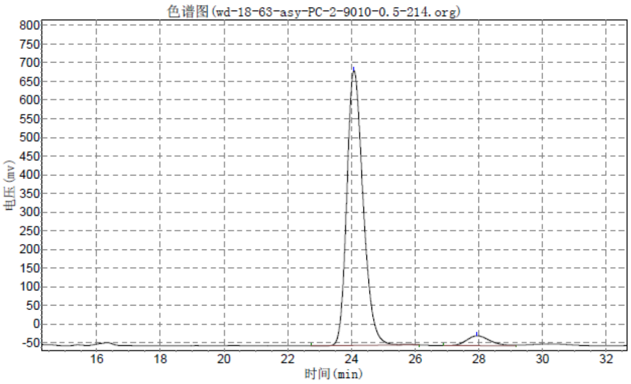

分析结果表

峰号	峰名	保留时间	峰高	峰面积	含量
1		24.077	735976.438	26178900.000	95.0656
2		27.947	26538.094	1358817.625	4.9344
总计			762514.531	27537717.625	100.0000

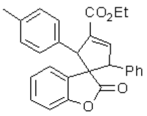

PC-2, *n*-Hexane:^{i}PrOH = 90:10, 0.5 ml/min, 214 nm

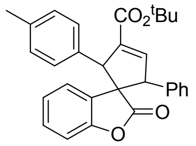

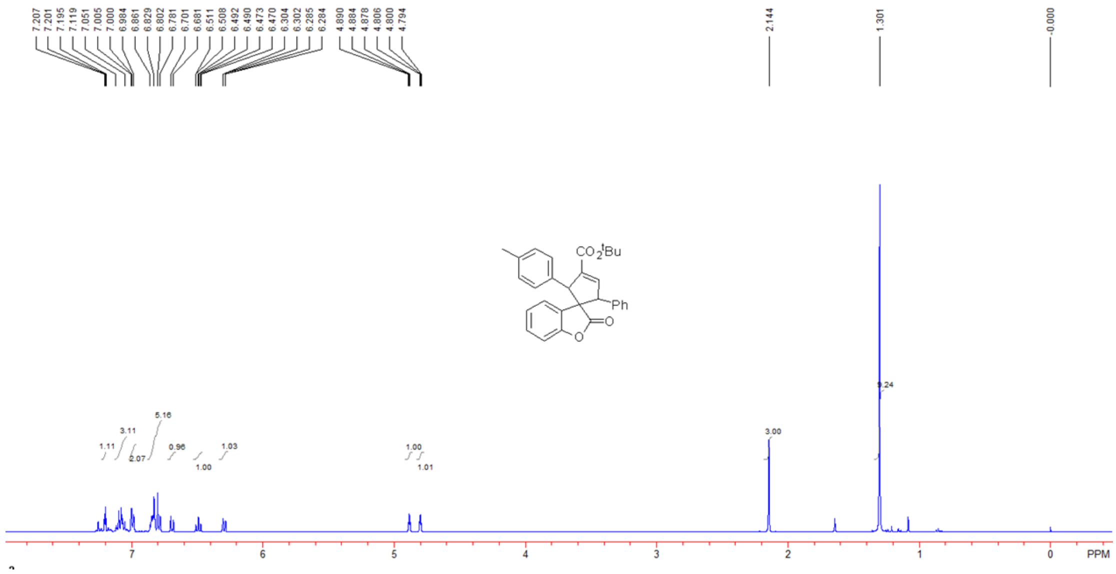

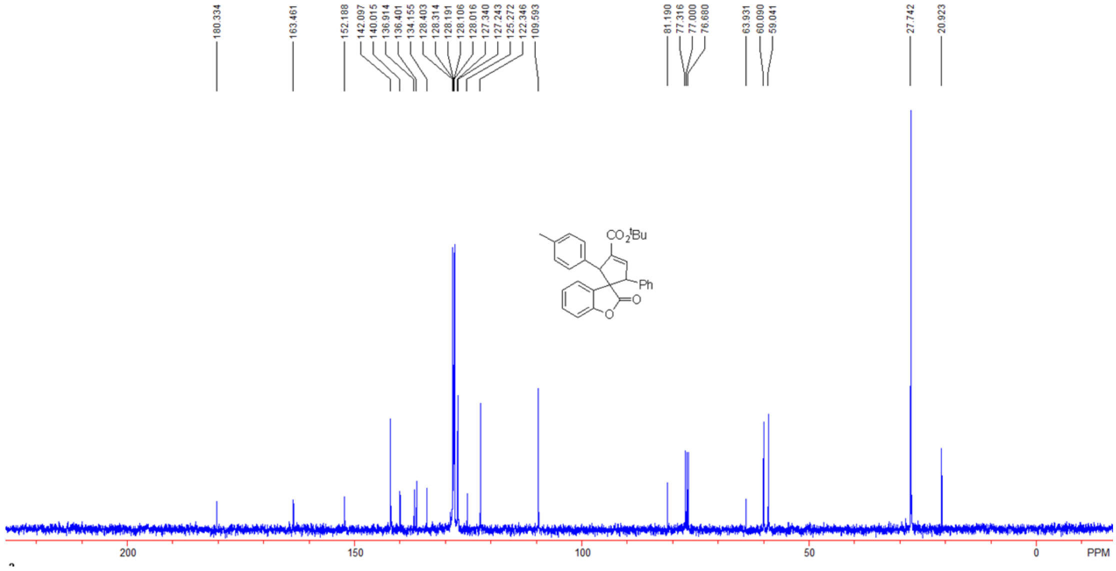

tert-Butyl 2-oxo-5'-phenyl-2'-(p-tolyl)-2H-spiro[benzofuran-3,1'-cyclopent[3]ene]-3'-carboxylate (5o)

A colorless solid, 83% yield, 37 mg, Mp: 60-62 ºC. ^{1}H NMR (400 MHz, $CDCl_3$, TMS) δ 7.20 (t, J = 2.4 Hz, 1H), 7.12-7.05 (m, 3H), 7.01-6.98 (m, 2H), 6.86-6.78 (m, 5H), 6.69 (d, J = 8.0 Hz, 1H), 6.49 (dt, J_1= 1.2 Hz, J_2= 8.0 Hz, 1H), 6.29 (dd, J_1 = 0.8 Hz, J_2 = 7.2 Hz, 1H), 4.88 (t, J = 2.4 Hz, 1H), 4.80 (t, J = 2.4 Hz, 1H), 2.14 (s, 3H), 1.30 (s, 9H); ^{13}C NMR (100 MHz, $CDCl_3$) δ 180.3, 163.5, 152.2, 142.1, 140.0, 136.9, 136.4, 134.2, 128.4, 128.3, 128.2, 128.1, 128.0, 127.3, 127.2, 125.3, 122.3, 109.6, 81.2, 63.9, 60.1, 59.0, 27.7, 20.9; IR (neat) ν 2975, 1797, 1712, 1617, 1462, 1162, 1073, 965, 750, 699 cm^{-1}; HRMS Calcd. for $C_{30}H_{32}NO_4^{+1}$ $(M+NH_4)^+$: 470.2326, found:

470.2341. $[\alpha]^{20}_{D}$ = -3.7 (c 1.3, $CHCl_3$) for 98% ee; Enantiomeric excess was determined by HPLC with a Chiralcel IC-H column, Hexane/^{i}PrOH = 90/10, 0.5 mL/min, 214 nm, t_{minor} = 13.187 min, t_{major} = 17.387 min.

N2000 数据工作站 1

实验时间：2013-08-31,12:24:57
谱图文件:I:\regio-and enantio\液相\wd-19-59-rac-IC-9010-0.5-214.org

实验者：
报告时间：2013-10-22,21:21:26
积分方法:面积归一法

使用仪器类型:气相色谱　　检测器:FID　　进样器:分流

柱温:程序升温

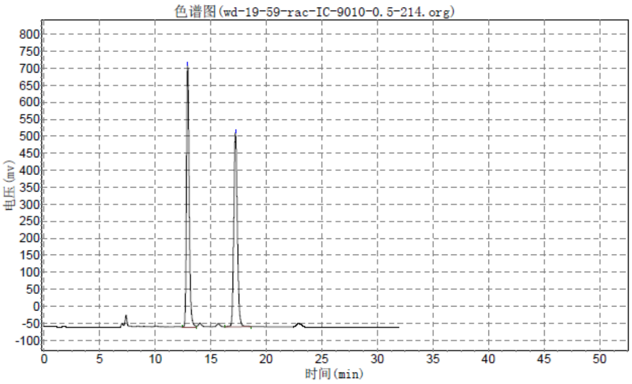

分析结果表

峰号	峰名	保留时间	峰高	峰面积	含量
1		12.928	768130.688	12880998.000	50.3778
2		17.230	570264.625	12687778.000	49.6222
总计			1338395.313	25568776.000	100.0000

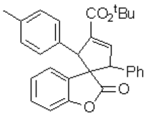

IC-H, *n*-Hexane:^{i}PrOH = 90:10, 0.5 ml/min, 214 nm

实验时间：2013-08-31，13:18:07
谱图文件:I:\regio-and enantio\液相\wd-19-59-asy-IC-9010-0.5-214.org

实验者：
报告时间：2013-10-22，21:24:54
积分方法:面积归一法

使用仪器类型:气相色谱 检测器:FID 进样器:分流

柱温:程序升温

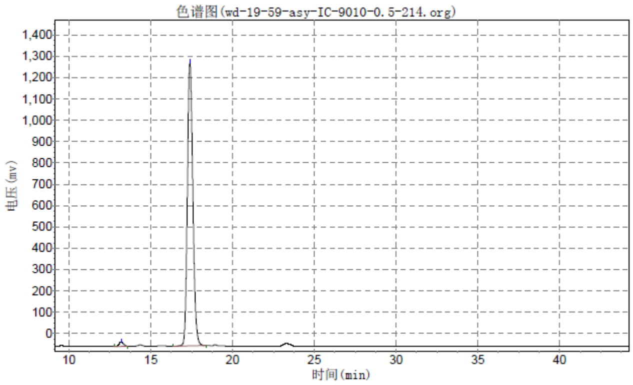

分析结果表

峰号	峰名	保留时间	峰高	峰面积	含量
1		13.187	20133.744	386423.594	1.2282
2		17.387	1329450.000	31077186.000	98.7718
总计			1349583.744	31463609.594	100.0000

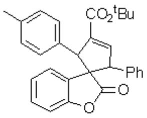

IC-H, *n*-Hexane:^{i}PrOH = 90:10, 0.5 ml/min, 214 nm

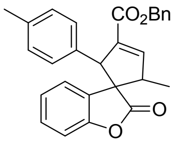

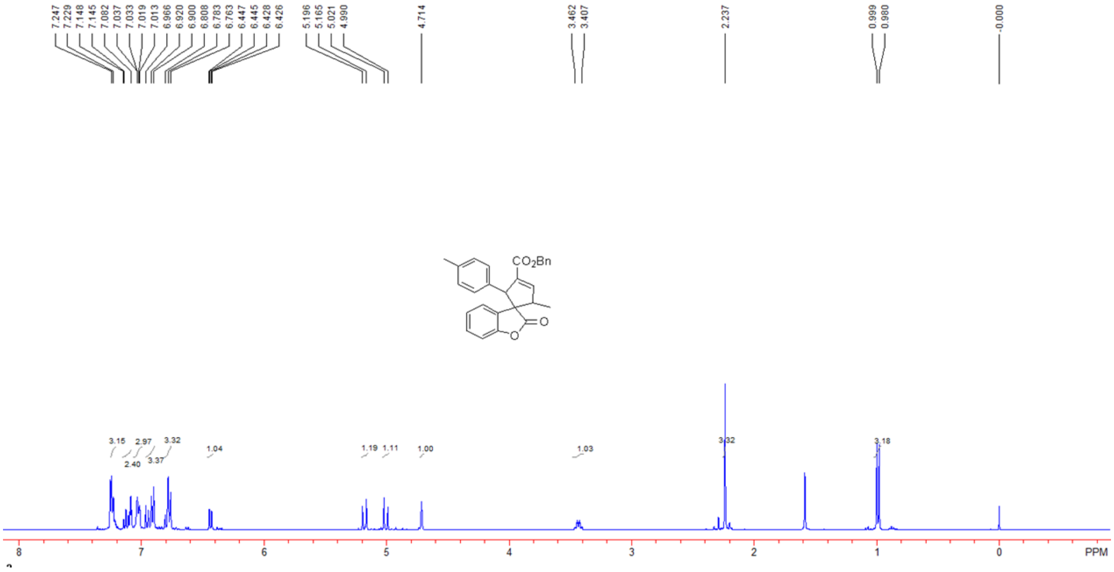

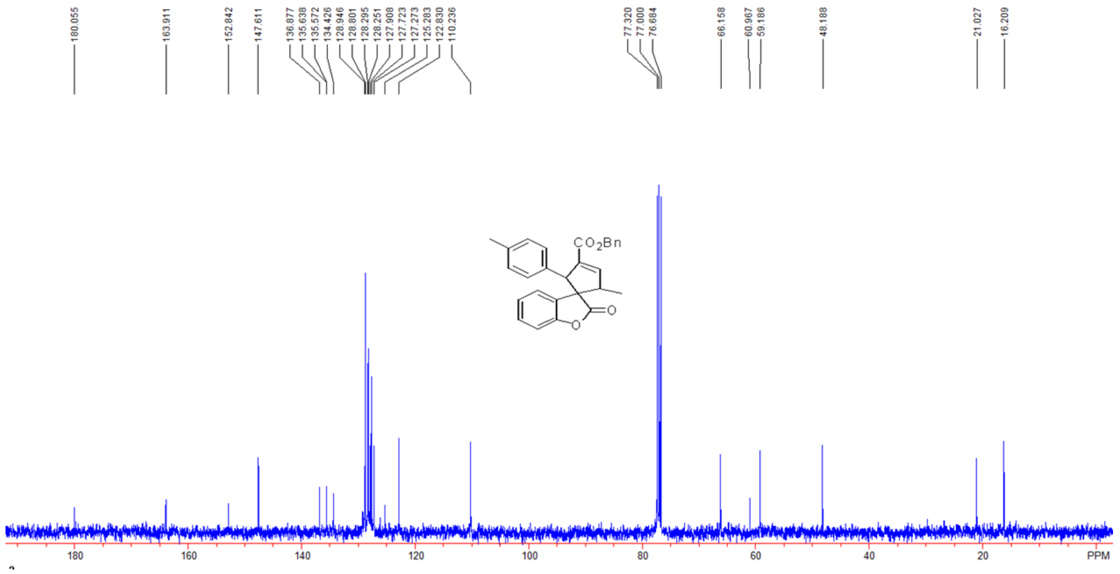

Benzyl 5'-methyl-2-oxo-2'-(p-tolyl)-2H-spiro[benzofuran-3,1'-cyclopent[3]ene]-3'-carboxylate (5p)

A coloeless oil, 62% yield, 26 mg. ^{1}H NMR (400 MHz, $CDCl_3$, TMS) δ 7.25-7.23 (m, 3H), 7.15-7.08 (m, 2H), 7.04-7.01 (m, 2H), 6.97-6.90 (m, 3H), 6.81-6.76 (m, 3H), 6.44 (dd, J_1 = 0.8 Hz, J_2 = 7.6 Hz, 1H), 5.18 (d, J = 12.4 Hz, 1H), 5.01 (d, J = 12.4 Hz, 1H), 4.71 (s, 1H), 3.46-3.41 (m, 1H), 2.24 (s, 3H), 0.99 (d, J = 7.6 Hz, 3H); ^{13}C NMR (100 MHz, $CDCl_3$) δ 180.1, 163.9, 152.8, 147.6, 136.9, 135.64, 135.57, 134.4, 128.9, 128.8, 128.3, 128.2, 127.9, 127.7, 127.3, 125.3, 122.8, 110.2, 66.2, 61.0, 59.2, 48.2, 21.0, 16.2; IR (neat) ν 2924, 1799, 1716, 1617, 1461, 1232, 1137, 1064, 751 cm^{-1}; HRMS Calcd. for $C_{28}H_{28}NO_4^{+1}$ (M^++H): 442.2013, found: 442.2025. $[\alpha]^{20}_D$ = -108.8 (c 1.0, $CHCl_3$) for 99% ee; Enantiomeric excess was determined by HPLC with a Chiralcel IC-H column, Hexane/^{i}PrOH = 90/10, 0.5 mL/min, 214 nm, t_{minor} = 35.963 min, t_{major} = 40.553 min.

N2000 数据工作站 1

实验时间：2013-08-27, 17:18:05
谱图文件:J:\regio-and enantio\液相\wd-19-49re-rac-IC-9010-0.5-214.org

实验者：
报告时间：2013-11-04, 21:28:38
积分方法:面积归一法

使用仪器类型:气相色谱　　检测器:FID　　进样器:分流

柱温:程序升温

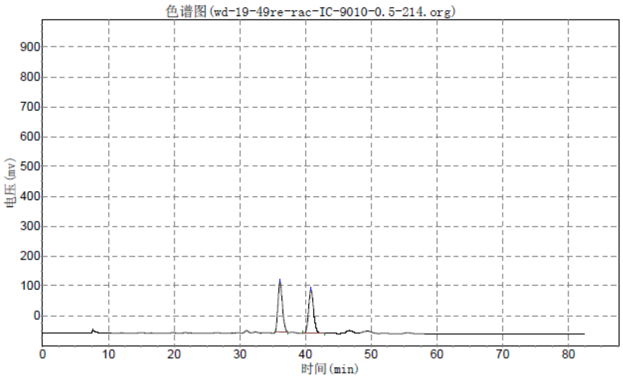

分析结果表

峰号	峰名	保留时间	峰高	峰面积	含量
1		36.097	168259.906	7848991.500	50.5319
2		40.810	145735.234	7683766.500	49.4681
总计			313995.141	15532758.000	100.0000

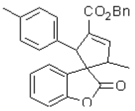

IC-H, Hexane: ^{i}PrOH = 90:10, 0.5 ml/min, 214 nm

N2000 数据工作站 1

实验时间：2013-08-27, 15:55:11
谱图文件:I:\6.regio-and enantio\液相\wd-19-49re-asy-IC-9010-0.5-214.org

实验者：
报告时间：2013-11-16, 13:02:15
积分方法:面积归一法

使用仪器类型:气相色谱　　检测器:FID　　进样器:分流

柱温:程序升温

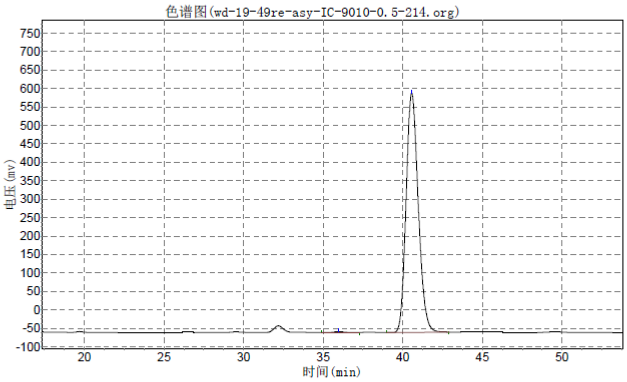

分析结果表

峰号	峰名	保留时间	峰高	峰面积	含量
1		35.963	2431.767	101378.602	0.2926
2		40.553	648808.188	34543640.000	99.7074
总计			651239.955	34645018.602	100.0000

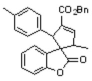

IC-H, Hexane:iPrOH = 90:10, 0.5 ml/min, 214 nm

7. Characterization and spectra charts for 7-8 and 10-11

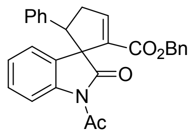

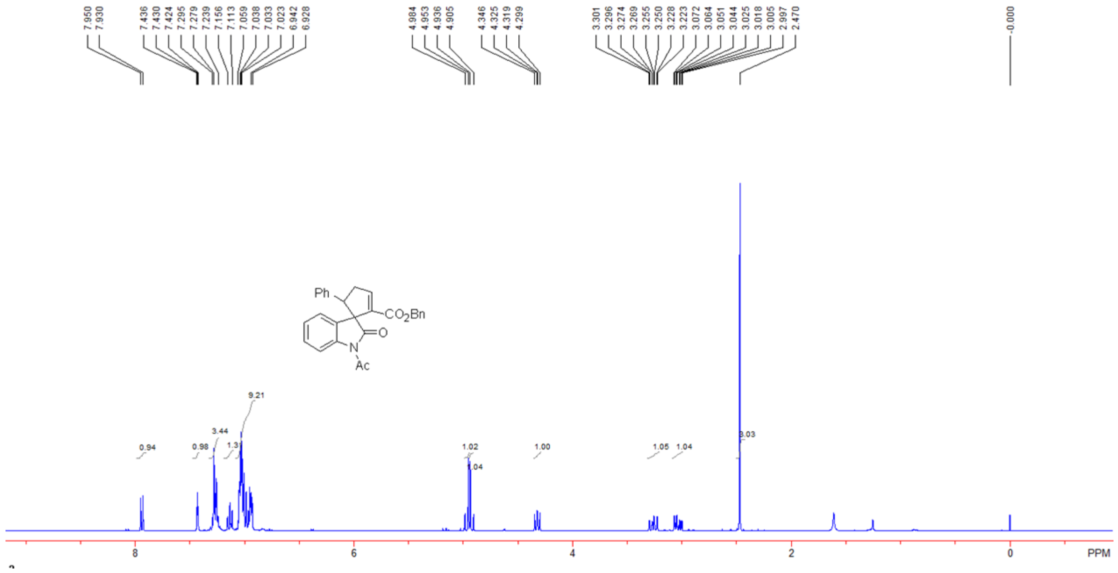

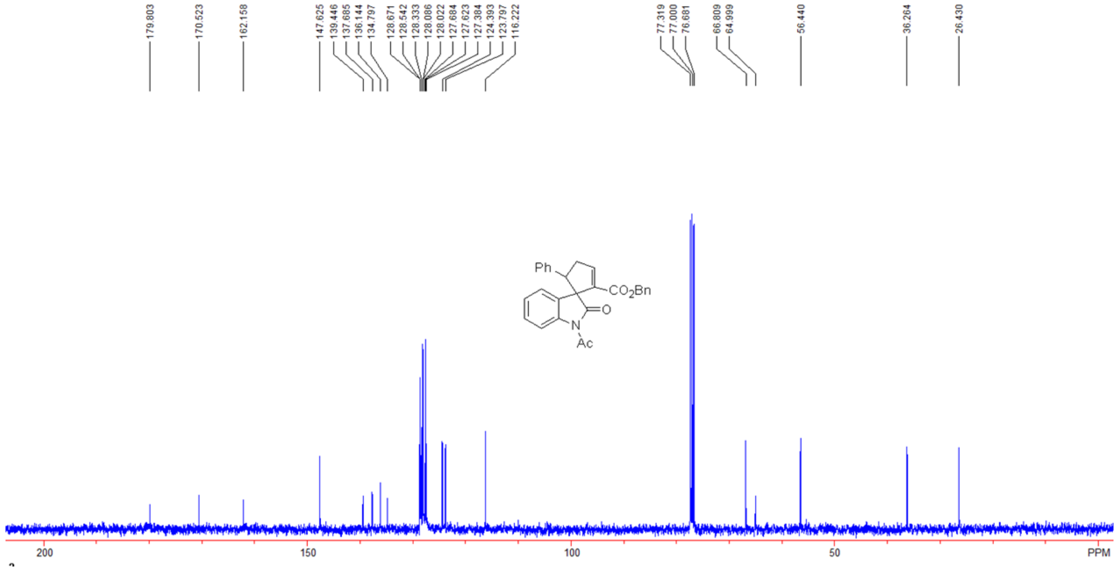

Benzyl 1'-acetyl-2'-oxo-5-phenylspiro[cyclopent[2]ene-1,3'-indoline]-2-carboxylate (7a)

A colorless oil, 78% yield, 33 mg. ^{1}H NMR (400 MHz, $CDCl_3$, TMS) δ 7.94 (d, J = 8.0 Hz, 1H), 7.43 (t, J = 2.4 Hz, 1H), 7.30-7.24 (m, 3H), 7.16-7.11 (m, 1H), 7.06-6.93 (m, 9H), 4.97 (d, J= 12.4 Hz, 1H), 4.92 (d, J = 12.4 Hz, 1H), 4.32 (dd, J_1 = 8.0 Hz, J_2 = 10.8 Hz, 1H), 3.26 (ddd, J_1 = 2.0 Hz, J_2 = 10.8 Hz, J_3 = 18.4 Hz, 1H), 3.03 (ddd, J_1 = 3.2 Hz, J_2 = 8.0 Hz, J_3 = 18.4 Hz, 1H), 2.47 (s, 3H); ^{13}C NMR (100 MHz, $CDCl_3$) δ 179.8, 170.5, 162.2, 147.6, 139.4, 137.7, 136.1, 134.8, 128.7, 128.5, 128.3, 128.1, 128.0, 127.7, 127.6, 127.4, 124.4, 123.8, 116.2, 66.8, 65.0, 56.4, 36.3, 26.4; IR (neat) ν 2919, 1747, 1712, 1604, 1463, 1370, 1270, 1170, 1093, 753, 697 cm

[1]; HRMS Calcd. for $C_{28}H_{24}NO_4^{+1}$ $(M+H)^+$: 438.1700, found: 438.1701. $[\alpha]^{20}_{D}$ = +80.3 (c 0.8, $CHCl_3$) for 96% ee; Enantiomeric excess was determined by HPLC with a Chiralcel IC-H column, Hexane/^{i}PrOH = 90/10, 0.7 mL/min, 214 nm, t_{minor} = 18.053 min, t_{major} = 43.273 min.

N2000 数据工作站 1

实验时间：2013-09-11, 11:32:56
谱图文件:I:\regio-and enantio\液相\wd-19-74-race-IC-9010-0.7-214.org
实验者：
报告时间：2013-10-26, 9:54:24
积分方法:面积归一法

使用仪器类型:气相色谱 检测器:FID 进样器:分流

柱温:程序升温

色谱图(wd-19-74-race-IC-9010-0.7-214.org)

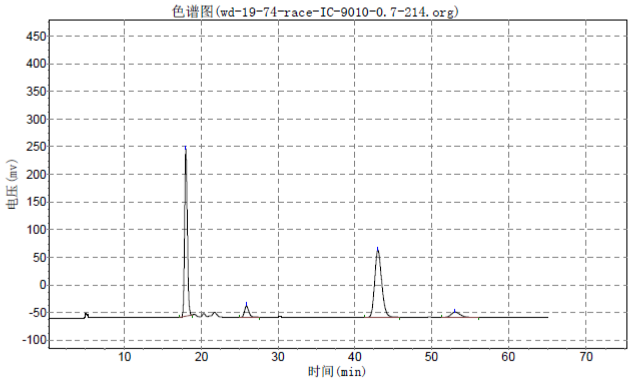

分析结果表

峰号	峰名	保留时间	峰高	峰面积	含量
1		17.992	302710.750	7500504.000	44.7326
2		25.880	20534.063	792647.250	4.7273
3		43.002	121685.414	7682238.500	45.8165
4		53.063	9585.549	792032.500	4.7236
总计			454515.775	16767422.250	100.0000

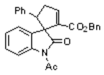

IC-H, Hexane:^{i}PrOH = 90:10, 0.7 mL/min, 214 nm

实验时间：2013-09-11, 12:55:39
谱图文件：I:\regio-and enantio\液相\wd-19-74-asy-IC-9010-0.7-214.org
实验者：
报告时间：2013-11-05, 9:10:47
积分方法：面积归一法

使用仪器类型：气相色谱　检测器：FID　进样器：分流

柱温：程序升温

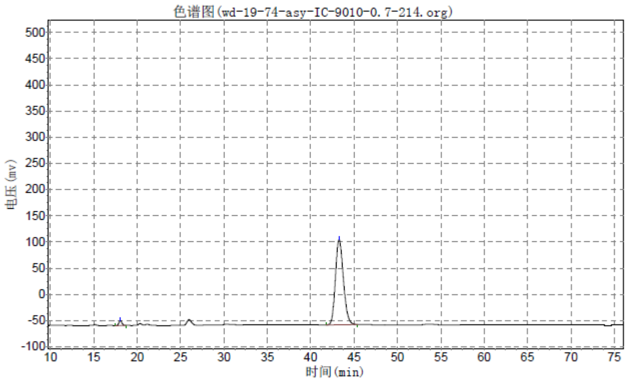

分析结果表

峰号	峰名	保留时间	峰高	峰面积	含量
1		18.053	9230.842	226504.391	2.1413
2		43.273	162445.188	10351497.000	97.8587
总计			171676.029	10578001.391	100.0000

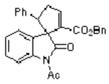

IC-H, Hexane:^{i}PrOH= 90:10, 0.7 ml/min, 214 nm

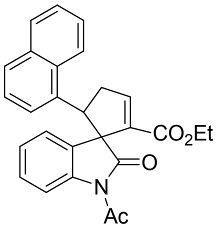

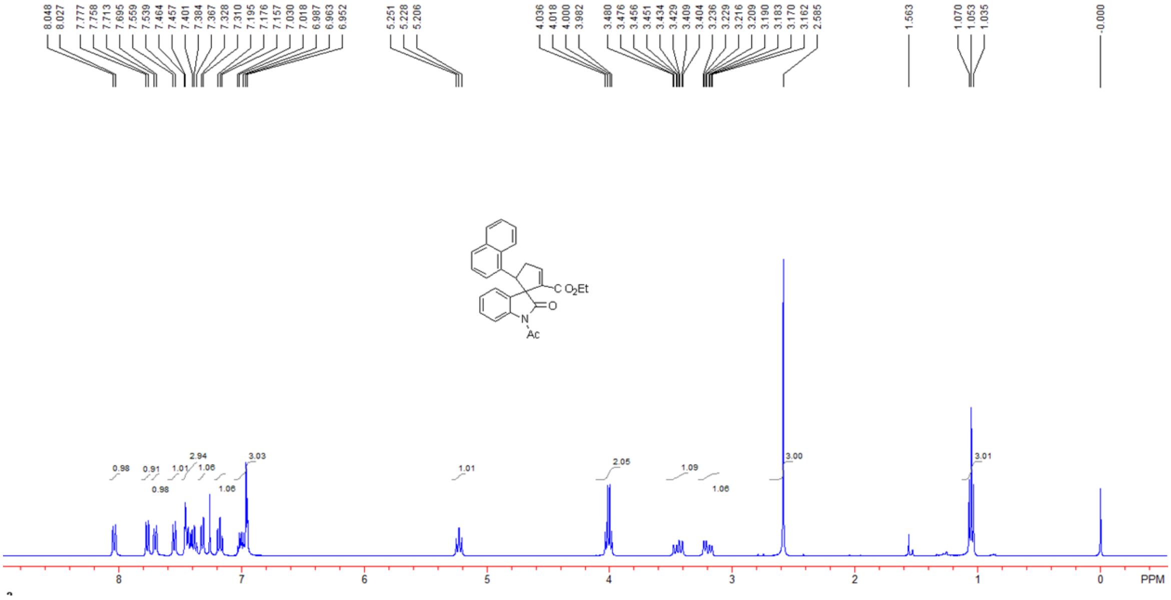

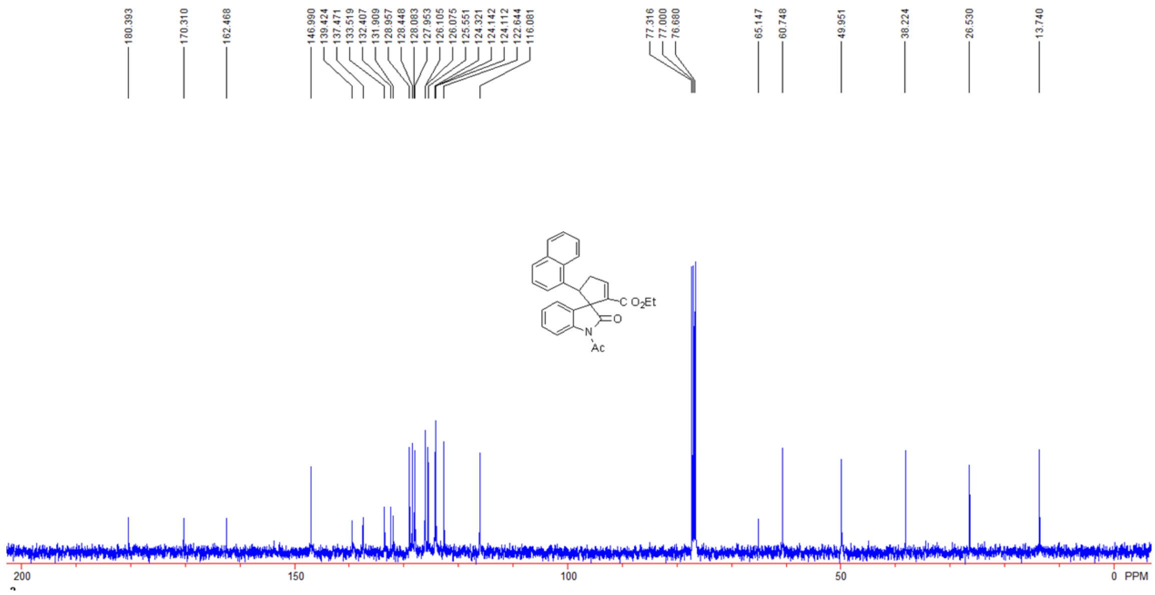

Ethyl 1'-acetyl-5-(naphthalen-1-yl)-2'-oxospiro[cyclopent[2]ene-1,3'-indoline]-2-carboxylate (7b)

A known product, 98% yield, 41 mg. ^{1}H NMR (400 MHz, $CDCl_3$, TMS) δ 8.04 (d, J = 10.0 Hz, 1H), 7.77 (d, J = 7.6 Hz, 1H), 7.70 (d, J = 7.2 Hz, 1H), 7.55 (d, J = 8.0 Hz, 1H), 7.46-7.37 (m, 3H), 7.32 (d, J = 7.2 Hz, 1H), 7.18 (t, J = 7.6 Hz, 1H), 7.03-6.95 (m, 3H), 5.23 (t, J= 8.0 Hz, 1H), 4.01 (q, J = 7.2 Hz, 2H), 3.44 (ddd, J_1 = 1.6 Hz, J_2 = 9.6 Hz, J_3 = 18.8 Hz, 1H), 3.20 (ddd, J_1 = 3.2 Hz, J_2 = 8.0 Hz, J_3 = 18.8 Hz, 1H), 2.59 (s, 3H), 1.05 (t, J = 7.2 Hz, 3H); ^{13}C NMR (100 MHz, $CDCl_3$) δ 180.4, 170.3, 162.5, 147.0, 139.4, 137.5, 133.5, 132.4, 131.9, 129.0, 128.4, 128.1, 128.0, 126.11, 126.08, 125.6, 124.3, 124.14, 124.11, 122.6, 116.1, 65.1, 60.7, 50.0, 38.2, 26.5, 13.7; $[\alpha]^{20}_{D}$ = +211.9 (c 0.5, $CHCl_3$) for 98% ee; Enantiomeric excess was determined by HPLC with a Chiralcel IA-H column, n-Heptane/EtOH = 99/1, 1.0 mL/min, 220 nm, t_{minor} = 17.318 min, t_{major} = 24.052 min.

HPLC Report

Sample Name:
Operator:
Time:13:23

Data File:WD-21-96-RAC IA n-HEP99ETOH1 220 1.0..c
Date:2014-03-05

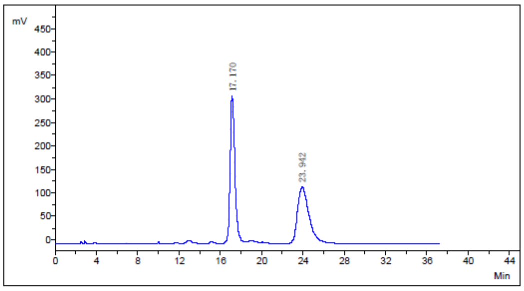

No.	PeakNo	ID. Name	R. Time	PeakHeight	PeakArea	PerCent
1	1		17.170	309964.8	9706779.8	50.8422
2	2		23.942	121277.0	9385208.2	49.1578
Total				431241.8	19091988.0	100.0000

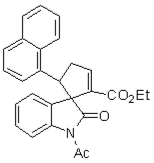

IA, n-Heptane: EtOH = 99:1 , 1.0 mL/min, 220 nm

HPLC Report

Sample Name:
Operator:
Time:14:02

Data File:WD-21-96-ASY...che
Date:2014-03-05

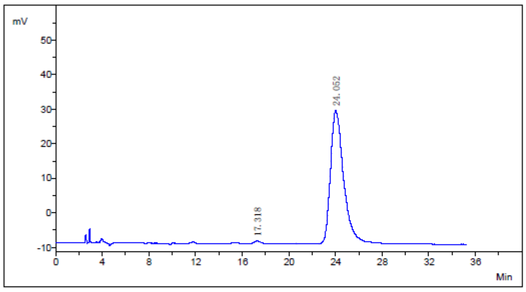

No.	PeakNo	ID. Name	R. Time	PeakHeight	PeakArea	PerCent
1	1		17.318	767.7	25015.2	0.8234
2	2		24.052	38537.5	3013057.4	99.1766
Total				39305.2	3038072.6	100.0000

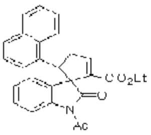

IA , n-Heptane:EtOH = 99:1, 1.0 mL/min, 220 nm

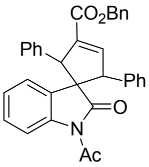

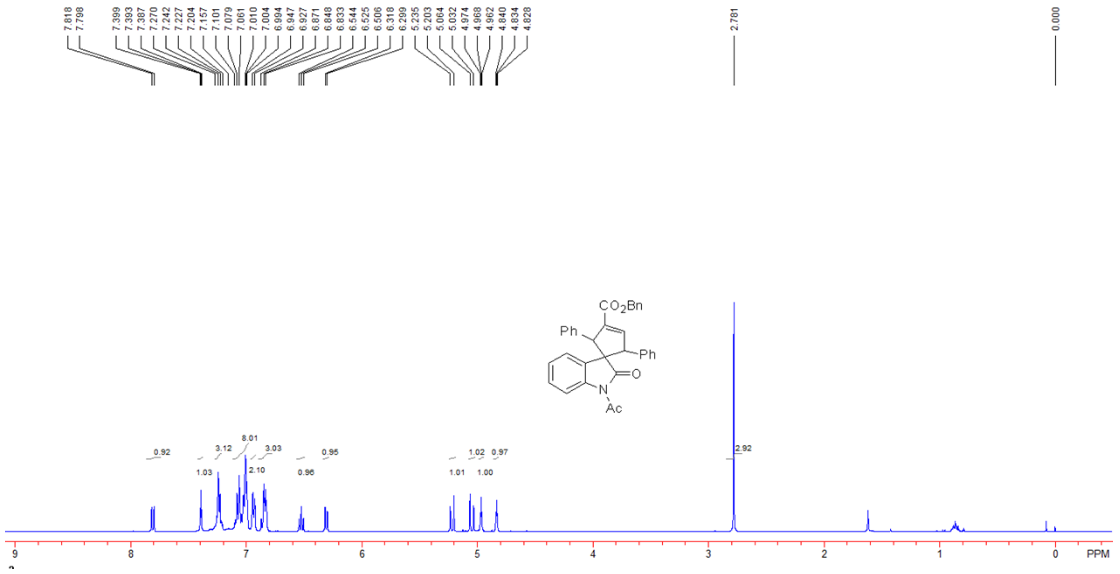

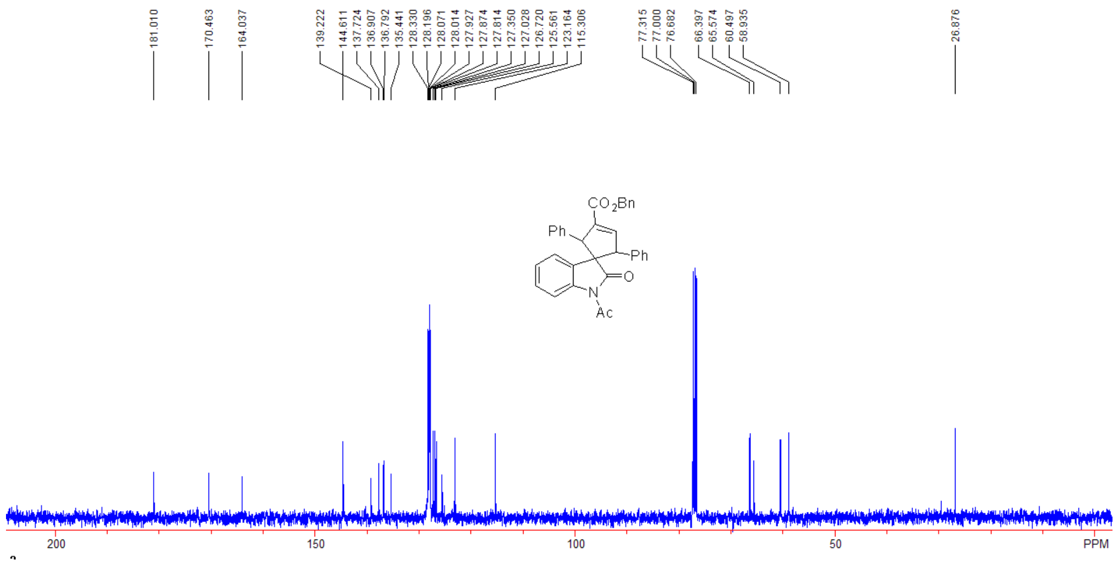

Benzyl 1'-acetyl-2'-oxo-2,5-diphenylspiro[cyclopent[3]ene-1,3'-indoline]-3-carboxylate (8)

A pale yellow solid, 89% yield, 44 mg, Mp: 138-140 ºC. ^{1}H NMR (400 MHz, $CDCl_3$, TMS) δ 7.81 (d, J = 8.0 Hz, 1H), 7.39 (t, J = 2.4 Hz, 1H), 7.27-7.20 (m, 3H), 7.10-6.99 (m, 8H), 6.95-6.93 (m, 2H), 6.87-6.83 (m, 3H), 6.53 (t, J =7.6 Hz, 1H), 6.31 (d, J = 7.6 Hz, 1H), 5.22 (d, J= 12.8 Hz, 1H), 5.05 (d, J = 12.8 Hz, 1H), 4.97 (t, J = 2.4 Hz, 1H), 4.83 (t, J = 2.4 Hz, 1H), 2.78 (s, 3H); ^{13}C NMR (100 MHz, $CDCl_3$) δ 181.0, 170.5, 164.0, 144.6, 139.2, 137.7, 136.9, 136.8, 135.4, 128.3, 128.2, 128.1, 128.0, 127.93, 127.87, 127.8, 127.4, 127.0, 126.7, 125.6, 123.2, 115.3, 66.4, 65.6, 60.5, 58.9, 26.9; IR (neat) ν 2922, 1751, 1715, 1467, 1454, 1371, 1295, 1166, 1096, 1021, 738 cm^{-1}; HRMS Calcd. for $C_{34}H_{28}NO_4^{+1}$ (M+H)$^+$: 514.2013, found: 514.1991. $[\alpha]^{20}_D$ = -30.3 (c 1.0, $CHCl_3$) for 99% ee; Enantiomeric excess was determined by HPLC with a Chiralcel AD-H column, Hexane/^{i}PrOH = 80/20, 0.6 mL/min, 214 nm, t_{major} = 12.371 min.

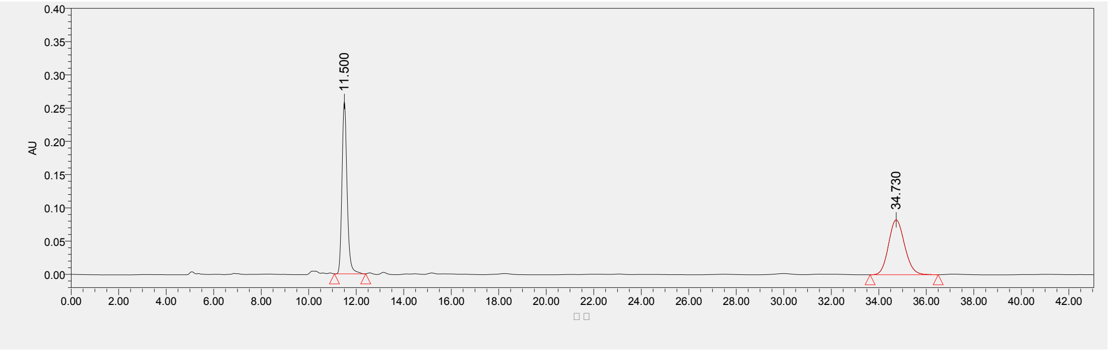

NO	R. Time	Peak Area	Precent	Peak Height
1	11.500	3875499	50.02	259157
2	34.730	3873008	49.98	82525

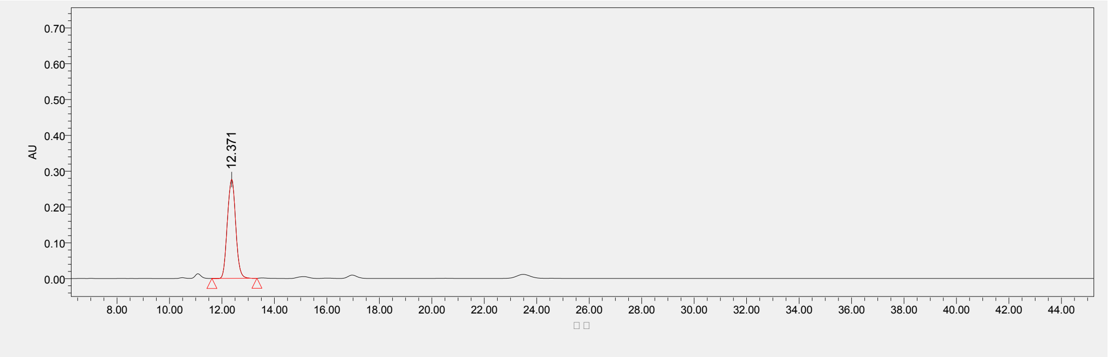

NO	R. Time	Peak Area	Precent	Peak Height
1	12.371	6121908	100.00	276352

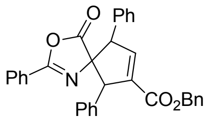

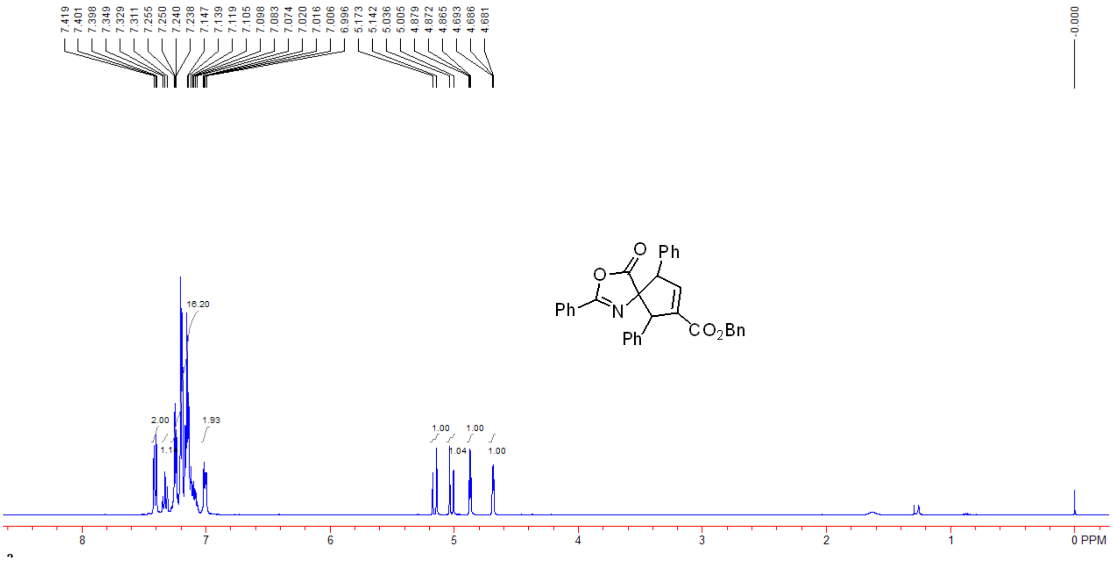

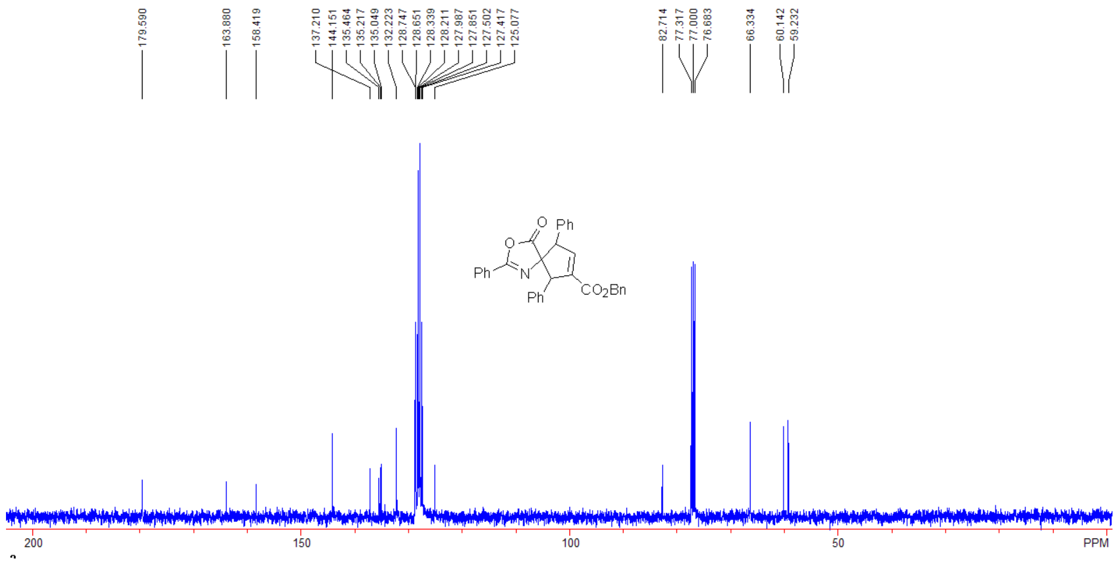

Benzyl 4-oxo-2,6,9-triphenyl-3-oxa-1-azaspiro[4.4]nona-1,7-diene-7-carboxylate (10a)

A colorless solid, 87% yield, 435 mg, Mp: 84-85 ºC. ^{1}H NMR (400 MHz, $CDCl_3$, TMS) δ 7.42-7.40 (m, 2H), 7.35-7.31 (m, 1H), 7.26-7.07 (m, 16H), 7.02-6.99 (m, 2H), 5.16 (d, J = 12.4 Hz, 1H), 5.02 (d, J = 12.4 Hz, 1H), 4.87 (t, J = 2.8 Hz, 1H), 4.69 (t, J = 2.8 Hz, 1H); ^{13}C NMR (100 MHz, $CDCl_3$) δ 179.6, 163.9, 158.4, 144.2, 137.2, 135.5, 135.2, 135.0, 132.2, 128.74, 128.65, 128.3, 128.2, 128.0, 127.9, 127.5, 127.4, 125.1, 82.7, 66.3, 60.1, 59.2; IR (neat) ν 3029, 1808, 1716, 1656, 1493, 1453, 1273, 1233, 1097, 960, 695 cm^{-1}; HRMS Calcd. for $C_{33}H_{26}NO_4^{+1}$ $(M+H)^+$: 500.1856, found: 500.1856. $[\alpha]^{20}_{D}$ = +39.5 (c 3.5, $CHCl_3$) for >99% ee; Enantiomeric excess was determined by HPLC with a Chiralcel AD-H column, Hexane/^{i}PrOH = 90/10, 0.6 mL/min, 214 nm, t_{major} = 14.857 min.

N2000 数据工作站 1

实验时间：2013-05-13，17:55:15
谱图文件：I:\regio-and enantio\液相\wd-18-16race-90100.6214.org

实验者：
报告时间：2013-10-22，21:33:00
积分方法：面积归一法

使用仪器类型：气相色谱　　检测器：FID　　进样器：分流

柱温：程序升温

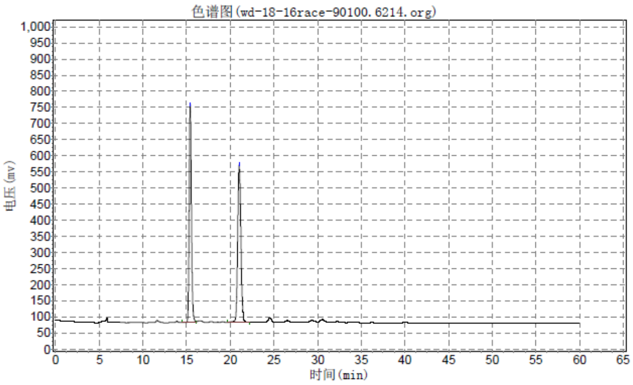

分析结果表

峰号	峰名	保留时间	峰高	峰面积	含量
1		15.415	673848.500	12328841.000	49.6782
2		21.015	488139.500	12488574.000	50.3218
总计			1161988.000	24817415.000	100.0000

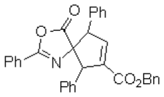

AD-H, Hexane:iPrOH = 90:10, 0.6 mL/min, 214 nm

N2000 数据工作站 1

实验时间：2013-09-13，16:20:13
谱图文件：I:\regio-and enantio\液相\wd-19-73re-asy-AD-9010-0.6-214.org

实验者：
报告时间：2013-10-22，21:31:56
积分方法：面积归一法

使用仪器类型：气相色谱　　检测器：FID　　进样器：分流

柱温：程序升温

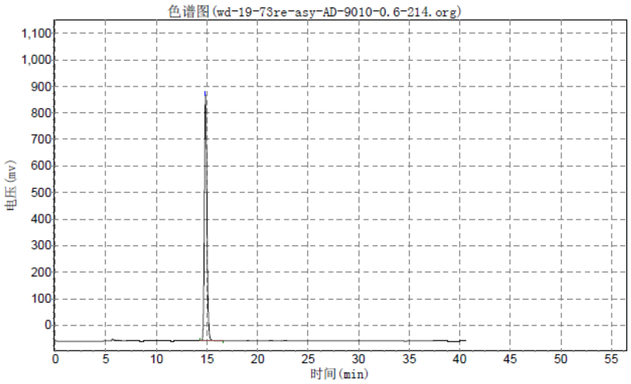

分析结果表

峰号	峰名	保留时间	峰高	峰面积	含量
1		14.857	917458.438	16438201.000	100.0000
总计			917458.438	16438201.000	100.0000

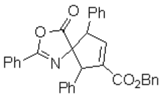

AD-H, Hexane:iPrOH = 90:10, 0.6 mL/min, 214 nm

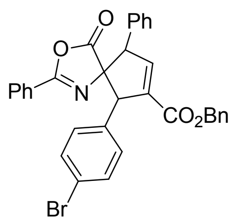

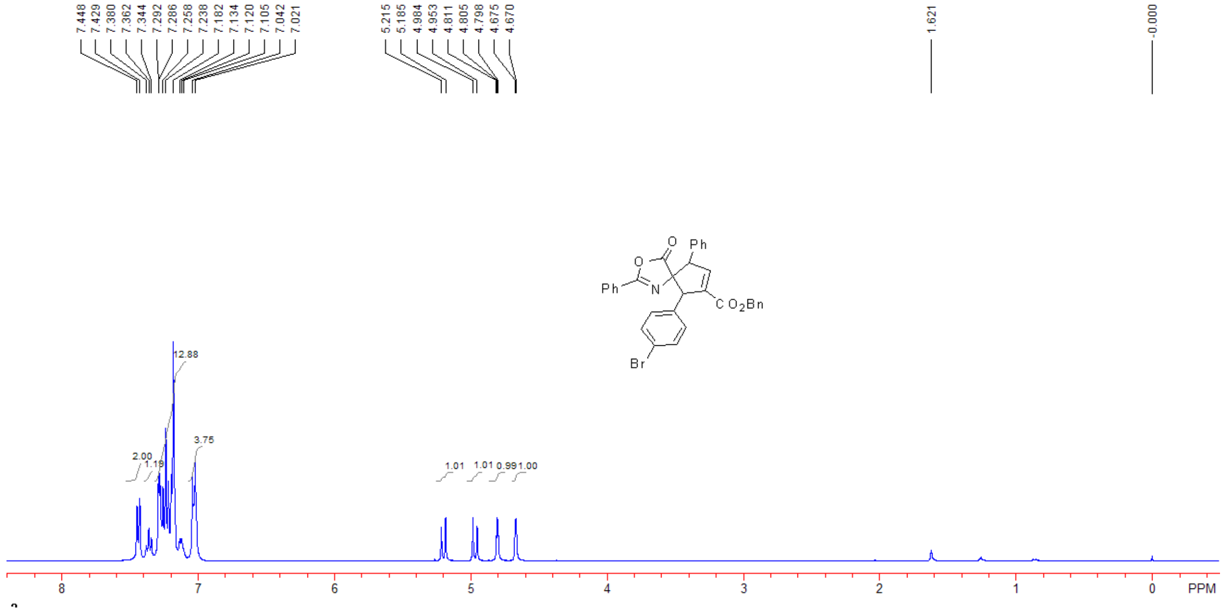

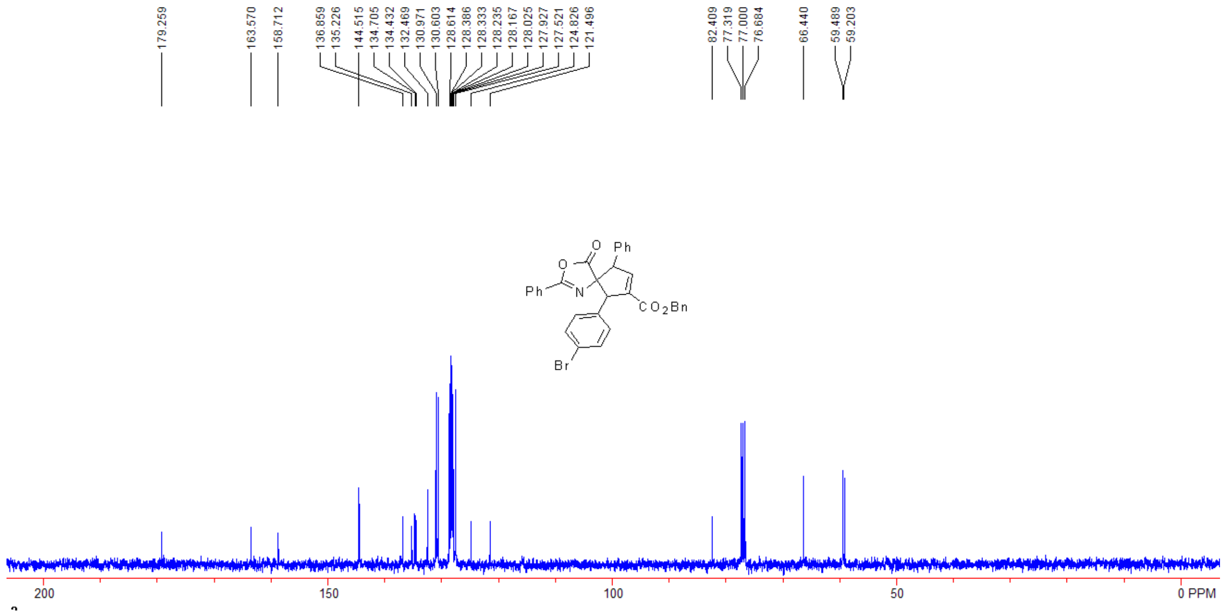

Benzyl 6-(4-bromophenyl)-4-oxo-2,9-diphenyl-3-oxa-1-azaspiro[4.4]nona-1,7-diene-7-carboxylate (10b)

A yellowish syrupy compound, 82% yield, 46 mg. ^{1}H NMR (400 MHz, $CDCl_3$, TMS) δ 7.44 (d, J = 7.6 Hz, 2H), 7.36 (t, J = 7.2 Hz, 1H), 7.29-7.11 (m, 13H), 7.04-7.02 (m, 4H), 5.20 (d, J = 12.0 Hz, 1H), 4.97 (d, J = 12.0 Hz, 1H), 4.81 (t, J = 2.0 Hz, 1H), 4.67 (t, J = 2.0 Hz, 1H); ^{13}C NMR (100 MHz, $CDCl_3$) δ 179.3, 163.6, 158.7, 144.5, 136.9, 135.2, 134.7, 134.4, 132.4, 131.1, 130.6,

128.6, 128.4, 128.3, 128.24, 128.17, 128.0, 127.9, 127.5, 124.8, 121.5, 82.4, 66.4, 59.5, 59.2; IR (neat) ν 2923, 1808, 1716, 1655, 1487, 1451, 1284, 1106, 1059, 958, 879, 750, 692 cm^{-1}; HRMS Calcd. for $C_{33}H_{25}BrNO_4^{+1}$ $(M+H)^+$: 578.0961, found: 578.0969. $[\alpha]^{20}_D$ = +22.9 (c 2.0, $CHCl_3$) for >99% ee; Enantiomeric excess was determined by HPLC with a Chiralcel PC-2 column, Hexane/^{i}PrOH = 90/10, 0.5 mL/min, 214 nm, t_{major} = 49.642 min.

N2000 数据工作站 1

实验时间：2014-04-24, 18:31:23
谱图文件:I:\SIOC液相\spiro\wd-22-8re-rac-PC-2-9010-0.5-214.org

实验者：
报告时间：2014-05-09, 11:18:18
积分方法:面积归一法

使用仪器类型:气相色谱　　检测器:FID　　进样器:分流

柱温:程序升温

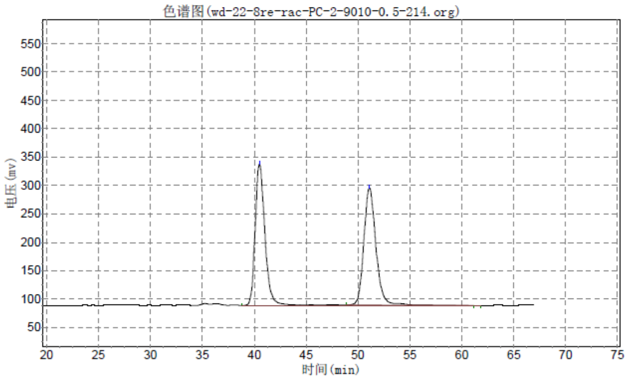

分析结果表

峰号	峰名	保留时间	峰高	峰面积	含量
1		40.515	248077.656	16894848.000	50.2120
2		51.115	205726.313	16752192.000	49.7880
总计			453803.969	33647040.000	100.0000

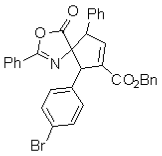

PC-2, Hexane:^{i}PrOH = 90/10, 0.5 mol/min, 214 nm

实验时间：2014-04-24, 17:22:35
谱图文件：I:\SIOC液相\spiro\wd-22-8-asy-PC-2-9010-0.5-214.org

实验者：
报告时间：2014-05-09, 11:24:03
积分方法：面积归一法

使用仪器类型：气相色谱 检测器：FID 进样器：分流

柱温：程序升温

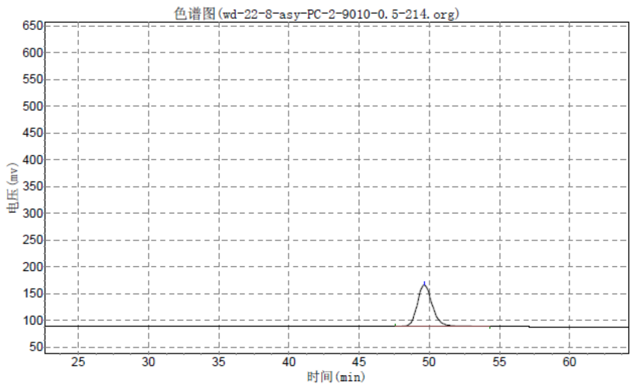

分析结果表

峰号	峰名	保留时间	峰高	峰面积	含量
1		49.642	77452.805	5768120.000	100.0000
总计			77452.805	5768120.000	100.0000

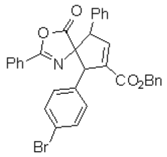

PC-2, Hexane:^{i}PrOH = 90/10, 0.5 mol/min, 214 nm

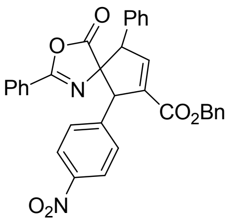

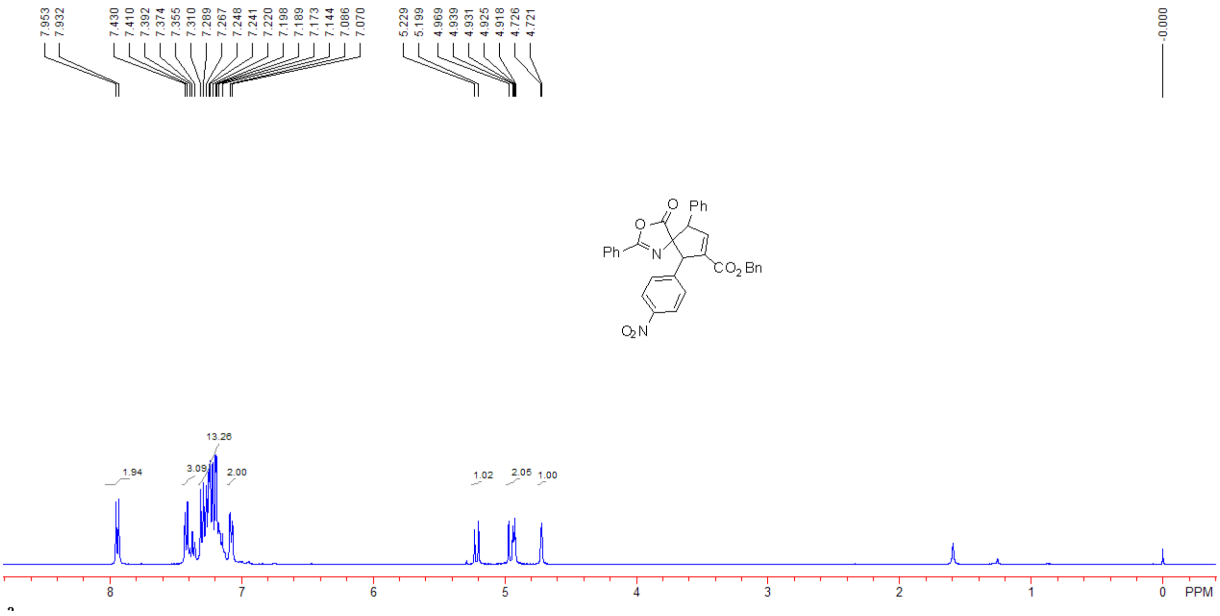

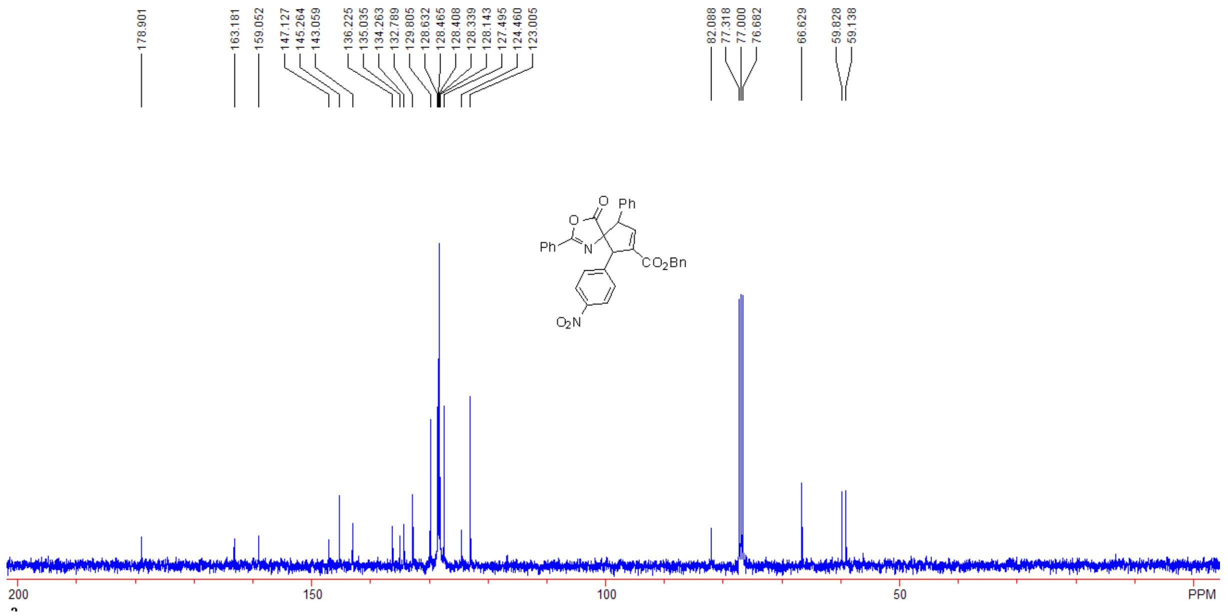

Benzyl 6-(4-nitrophenyl)-4-oxo-2,9-diphenyl-3-oxa-1-azaspiro[4.4]nona-1,7-diene-7-carboxylate (10c)

A syrupy compound, 71% yield, 38 mg. ^{1}H NMR (400 MHz, $CDCl_3$, TMS) δ 7.94 (d, J = 8.4 Hz, 2H), 7.43-7.36 (m, 3H), 7.31-7.14 (m, 13H), 7.08 (d, J = 6.4 Hz, 2H), 5.21 (d, J = 12.0 Hz, 1H), 4.97-4.92 (m, 2H), 4.72 (t, J = 2.4 Hz, 1H); ^{13}C NMR (100 MHz, $CDCl_3$) δ 178.9, 163.2, 159.1, 147.1, 145.3, 143.1, 136.2, 135.0, 134.3, 132.8, 129.8, 128.6, 128.5, 128.4, 128.3, 128.1, 127.5, 124.5, 123.0, 82.1, 66.6, 59.8, 59.1; IR (neat) ν 2927, 1809, 1716, 1655, 1519, 1494, 1451, 1346, 1275, 1108, 960, 880, 750, 691 cm^{-1}; HRMS Calcd. for $C_{33}H_{25}N_2O_6^{+1}$ (M+H)$^+$: 545.1707, found: 545.1711. $[\alpha]^{20}_D$ = +55.6 (c 0.6, $CHCl_3$) for >99% ee; Enantiomeric excess was determined by HPLC with a Chiralcel PC-2 column, Hexane/^{i}PrOH = 90/10, 0.7 mL/min, 214 nm, t_{major} = 34.003 min.

实验时间：2014-05-28, 12:28:18
谱图文件:I:\SIOC液相\spiro\wd-22-9-race-re-PC-2-9010-0.9-214.org

实验者：
报告时间：2014-05-28, 16:15:39
积分方法:面积归一法

使用仪器类型:气相色谱 检测器:FID 进样器:分流

柱温:程序升温

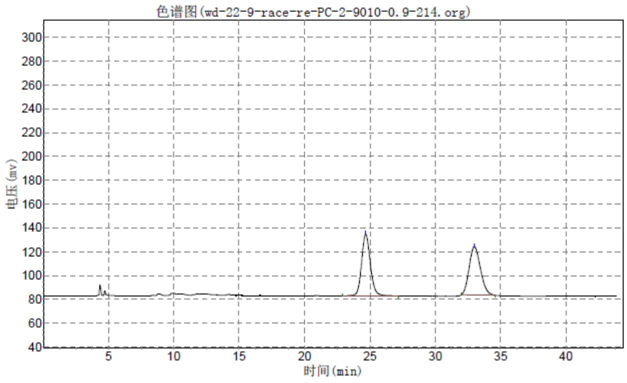

分析结果表

峰号	峰名	保留时间	峰高	峰面积	含量
1		24.675	51796.070	2424581.000	49.7948
2		32.983	40652.609	2444564.500	50.2052
总计			92448.680	4869145.500	100.0000

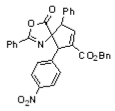

PC-2, Hexane:^{i}PrOH = 90:10, 0.9 mL/min, 214 nm

N2000 数据工作站 1

实验时间：2014-05-28, 11:42:30
谱图文件:I:\SIOC液相\spiro\wd-22-9-asy-PC-2-9010-0.9-214.org

实验者：
报告时间：2014-05-28, 16:23:50
积分方法:面积归一法

使用仪器类型:气相色谱 检测器:FID 进样器:分流

柱温:程序升温

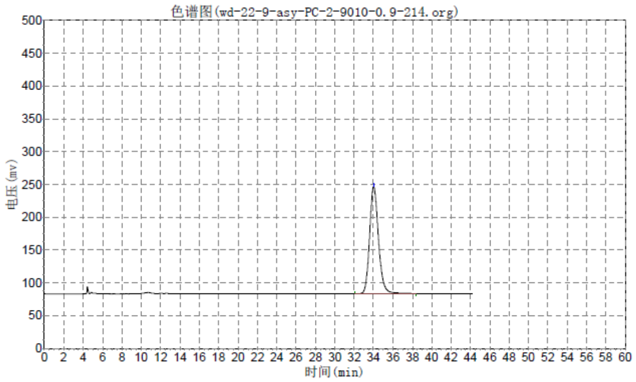

分析结果表

峰号	峰名	保留时间	峰高	峰面积	含量
1		34.003	162902.344	10390608.000	100.0000
总计			162902.344	10390608.000	100.0000

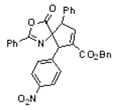

PC-2, Hexane:^{i}PrOH = 90:10, 0.9 mL/min, 214 nm

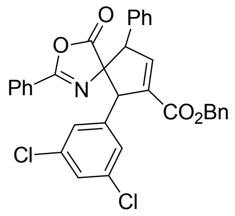

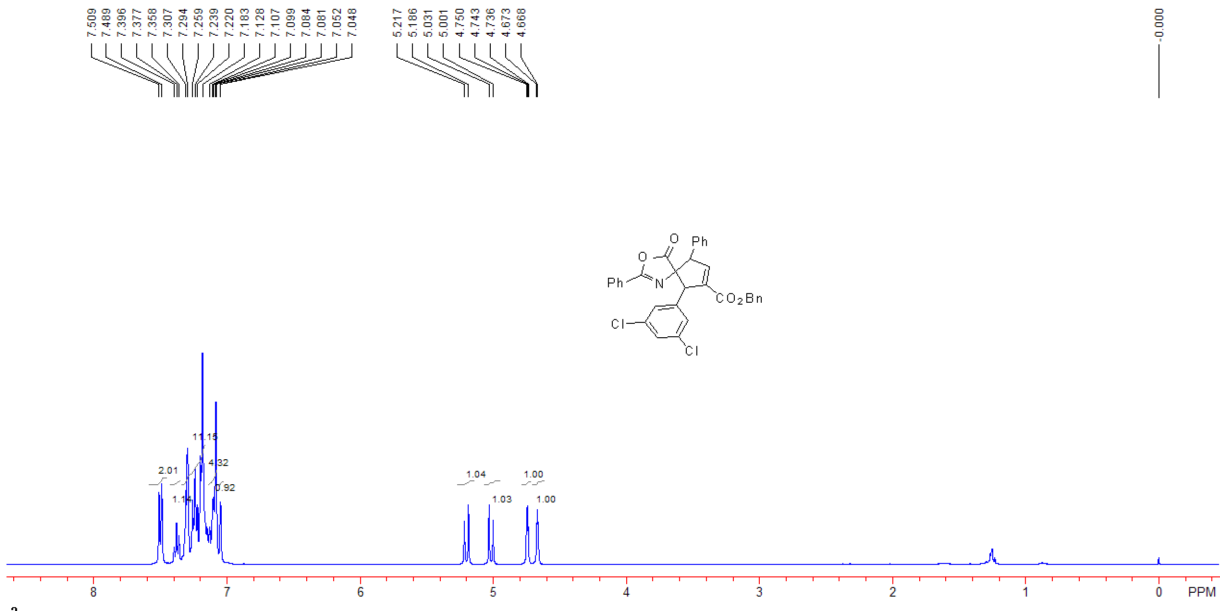

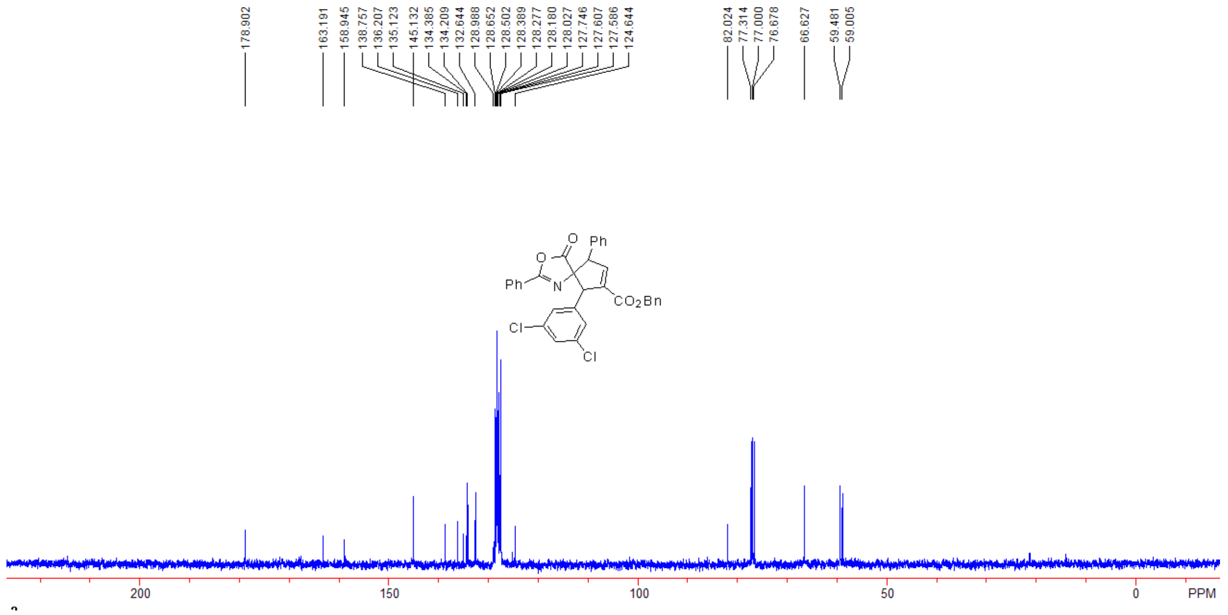

Benzyl 6-(3,5-dichlorophenyl)-4-oxo-2,9-diphenyl-3-oxa-1-azaspiro[4.4]nona-1,7-diene-7-carboxylate (10d)

A syrupy compound, 85% yield, 48 mg. ^{1}H NMR (400 MHz, $CDCl_3$, TMS) δ 7.50 (d, J = 8.0 Hz, 2H), 7.38 (t, J = 7.6 Hz, 1H), 7.31-7.18 (m, 11H), 7.13-7.05 (m, 4H), 7.05-7.04 (m, 1H), 5.20 (d, J = 12.4 Hz, 1H), 5.02 (d, J = 12.4 Hz, 1H), 4.74 (t, J = 2.8 Hz, 1H), 4.67 (t, J = 2.8 Hz, 1H); ^{13}C NMR (100 MHz, $CDCl_3$) δ 178.9, 163.2, 158.9, 145.1, 138.8, 136.2, 135.1, 134.4, 134.2, 132.6,

129.0, 128.7, 128.5, 128.4, 128.3, 128.2, 128.0, 127.7, 127.61, 127.59, 124.6, 82.0, 66.6, 59.5, 59.0; IR (neat) ν 2927, 1812, 1718, 1655, 1569, 1452, 1275, 1093, 961, 878, 750, 695 cm^{-1}; HRMS Calcd. for $C_{33}H_{24}Cl_2NO_4^{+1}$ $(M+H)^+$: 568.1077, found: 568.1086. $[\alpha]^{20}_D$ = +39.5 (c 2.4, $CHCl_3$) for >99% ee; Enantiomeric excess was determined by HPLC with a Chiralcel PC-2 column, Hexane/^{i}PrOH = 90/10, 0.5 mL/min, 214 nm, t_{major} = 58.405 min.

N2000 数据工作站 1

实验时间：2014-05-05, 20:08:35
谱图文件：I:\SIOC液相\spiro\wd-22-15-rac-PC-2-9010-0.5-214.org

实验者：
报告时间：2014-05-09, 11:29:13
积分方法：面积归一法

使用仪器类型：气相色谱 检测器：FID 进样器：分流

柱温：程序升温

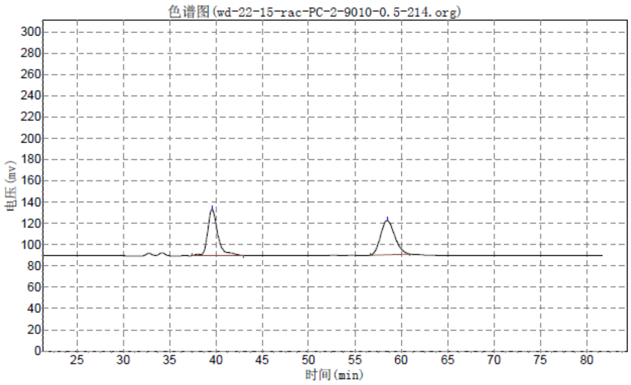

分析结果表

峰号	峰名	保留时间	峰高	峰面积	含量
1		39.592	43537.438	3283894.000	49.2498
2		58.482	32190.211	3383942.750	50.7502
总计			75727.648	6667836.750	100.0000

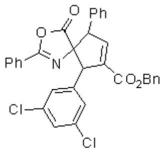

PC-2, Hexane:iPrOH = 90/10, 0.5 mol/min, 214 nm

实验时间：2014-05-05, 18:45:22
谱图文件：I:\SIOC液相\spiro\wd-22-15-asy-PC-2-9010-0.5-214.org

实验者：
报告时间：2014-05-09, 11:32:43
积分方法：面积归一法

使用仪器类型：气相色谱　　检测器：FID　　进样器：分流

柱温：程序升温

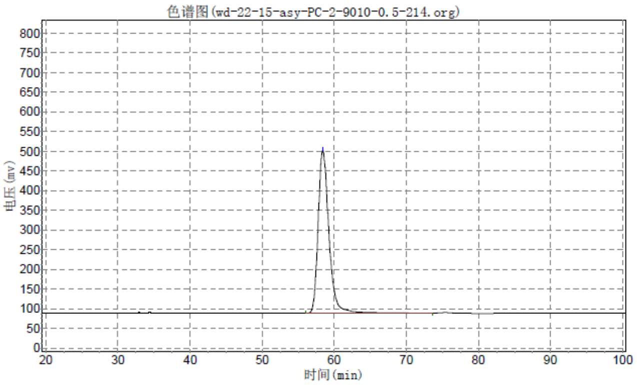

分析结果表

峰号	峰名	保留时间	峰高	峰面积	含量
1		58.405	413622.219	43206536.000	100.0000
总计			413622.219	43206536.000	100.0000

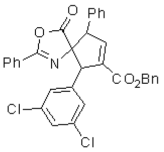

PC-2, Hexane:iPrOH = 90/10, 0.5 mol/min, 214 nm

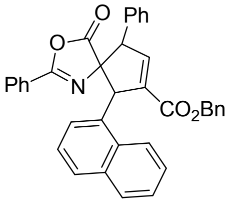

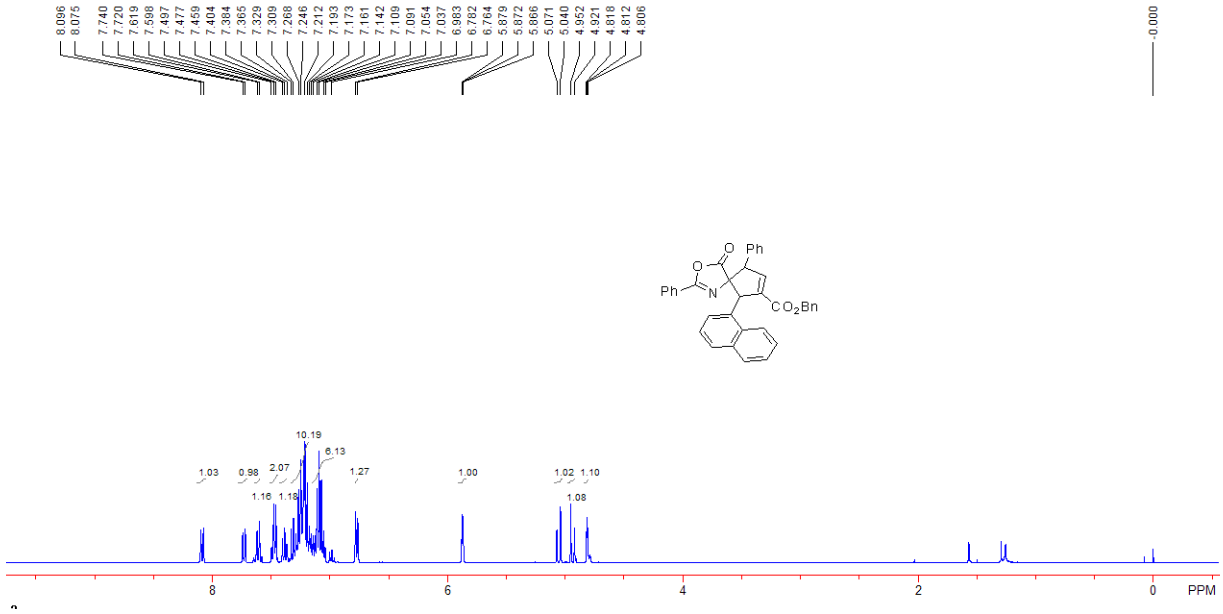

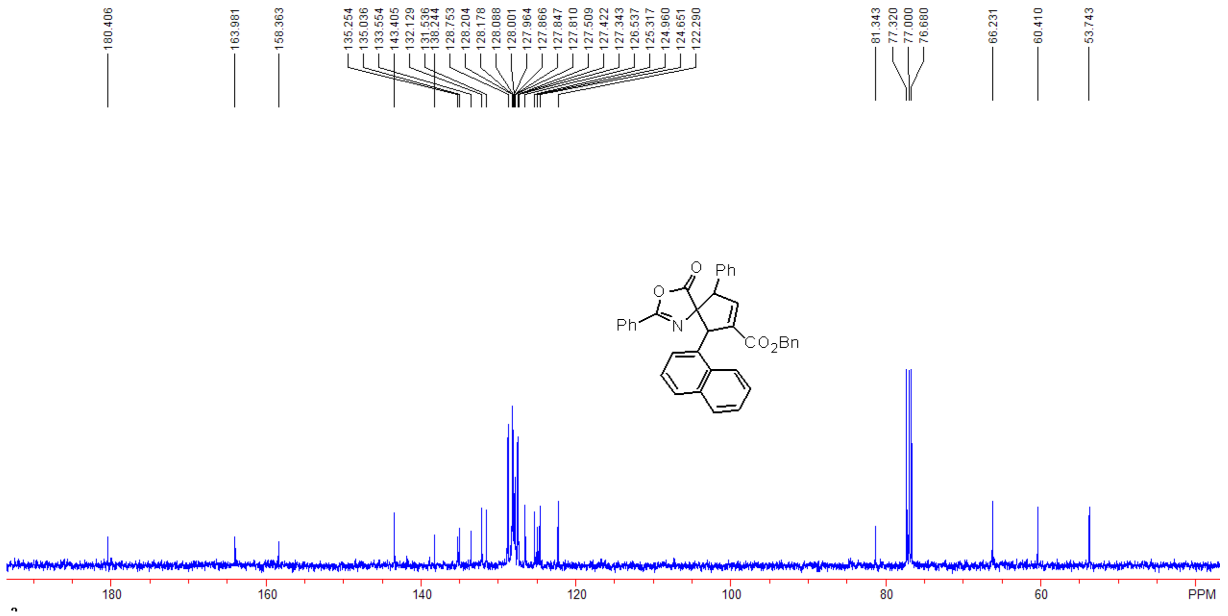

Benzyl 6-(naphthalen-1-yl)-4-oxo-2,9-diphenyl-3-oxa-1-azaspiro[4.4]nona-1,7-diene-7-carboxylate (10e)

A yellowish solid, 95% yield, 52 mg, Mp: 72-73 ºC. ^{1}H NMR (400 MHz, $CDCl_3$, TMS) δ 8.09 (d, J = 8.4 Hz, 1H), 7.73 (d, J = 8.0 Hz, 1H), 7.62-7.60 (m, 1H), 7.50-7.46 (m, 2H), 7.38 (t, J = 8.0 Hz, 1H), 7.33-7.16 (m, 10H), 7.14-6.99 (m, 6H), 6.77 (d, J = 7.2 Hz, 1H), 5.87 (t, J = 2.4 Hz, 1H), 5.06 (d, J = 12.4 Hz, 1H), 4.94 (d, J = 12.4 Hz, 1H), 4.81 (t, J = 2.4 Hz, 1H); ^{13}C NMR (100 MHz, $CDCl_3$) δ 180.4, 164.0, 158.4, 143.4, 138.2, 135.3, 135.0, 133.6, 132.1, 131.5, 128.8, 128.20, 128.18, 128.1, 128.00, 127.96, 127.9, 127.84, 127.81, 127.5, 127.4, 127.3, 126.5, 125.3, 125.0, 124.7, 122.3, 81.3, 66.2, 60.4, 53.7; IR (neat) ν 3064, 1806, 1717, 1655, 1495, 1451, 1287, 1247, 1105, 959, 879, 734, 694 cm^{-1}; HRMS Calcd. for $C_{37}H_{28}NO_4^{+1}$ (M+H)$^+$: 550.2013, found: 550.2020. $[\alpha]^{20}_{D}$ = +89.5 (c 0.5, $CHCl_3$) for >99% ee; Enantiomeric excess was determined by HPLC with a Chiralcel PC-2 column, Hexane/^{i}PrOH = 90/10, 0.5 mL/min, 214 nm, t_{major} = 35.273 min.

实验时间：2014-03-11,10:38:01
谱图文件:I:\6.regio-and enantio\液相\wd-22-1A-race-PC-2-9010-0.5-214.org

实验者：
报告时间：2014-03-11,16:57:38
积分方法:面积归一法

使用仪器类型:气相色谱 检测器:FID 进样器:分流

柱温:程序升温

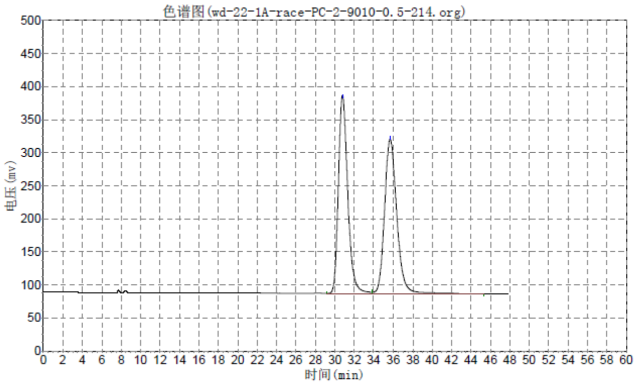

分析结果表

峰号	峰名	保留时间	峰高	峰面积	含量
1		30.848	298563.313	19999694.000	49.5691
2		35.715	233528.703	20347404.000	50.4309
总计			532092.016	40347098.000	100.0000

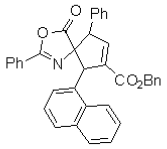

PC-2, Hexane:iPrOH = 90:10, 0.5 ml/min, 214 nm

实验时间：2014-03-11,11:37:23
谱图文件:I:\6.regio-and enantio\液相\wd-22-1B-asy-PC-2-9010-0.5-214.org

实验者：
报告时间：2014-03-11,16:59:24
积分方法:面积归一法

使用仪器类型:气相色谱 检测器:FID 进样器:分流

柱温:程序升温

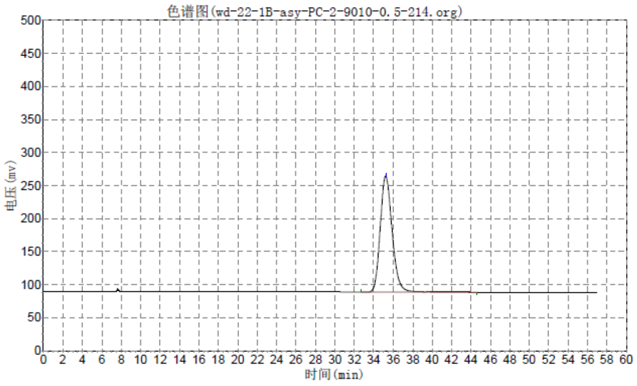

分析结果表

峰号	峰名	保留时间	峰高	峰面积	含量
1		35.273	175751.500	15105462.000	100.0000
总计			175751.500	15105462.000	100.0000

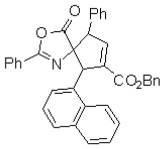

PC-2, Hexane:iPrOH = 90:10, 0.5 ml/min, 214 nm

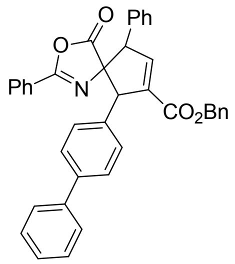

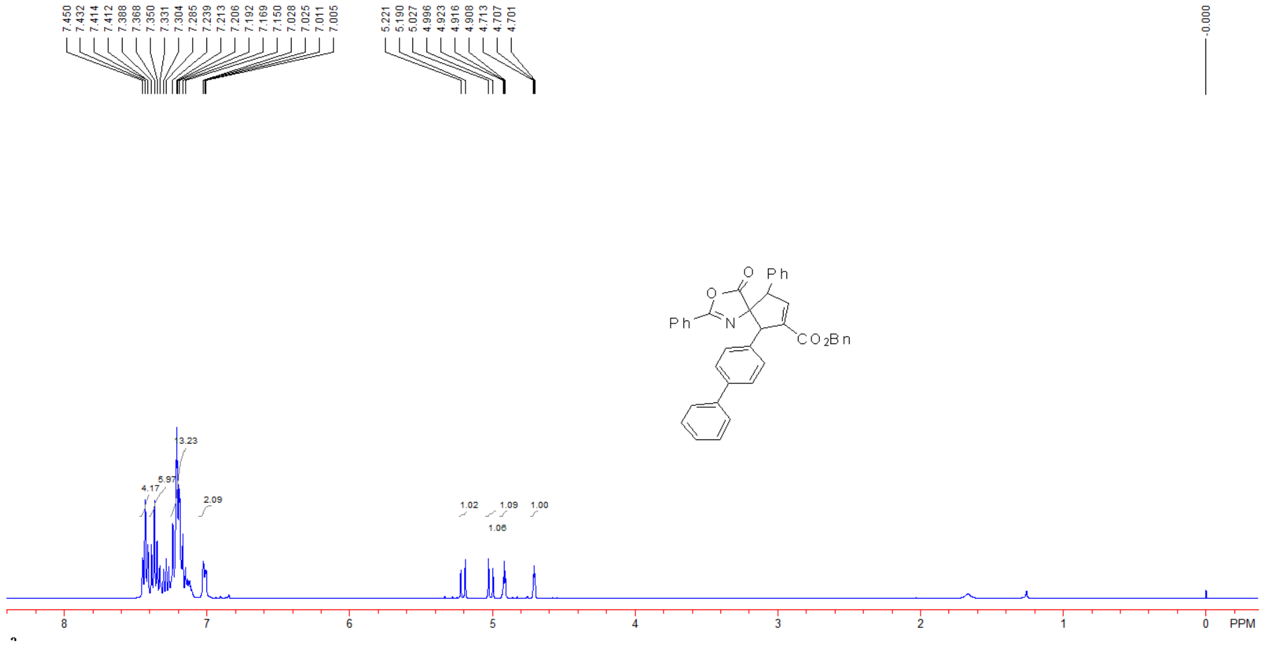

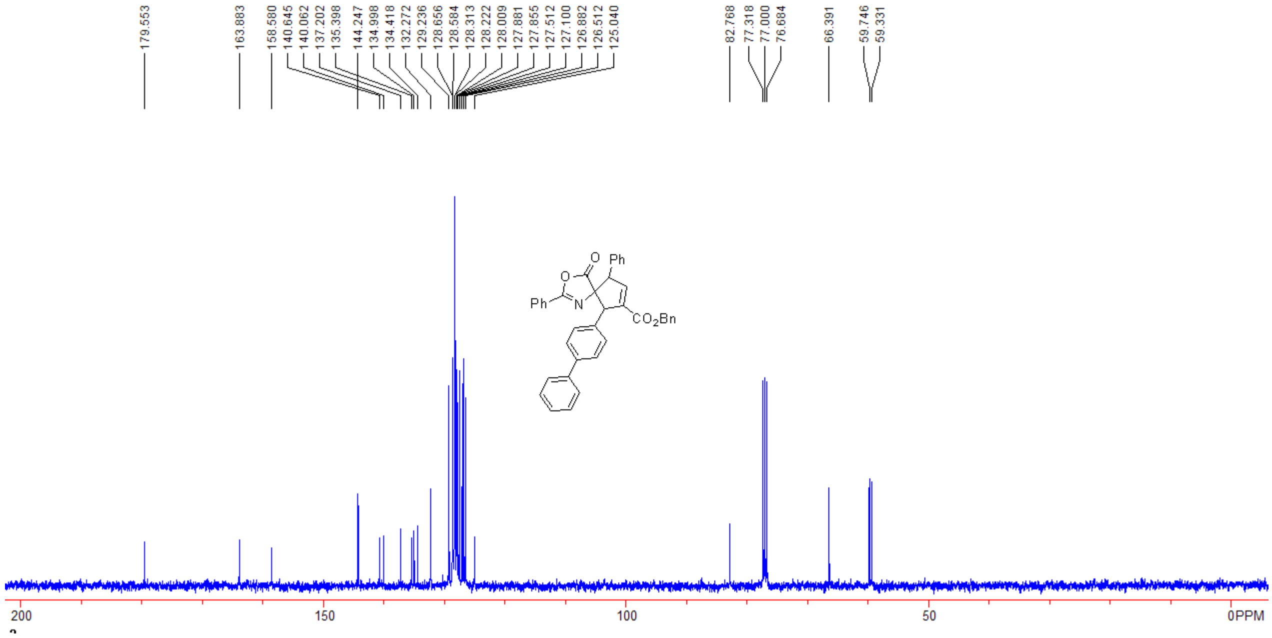

Benzyl 6-([1,1'-biphenyl]-4-yl)-4-oxo-2,9-diphenyl-3-oxa-1-azaspiro[4.4]nona-1,7-diene -7-carboxylate (10f)

A white solid, 80% yield, 46 mg, Mp: 64-66 ºC. ^{1}H NMR (400 MHz, $CDCl_3$, TMS) δ 7.45-7.41 (m, 4H), 7.39-7.29 (m, 6H), 7.24-7.15 (m, 13H), 7.03-7.01 (m, 2H), 5.21 (d, J = 12.4 Hz, 1H),

5.01 (d, J = 12.4 Hz, 1H), 4.92 (t, J = 2.4 Hz, 1H), 4.71 (t, J = 2.4 Hz, 1H); ^{13}C NMR (100 MHz, $CDCl_3$) δ 179.6, 163.9, 158.6, 144.2, 140.6, 140.1, 137.2, 135.4, 135.0, 134.4, 132.3, 129.2, 128.7, 128.6, 128.3, 128.2, 128.0, 127.88, 127.86, 127.5, 127.1, 126.9, 126.5, 125.0, 82.8, 66.4, 59.7, 59.3; IR (neat) ν 3028, 1807, 1716, 1655, 1494, 1451, 1319, 1290, 1234, 1106, 959, 879, 754, 692 cm^{-1}; HRMS Calcd. for $C_{37}H_{28}NO_4^{+1}$ (M+H)$^+$: 576.2169, found: 576.2173. $[\alpha]^{20}_D$ = +25.9 (c 2.3, $CHCl_3$) for >99% ee with 97:3 dr; Enantiomeric excess was determined by HPLC with a Chiralcel PC-2 column, Hexane/^{i}PrOH = 90/10, 0.8 mL/min, 214 nm, t_{major} = 24.535 min.

N2000 数据工作站 1

实验时间：2014-08-22, 14:13:55
谱图文件：J:\SIOC液相\王德\wd-22-32A-rac-PC-2-9010-0.8-214.org

实验者：
报告时间：2014-08-26, 22:31:02
积分方法：面积归一法

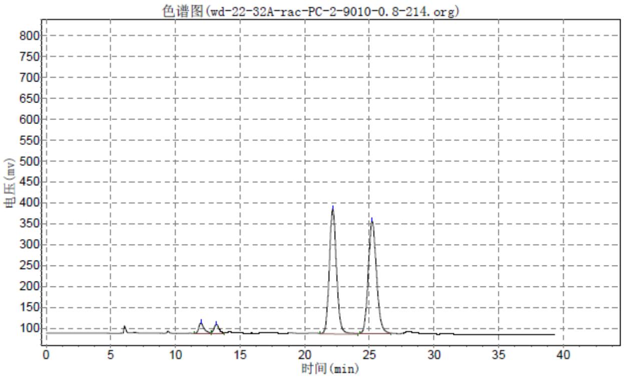

分析结果表

峰号	峰名	保留时间	峰高	峰面积	含量
1		11.965	24673.375	707454.688	2.8592
2		13.152	21378.002	617669.375	2.4963
3		22.157	298463.906	11630779.000	47.0065
4		25.210	266543.594	11787019.000	47.6379
总计			611058.877	24742922.063	100.0000

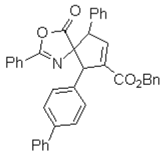

PC-2, Hexane:^{i}PrOH = 90:10, 0.8 ml/min, 214 nm

实验时间：2014-08-22, 14:58:17
谱图文件:J:\SIOC液相\王德\wd-22-32B-asy-PC-2-9010-0.8-214.org

实验者：
报告时间：2014-08-26, 22:36:48
积分方法:面积归一法

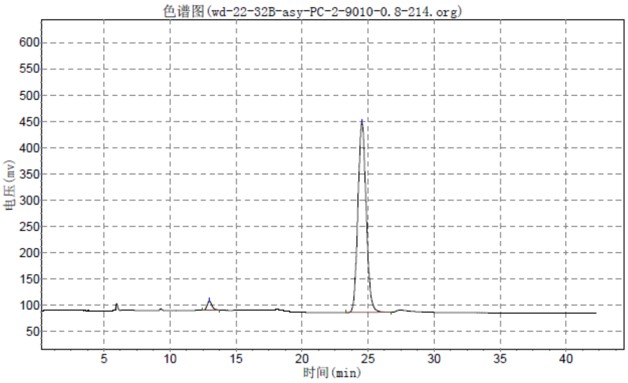

分析结果表

峰号	峰名	保留时间	峰高	峰面积	含量
1		12.973	17223.568	437533.531	2.6986
2		24.535	361739.000	15775939.000	97.3014
总计			378962.568	16213472.531	100.0000

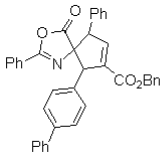

PC-2, Hexane:^{i}PrOH = 90:10, 0.8 ml/min, 214 nm

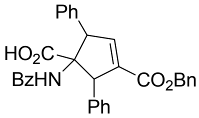

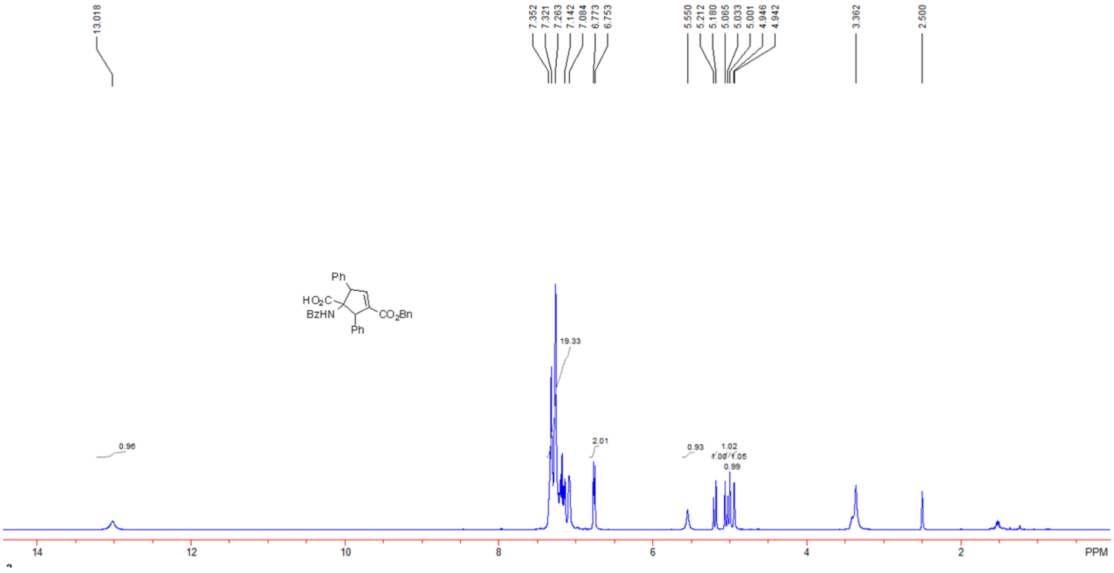

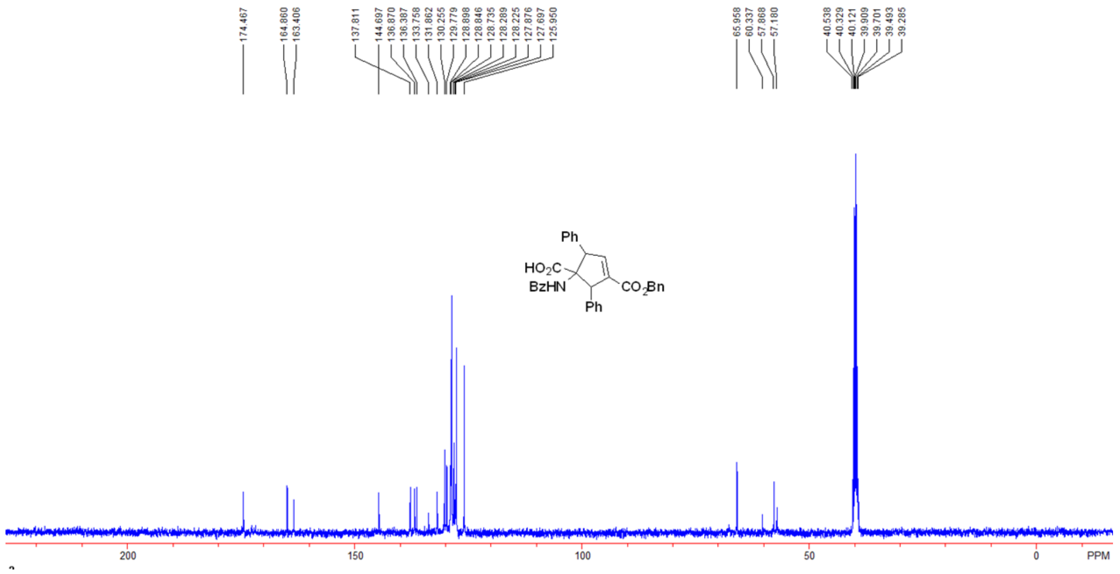

1-Benzamido-3-((benzyloxy)carbonyl)-2,5-diphenylcyclopent-3-enecarboxylic acid (11)

A pale yellow solid, 93% yield, 46 mg, Mp: 184-185 ºC. ^{1}H NMR (400 MHz, d^{6}-DMSO, TMS) δ 13.0 (br, 1H), 7.35-7.08 (m, 19H), 6.76 (d, J = 8.0 Hz, 2H), 5.55 (s, 1H), 5.20 (d, J = 12.8 Hz, 1H), 5.05 (d, J = 12.8 Hz, 1H), 5.00 (s, 1H), 4.94 (d, J = 1.6 Hz, 1H); ^{13}C NMR (100 MHz, d^{6}-DMSO) δ 174.5, 164.9, 163.4, 144.7, 137.8, 136.9, 136.4, 133.8, 131.9, 130.3, 129.8, 128.9, 128.8, 128.7, 128.3, 128.2, 127.9, 127.7, 126.0, 66.0, 60.3, 57.9, 57.2; IR (neat) ν 3413, 3025, 1716, 1670, 1646, 1510, 1481, 1454, 1273, 1232, 1097, 698 cm^{-1}; HRMS Calcd. for $C_{33}H_{28}NO_5^{+1}$ (M+H)$^+$: 518.1962, found: 518.1964. $[\alpha]^{20}_{D}$ = +25.6 (c 0.3, $CHCl_3$) for >99% ee; Enantiomeric excess was determined by HPLC with a Chiralcel IC-H column, Hexane/^{i}PrOH = 60/40, 0.5 mL/min, 230 nm, t_{minor} = 15.877 min, t_{major} = 25.127 min.

HPLC REPORT

Sample Name:wd-19-71-rac-ic-6-4-0.5-230.che
Time:09:48
Column:
Wave Length:

Date:2013-09-22
Method:
Flow Rate:
Mobile Phase:

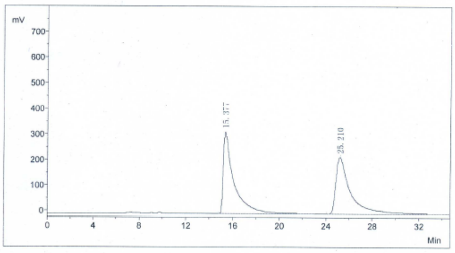

No.	PeakNo	ID.Name	R.Time	PeakHeight	PeakArea	PerCent
1	1	Unknown	15.377	318782.9	20067999.6	50.4998
2	2	Unknown	25.210	222195.6	19670737.2	49.5002
Total				540978.6	39738736.8	100.0000

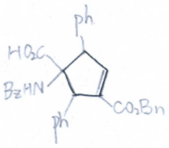

IC-H, iPrOH : Hexane = 60:40
0.5 ml/min, 230 nm

HPLC REPORT

Sample Name:wd-19-79.che
Time:10:25
Column:
Wave Length:

Date:2013-09-22
Method:
Flow Rate:
Mobile Phase:

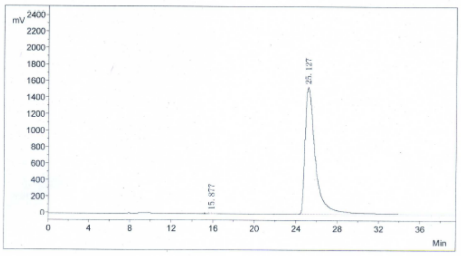

No.	PeakNo	ID.Name	R.Time	PeakHeight	PeakArea	PerCent
1	1	Unknown	15.877	856.3	14011.9	0.0127
2	2	Unknown	25.127	1531718.0	109964789.4	99.9873
Total				1532574.3	109978801.3	100.0000

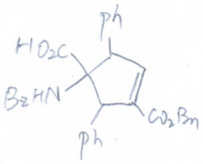

IC-H, 60:40, 0.5 ml/min, 23[illegible] nm

8. X-ray data of 3m, 5j, 7b

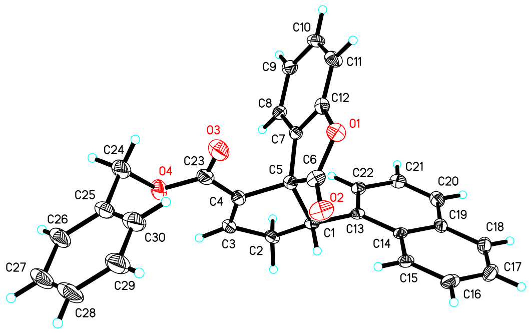

The crystal data of **3m** have been deposited in CCDC with number 961159. Empirical formula: $C_{30}H_{22}O_4$, Formula weight: 446.47, Temperature: 140(2)K, Crystal system: Monoclinic, Space group: P 21, Unit cell dimensions: a = 7.07170(10) Å, α = 90°, b = 8.84390(10) Å, β = 96.5560(10)°, c = 19.5443(3) Å, γ = 90°, Volume: 1214.33(3) $Å^3$, Z = 2, Density (calculated): 1.221 Mg/m^3, Absorption coefficient: 0.647 mm^{-1}, F(000): 468, Crystal size: 0.260 x 0.220 x 0.160 mm^3, Final R indices [I>2sigma(I)]: R1 = 0.0746, wR2 = 0.2230.

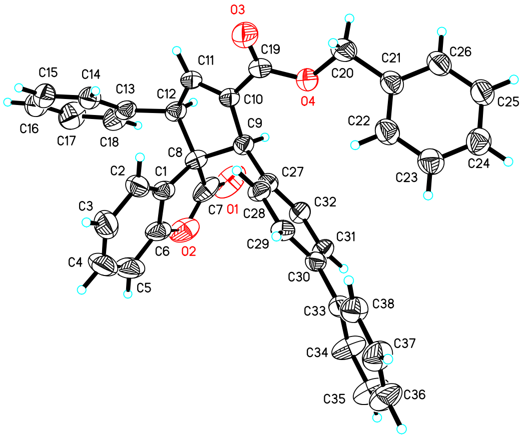

The crystal data of **5j** have been deposited in CCDC with number 967550. Empirical formula: $C_{38.50}H_{29}ClO_4$; Formula weight: 591.07; Temperature: 293(2)K; Wavelength: 0.71073 Å; Crystal system: Monoclinic; Space group: C2; Unit cell dimensions: a = 16.8074(19)Å, α = 90°. b = 10.9989(12)Å, β = 92.018(3)°. c = 16.649(2)Å, γ = 90°; Volume: 3075.8(6) $Å^3$, Z = 4. Density (calculated): 1.276 Mg/m^3, F(000): 1236; Crystal size: 0.213 x 0.156 x 0.123 mm^3. Final R indices [I>2sigma(I)], R1 = 0.0540, wR2 = 0.1364.

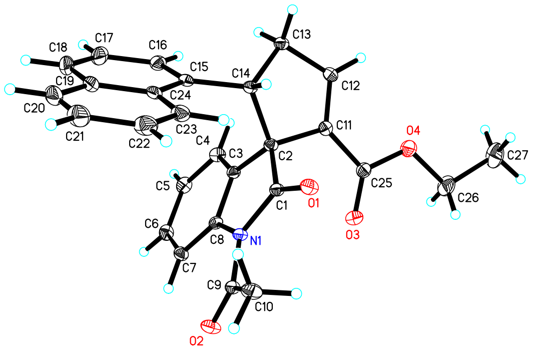

The crystal data of **7b** have been deposited in CCDC with number 1010550. Empirical formula: $C_{27}H_{23}NO_4$; Formula weight: 425.46; Temperature: 296(2)K; Wavelength: 1.54178 Å; Crystal system: Monoclinic; Space group: P 21; Unit cell dimensions: a = 8.16260(10)Å, α = 90°. b = 12.2302(2)Å, β = 101.4020(10)°. c = 11.07420(10)Å, γ = 90°; Volume: 1083.72(2)$Å^{3,}$ Z = 2. Density (calculated): 1.340 Mg/m^3, F(000): 448; Crystal size: 0.260 x 0.220 x 0.180 mm^3. Final R indices [I>2sigma(I)], R1 = 0.0365, wR2 = 0.0941.

9. Theoretical investigations and Computational Details

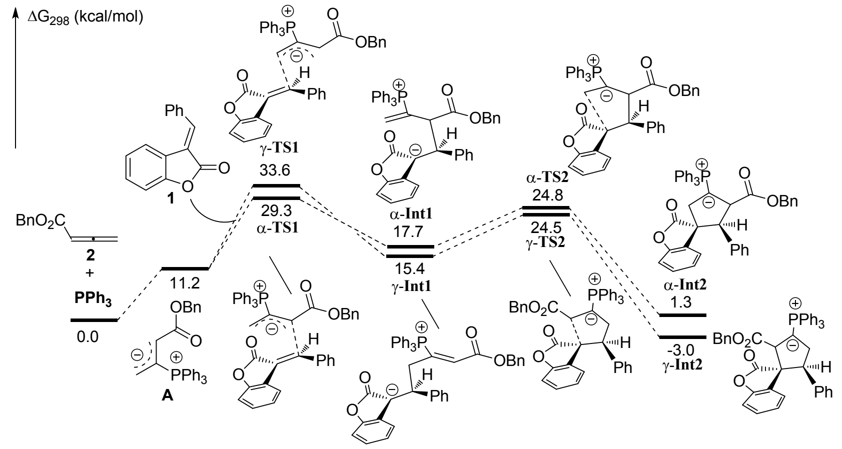

Scheme S1. Theoretical Investigations on Phosphine-catalyzed [3+2] Cycloaddition of **1** and **2**.

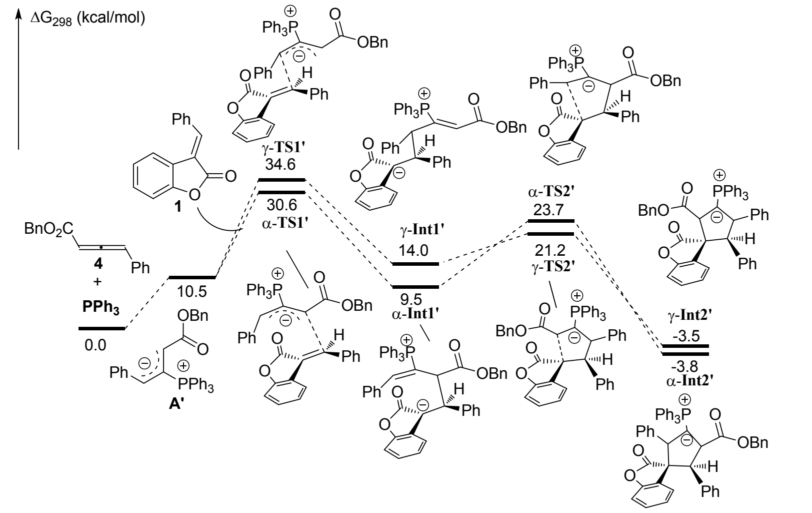

Scheme S2. Theoretical Investigations on Phosphine-catalyzed [3+2] Cycloaddition of **1** and **4**.

As shown in *Scheme S1*, for allenoate **2** (R^3 = H), the reactions starts from the formation of a zwitterionic intermediate **A** between **2** and PPh_3, which can undergo a Michael addition to generate a zwitterionic intermediate α-**INT1** (via α-addition) or γ-**INT1** (via γ-addition). Subsequently, they convert to α-**INT2** or γ-**INT2** via a ring-closure reaction, respectively. For both α-addition and γ-addition, the first addition step is rate-determing step. Although the energy barrier of γ-addition is higher than that of α-addition by 4.3 kcal/mol, the intermediates γ-**INT1** and γ-**INT2** are thermodynamically favorable, which may account for why γ-addition adducts were experimentally obtained as the major products. In the case of allenoate **4** (R^3 = Ph), probably due to the steric hindrance between the R^3 substituents and benzofuranone, the intermediates γ-**INT1'** and γ-**INT2'** are higher than α-**INT1'** and α-**INT2'** in energy by 4.5 kal/mol and 0.3 kcal/mol, respectively (*Scheme S2*). Meanwhile, the energy barrier of α-addition

is lower than that of γ-addition by 4.0 kcal/mol. Thus, the α-addition adduct is favorable with respect to the allenoate **4**.

The geometries of all systems have been optimized at mPW1K/6-31G(d) level. The subsequent frequency calculations on the stationary points were carried out at the same level of theory to ascertain the nature of the stationary points as minima or first-order saddle points on the respective potential energy surfaces. All transition states were characterized by one and only one imaginary frequency pertaining to the desired reaction coordinate. The intrinsic reaction coordinate (IRC) calculations were carried out at the same level of theory to further authenticate the transition states. The conformational space of flexible systems has first been searched manually. Thermochemical corrections to 298.15 K have been calculated for all minima from unscaled vibrational frequencies obtained at this same level. All quantum mechanical calculations have been performed with Gaussian 09

Table SI-2

	E_{tot}	H_{298}	G_{298}
**1**	-727.8550607	-727.626222	-727.67897
**2**	-575.4076453	-575.207783	-575.261672
**PPh_3**	-1036.114032	-1035.814568	-1035.87713
**A**	-1611.531608	-1611.029412	-1611.120916
α-**TS1**	-2339.384681	-2338.65202	-2338.771015
α-**INT1**	-2339.406339	-2338.670539	-2338.789511
α-**TS2**	-2339.396764	-2338.662151	-2338.778252
α-**INT2**	-2339.432607	-2338.695352	-2338.815648
γ-**TS1**	-2339.375829	-2338.643285	-2338.764143
γ-**INT1**	-2339.411206	-2338.675214	-2338.793225
γ-**TS2**	-2339.394999	-2338.660543	-2338.778693
γ-**INT2**	-2339.439185	-2338.702556	-2338.822507

Table SI-3

	E_{tot}	H_{298}	G_{298}
**4**	-806.406362	-806.117489	-806.183181
**A’**	-1842.531718	-1841.940477	-1842.043613
α-**TS1’**	-2570.366705	-2569.544802	-2569.673887
α-**INT1’**	-2570.400747	-2569.576442	-2569.707461
α-**TS2’**	-2570.382481	-2569.55967	-2569.684857
α-**INT2’**	-2570.424494	-2569.599365	-2569.728692
γ-**TS1’**	-2570.35728	-2569.536277	-2569.667425
γ-**INT1’**	-2570.394804	-2569.570867	-2569.700323
γ-**TS2’**	-2570.382621	-2569.559939	-2569.688867
γ-**INT2’**	-2570.423669	-2569.599095	-2569.72819

10. Archive Entries

1

1\1\GINC-SHI_02\FOpt\RmPWPW91\6-31G(d)\C15H10O2\YIN\04-Dec-2013\0\\#p
opt 6-31g(d) iop(3/76=0572004280) mpwpw91\\alkene_ewg\\0,1\C,0.0194509
298,0.1312167634,-0.0041738979\C,0.1601305241,0.0845590908,1.381602275
\C,1.4321238878,0.2051069217,1.9237508404\C,2.5102451115,0.3983206953,
1.0733132841\C,2.3307079711,0.467566868,-0.3020955686\C,1.070382055,0.
3267338731,-0.867118099\C,-2.0403218183,-0.2856388805,0.69284422\H,1.5
88871247,0.1378352521,2.9881693759\H,3.5024263122,0.4944874665,1.48763
96613\H,3.1835605891,0.6245187015,-0.945529195\H,0.9114405299,0.358399
4671,-1.933188408\O,-1.2668377342,-0.0649160101,-0.4110607377\O,-3.206
3089164,-0.5124191383,0.6123049062\C,-1.1748526317,-0.1713112399,1.898
361943\C,-1.743670589,-0.3471796376,3.1004605477\H,-2.786722819,-0.640
0647393,3.0619229852\C,-1.1701718038,-0.2042142904,4.4310340757\C,-1.6
180262301,-1.0416158131,5.4539239312\C,-0.2361205184,0.785418515,4.738
3630259\C,-1.107680093,-0.9286016369,6.7339452494\H,-2.3627647358,-1.7
933017494,5.2336975026\C,0.2605772657,0.9092479403,6.0232357001\H,0.06
96842962,1.4823102685,3.9727030341\C,-0.1642082346,0.0456912381,7.0215
853553\H,-1.4531012554,-1.5949006927,7.5104558735\H,0.9739470032,1.688
2346952,6.248820323\H,0.2274382114,0.1415074176,8.0234753582\\Version=
EM64L-G09RevA.01\State=1-A\HF=-727.8550607\RMSD=6.631e-09\RMSF=1.248e-
05\Dipole=1.4502832,0.1973858,1.2703417\Quadrupole=-3.7688601,-2.04657
84,5.8154385,2.8190198,-7.7762991,-2.63866\PG=C01 [X(C15H10O2)]\\@

2

1\1\GINC-SHI_02\FOpt\RmPWPW91\6-31G(d)\C11H10O2\YIN\03-Dec-2013\0\\#p
opt 6-31g(d) iop(3/76=0572004280) mpwpw91\\substrate1\\0,1\C,3.0566622
78,2.5154093149,-0.013019512\H,2.999385055,3.0769562964,0.908876402\H,
2.9988500368,3.0646870104,-0.9422425148\C,3.1841517331,1.2268574198,-0
.0045036055\C,3.3289790345,-0.0703529483,0.0040759144\H,4.3101976156,-
0.5220723502,0.006893609\C,2.2051572337,-1.0229888926,0.0107826556\O,2
.3517691344,-2.2149542802,0.0186163461\O,1.0173565883,-0.4228187488,0.
0072366704\C,-0.1037402512,-1.2866914955,0.0134011106\H,-0.0550350313,
-1.9391959231,-0.8576952037\H,-0.0540783116,-1.9278872115,0.8928073926
\C,-1.3627965366,-0.4727425104,0.0088431674\C,-2.5833855501,-1.1391756
866,0.0123917091\C,-1.3508757251,0.9139575885,0.0014527083\C,-3.772026
3642,-0.4321737768,0.0086763838\H,-2.6036258459,-2.2210692794,0.018110
1132\C,-2.5435385713,1.6221483929,-0.0023023813\H,-0.4072399657,1.4360
402983,-0.0013950266\C,-3.755672926,0.9546367415,0.0012922063\H,-4.712
4143591,-0.9638238704,0.0114953405\H,-2.5214415018,2.7023683286,-0.008
0723084\H,-4.6828247695,1.5087715825,-0.001635177\\Version=EM64L-G09Re
vA.01\State=1-A\HF=-575.4076453\RMSD=4.636e-09\RMSF=7.543e-06\Dipole=-
0.2243949,0.9825133,-0.0063217\Quadrupole=2.8436545,0.7548477,-3.59850
22,7.046806,-0.0464149,-0.0248211\PG=C01 [X(C11H10O2)]\\@

PPh_3

1\1\GINC-C0110\FOpt\RmPWPW91\6-31G(d)\C18H15P1\SHENGM\13-Jan-2014\0\\#
opt 6-31g(d) iop(3/76=0572004280) mpwpw91\\Title Card Required\\0,1\C
,-1.2231740853,-1.1040733492,-0.4136195707\C,-1.4408514781,-2.34484051
21,-1.0110693969\C,-1.9498626608,-0.7752433431,0.7272336515\C,-2.34486
30723,-3.2432335391,-0.4707444075\H,-0.8983783472,-2.605730365,-1.9095
365883\C,-2.8643394426,-1.6698843793,1.261411567\H,-1.8030783123,0.185
5742745,1.1982831136\C,-3.0610148839,-2.9058087224,0.6672424025\H,-2.4
98244556,-4.2021743239,-0.9441264402\H,-3.4233549213,-1.399417253,2.14
56808187\H,-3.7742575726,-3.6012287762,1.0853002517\C,1.5679883391,-0.
5054390835,-0.4147796454\C,2.7521613705,-0.0753278833,-1.011899665\C,1
.6451738061,-1.3015303306,0.7245594213\C,3.9815477449,-0.4136058053,-0
.4728380887\H,2.7079764214,0.5266164646,-1.9092569878\C,2.8764705487,-
1.6506069484,1.257535998\H,0.7389506525,-1.6535973282,1.1950136865\C,4
.0459502639,-1.2051160755,0.6634400314\H,4.8894214062,-0.068578592,-0.
9460279225\H,2.9205725627,-2.2718269831,2.1405531764\H,5.004398371,-1.
4786168625,1.0801924429\C,-0.3462807079,1.613616648,-0.4145940124\C,-1
.3136392296,2.4218954579,-1.0101270492\C,0.3053629484,2.0800581755,0.7
23691473\C,-1.637847289,3.6547509491,-0.4704532467\H,-1.8133380996,2.0
815350659,-1.9067750454\C,-0.0105013237,3.3200152178,1.2573231335\H,1.
0657963127,1.4734173644,1.1930447843\C,-0.9841895561,4.1076712835,0.66
49746701\H,-2.3929257293,4.2665715005,-0.9423879534\H,0.505902082,3.66
99442009,2.1396328114\H,-1.2285589046,5.0736279919,1.0825016989\P,-0.0
009036576,0.0015428613,-1.2160511127\\Version=EM64L-G09RevA.02\State=1
-A\HF=-1036.1140317\RMSD=3.427e-09\RMSF=2.861e-05\Dipole=-0.0001012,-0
.0012756,0.6223218\Quadrupole=1.759969,1.7673772,-3.5273462,-0.00534,-
0.0063715,0.0017909\PG=C01 [X(C18H15P1)]\\@

A

1\1\GINC-SHI_03\FOpt\RmPWPW91\6-31G(d)\C29H25O2P1\YIN\07-Dec-2013\0\\#
p opt 6-31g(d) iop(3/76=0572004280) mpwpw91\\pph3_s1_2\\0,1\C,0.105996
3688,0.1400842652,-0.0687301506\C,0.2060231033,-0.0773848153,1.3009394
647\C,1.4521978399,-0.2326117058,1.8810989036\C,2.599829761,-0.1480426
596,1.1073622335\C,2.5023494538,0.0682425638,-0.2570361022\C,1.2562981
801,0.1941261449,-0.849404729\H,-0.689741941,-0.1259569659,1.900474538
1\H,1.5268135936,-0.4098646641,2.9436513872\H,3.5707466766,-0.25778426
69,1.5679561321\H,3.393064804,0.1256697476,-0.8648905713\H,1.185228866
1,0.3198691541,-1.9203136528\P,-1.5165377327,0.2499913373,-0.857594942
7\C,-3.5091596884,-1.4777713158,-0.276026516\H,-3.1792733434,-2.045887
4329,-1.1262783733\H,-4.3634737828,-1.8575932678,0.266402362\C,-2.9264
492743,-0.3288417804,0.1376088374\C,-3.3407001913,0.4987958153,1.21382
32618\H,-4.2339044791,0.2443463737,1.7602825304\C,-2.6519257147,1.6674
830459,1.4891542038\O,-1.6153857337,2.0272895734,0.8988379116\O,-3.168
2379162,2.4378687886,2.4722518312\C,-2.471011933,3.62648535,2.74560609
67\H,-1.4582999671,3.3980035008,3.0797959567\H,-2.3706482126,4.2133266

953,1.8320930725\C,-3.205755007,4.4083146246,3.7945052406\C,-2.5985723
252,5.5319629719,4.3451881143\C,-4.4798722659,4.0595868371,4.217860839
9\C,-3.2521059393,6.295271182,5.2960651084\H,-1.6027018506,5.810981157
7,4.0261786899\C,-5.1343658616,4.8223295955,5.1735587591\H,-4.95047930
93,3.185610507,3.7966984676\C,-4.5260614116,5.9417304052,5.7149529846\
H,-2.7655268632,7.1649276856,5.7139653286\H,-6.125842104,4.5373622528,
5.4956170556\H,-5.0377591189,6.5342213594,6.4594793769\C,-1.3075179074
,-0.9562941797,-2.2183076572\C,-1.7123130539,-0.6808663373,-3.51990846
68\C,-0.7227934243,-2.1928718966,-1.9399603402\C,-1.5402813201,-1.6232
860318,-4.5226032108\H,-2.1593978283,0.2715159394,-3.758266808\C,-0.55
90958681,-3.1335614381,-2.940005631\H,-0.3971776149,-2.4207476276,-0.9
357324472\C,-0.9668667115,-2.8492591767,-4.2349640525\H,-1.8559677759,
-1.394036367,-5.5296409384\H,-0.109229941,-4.087807577,-2.7091234114\H
,-0.8340239576,-3.5822971725,-5.017146798\C,-1.8880675509,1.7945178367
,-1.7115889078\C,-3.2068552038,2.023339566,-2.0871674721\C,-0.90159065
18,2.7207386283,-2.0242811362\C,-3.5315905007,3.1759265553,-2.78528230
02\H,-3.9759623014,1.3113860987,-1.8239902658\C,-1.2339243676,3.866464
3433,-2.7242813604\H,0.1147236174,2.5637737702,-1.6969468553\C,-2.5470
587235,4.094644889,-3.1077137133\H,-4.5577099296,3.3564562187,-3.06945
18655\H,-0.4670380116,4.5891255929,-2.9609707248\H,-2.8030323788,4.993
3091604,-3.6494485216\\Version=EM64L-G09RevA.01\State=1-A\HF=-1611.531
6084\RMSD=6.394e-09\RMSF=1.817e-06\Dipole=1.3724696,-0.5644529,-1.8869
914\Quadrupole=0.2405193,-0.6243785,0.3838592,-3.6896529,2.6100689,1.7
363834\PG=C01 [X(C29H25O2P1)]\\@

α-**TS1**

1\1\GINC-SHI_02\FTS\RmPWPW91\6-31G(d)\C44H35O4P1\YIN\28-Feb-2014\0\\#p
 6-31g(d) iop(3/76=0572004280) mpwpw91 opt=(calcfc,ts,noeigen)\\pph3_s
1_alpha_ts1_new_2\\0,1\C,-2.0621547429,-1.6885204997,1.5875536868\C,-1
.2448876157,-1.2166225714,2.604919062\C,-1.4982107844,-1.5787666627,3.
915548437\C,-2.5538903395,-2.422231407,4.2198379985\C,-3.3735733523,-2
.895956093,3.2091881827\C,-3.1386712605,-2.5189678253,1.8982942977\H,-
0.4223330005,-0.5687938333,2.3729071432\H,-0.8663821284,-1.1955699492,
4.7025314784\H,-2.7427437075,-2.7038491431,5.2454670116\H,-4.204640817
5,-3.5462999527,3.4379389044\H,-3.8026518127,-2.8686256847,1.121122653
3\P,-1.8544454841,-1.2691307805,-0.1640728994\C,-1.5004417932,1.077655
704,-1.4672591765\H,-2.5659579779,1.097582151,-1.6136380548\C,-0.85154
5745,0.1191849416,-0.7699152289\C,0.5652136727,0.1839769357,-0.5689489
925\C,1.2289506263,-0.94511941,0.0028820422\O,0.6622963906,-1.82904953
31,0.6326680903\O,2.5507373946,-0.9605110315,-0.1844159694\C,3.2395314
782,-2.0516514486,0.3960377133\H,3.1471492311,-1.9913703747,1.48123176
73\H,2.769241764,-2.9838737204,0.0899859241\C,4.6774738401,-2.01032440
13,-0.0220607533\C,5.3007194088,-0.8183167325,-0.3652550943\C,5.417408
1864,-3.1862636907,-0.0255781115\C,6.6437678298,-0.8075680798,-0.70553
82864\H,4.7298681645,0.0969830451,-0.3716972568\C,6.7615527495,-3.1737
356363,-0.3578995834\H,4.9374337022,-4.1213863611,0.2310136742\C,7.379

355639,-1.9816231463,-0.7008529109\H,7.1164915961,0.1260975383,-0.9746
813001\H,7.3236824889,-4.0964292277,-0.3575884556\H,8.4264253936,-1.96
93297857,-0.96669285\C,-3.5686969275,-0.8995284392,-0.6437787508\C,-4.
2186538677,-1.5778799425,-1.668057069\C,-4.2458540349,0.0868609015,0.0
767122893\C,-5.5357478711,-1.2717146195,-1.9754530445\H,-3.7062504604,
-2.3476833031,-2.2243777569\C,-5.5563668921,0.3910086144,-0.2441308097
\H,-3.7403323733,0.6221336255,0.8709629297\C,-6.2031742026,-0.28768112
89,-1.2668505439\H,-6.0366038897,-1.8048640781,-2.7699758403\H,-6.0723
290566,1.1616882423,0.308749288\H,-7.2283170633,-0.0488186653,-1.50977
71729\C,-1.3716292193,-2.7343785131,-1.1097246041\C,-1.0731197106,-2.5
783741933,-2.4603859315\C,-1.3079830607,-3.9945999776,-0.52827668\C,-0
.7325381383,-3.6812870354,-3.2255675518\H,-1.0980826275,-1.5955374114,
-2.9089026189\C,-0.9669922842,-5.0927260802,-1.298876876\H,-1.50169532
71,-4.1142941133,0.526162615\C,-0.6826484529,-4.9390072235,-2.64651966
4\H,-0.499104806,-3.5544900603,-4.272232957\H,-0.91366442,-6.069503070
2,-0.8414793329\H,-0.4132247076,-5.7979129216,-3.2434542235\H,1.118517
7648,0.7090130529,-1.3307753349\C,0.7893454324,1.9120502649,0.82009424
27\H,0.5692893166,1.2805052348,1.6686674305\C,2.2216921018,2.220040594
7,0.6620167082\C,3.0582954785,2.0875024814,1.7688403848\C,2.786360853,
2.6481935286,-0.5376853556\C,4.4016284999,2.4135427047,1.6938649997\H,
2.6427607787,1.7426713379,2.7053640958\C,4.1298767081,2.9710807982,-0.
6178233061\H,2.1686287545,2.7192579622,-1.4206189585\C,4.9425892849,2.
8648790298,0.5009480514\H,5.0265595508,2.3136948282,2.5692759845\H,4.5
441579817,3.3034306174,-1.5587957733\H,5.9900322739,3.1215408969,0.440
1268728\C,-0.2524128798,2.7828189077,0.5232860888\C,-0.4055384214,4.03
65954323,-0.1876155539\C,-1.554013475,2.4826818655,1.0699831502\C,-1.7
539298905,4.3840588222,-0.0379610625\C,0.4129898068,4.9246358956,-0.87
70885577\C,-2.3142146328,5.5272236826,-0.5508655251\C,-0.1321511107,6.
0891637629,-1.4024178737\H,1.4670242657,4.7345117446,-0.9910279606\C,-
1.4778925879,6.388691718,-1.2511177626\H,-3.3614329061,5.7399955701,-0
.4013240436\H,0.5096685193,6.7751281023,-1.9359735701\H,-1.8794178606,
7.2995917728,-1.6698046755\O,-1.9225211872,1.5676609908,1.77427086\O,-
2.4378338422,3.4633503224,0.6899530177\H,-0.927797178,1.8520561374,-1.
9549824112\\Version=EM64L-G09RevA.01\State=1-A\HF=-2339.3846812\RMSD=2
.862e-09\RMSF=1.560e-06\Dipole=-0.5602204,-2.3229692,-0.8757026\Quadru
pole=7.8962686,-5.2897916,-2.606477,10.4113226,4.2838085,-3.973092\PG=
C01 [X(C44H35O4P1)]\\@

α-**INT1**

1\1\GINC-SHI_02\FOpt\RmPWPW91\6-31G(d)\C44H35O4P1\YIN\06-Mar-2014\0\\#
p opt 6-31g(d) iop(3/76=0572004280) mpwpw91\\pph3_s1_alpha_new_2\\0,1\
C,-0.2020472735,-0.3964255265,0.1597570367\C,0.0551381753,0.300865322,
1.3373234121\C,1.1676997735,-0.0217369286,2.0967777567\C,2.0315084541,
-1.0212906568,1.6824046074\C,1.7783121949,-1.7091837531,0.5066144812\C
,0.6629031749,-1.4061441652,-0.2516241893\H,-0.6160717278,1.0753111763
,1.6730600551\H,1.3535263854,0.5138041493,3.015790972\H,2.8996637331,-

1.2672245815,2.2762600309\H,2.4423525427,-2.4961883537,0.1823325545\H,
0.4611074632,-1.9660025614,-1.1487051788\P,-1.5933762159,0.0663062596,
-0.9003507457\C,-4.1681232832,-0.5945171441,-1.3258184121\H,-3.8670527
846,-0.8402985885,-2.3313204359\C,-3.3204782538,-0.18139325,-0.3861551
221\C,-3.8379969983,0.0688837711,1.013546785\C,-3.5429393528,1.4806167
869,1.4650090868\O,-2.5237054366,1.8577162844,1.9773105285\O,-4.562301
4571,2.2956817816,1.2028525521\C,-4.3879701766,3.6543542389,1.58677342
79\H,-4.4415231565,3.7076650024,2.6742877211\H,-3.3953146979,3.9844575
548,1.2925177346\C,-5.4520265386,4.4884993037,0.9433940271\C,-6.717574
0457,3.980586031,0.6785071625\C,-5.1809149332,5.8167997159,0.639008372
2\C,-7.6927575167,4.7892200485,0.1185707959\H,-6.9343489623,2.94748946
35,0.9034687822\C,-6.1587062057,6.6286278724,0.0878953024\H,-4.1966613
266,6.21987062,0.8363543205\C,-7.4181759732,6.1153159542,-0.1756858321
\H,-8.6718433025,4.3815216979,-0.086642707\H,-5.9341139951,7.660145427
3,-0.1412791132\H,-8.1811035976,6.7443092162,-0.6102655716\C,-1.363573
2217,-0.6367922081,-2.5577809286\C,-1.0059848115,0.2055918636,-3.60923
32997\C,-1.5336344957,-2.0038728925,-2.7902795064\C,-0.8171625756,-0.3
129250395,-4.8794993767\H,-0.8761878231,1.2636008446,-3.4471143011\C,-
1.3435538641,-2.5072344616,-4.0657216054\H,-1.7999574357,-2.6451047668
,-1.9576558164\C,-0.9863345887,-1.6676761927,-5.1094048077\H,-0.540605
033,0.3468943509,-5.6882566814\H,-1.4769419435,-3.5645472968,-4.239927
1449\H,-0.8408752034,-2.0691557585,-6.1017142338\C,-1.4291994386,1.865
6286507,-1.125541817\C,-2.5437290729,2.6282219905,-1.4671586168\C,-0.1
821353798,2.4809571364,-1.0400711673\C,-2.4153720535,3.9868736258,-1.7
03739494\H,-3.5161566844,2.1652973763,-1.5386481439\C,-0.0574893905,3.
8382826726,-1.2829340691\H,0.6907576346,1.9024735981,-0.7797274192\C,-
1.1726392911,4.592802409,-1.6111552576\H,-3.2899329952,4.5691974276,-1
.9527626066\H,0.912901001,4.3062884209,-1.2103978424\H,-1.0727254855,5
.6524776901,-1.7950737322\H,-4.9193570962,-0.0086331831,0.9215062354\C
,-3.3684740447,-1.0089708118,2.0264272746\H,-2.2791171777,-0.951838533
2,2.0547469037\C,-3.8552253621,-0.7443656404,3.4342449065\C,-3.1655848
023,-1.3452289286,4.484051071\C,-4.9659946161,0.031620668,3.7407074078
\C,-3.5667080558,-1.1726197106,5.795057441\H,-2.3111199803,-1.96702565
83,4.2567964212\C,-5.3715987005,0.2098743653,5.0570177767\H,-5.5415021
757,0.50209208,2.9563542975\C,-4.6741077174,-0.3897756691,6.0890715504
\H,-3.0154162098,-1.6515334982,6.591436996\H,-6.2399467626,0.817059466
4,5.2697388367\H,-4.9898781525,-0.2533497335,7.1130784678\C,-3.7292785
62,-2.3695069257,1.5345743734\C,-4.8222068045,-3.250403791,1.807950854
7\C,-2.9453136748,-2.9834601627,0.5712832506\C,-4.6099200997,-4.365698
2896,0.9783533501\C,-5.9444751481,-3.2659654592,2.6356097654\C,-5.4367
344715,-5.4605178861,0.9410009381\C,-6.7924965548,-4.3646277138,2.6058
623022\H,-6.1506963074,-2.4422569772,3.3018765984\C,-6.5515519039,-5.4
508244283,1.7736961901\H,-5.220332035,-6.2911182702,0.2858137652\H,-7.
6602743966,-4.3745185788,3.2508758022\H,-7.2278420024,-6.2930881627,1.
7728922951\O,-1.910388104,-2.6007762964,-0.0081033084\O,-3.4888183879,
-4.206296863,0.2331983248\H,-5.2128873035,-0.7284475236,-1.0855521484\

\Version=EM64L-G09RevA.01\State=1-A\HF=-2339.406339\RMSD=5.534e-09\RMSF=5.910e-06\Dipole=1.6140793,3.9180353,-2.3396552\Quadrupole=6.2727315,-12.8944373,6.6217059,-9.5906076,-1.3470471,3.0339596\PG=C01 [X(C44H35O4P1)]\\@

α-**TS2**

1\1\GINC-SHI_02\FTS\RmPWPW91\6-31G(d)\C44H35O4P1\YIN\24-Jan-2014\0\\#p opt=(calcfc,ts,noeigen) 6-31g(d) iop(3/76=0572004280) mpwpw91\\pph3_s1_alpha_ts2\\0,1\C,0.4928870109,2.4742758974,1.5182853801\C,1.5676694758,1.8688342486,2.1674913292\C,1.8522927045,2.2091311683,3.4798331399\C,1.0851605671,3.1550658234,4.1400497314\C,0.0373417219,3.7817932216,3.4833424124\C,-0.2569707719,3.4492263556,2.1725986169\H,2.2030241268,1.1662424136,1.639807918\H,2.6841184038,1.7365199193,3.9801178618\H,1.3115656339,3.4154950164,5.1636454583\H,-0.5498310264,4.5335858361,3.9895375375\H,-1.0648168499,3.952801701,1.6637466758\P,0.2561116734,2.0808576041,-0.2312115874\C,1.1671673941,-0.0759707895,-1.6852996129\H,1.743546419,0.5823081768,-2.3225772672\H,0.8670665768,-1.0049698765,-2.1438433973\C,0.5002153656,0.397733605,-0.5833826038\C,0.0297869158,-0.6730815556,0.3536010826\H,-0.3812624545,-0.2508205084,1.2691873441\C,-1.070466546,-1.4526328611,-0.3154880901\O,-0.9351775685,-2.2567816267,-1.1958687762\O,-2.2686727625,-1.0919991824,0.1604762861\C,-3.3878691681,-1.7171240842,-0.4503499255\H,-3.360773945,-1.5398695829,-1.5229458255\H,-3.310534636,-2.7936071888,-0.2986621646\C,-4.6364264021,-1.1622501154,0.1624029866\C,-5.7430661443,-0.895835125,-0.6319537066\C,-4.7191023156,-0.9302772588,1.5307763877\C,-6.9148055638,-0.40991411,-0.0727845613\H,-5.6874344622,-1.0651957259,-1.6984889221\C,-5.8849864121,-0.4360891642,2.0903971275\H,-3.8609446704,-1.1290332007,2.1553710533\C,-6.9877308684,-0.1750675376,1.290206674\H,-7.768212971,-0.2096162807,-0.7042032674\H,-5.935500695,-0.2588018106,3.1550326105\H,-7.8984431746,0.206580316,1.7282083502\C,1.3863122306,3.1628519978,-1.1383265018\C,0.9700714874,4.4081504722,-1.6057800854\C,2.7312537932,2.8048566162,-1.2225273316\C,1.8806897749,5.265617157,-2.1979705326\H,-0.059242872,4.7162828666,-1.5054059734\C,3.6322284062,3.6695050099,-1.8217658395\H,3.0869763981,1.8742055915,-0.7959186275\C,3.2099658654,4.8923164471,-2.3166209476\H,1.5500069698,6.2266904355,-2.5628217742\H,4.6705935662,3.3819844647,-1.8895461429\H,3.9186071043,5.5617330043,-2.7819176015\C,-1.4508228998,2.5042916814,-0.68769219\C,-1.7520170492,2.8191053394,-2.012771027\C,-2.4949723261,2.2830505436,0.2079664963\C,-3.0671967629,2.9688914186,-2.4140978828\H,-0.9603191512,2.9364098959,-2.7376299408\C,-3.8109002976,2.4239160184,-0.2000524535\H,-2.2891481951,1.9779609698,1.2225147062\C,-4.0982363061,2.7787267303,-1.5070871439\H,-3.2859470169,3.2232696559,-3.4405814116\H,-4.6099192625,2.2343992121,0.5005975525\H,-5.1250712398,2.8864998203,-1.823458519\C,1.302733266,-1.5465599576,0.7019895929\H,1.7989990395,-0.9328822884,1.4568320881\C,0.9774194554,-2.8518715039,1.4015991711\C,1.922353141,-3.8731365982,1.4689479336\C,-0.2170862362,-3.0323215003,2.0947438727\C,1.6749802,-5.0337229158,2.182

0945568\H,2.8625410934,-3.7572777206,0.9534341908\C,-0.4687931319,-4.1
937918311,2.8081815475\H,-0.9718404695,-2.2598802794,2.0945290048\C,0.
4754599234,-5.204004606,2.8533687339\H,2.4269325376,-5.8091654956,2.20
95644025\H,-1.4079409728,-4.3042201699,3.3315513463\H,0.2810028079,-6.
1110998357,3.4068954992\C,2.2904008934,-1.613630697,-0.4160544571\C,2.
6082517087,-2.6361719391,-1.380912594\C,3.4285593986,-0.7624268316,-0.
3617998285\C,3.8581156548,-2.2846756595,-1.9033292513\C,1.9617641607,-
3.7510649267,-1.8999348261\C,4.4943925772,-2.9950103359,-2.8909294364\
C,2.5933811032,-4.486535455,-2.895274243\H,0.9855615101,-4.031860412,-
1.5378895801\C,3.8397229277,-4.1191162839,-3.3852524418\H,5.4608123101
,-2.6871787654,-3.2598115766\H,2.1007393015,-5.3593172457,-3.299190014
7\H,4.3077891824,-4.7078734584,-4.1605433178\O,4.3437612905,-1.1604918
441,-1.3133477531\O,3.6577515295,0.2135454899,0.3384212957\\Version=EM
64L-G09RevA.01\State=1-A\HF=-2339.3967642\RMSD=9.419e-09\RMSF=2.195e-0
6\Dipole=-2.9418491,3.1353352,0.363363\Quadrupole=-4.2096483,1.3707657
,2.8388826,1.8001374,-0.8786664,-6.2281126\PG=C01 [X(C44H35O4P1)]\\@

α-**INT2**

1\1\GINC-SHI_03\FOpt\RmPWPW91\6-31G(d)\C44H35O4P1\YIN\21-Jan-2014\0\\#
p opt 6-31g(d) iop(3/76=0572004280) mpwpw91\\pph3_s1_alpha_step2\\0,1\
C,-0.1548444139,0.5107426776,-0.6673953247\C,-0.499194337,0.956871521,
0.6007960781\C,0.4872057498,1.2776329763,1.5233416251\C,1.8219124151,1
.1537886124,1.1814028979\C,2.1761814715,0.6902039933,-0.079315797\C,1.
1938897761,0.3612659994,-0.9956223974\H,-1.5409412548,1.0121362583,0.8
822165294\H,0.2060795135,1.6121913738,2.5109093321\H,2.59028951,1.4033
666043,1.8987502069\H,3.2180842248,0.5762531531,-0.3416814178\H,1.4763
445383,-0.0223294443,-1.9667529708\P,-1.5168040316,0.0677908774,-1.827
1373939\C,-3.82366588,-0.9502402785,-0.5678648969\H,-3.1514060682,-1.6
220936514,-0.034955231\H,-4.4623364065,-1.5761828782,-1.1934142944\C,-
3.1239392675,0.1169496487,-1.362486179\C,-3.9669002995,1.3597047776,-1
.3505578942\H,-3.4038921797,2.2863092553,-1.4432660903\C,-4.9994933867
,1.3869753281,-2.4535511933\O,-5.651550152,0.4520963278,-2.8462871639\
O,-5.1174664602,2.6081707185,-2.97228215\C,-6.1024604048,2.7749575615,
-3.981266577\H,-5.7902602226,2.2472886971,-4.8807522459\H,-7.029327560
5,2.3137302345,-3.6429733834\C,-6.2826932504,4.2376762241,-4.241331043
6\C,-6.3822786794,4.7120877022,-5.5412352286\C,-6.3973173071,5.1323881
543,-3.182891165\C,-6.6008535064,6.0590856847,-5.78624654\H,-6.2875199
413,4.0242872894,-6.3707337454\C,-6.6064181235,6.4780639666,-3.4268167
755\H,-6.3144813551,4.7696944124,-2.1689004853\C,-6.711631459,6.945396
4962,-4.7286227721\H,-6.6764922647,6.4148814383,-6.8035015375\H,-6.689
5627318,7.1651515336,-2.5972836561\H,-6.8763284938,7.9964107982,-4.916
3783415\C,-1.0367533216,-1.6320034353,-2.280040575\C,-1.2454602629,-2.
1158811505,-3.5676711399\C,-0.5295840446,-2.4885954813,-1.3066853085\C
,-0.9468926597,-3.431193156,-3.8776161944\H,-1.6342736432,-1.463677365
3,-4.3343883164\C,-0.2471456572,-3.8095611408,-1.6150304221\H,-0.35030
13195,-2.1234662703,-0.3060270227\C,-0.4516380853,-4.2811870887,-2.900

9581761\H,-1.1032113646,-3.7932177326,-4.8831369728\H,0.1379433898,-4.4672137311,-0.849704957\H,-0.2241946971,-5.3090613688,-3.1430303991\C,-1.2945153641,1.1713865974,-3.2568465951\C,-2.2379753135,1.0923345678,-4.2818256909\C,-0.260821836,2.0966041171,-3.3600525663\C,-2.1302810978,1.9024328595,-5.3985747404\H,-3.0653090924,0.4032629903,-4.1866562998\C,-0.162219509,2.9134325239,-4.4752347795\H,0.4604022304,2.1950489829,-2.5642483209\C,-1.090544878,2.8134933703,-5.497690035\H,-2.8644433646,1.8288975125,-6.1873546167\H,0.6397013957,3.6340297946,-4.5396772034\H,-1.0110511374,3.452122374,-6.3652100041\C,-4.6010149119,1.3157126704,0.0663696637\H,-3.7695111151,1.6303048246,0.6940618464\C,-5.7401687002,2.2471234188,0.3861639428\C,-6.9679663152,2.2244549137,-0.2728923109\C,-5.5569588858,3.1964000813,1.3883643964\C,-7.9686147464,3.1237779684,0.0563517639\H,-7.1542490377,1.4946178937,-1.0442120914\C,-6.5562543327,4.095281364,1.7235163132\H,-4.6139044718,3.2287933817,1.916794869\C,-7.7680758384,4.0634488669,1.0550009133\H,-8.912364537,3.0848624468,-0.4682122118\H,-6.3854650214,4.8190354368,2.5072385211\H,-8.5517046489,4.7614712008,1.3113267889\C,-4.7676617175,-0.1965867983,0.4269706555\C,-6.1321844058,-0.8100632081,0.5138511948\C,-4.2994216139,-0.35435545,1.869354178\C,-6.3299161717,-1.2256211224,1.8174513921\C,-7.0981479866,-1.1005245548,-0.4314557597\C,-7.4608697868,-1.8761654913,2.250304071\C,-8.2564482021,-1.753658757,-0.0270941132\H,-6.9432516896,-0.8287270577,-1.4650122216\C,-8.4382816441,-2.1281521866,1.2965557994\H,-7.5691291074,-2.1797482483,3.2795144433\H,-9.0220671412,-1.9779081982,-0.7549268418\H,-9.3454639603,-2.635027839,1.59096959\O,-5.2549397357,-0.9513379821,2.6217870911\O,-3.2619203493,-0.0077354075,2.3459275186\\Version=EM64L-G09RevA.01\State=1-A\HF=-2339.4326065\RMSD=5.692e-09\RMSF=3.018e-06\Dipole=0.968529,0.3504148,-1.5270859\Quadrupole=6.1302491,0.1054095,-6.2356585,-3.6419138,-1.3236801,1.8070096\PG=C01 [X(C44H35O4P1)]\\@

γ-TS1

1\1\GINC-SHI_03\FTS\RmPWPW91\6-31G(d)\C44H35O4P1\YIN\01-Mar-2014\0\\#p opt=(calcfc,ts,noeigen) 6-31g(d) iop(3/76=0572004280) mpwpw91\\pph3_s1_gamma_ts1_new\\0,1\C,0.147247059,-2.2558819584,1.610834431\C,0.5251160797,-1.1902243823,2.4202527575\C,0.5347344265,-1.343913472,3.7954346377\C,0.1836131091,-2.5572970174,4.3653806461\C,-0.192706194,-3.620901068,3.5610646168\C,-0.2241995071,-3.4695678928,2.1856564557\H,0.8163240685,-0.2539041464,1.9736362363\H,0.8233085681,-0.5139728433,4.4229416832\H,0.1962995931,-2.6724629988,5.439185002\H,-0.4768577715,-4.5641482058,4.0027590057\H,-0.552268327,-4.2900295957,1.5644054447\P,0.0729343152,-2.0722620674,-0.1816710233\C,-1.3230251453,0.1289067011,-1.0714987564\H,-2.1458143386,-0.5579885097,-1.1752462955\C,-0.0418209842,-0.3498298407,-0.7659510779\C,1.1181802375,0.3939256113,-0.9035634864\H,1.0641404703,1.3365120824,-1.4239474509\C,2.3677721432,0.0304358555,-0.3312800042\O,2.5624059962,-0.9336847972,0.3973204972\O,3.3574978435,0.8744969622,-0.6624039697\C,4.6221233436,0.6048546131,-0.0932001864\H,4.

489632892,0.3184452362,0.9495371936\H,5.0768203432,-0.245632325,-0.600
5824676\C,5.4843564476,1.8241538537,-0.2137626493\C,6.8549123542,1.688
4485586,-0.3882917421\C,4.9413455807,3.098471936,-0.1038190421\C,7.674
4008445,2.8044108344,-0.4420667586\H,7.2862960979,0.701266121,-0.48632
86595\C,5.7585204654,4.2144299031,-0.1652544727\H,3.874136336,3.208585
5298,0.0177299524\C,7.1274490576,4.0719783878,-0.3313803407\H,8.739033
4193,2.6827237341,-0.5797739387\H,5.3247024518,5.2007857207,-0.0840775
689\H,7.7630472472,4.9441217749,-0.3795467819\C,-1.4297125426,-2.96313
38471,-0.660519363\C,-1.4288580488,-3.8726590577,-1.7167906581\C,-2.60
95268262,-2.7273642627,0.0431228101\C,-2.5971171855,-4.5288841705,-2.0
645253382\H,-0.5220068211,-4.0758730013,-2.2640172338\C,-3.778254417,-
3.370906521,-0.3243803381\H,-2.650332681,-2.0200185018,0.8562032206\C,
-3.7715559608,-4.2760200966,-1.3732007139\H,-2.5877852248,-5.236099091
,-2.8806030474\H,-4.6861828859,-3.1389812661,0.2099248816\H,-4.6829427
496,-4.782589315,-1.6554885699\C,1.4088541578,-2.933163215,-1.03667421
73\C,1.6537493567,-2.6366367002,-2.3736497206\C,2.1354301393,-3.930165
3526,-0.3990983719\C,2.6236893066,-3.341834403,-3.0670936651\H,1.10106
95789,-1.8511547761,-2.86723811\C,3.1004934594,-4.6318098306,-1.099735
3472\H,1.9674924003,-4.1413266349,0.645862699\C,3.3448909386,-4.340023
0073,-2.4324654858\H,2.8167984891,-3.1059368839,-4.1029552392\H,3.6696
79353,-5.4012333474,-0.5997542476\H,4.1016170925,-4.8873094613,-2.9749
786046\C,-2.1266581481,1.2850787302,0.5492989713\H,-2.0256359949,0.476
5682089,1.2613517565\C,-1.0828647238,2.3182259525,0.6898357048\C,-0.40
40040557,2.408996124,1.9037753406\C,-0.7361830935,3.2141510318,-0.3192
681041\C,0.5596600833,3.3807731425,2.1169129948\H,-0.6601820931,1.7244
022497,2.7002512909\C,0.2309717611,4.182438743,-0.1131515365\H,-1.2159
641729,3.1432217506,-1.2836799047\C,0.8793199377,4.2750883991,1.108949
171\H,1.0597163538,3.4395950455,3.0727811061\H,0.4813307971,4.86559431
05,-0.9118320217\H,1.6289467625,5.0355233634,1.2720720764\C,-3.4613571
105,1.5323749169,0.2352620576\C,-4.2088543329,2.5917082883,-0.39953632
97\C,-4.4315203887,0.5149752254,0.5756859585\C,-5.5396274661,2.1530342
634,-0.4071179231\C,-3.9500544673,3.8589246638,-0.9145464584\C,-6.5870
263206,2.8816302096,-0.9109620624\C,-4.9951352821,4.6149312787,-1.4278
106209\H,-2.9559525066,4.2724652751,-0.9011360265\C,-6.2970567264,4.13
59473487,-1.4358897083\H,-7.5909463589,2.4869921951,-0.8883377864\H,-4
.7866771621,5.5975109492,-1.8256274862\H,-7.0930365692,4.740917726,-1.
8440421214\O,-5.6722446297,0.9259995649,0.1611790402\O,-4.296714061,-0
.5499077283,1.1323421988\H,-1.3680694834,0.9807312197,-1.7335478037\\V
ersion=EM64L-G09RevA.01\State=1-A\HF=-2339.375829\RMSD=8.934e-09\RMSF=
1.433e-06\Dipole=2.3234883,-2.0282377,-0.2785968\Quadrupole=-20.432798
3,16.5349437,3.8978546,3.7025517,4.7851573,1.4686706\PG=C01 [X(C44H35O
4P1)]\\@

γ-INT1

1\1\GINC-SHI_03\FOpt\RmPWPW91\6-31G(d)\C44H35O4P1\YIN\24-Jan-2014\0\\#
p opt 6-31g(d) iop(3/76=0572004280) mpwpw91\\pph3_s1_gamma\\0,1\C,-0.6

587497575,0.7959127057,0.5166942312\C,-1.0817789584,1.0759671551,1.809
126961\C,-0.1414887006,1.3615779776,2.7851488787\C,1.2062605789,1.3939
041373,2.4729743673\C,1.6297229416,1.1173563914,1.1813539164\C,0.70257
65913,0.8016227571,0.206023667\H,-2.126815437,1.0608702215,2.068935140
6\H,-0.4771490549,1.5480936648,3.7933078729\H,1.9325189111,1.621250556
5,3.2394789177\H,2.6814883181,1.1269756848,0.9374585057\H,1.0408384113
,0.5461223051,-0.7880279005\P,-1.801382402,0.3448388575,-0.8028993934\
C,-3.9641544324,-1.5565965593,-0.5513350393\H,-3.1571773933,-2.1796938
898,-0.9251764491\C,-3.489189428,-0.1313622891,-0.3351508268\C,-4.3546
157244,0.7654775458,0.1547764045\H,-5.3649286794,0.46508122,0.37747073
05\C,-4.0331251359,2.1617214918,0.4574007565\O,-2.941384186,2.65032715
54,0.2756090714\O,-5.0795552128,2.80731296,0.9339780675\C,-4.882124616
,4.121904805,1.4446010765\H,-3.8140855754,4.324617769,1.4652311744\H,-
5.346726937,4.8161504376,0.747352714\C,-5.4970321295,4.2350251936,2.80
66029212\C,-6.1112095539,5.4226090116,3.1803822162\C,-5.4185092392,3.1
856652704,3.7158168647\C,-6.6338337429,5.5737582608,4.4544498378\H,-6.
1846350315,6.2361041347,2.4705788142\C,-5.9515057618,3.3384499061,4.98
53232564\H,-4.9590464634,2.2465670898,3.4358654343\C,-6.5552586442,4.5
289260066,5.3601741047\H,-7.1102247358,6.5023239712,4.7339921973\H,-5.
8928967318,2.5169600192,5.6843108953\H,-6.968268822,4.639577317,6.3524
122911\C,-1.0165774383,-1.1102121952,-1.5442812343\C,-0.800738372,-1.2
244353359,-2.9134198457\C,-0.6110624742,-2.136341954,-0.6899074367\C,-
0.1891910593,-2.359285535,-3.4235731439\H,-1.0961032804,-0.4315179917,
-3.5831269965\C,-0.0100894789,-3.2683442853,-1.207783149\H,-0.77711217
01,-2.0662802177,0.3759451627\C,0.2031535981,-3.3798085577,-2.57408484
75\H,-0.0188216644,-2.4410995279,-4.4867491141\H,0.2863329287,-4.05886
63224,-0.5353493884\H,0.6774263534,-4.2631896241,-2.9758137824\C,-1.86
18074779,1.5966665933,-2.1064932228\C,-2.8256623875,1.4763960005,-3.10
36279283\C,-0.953295662,2.6468099544,-2.1439697362\C,-2.8667538537,2.3
932316265,-4.1403010938\H,-3.5507305808,0.6758361873,-3.06800525\C,-1.
0002829034,3.5601628482,-3.1829489819\H,-0.229931707,2.7656643363,-1.3
524702036\C,-1.9519721472,3.4332608555,-4.182042485\H,-3.6175984462,2.
2974226189,-4.9103137888\H,-0.2975824764,4.3796162715,-3.2044863904\H,
-1.9870280342,4.1503249119,-4.9887976963\C,-4.4787750975,-2.1356034425
,0.7674630078\H,-5.3606114703,-1.5545055845,1.0510297606\C,-4.94918596
28,-3.5715714403,0.6567744592\C,-4.9590042003,-4.2859412066,-0.5347375
032\C,-5.420412909,-4.2067282894,1.8047101111\C,-5.425546329,-5.593411
0318,-0.5815048008\H,-4.6009441335,-3.8319206453,-1.4468518729\C,-5.88
92414505,-5.5055528081,1.7614765554\H,-5.401660311,-3.6679709086,2.741
3685047\C,-5.8937464219,-6.2082804193,0.5644517108\H,-5.4207393272,-6.
1278801258,-1.5209312635\H,-6.2478636877,-5.9749478107,2.6662453597\H,
-6.2562786497,-7.2253856779,0.5297219966\C,-3.4817459685,-1.9110820681
,1.8675409473\C,-2.4349340623,-2.7489222917,2.3574876521\C,-3.54337833
36,-0.7959488701,2.7076421358\C,-1.8901740045,-2.0680271561,3.46005028
62\C,-1.8838511465,-3.9845192249,2.0149535892\C,-0.8444225345,-2.54858
67204,4.2082479059\C,-0.8233943944,-4.4816404859,2.7629529486\H,-2.286

91022,-4.5570842156,1.1925084603\C,-0.3031599197,-3.7786182032,3.84256
77899\H,-0.4660627487,-1.9881508515,5.0499827044\H,-0.4028526062,-5.44
40941799,2.5054848106\H,0.5198344614,-4.1893683205,4.4087417759\O,-2.5
395017501,-0.9019178668,3.6704818101\O,-4.2508967043,0.216729303,2.700
899789\H,-4.7427228127,-1.5543275917,-1.3204856609\\Version=EM64L-G09R
evA.01\State=1-A\HF=-2339.4112057\RMSD=3.531e-09\RMSF=3.892e-06\Dipole
=1.8528243,1.3756717,-3.7129786\Quadrupole=-3.8746706,-2.1993733,6.074
0439,-0.4420837,-1.6751142,1.763467\PG=C01 [X(C44H35O4P1)]\\@

γ-TS2

1\1\GINC-SHI_03\FTS\RmPWPW91\6-31G(d)\C44H35O4P1\YIN\24-Jan-2014\0\\#p
opt=(calcfc,ts,noeigen) 6-31g(d) iop(3/76=0572004280) mpwpw91\\pph3_s
1_gamma_ts2\\0,1\C,-1.806081851,-1.6662320011,1.6699947435\C,-1.013449
9133,-0.9768199269,2.577461202\C,-1.243611052,-1.112242139,3.937535359
7\C,-2.2543603697,-1.9386626447,4.3966494055\C,-3.0502320256,-2.629918
5799,3.4946449324\C,-2.8330791167,-2.4903701837,2.135859928\H,-0.22185
32685,-0.3376552213,2.227566736\H,-0.6279234591,-0.561114973,4.6315775
25\H,-2.4282768801,-2.0423362497,5.4578001537\H,-3.8447806428,-3.27051
91949,3.8477575411\H,-3.4676711417,-3.0192638504,1.4392321489\P,-1.540
388486,-1.5296519384,-0.1259211882\C,-1.466728935,1.0097040977,-1.4166
969251\H,-2.3809007271,1.1791650158,-0.8496535353\C,-0.7216774373,-0.1
836017735,-0.8872371284\C,0.645971499,-0.0156319507,-0.9180785893\H,1.
1200411313,0.4972718654,-1.7431508491\C,1.5701595257,-0.8454164071,-0.
130818446\O,1.2298872206,-1.5839602548,0.764937417\O,2.8213271399,-0.6
705943357,-0.5079738692\C,3.8233233085,-1.30505012,0.2725197791\H,3.68
83503984,-1.0038236554,1.3111453645\H,3.6849399309,-2.3837448017,0.226
0397212\C,5.1672686684,-0.8988537368,-0.2487333274\C,6.2425436163,-1.7
646351939,-0.0972282443\C,5.3657614027,0.3431423864,-0.8405756499\C,7.
5085493199,-1.3954062184,-0.5202773076\H,6.0899892053,-2.7366200334,0.
3532812198\C,6.6323073841,0.704601462,-1.2708913959\H,4.5338938403,1.0
195903907,-0.9779223304\C,7.7054600589,-0.1574571142,-1.1102663942\H,8
.3370909761,-2.0779240434,-0.3974319285\H,6.7771486848,1.6692974654,-1
.7348799008\H,8.6902660069,0.1318906485,-1.4476557997\C,-3.2405603572,
-1.4767669398,-0.7743256538\C,-3.6060733044,-2.198554361,-1.9051481877
\C,-4.1812776966,-0.6537101506,-0.1566626211\C,-4.8907766072,-2.094243
3741,-2.4139368996\H,-2.8936562758,-2.8512586628,-2.3861306835\C,-5.46
08066045,-0.5463944186,-0.6721249081\H,-3.9169289744,-0.1021917902,0.7
339950941\C,-5.8171179063,-1.2670063735,-1.8016522788\H,-5.1662785092,
-2.6636148516,-3.2891526319\H,-6.1802805535,0.0970375121,-0.1881068122
\H,-6.8175529577,-1.1865817513,-2.2005216775\C,-0.811615083,-3.0893522
532,-0.6972470004\C,-0.3236443401,-3.1651644473,-1.9996680659\C,-0.751
7163993,-4.2093746044,0.1238352667\C,0.2031073252,-4.3533647894,-2.477
091278\H,-0.341121345,-2.2893204829,-2.6314330459\C,-0.222760623,-5.39
45034449,-0.3584722693\H,-1.0944869027,-4.1526955984,1.144878165\C,0.2
518049415,-5.4697485449,-1.657751695\H,0.583656457,-4.4021596189,-3.48
6471886\H,-0.1714954965,-6.2576150563,0.2884299805\H,0.6681902022,-6.3

946327826,-2.0288752432\C,-0.547829895,2.2755885419,-1.3526895635\H,0.
0313954601,2.2898192127,-2.2801851138\C,-1.367186512,3.5565648798,-1.3
505985258\C,-2.6254768876,3.6160760732,-1.9452059904\C,-0.8504134922,4
.730528475,-0.8097811731\C,-3.3445570167,4.8002147458,-1.9893204281\H,
-3.0579221029,2.7328018648,-2.3914087006\C,-1.5663892539,5.9148195029,
-0.850577728\H,0.1252762794,4.7135061517,-0.3488106648\C,-2.8198326619
,5.9564590928,-1.4387361516\H,-4.318472736,4.8153536298,-2.4577399964\
H,-1.1399785695,6.8086833821,-0.4185807401\H,-3.3793481143,6.880090016
7,-1.4697813673\C,0.4720702605,2.1828564442,-0.2664481011\C,0.25890399
33,2.3103602688,1.1404045748\C,1.8742079824,2.3880207976,-0.4787947035
\C,1.5296380785,2.4627638738,1.7142260049\C,-0.8561909317,2.328310121,
1.9684612577\C,1.7317877974,2.6004661904,3.0634533543\C,-0.6710095363,
2.474758531,3.3393339708\H,-1.8511860117,2.257894874,1.5543516735\C,0.
6007367478,2.5988062517,3.8807457113\H,2.7256158327,2.7207220575,3.466
3488697\H,-1.5317882306,2.4974891983,3.9919324478\H,0.7205490225,2.716
5196735,4.9478851087\O,2.4947874666,2.4827929774,0.765040023\O,2.53318
60085,2.4046228035,-1.4923648245\H,-1.7814169792,0.8764953628,-2.45330
12602\\Version=EM64L-G09RevA.01\State=1-A\HF=-2339.3949988\RMSD=4.395e
-09\RMSF=1.301e-06\Dipole=-3.0387493,-3.2415449,0.4206772\Quadrupole=3
.7533257,-2.8561876,-0.8971381,-3.579083,5.1371641,6.2550864\PG=C01 [X
(C44H35O4P1)]\\@

γ-INT2

1\1\GINC-SHI_03\FOpt\RmPWPW91\6-31G(d)\C44H35O4P1\YIN\21-Jan-2014\0\\#
p opt 6-31g(d) iop(3/76=0572004280) mpwpw91\\pph3_s1_gamma_step2\\0,1\
C,0.522799323,0.9598453178,-0.8539635034\C,0.6926313535,1.7327375238,0
.2868594226\C,1.9178619512,2.3217794372,0.5582741343\C,2.9822765252,2.
1435482922,-0.3096499011\C,2.8219937523,1.3741644534,-1.4527606235\C,1
.6001535563,0.7811007869,-1.7209393134\H,-0.1492906515,1.8859029435,0.
9433172622\H,2.0389598522,2.9232685088,1.4474596491\H,3.9375422891,2.6
01258797,-0.0966569891\H,3.6507898809,1.2300706655,-2.1306768271\H,1.4
869190634,0.1694891275,-2.6052514559\P,-1.1035111589,0.1523630962,-1.1
727399424\C,-2.2153945161,-1.3105406115,0.9603397654\H,-1.4715704164,-
1.2671613832,1.7640385768\C,-2.2186563066,-0.1018316049,0.052634444\C,
-3.3899664734,0.7886878409,0.3845707738\H,-4.2287773003,0.6813053422,-
0.3165625135\C,-3.1601754616,2.2756545403,0.4501221106\O,-2.1591001087
,2.8592751156,0.1356163033\O,-4.2612363269,2.9006556636,0.8662673717\C
,-4.1929006533,4.3149663878,0.9332716713\H,-3.3863155571,4.6010409936,
1.6058396966\H,-3.9373190821,4.6963094084,-0.0552266917\C,-5.507632457
,4.8596487282,1.4027340819\C,-5.5570372865,6.1688164239,1.8679067359\C
,-6.6767265879,4.1132283515,1.3572441911\C,-6.7555227539,6.7281409167,
2.2742880911\H,-4.6488254921,6.755163745,1.9153847816\C,-7.8756799375,
4.6725108607,1.772231839\H,-6.6505584024,3.092306811,1.0099636239\C,-7
.9211030696,5.9789257951,2.2282415593\H,-6.7779317989,7.7465480125,2.6
343142642\H,-8.7770066833,4.07804215,1.7397623052\H,-8.8572974529,6.41
05945497,2.5509674955\C,-0.541621967,-1.455836066,-1.826939555\C,-1.03

27390415,-1.9775161806,-3.0186834035\C,0.3512115156,-2.2143855537,-1.0
726419757\C,-0.6391396174,-3.2353248935,-3.4468708728\H,-1.7172656163,
-1.3996213203,-3.6210327741\C,0.7316383401,-3.4769193863,-1.4946654513
\H,0.7583510839,-1.8133262206,-0.1554217409\C,0.2383452243,-3.98859617
9,-2.684386267\H,-1.019761213,-3.6257178352,-4.3793320685\H,1.41818667
55,-4.0580587966,-0.8966938336\H,0.5403484861,-4.9705529408,-3.0180355
087\C,-1.843507323,1.098035592,-2.5336728034\C,-3.1185442177,0.7309758
554,-2.9603338077\C,-1.2010842895,2.168048808,-3.1459122496\C,-3.73413
71448,1.4145118469,-3.9951615594\H,-3.6238216651,-0.0903736082,-2.4718
595115\C,-1.8201201417,2.8509270623,-4.1799884099\H,-0.2251906668,2.47
71260404,-2.805366267\C,-3.0834568424,2.4748315564,-4.6066466334\H,-4.
7229841226,1.1239134938,-4.31787322\H,-1.3163483753,3.6839889717,-4.64
76692772\H,-3.5645396626,3.0121863402,-5.4108888987\C,-3.6234650037,-1
.2889792769,1.5510288953\H,-4.3096736863,-1.5929826713,0.7584435518\C,
-3.905477121,-2.1502872988,2.751010144\C,-2.9919367104,-2.3308201669,3
.7853547911\C,-5.1400857349,-2.787548914,2.8465325847\C,-3.3024375216,
-3.1255981543,4.8763509274\H,-2.0288367786,-1.8460401069,3.7455311682\
C,-5.4556255116,-3.57924154,3.9381651233\H,-5.8640590198,-2.6540677754
,2.0544752007\C,-4.5347739769,-3.7532072598,4.9577309081\H,-2.57714246
77,-3.2529455289,5.6670830692\H,-6.4207374599,-4.0621512283,3.98860210
16\H,-4.7748884423,-4.3731018754,5.8093247743\C,-3.8997641431,0.227905
8061,1.7313001368\C,-3.3145858806,0.8516659508,2.9570660502\C,-5.38282
57815,0.4714006447,1.9529020674\C,-4.368330978,1.28912823,3.7405608843
\C,-2.0200493548,1.0660369061,3.3886007601\C,-4.2020144501,1.915318729
8,4.9520947369\C,-1.8176114628,1.6976862159,4.6111966952\H,-1.18161552
72,0.7556939276,2.7837646287\C,-2.894671135,2.1100975201,5.3815539973\
H,-5.0511628501,2.2432015962,5.5301503066\H,-0.8114299876,1.8703613883
,4.962980646\H,-2.7186897814,2.5984712892,6.3286300298\O,-5.5847260831
,1.0481100969,3.1639619669\O,-6.2844320327,0.1945395517,1.2256204648\H
,-2.0460266799,-2.255586526,0.442203421\\Version=EM64L-G09RevA.01\Stat
e=1-A\HF=-2339.4391845\RMSD=7.404e-09\RMSF=2.941e-06\Dipole=1.7490601,
0.1649704,-0.795677\Quadrupole=-5.7835754,-1.2584907,7.0420661,-3.1929
785,1.3176019,5.1027066\PG=C01 [X(C44H35O4P1)]\\@

4

1\1\GINC-SHI_03\FOpt\RmPWPW91\6-31G(d)\C17H14O2\YIN\02-Dec-2013\0\\#p
opt 6-31g(d) iop(3/76=0572004280) mpwpw91\\Title Card Required\\0,1\C,
0.1014977846,-0.0501964441,-0.0931287659\H,0.2063772374,-0.1067669979,
0.9843871593\C,1.1972566555,-0.0062504591,-0.7959595676\C,2.2959827974
,0.0308205013,-1.4978168666\H,2.773406469,-0.8742916631,-1.847455142\C
,2.9876044327,1.2773879507,-1.8804214732\O,3.995115757,1.2963928302,-2
.5340207024\O,2.3762828872,2.3688212847,-1.4279918385\C,2.9816606276,3
.6002407001,-1.7835470609\H,3.9874317255,3.6399882826,-1.3688940038\H,
3.0826614471,3.6405204242,-2.8680571581\C,2.139523279,4.729783252,-1.2
745689406\C,2.7229916193,5.9782833288,-1.097289482\C,0.784233437,4.574
2755342,-1.0174910481\C,1.9642433206,7.0579325513,-0.6792878328\H,3.78
04536226,6.1071936415,-1.2858830788\C,0.0265901446,5.6536027658,-0.591

4809035\H,0.3273490541,3.6045656431,-1.140597263\C,0.6114696279,6.8979
052505,-0.4240584982\H,2.4318527362,8.0224485101,-0.5449961942\H,-1.02
62243364,5.5191414692,-0.3897899772\H,0.0184293945,7.7374519061,-0.092
0524341\C,-1.2662414618,-0.0232016176,-0.6204453738\C,-2.3356836549,-0
.1066933312,0.2663760685\C,-1.5311432662,0.0842278077,-1.9851249024\C,
-3.6404188257,-0.0857370609,-0.1966879184\H,-2.141634809,-0.1893637162
,1.3266880942\C,-2.8331421585,0.1054247791,-2.4455936921\H,-0.70819107
08,0.15131116,-2.6821248271\C,-3.8935325505,0.0201104381,-1.5537451731
\H,-4.459018003,-0.1521683057,0.5048261356\H,-3.0237405034,0.189193695
2,-3.5054995681\H,-4.9104395872,0.0368334937,-1.9170026242\\Version=EM
64L-G09RevA.01\State=1-A\HF=-806.406362\RMSD=5.858e-09\RMSF=1.527e-06\
Dipole=-1.0374182,0.1341356,0.4784441\Quadrupole=-0.7102628,1.7951566,
-1.0848938,5.0386624,2.0956365,-1.2851146\PG=C01 [X(C17H14O2)]\\@

A′

1\1\GINC-SHI_03\FOpt\RmPWPW91\6-31G(d)\C35H29O2P1\YIN\09-Dec-2013\0\\#
p opt 6-31g(d) iop(3/76=0572004280) mpwpw91\\pph3_s2_2\\0,1\C,-0.06474
94766,-0.1172136001,0.0182139552\C,-0.0526814388,-0.1857108386,1.40674
47092\C,1.1515477815,-0.2984285069,2.0778835418\C,2.3454821856,-0.3199
541012,1.3731059938\C,2.3362338904,-0.2509192947,-0.0101053317\C,1.132
0282068,-0.1669428421,-0.6897984379\H,-0.9833383971,-0.1482661456,1.95
09613256\H,1.1572173153,-0.3598344252,3.1558816167\H,3.2836420547,-0.3
971212136,1.902908479\H,3.2632820888,-0.2759280979,-0.5633405854\H,1.1
295513742,-0.1558273886,-1.7703170998\P,-1.6362235251,-0.0648159709,-0
.8726212995\C,-3.6000517099,-1.7393728514,-0.1008923102\H,-3.190204491
4,-2.3160918983,-0.9130209489\C,-3.0983875268,-0.4872395196,0.12957340
81\C,-3.6075399703,0.5298249073,0.9704770579\H,-4.596028344,0.43473477
42,1.3838391738\C,-2.9009514303,1.7146769561,1.1602172732\O,-1.7673716
449,1.9399483003,0.7109290285\O,-3.5410497561,2.6525819933,1.890211144
4\C,-2.8454163036,3.8609748407,2.0724160573\H,-1.9038113249,3.67529326
09,2.5894894835\H,-2.5878774347,4.2849220481,1.1011275234\C,-3.6976740
689,4.8159535255,2.8545467477\C,-3.1139003442,5.961604782,3.3840304399
\C,-5.0566584359,4.6078734026,3.0413900528\C,-3.8726247193,6.885167216
2,4.0810865147\H,-2.0533572031,6.130894124,3.2507028669\C,-5.816839812
4,5.5306786343,3.7437601317\H,-5.5110582847,3.7160631553,2.6398471805\
C,-5.2304495735,6.6719011627,4.2640708232\H,-3.4027127305,7.7694692728
,4.487064504\H,-6.8735735783,5.353866419,3.8847597009\H,-5.8246931185,
7.3890518069,4.8115313902\C,-1.3891883215,-1.3968987811,-2.0986772525\
C,-1.7761150959,-1.2500248436,-3.4269902638\C,-0.8027463411,-2.5972026
261,-1.6933157314\C,-1.5857622528,-2.2836844575,-4.3307126861\H,-2.222
4345032,-0.3270457449,-3.7626369671\C,-0.6214294717,-3.6292016393,-2.5
95901835\H,-0.4915295459,-2.7259353956,-0.6674495304\C,-1.0117988758,-
3.4734592861,-3.9174399976\H,-1.8869215547,-2.1543120186,-5.3596896318
\H,-0.1719234074,-4.5544104674,-2.2672384948\H,-0.8649554292,-4.277951
1044,-4.623101037\C,-1.9152006964,1.4030528149,-1.8829587264\C,-3.2087
553934,1.6348580033,-2.3375854378\C,-0.8852303618,2.2648612036,-2.2381

024869\C,-3.4637670367,2.7228721041,-3.1580954665\H,-4.0136812611,0.97
85024606,-2.0399005211\C,-1.1478561701,3.3457836295,-3.0598460925\H,0.
1103930558,2.1108530914,-1.851424107\C,-2.4351618557,3.5747232199,-3.5
22995932\H,-4.4702202251,2.9060462781,-3.50401569\H,-0.3471671257,4.01
80846844,-3.3304519015\H,-2.6366706024,4.4231384388,-4.1602925089\C,-4
.6781034815,-2.4145815746,0.6013863207\C,-5.3098596804,-3.4895830861,-
0.0381650647\C,-5.0914832416,-2.1088622251,1.9037383657\C,-6.320488242
8,-4.2039175972,0.5747530657\H,-4.9978604817,-3.7568563817,-1.03885902
99\C,-6.0968008581,-2.8324174365,2.5198508792\H,-4.5972282781,-1.32055
90073,2.448155378\C,-6.7259130778,-3.8764678459,1.8602091744\H,-6.7931
847726,-5.0212050744,0.048670263\H,-6.3889198587,-2.5780860213,3.52892
07596\H,-7.5166164421,-4.4318645356,2.343011646\\Version=EM64L-G09RevA
.01\State=1-A\HF=-1842.5317182\RMSD=8.645e-09\RMSF=2.385e-06\Dipole=1.
7281116,-0.467674,-1.7900911\Quadrupole=1.2258782,-3.9481537,2.7222755
,-3.6181933,0.3387796,2.5032991\PG=C01 [X(C35H29O2P1)]\\@

α-TS1’

1\1\GINC-SHI_02\FTS\RmPWPW91\6-31G(d)\C50H39O4P1\YIN\28-Feb-2014\0\\#p
opt=(calcfc,ts,noeigen) 6-31g(d) iop(3/76=0572004280) mpwpw91\\Title
Card Required\\0,1\C,2.6209388982,0.9610426227,1.8921801148\C,2.121789
325,-0.0230738487,2.7329529139\C,2.507548224,-0.0476946572,4.063124784
7\C,3.3723793779,0.9127718382,4.5599138029\C,3.8746090195,1.895746291,
3.7206677381\C,3.5145645426,1.9118946135,2.3851151217\H,1.4311055643,-
0.759162326,2.3563449844\H,2.1188873452,-0.8210237674,4.7074649757\H,3
.6620444461,0.8933162362,5.6003763326\H,4.5565403914,2.641779159,4.100
7660028\H,3.9415560221,2.656022661,1.7281184557\P,2.2274141394,0.96893
65486,0.1300360101\C,2.1839890636,-1.3219044301,-1.2418557928\H,3.2405
981112,-1.1479274782,-1.1123873092\C,1.3451530275,-0.4521424649,-0.623
0612174\C,-0.1143039517,-0.4246069381,-0.6269590749\C,-0.7196493042,0.
5280142326,0.2530359352\O,-0.1441848085,1.0128994546,1.2238119878\O,-1
.9821611297,0.8010168465,-0.052087879\C,-2.7280630732,1.6437996305,0.8
138905606\H,-3.4244554828,1.0006805378,1.3524284998\H,-2.0566769257,2.
1110148082,1.5274664279\C,-3.467072621,2.6672430113,0.005495705\C,-3.5
473451257,3.9792551248,0.4503691943\C,-4.1093987342,2.3118867578,-1.17
58050681\C,-4.2650496933,4.9255819725,-0.2648713794\H,-3.0429532626,4.
2647198202,1.363608746\C,-4.8171518895,3.2587736876,-1.8956659847\H,-4
.0478713087,1.2941194782,-1.5320759853\C,-4.9003273832,4.5669711307,-1
.4416049872\H,-4.3220315033,5.9428628211,0.0952810507\H,-5.3103630486,
2.9721001712,-2.8131478735\H,-5.4581408675,5.3021890747,-2.0035563474\
C,3.9067313959,1.0142911322,-0.593787432\C,4.1842730106,1.7444852131,-
1.7458031472\C,4.9343076407,0.2862869398,0.0085829599\C,5.4587631665,1
.7401001676,-2.2879800891\H,3.4104948574,2.3282910366,-2.2187015586\C,
6.2042603551,0.2781358034,-0.5409032513\H,4.7429639027,-0.2744452898,0
.9115534555\C,6.4691555471,1.0055848463,-1.690418602\H,5.6600599975,2.
3155264628,-3.1792047927\H,6.9882847642,-0.2923958945,-0.0655869056\H,
7.4617413634,1.0034312874,-2.1159613832\C,1.45525981,2.5037846348,-0.4

230223663\C,0.9317520334,2.5519061843,-1.7123586948\C,1.3713714011,3.6
198204369,0.4009848086\C,0.3414963061,3.7170076973,-2.1751658735\H,0.9
630876095,1.6787888689,-2.3481036666\C,0.7875239489,4.7813933898,-0.07
15696391\H,1.7288368309,3.5738644759,1.4175439668\C,0.2724101448,4.832
0021568,-1.357662438\H,-0.0785439576,3.7433565944,-3.1691134426\H,0.71
85814057,5.6442376713,0.5740199553\H,-0.1981880571,5.7351072144,-1.716
3110337\H,-0.5926176668,-0.5241592314,-1.5925932548\C,-1.0347711495,-2
.1868082396,-0.0112733367\H,-0.4968600129,-2.6827541222,-0.803377956\C
,-0.4821309172,-2.5093340022,1.3176134838\C,0.500877219,-3.4938642974,
1.4159166059\C,-0.9029928925,-1.8797757482,2.4883253066\C,1.0105295621
,-3.8740263718,2.646298509\H,0.8529173995,-3.9777082469,0.5158647023\C
,-0.4005959309,-2.2636832316,3.7200841807\H,-1.6243475771,-1.080714998
1,2.4324566291\C,0.5515535106,-3.2693095407,3.8057337967\H,1.758794321
8,-4.6515898569,2.6992257196\H,-0.7532377016,-1.7722264182,4.615580409
3\H,0.9338411366,-3.5773682674,4.7684654029\C,-2.414632851,-2.06297134
59,-0.2749894182\C,-3.6254157004,-1.9954982505,0.5092646088\C,-2.80786
95123,-1.8069575716,-1.6282615646\C,-4.6473037067,-1.713922625,-0.4103
750733\C,-4.0116448914,-2.2192979679,1.8303184165\C,-5.9755608547,-1.6
066531048,-0.0829450687\C,-5.3506678965,-2.1117274031,2.1843308343\H,-
3.2910915086,-2.5017843189,2.5797425412\C,-6.3257124792,-1.8001505668,
1.2485183765\H,-6.7095929791,-1.3855943789,-0.8425657499\H,-5.63520603
15,-2.284314491,3.2125646412\H,-7.3600354845,-1.7191196447,1.548214563
3\O,-2.1500374165,-1.7094896353,-2.6454914378\O,-4.1646272373,-1.58445
5113,-1.6716941763\C,1.9464142026,-2.4926063781,-2.0811452917\C,0.8000
890976,-2.7183012125,-2.8459703113\C,2.9891133386,-3.423211227,-2.1656
355919\C,0.694521788,-3.852224501,-3.6320251906\H,-0.0315421,-2.031783
5018,-2.8363416659\C,2.878588535,-4.5568194116,-2.9476639062\H,3.89476
78767,-3.2515582036,-1.5995515622\C,1.7248987418,-4.7778002943,-3.6845
625433\H,-0.2089952389,-4.0043029566,-4.2034264314\H,3.6937812863,-5.2
647138497,-2.9862128287\H,1.6330198594,-5.6611791233,-4.2994156439\\Ve
rsion=EM64L-G09RevA.01\State=1-A\HF=-2570.3667053\RMSD=4.609e-09\RMSF=
1.183e-06\Dipole=4.4492686,2.2257945,1.2756936\Quadrupole=0.2320583,1.
0110898,-1.2431481,-9.5437182,-1.214136,-5.0355707\PG=C01 [X(C50H39O4P
1)]\\@

α-INT1'

1\1\GINC-SHI_03\FOpt\RmPWPW91\6-31G(d)\C50H39O4P1\YIN\06-Mar-2014\0\\#
p opt 6-31g(d) iop(3/76=0572004280) mpwpw91\\pph3_s22_alpha_new_2\\0,1
\C,0.0118818727,-0.0421796046,0.0025391578\C,0.0100335935,-0.040050540
1,1.3952285587\C,1.2118880105,-0.0417167549,2.083965457\C,2.4129365437
,-0.0286454885,1.3953433608\C,2.4135883108,-0.0221422179,0.0099118112\
C,1.2201766339,-0.0357718196,-0.6877770216\H,-0.9192410862,-0.05808943
64,1.9428527099\H,1.2016821177,-0.0544803089,3.1636243635\H,3.34777507
32,-0.0266626711,1.9365762158\H,3.3460116063,-0.0209699024,-0.53427420
8\H,1.2343437421,-0.0575161856,-1.763844976\P,-1.5424878198,0.05650737
23,-0.9215914001\C,-3.3500304919,-1.5626360962,-2.0761439799\H,-2.9977

681148,-1.0572071973,-2.9653870181\C,-2.7658003115,-1.2896018125,-0.90
18550333\C,-3.0978831135,-2.0909148928,0.3370873229\C,-3.5681255345,-1
.2055192261,1.4670735545\O,-2.8738964406,-0.6684693377,2.2876524829\O,
-4.8927506965,-1.0621751484,1.4476803064\C,-5.4444660896,-0.2492888655
,2.4783925638\H,-5.2510182774,-0.7349260113,3.4344336668\H,-4.93430345
28,0.7104609031,2.4902687966\C,-6.910835076,-0.0874736398,2.2269589594
\C,-7.6877675496,-1.158768794,1.8000125723\C,-7.5212584273,1.137617044
9,2.4588750221\C,-9.0496190001,-1.0029868794,1.6077320884\H,-7.2196846
872,-2.1132874207,1.6100593063\C,-8.8865761418,1.2922600599,2.27564789
64\H,-6.9242595088,1.9774330377,2.7877881423\C,-9.6536560332,0.2220333
1,1.8474556208\H,-9.642838089,-1.841774331,1.273674347\H,-9.3486051396
,2.2507684072,2.4620028398\H,-10.7168630821,0.3410244165,1.6991890928\
C,-1.1993980187,0.5577996434,-2.6343800773\C,-1.5416272981,1.845573045
3,-3.0442250319\C,-0.5912370365,-0.3171762308,-3.5386536251\C,-1.27781
662,2.2556751865,-4.3405671861\H,-2.0150228657,2.5329100915,-2.3619088
696\C,-0.3354676037,0.1061436325,-4.8315182904\H,-0.323241177,-1.31217
9635,-3.2012210722\C,-0.6760441411,1.3877566793,-5.235383328\H,-1.5475
95201,3.2550858041,-4.6476827782\H,0.133951694,-0.5753113984,-5.525356
6096\H,-0.4735121538,1.7088695792,-6.2467388725\C,-2.4161262628,1.4814
148011,-0.1944335604\C,-3.7978583509,1.5889993457,-0.334698936\C,-1.71
30141482,2.5111344668,0.4267312092\C,-4.4663853819,2.7010788307,0.1497
048259\H,-4.3544300428,0.8020585896,-0.8200678238\C,-2.3847592507,3.62
20289442,0.9081775079\H,-0.6419097695,2.4451769589,0.5382214618\C,-3.7
606843572,3.7174581786,0.7736527641\H,-5.5387907126,2.7672006989,0.040
9971149\H,-1.83040764,4.4122692355,1.3920658833\H,-4.2816506867,4.5844
619106,1.1526875081\H,-3.9480946137,-2.703102412,0.0490843025\C,-1.945
500058,-3.0530821451,0.7344251891\H,-1.0736653243,-2.4338224963,0.9477
659447\C,-2.2435985969,-3.8331199908,1.9960319072\C,-1.1681091141,-4.3
562617,2.7085880478\C,-3.5258008533,-4.0865817762,2.4672557002\C,-1.36
44585301,-5.1016827569,3.8556456161\H,-0.1664547428,-4.1811959944,2.34
17370168\C,-3.7279701951,-4.8324941568,3.6209489747\H,-4.389952904,-3.
7179858123,1.9326585286\C,-2.6495351971,-5.3418189412,4.3204453781\H,-
0.5126828012,-5.4981898988,4.3893688497\H,-4.7350227592,-5.0176005734,
3.9668260332\H,-2.8059702967,-5.9235945388,5.2171335191\C,-1.588931516
3,-3.9322305529,-0.4185359871\C,-1.9969806325,-5.2478356817,-0.8017726
267\C,-0.765978239,-3.4538972568,-1.4253353304\C,-1.3679261868,-5.4841
587419,-2.0366618561\C,-2.7990964302,-6.2646122157,-0.2840734626\C,-1.
4948363616,-6.6485088964,-2.752996847\C,-2.9408721231,-7.4463759301,-0
.9977407909\H,-3.300248905,-6.1434193198,0.6637798897\C,-2.3028477693,
-7.646092082,-2.2160697633\H,-0.9800087488,-6.7745937744,-3.6940152595
\H,-3.561905869,-8.2324736579,-0.5908836342\H,-2.4278679738,-8.5784488
926,-2.747144181\O,-0.193553545,-2.3571289911,-1.5696261855\O,-0.63548
16102,-4.4086419513,-2.4179212676\C,-4.4381144103,-2.5297651027,-2.314
8062378\C,-4.2130661071,-3.6071234216,-3.1675140978\C,-5.6997813482,-2
.361442768,-1.7503848537\C,-5.2255637933,-4.5120793064,-3.4304995415\H
,-3.230828578,-3.7560743596,-3.5910572182\C,-6.7165590402,-3.258929690

9,-2.032379539\H,-5.8849116667,-1.5282974403,-1.0879454576\C,-6.481091
461,-4.3368913144,-2.8697196691\H,-5.0263058019,-5.3629688517,-4.06421
46999\H,-7.6931815987,-3.1133555001,-1.5938066622\H,-7.2712178877,-5.0
426023911,-3.0803457735\\Version=EM64L-G09RevA.01\State=1-A\HF=-2570.4
007474\RMSD=4.708e-09\RMSF=2.194e-06\Dipole=-1.2899272,4.3497535,0.102
9218\Quadrupole=7.1422536,-5.8773159,-1.2649377,10.7903213,-1.4777303,
-4.3894629\PG=C01 [X(C50H39O4P1)]\\@

α-TS2’

1\1\GINC-SHI_02\FTS\RmPWPW91\6-31G(d)\C50H39O4P1\YIN\25-Feb-2014\0\\#p
opt=(calcfc,ts,noeigen) 6-31g(d) iop(3/76=0572004280) mpwpw91\\Title
Card Required\\0,1\C,1.6789647907,1.5538239828,-2.2833055851\C,0.63458
81929,1.4822955785,-3.2050420089\C,0.9160242217,1.3971482774,-4.557862
0881\C,2.2295554499,1.4000059752,-4.9996237018\C,3.2673285502,1.510374
0589,-4.0883130135\C,2.9961581683,1.5949305885,-2.7329394491\H,-0.3952
465458,1.5244376063,-2.8710369502\H,0.102963548,1.3364082901,-5.265482
0035\H,2.444561974,1.3314394631,-6.0559311055\H,4.2911755757,1.5361109
901,-4.4309050051\H,3.8107867566,1.7037569046,-2.0339708301\P,1.213310
7552,1.7923977236,-0.5475526595\C,-1.0849956216,1.2063831203,0.7896494
498\H,-1.2951961813,2.2668966273,0.6946046976\C,-0.0430107782,0.720545
9381,0.0005200635\C,-0.1036719393,-0.6716462588,-0.5559949988\H,0.7081
504814,-0.7993155859,-1.274321494\C,0.0781478259,-1.7942416312,0.43924
193\O,-0.781940998,-2.4476747082,0.9539378912\O,1.3820644466,-2.004360
2566,0.6702824434\C,1.6821564686,-3.0631262145,1.5721501375\H,1.334682
2037,-2.7984789495,2.5686346759\H,1.1315071386,-3.9496771672,1.2622313
844\C,3.1602499844,-3.2944171069,1.5527060436\C,3.9014535505,-3.247067
8762,2.7240720059\C,3.8097613215,-3.5809638723,0.3561583775\C,5.267859
8447,-3.4846046416,2.7068288102\H,3.4076381764,-3.0200794382,3.6585910
816\C,5.173989861,-3.8101811669,0.3333350668\H,3.2393299068,-3.6201591
542,-0.5609101196\C,5.9076727439,-3.7638493607,1.5111413736\H,5.830991
004,-3.4490419027,3.6280493743\H,5.6662371756,-4.0343264587,-0.6019335
857\H,6.9715553043,-3.9505724882,1.4948433889\C,0.8034772181,3.5567015
687,-0.4471706436\C,1.7813921232,4.4928167678,-0.115209136\C,-0.445058
6049,3.9929698588,-0.8886772243\C,1.4977414651,5.846357974,-0.17430233
64\H,2.7682906621,4.1737299238,0.1816017721\C,-0.7197269018,5.34958988
55,-0.9384800324\H,-1.19934706,3.2880465041,-1.2196581196\C,0.24338468
29,6.2762311419,-0.5752869349\H,2.2610669117,6.5631551759,0.08972692\H
,-1.6927028492,5.6758605238,-1.2738742271\H,0.0224571302,7.3327209399,
-0.6181203393\C,2.671809345,1.493550944,0.4966548643\C,2.7733126008,2.
1359695587,1.7312149384\C,3.5498229104,0.4512079045,0.2070639254\C,3.7
72158605,1.7875471049,2.6223957765\H,2.0636092895,2.9019122365,2.00604
77111\C,4.5446378481,0.1004103611,1.1041457851\H,3.4468445522,-0.11311
66319,-0.7067783134\C,4.665400038,0.7757168333,2.3066406864\H,3.843480
9088,2.300681768,3.5699618534\H,5.2081391712,-0.7186175646,0.870717767
5\H,5.4399206925,0.4981043294,3.0061239439\C,-1.4709816188,-0.76774040
62,-1.3210422098\H,-1.2861649747,-0.1494867423,-2.2001867516\C,-1.7734

078587,-2.1437167803,-1.8888066335\C,-3.070933008,-2.5004790912,-2.244
0738774\C,-0.7528165245,-3.0402652648,-2.1983097693\C,-3.3394469937,-3
.7103308167,-2.8628928271\H,-3.8833765506,-1.8241190613,-2.0321326222\
C,-1.0168725283,-4.2511816175,-2.8164448978\H,0.2749154395,-2.80200415
28,-1.9636208009\C,-2.3151842597,-4.5960680156,-3.1501317724\H,-4.3592
99818,-3.9581842458,-3.1199457707\H,-0.2017133923,-4.9255189064,-3.037
7478135\H,-2.5249697445,-5.5409196513,-3.6300131509\C,-2.5825971258,-0
.0615337569,-0.6227195927\C,-3.6750773356,-0.5388917045,0.1783355726\C
,-3.0209747122,1.1806066196,-1.1753462857\C,-4.6454975631,0.4677354878
,0.1393467089\C,-3.9267380396,-1.6725640918,0.9437920124\C,-5.84470527
01,0.3964690334,0.8062269811\C,-5.1333290936,-1.7648966844,1.621583525
6\H,-3.1870010587,-2.453399856,1.0101683415\C,-6.0813208645,-0.7499242
805,1.5565957513\H,-6.565675146,1.1968860607,0.7386112485\H,-5.3385533
084,-2.6435327567,2.2159550247\H,-7.0138296812,-0.8500517896,2.0927895
331\O,-4.2449092623,1.5144727134,-0.6324625887\O,-2.4941293974,1.91600
0658,-1.9950310286\C,-1.5724429217,0.7044410861,2.0773070938\C,-2.7779
441746,1.1923994573,2.5865121347\C,-0.7982045066,-0.1203355475,2.88962
6596\C,-3.2251166925,0.813978665,3.8371976294\H,-3.3651956572,1.872630
2983,1.9877329152\C,-1.238684802,-0.4886954225,4.1482762863\H,0.178624
5628,-0.4289910381,2.5501931559\C,-2.4595204209,-0.0358671366,4.621082
9586\H,-4.1716312885,1.1850575463,4.2010185342\H,-0.6208380143,-1.1239
830448,4.7665846914\H,-2.8045884893,-0.3284097576,5.602123463\\Version
=EM64L-G09RevA.01\State=1-A\HF=-2570.382481\RMSD=8.378e-09\RMSF=2.148e
-06\Dipole=3.995851,0.852158,0.5152905\Quadrupole=-0.6227591,-0.282162
9,0.904922,-1.2838949,-4.0536067,2.154792\PG=C01 [X(C50H39O4P1)]\\@

α-INT2’

1\1\GINC-SHI_03\FOpt\RmPWPW91\6-31G(d)\C50H39O4P1\YIN\10-Mar-2014\0\\#
p opt 6-31g(d) iop(3/76=0572004280) mpwpw91\\pph3_s1_alpha_step2_new_2
\\0,1\C,-0.0926632258,-0.0073325395,0.0820417149\C,-0.2031803102,-0.25
70616708,1.4439407778\C,0.9309760411,-0.3051091949,2.2407230506\C,2.18
06170002,-0.0980322828,1.6829680606\C,2.3007861214,0.1554928985,0.3235
283789\C,1.1714675763,0.192612983,-0.4747170746\H,-1.1824713258,-0.382
4991563,1.8779897736\H,0.8338379691,-0.5014714394,3.2982403687\H,3.063
732486,-0.1355982689,2.3043018346\H,3.2752760827,0.315289623,-0.114692
6796\H,1.2736826762,0.3706208961,-1.5359923488\P,-1.6238884812,0.01393
83169,-0.9376276514\C,-3.2394449758,-2.0726027214,0.0705665532\H,-2.58
68872409,-2.3446218907,0.9085602182\C,-3.0725233144,-0.6111276991,-0.3
257805367\C,-4.0869620561,0.2375897256,0.4336037989\C,-3.6001170608,1.
4401028926,1.2063658818\O,-2.6750546826,1.455452024,1.9755074301\O,-4.
3662799943,2.5033029745,0.9792297304\C,-4.0329776611,3.6734097567,1.71
64172086\H,-3.9844137278,3.4146191687,2.7730423622\H,-3.0442793227,4.0
12893438,1.4168691226\C,-5.0731273858,4.7158602344,1.4537888116\C,-6.4
243522403,4.411987485,1.5821740433\C,-4.7015101101,6.0090372485,1.1159
997443\C,-7.3824742392,5.3881722477,1.3749688026\H,-6.7203964729,3.404
6945058,1.8382376762\C,-5.6604166795,6.9907441663,0.9170312987\H,-3.65

31808138,6.2508245498,1.0064162549\C,-7.0035073498,6.6814167915,1.0446
873051\H,-8.4295101346,5.1413706027,1.475644532\H,-5.3566740017,7.9944
395913,0.6570706497\H,-7.7534000229,7.4429337295,0.8871568211\C,-1.022
2118439,-0.8286200649,-2.4424258986\C,-1.3448851207,-0.348923812,-3.70
7147053\C,-0.2658717597,-1.9926681091,-2.3317671376\C,-0.9225244922,-1
.0230762959,-4.8411515157\H,-1.9170705323,0.5601982415,-3.8123585698\C
,0.1462889156,-2.670669866,-3.4653369775\H,-0.0027447206,-2.3773149981
,-1.3580703875\C,-0.1799559754,-2.1862336017,-4.7218348775\H,-1.170663
8228,-0.6348426416,-5.818231372\H,0.7186574102,-3.5806070891,-3.364864
6003\H,0.1466073701,-2.7131872969,-5.6065395344\C,-1.9071968438,1.7526
833382,-1.3749943318\C,-3.0716125072,2.0850581549,-2.0656668528\C,-1.0
054612349,2.7575099241,-1.0404452473\C,-3.3231736184,3.3971155967,-2.4
259394772\H,-3.7826282252,1.3077579418,-2.3052106977\C,-1.2598836037,4
.0705585037,-1.4034510551\H,-0.1118305037,2.5174967222,-0.4858338333\C
,-2.4154592652,4.3916019614,-2.0971855533\H,-4.2323517168,3.6447225157
,-2.9531104498\H,-0.5534123351,4.8436514532,-1.1387953588\H,-2.6133669
122,5.4163849511,-2.3753669371\H,-4.8885166201,0.6047152555,-0.2104827
857\C,-4.6634868929,-0.7505124017,1.4619991208\H,-3.8983681476,-0.8731
419889,2.2302493438\C,-5.9447789622,-0.3737220566,2.1545431747\C,-6.08
10414482,-0.6689299442,3.5084135046\C,-7.0120977318,0.2393706474,1.503
7710657\C,-7.2462868877,-0.3670772475,4.1936135455\H,-5.2627807748,-1.
1481289976,4.0275659192\C,-8.1772627204,0.5460034655,2.1876050568\H,-6
.9408716316,0.4792387822,0.4544764652\C,-8.2995082695,0.2441802881,3.5
347171282\H,-7.3276535704,-0.6078731286,5.2435213879\H,-8.9948708228,1
.0182527086,1.6620137038\H,-9.2092835916,0.4837962014,4.0656863917\C,-
4.662707925,-2.0882770868,0.6952160699\C,-5.8183783349,-2.3079952907,-
0.2262809746\C,-4.8298389704,-3.2488649074,1.6633151784\C,-6.503968315
6,-3.4202737243,0.2281558396\C,-6.288145905,-1.6496747124,-1.346579536
3\C,-7.6396926084,-3.9124785018,-0.3691316819\C,-7.4351105933,-2.11947
15913,-1.9751541603\H,-5.7632165551,-0.7912435897,-1.7371933649\C,-8.1
011104254,-3.2355583239,-1.4903775022\H,-8.1403382894,-4.7836947554,0.
0222894874\H,-7.8089513906,-1.6130509882,-2.8525741456\H,-8.9905271426
,-3.5881096827,-1.9913750103\O,-4.1411943296,-3.5256848472,2.594555830
6\O,-5.9264131201,-3.9770227171,1.3372578771\C,-3.0513131854,-3.153469
3098,-0.9679932567\C,-2.5161447885,-4.3761935573,-0.5748803824\C,-3.43
61281868,-2.9976032101,-2.2948556259\C,-2.3741203642,-5.4180330176,-1.
4783913498\H,-2.2132314145,-4.5137040163,0.4547385096\C,-3.2977850272,
-4.0346268186,-3.1997344916\H,-3.8254544483,-2.0445254259,-2.615959905
1\C,-2.7662571929,-5.2500262743,-2.7955526863\H,-1.957268395,-6.359506
0626,-1.1504018419\H,-3.5990419543,-3.8912023825,-4.2274012602\H,-2.65
63632409,-6.0586653263,-3.5038676387\\Version=EM64L-G09RevA.01\State=1
-A\HF=-2570.4244935\RMSD=6.961e-09\RMSF=2.543e-06\Dipole=0.5579506,1.6
62562,-1.9093025\Quadrupole=10.5440857,-5.0199948,-5.5240909,-3.878641
9,0.4899365,4.4760224\PG=C01 [X(C50H39O4P1)]\\@

γ-TS1’

1\1\GINC-SHI_03\FTS\RmPWPW91\6-31G(d)\C50H39O4P1\YIN\10-Feb-2014\0\\#p
6-31g(d) iop(3/76=0572004280) mpwpw91 opt=(calcfc,ts,noeigen)\\pph3_s
2_gamma_ts1\\0,1\C,-0.1832410712,-2.6467785391,0.1502508625\C,-0.54453
849,-2.5854788933,-1.1888728979\C,-0.4663318179,-3.7269974322,-1.96912
98865\C,-0.0602688267,-4.9282773013,-1.4141056621\C,0.2974951076,-4.99
17949614,-0.0758125101\C,0.2556808207,-3.8502965715,0.7035321584\H,-0.
856860196,-1.6557639328,-1.6361952877\H,-0.7159507259,-3.6630170074,-3
.0167340527\H,-0.0078922327,-5.81595871,-2.027282555\H,0.6281566479,-5
.9239486282,0.3573831341\H,0.5779904064,-3.8969420175,1.7338425501\P,-
0.1909292097,-1.1837493032,1.2051997373\C,0.7259225227,1.3490852543,0.
5326941287\H,1.4998965148,0.9849389234,1.1915900109\C,-0.4017104604,0.
4680454734,0.4338933668\C,-1.6502377648,0.806137034,-0.0068938741\H,-1
.8487393698,1.8154645873,-0.3175975149\C,-2.7485457257,-0.1104045408,-
0.0915859371\O,-2.69677428,-1.2954767784,0.1902561879\O,-3.8701361894,
0.4708143726,-0.5188045685\C,-4.9948579451,-0.3820759084,-0.6570046166
\H,-4.6981946887,-1.2628817533,-1.2253213774\H,-5.3120391898,-0.725694
6316,0.3261748392\C,-6.0895951924,0.3693251403,-1.3480196863\C,-7.4047
277132,0.224007213,-0.9301537207\C,-5.813674784,1.1815250228,-2.441904
9453\C,-8.4330981344,0.8707759073,-1.5975799789\H,-7.6282449634,-0.398
0268266,-0.0740274684\C,-6.8383280151,1.8350005474,-3.1035635794\H,-4.
7908307807,1.3063792145,-2.7649796497\C,-8.1515777682,1.6792199859,-2.
6855857704\H,-9.4523124239,0.7489315665,-1.2607793059\H,-6.6115303534,
2.4670396249,-3.9498283298\H,-8.9502774566,2.1891340668,-3.2042243693\
C,1.4480512491,-1.2638256485,1.9916760383\C,1.6197787547,-1.0209091173
,3.3520959495\C,2.5516392413,-1.6130965982,1.214378284\C,2.8774224289,
-1.1222043735,3.9239029219\H,0.7751263332,-0.7640441334,3.971855527\C,
3.8057000988,-1.7143286151,1.7918356881\H,2.4428068529,-1.802580411,0.
1574459701\C,3.9699037232,-1.4702418142,3.1466173073\H,2.9988072779,-0
.9360174806,4.9806433682\H,4.6477700901,-1.98257465,1.171972732\H,4.94
82197778,-1.5552408497,3.5965863464\C,-1.3625612015,-1.2903054611,2.57
93091555\C,-1.628955701,-0.1398609392,3.3162754816\C,-1.9567760479,-2.
495168712,2.9317587949\C,-2.4755389258,-0.2043375061,4.4108950796\H,-1
.1917224139,0.8061205648,3.0301665531\C,-2.800108696,-2.5510545191,4.0
275484856\H,-1.7873734406,-3.378652904,2.336347909\C,-3.0577961874,-1.
4091118242,4.7694756472\H,-2.6841269336,0.6913108017,4.9767553976\H,-3
.2670345467,-3.4876319335,4.2935932794\H,-3.7201720798,-1.4562857016,5
.621222651\C,1.8743769284,1.4024535424,-1.2238450271\H,1.060466741,1.9
39796457,-1.693722864\C,2.9689062,2.279907023,-0.7423491545\C,3.127561
9135,3.5346785967,-1.3237654552\C,3.8460791707,1.9090347669,0.27269109
49\C,4.1454828291,4.3826300421,-0.9215229361\H,2.4464632951,3.84544063
22,-2.1024800194\C,4.8594386026,2.7583714187,0.683183027\H,3.725872605
7,0.9504030617,0.7562639865\C,5.0165158303,3.998129968,0.0842284504\H,
4.2522079924,5.3495307581,-1.3913864106\H,5.5264301952,2.4510860306,1.
4761212184\H,5.8068695845,4.6615525815,0.40384898\C,2.1512851037,0.203
9315362,-1.9012931719\C,3.312250957,-0.6352809654,-2.0765401977\C,1.13
68603398,-0.3336996395,-2.7672909432\C,2.9275877481,-1.6134151085,-3.0

060004892\C,4.6243103893,-0.6790024363,-1.6091382678\C,3.7541657616,-2
.6119268533,-3.4569475111\C,5.4777002792,-1.6805722878,-2.0560757179\H
,4.9916954428,0.0682643443,-0.9252794524\C,5.0535644358,-2.6420443798,
-2.9621472024\H,3.3981562529,-3.3342859179,-4.1753009559\H,6.496490171
9,-1.701540962,-1.6956446481\H,5.735024201,-3.4109191045,-3.2949367241
\O,1.6466200209,-1.4407360338,-3.4158790042\O,-0.0036019626,0.01434614
06,-2.9700545581\C,0.4905760368,2.7975488706,0.7345205881\C,1.03714784
03,3.4109347027,1.8640177587\C,-0.2112748667,3.6072800618,-0.161421196
2\C,0.8686617081,4.761843683,2.1054110287\H,1.6094471936,2.8122959565,
2.5590991437\C,-0.3840128084,4.9607242828,0.0813786953\H,-0.6054550435
,3.1911464681,-1.0767785575\C,0.1485678038,5.5455658427,1.217119424\H,
1.3034790771,5.2045978362,2.9901168945\H,-0.9337458084,5.5606276587,-0
.6297015938\H,0.0131869323,6.6008000543,1.4036537696\\Version=EM64L-G0
9RevA.01\State=1-A\HF=-2570.3572801\RMSD=3.430e-09\RMSF=8.043e-07\Dipo
le=-0.3506784,-1.0893426,3.3291642\Quadrupole=-0.4284012,5.5797223,-5.
1513211,-0.0320073,7.6847315,-5.1201039\PG=C01 [X(C50H39O4P1)]\\@

γ-INT1'

1\1\GINC-SHI_03\FOpt\RmPWPW91\6-31G(d)\C50H39O4P1\YIN\27-Jan-2014\0\\#
p opt 6-31g(d) iop(3/76=0572004280) mpwpw91\\pph3_s2_gamma\\0,1\C,0.04
91708066,0.1043961838,-0.1368179219\C,0.0730474884,0.0307955792,1.2494
520785\C,1.2903991512,-0.0501519914,1.9050471803\C,2.4753109256,-0.031
4061537,1.1906504528\C,2.4530762384,0.0471965198,-0.1939812838\C,1.243
3608168,0.0983517038,-0.8606745926\H,-0.8391279386,0.0232969262,1.8216
23054\H,1.2982689364,-0.1390190606,2.9802035447\H,3.4202962816,-0.0916
068295,1.7104145977\H,3.3748977684,0.0481099983,-0.7562101761\H,1.2309
679548,0.1189545877,-1.9409367178\P,-1.4876321767,0.1582204903,-1.0797
692547\C,-3.7697124526,-1.6016873572,-0.6955889277\H,-3.146396358,-2.1
296213134,-1.4129711778\C,-3.008873104,-0.3501921276,-0.2340280491\C,-
3.5433420286,0.4103950653,0.7328653836\H,-4.4860324473,0.1327350262,1.
1711499318\C,-2.9677307727,1.6530426262,1.2436898658\O,-1.8880679941,2
.089157943,0.9158090846\O,-3.7913976599,2.2695271161,2.0736168011\C,-3
.318664197,3.4815408397,2.6479866474\H,-2.5813766846,3.2401771525,3.41
08527933\H,-2.8163183555,4.0591509105,1.8737653607\C,-4.477591304,4.22
99466645,3.2273409451\C,-4.3490931493,4.8746889655,4.4491087771\C,-5.6
76829866,4.3314887621,2.5322574812\C,-5.3974317946,5.619177041,4.96668
76165\H,-3.4250164219,4.790389844,5.0043708665\C,-6.7275739932,5.06607
94787,3.0522545566\H,-5.7885139087,3.8198076077,1.58772578\C,-6.589683
0225,5.7156807976,4.2696847289\H,-5.2843112162,6.1140830396,5.92012096
02\H,-7.6575414781,5.1320676028,2.5064346365\H,-7.4104923399,6.2890640
835,4.674947666\C,-1.1800271221,-1.0175314945,-2.4248011447\C,-1.37981
35129,-0.6839161344,-3.7605691229\C,-0.7061988924,-2.2863737648,-2.090
2041666\C,-1.1190259655,-1.6175893693,-4.7521047265\H,-1.7307199156,0.
3001146409,-4.0314774274\C,-0.4566484937,-3.2143055356,-3.0845054682\H
,-0.5439435615,-2.5612114541,-1.0573747921\C,-0.6617488157,-2.88076165
41,-4.4153091176\H,-1.2709365076,-1.3527214528,-5.7879363186\H,-0.1022

35334,-4.1958830176,-2.8089045156\H,-0.4612616538,-3.6056918521,-5.190
547023\C,-1.7292694839,1.7656333594,-1.8755119287\C,-2.962784764,2.048
0034807,-2.4556159949\C,-0.7000942868,2.6970467838,-1.9424504931\C,-3.
1554280665,3.2520655792,-3.1125150255\H,-3.775680153,1.3381439364,-2.3
908968879\C,-0.90142698,3.8981639373,-2.5999128405\H,0.2449944899,2.49
69461573,-1.4620162469\C,-2.1256563363,4.1760227716,-3.1871218208\H,-4
.1146238735,3.4676263118,-3.5591483606\H,-0.1022244869,4.6229633947,-2
.6430522879\H,-2.2795983417,5.1163984965,-3.6955063213\C,-3.9272208361
,-2.521426614,0.5237515675\H,-4.571672558,-2.011116535,1.2432150409\C,
-4.585564687,-3.8550277766,0.2360983695\C,-4.9718339464,-4.6353386741,
1.3240608116\C,-4.8049367214,-4.3584384244,-1.0392093335\C,-5.56011826
01,-5.8726650176,1.1451242445\H,-4.7883826378,-4.2637409585,2.32281042
64\C,-5.3936627734,-5.6025695362,-1.2238963723\H,-4.5282582123,-3.7825
528357,-1.9093459626\C,-5.7748100757,-6.3640635464,-0.1348228745\H,-5.
8491598275,-6.4579011394,2.0062432788\H,-5.5558738298,-5.9709733933,-2
.2268826961\H,-6.2340930321,-7.3313562898,-0.2781856359\C,-2.606857295
6,-2.6675333258,1.2252676539\C,-1.5922816916,-3.6652138589,1.102818596
2\C,-2.2681760221,-1.866488186,2.3234500863\C,-0.6566646166,-3.3765001
481,2.1117768643\C,-1.3462897671,-4.7658688155,0.2805252356\C,0.486178
2957,-4.1081014467,2.3193861061\C,-0.1922833457,-5.5141377336,0.479244
3804\H,-2.053309522,-5.0481108065,-0.4855625494\C,0.7184017091,-5.1931
680088,1.4780437157\H,1.1702326056,-3.846405533,3.1126487152\H,-0.0073
127659,-6.373810589,-0.1501925821\H,1.6069708803,-5.7924263129,1.61216
22529\O,-1.0449636863,-2.3043478944,2.8351330394\O,-2.8043290255,-0.88
12924685,2.8315147156\C,-5.0464411414,-1.2103312141,-1.4153788054\C,-5
.0326313528,-1.0500362375,-2.7986471104\C,-6.243338981,-0.9857367435,-
0.7423573058\C,-6.1697079752,-0.6595276754,-3.4884215134\H,-4.11987426
05,-1.2498063452,-3.344734239\C,-7.3822265702,-0.5971532507,-1.4282745
21\H,-6.3021390416,-1.1344270095,0.3254414711\C,-7.3503482358,-0.42683
25552,-2.8030187056\H,-6.1344230984,-0.5490061541,-4.5628806212\H,-8.3
018911609,-0.4363481463,-0.884696459\H,-8.2413395877,-0.1285938824,-3.
33582568\\Version=EM64L-G09RevA.01\State=1-A\HF=-2570.3948039\RMSD=3.7
70e-09\RMSF=2.258e-06\Dipole=0.5146026,2.5835815,-3.3943418\Quadrupole
=6.23311,-7.1563108,0.9232008,-0.8993367,-2.9401813,8.4915084\PG=C01 [
X(C50H39O4P1)]\\@

γ-TS2'

1\1\GINC-SHI_02\FTS\RmPWPW91\6-31G(d)\C50H39O4P1\YIN\07-Mar-2014\0\\#p
opt=(calcfc,ts,noeigen) 6-31g(d) iop(3/76=0572004280) mpwpw91\\Title
Card Required\\0,1\C,-1.1008121698,-2.0693381236,2.0788957982\C,-0.214
7841387,-1.4085186287,2.9172903094\C,-0.1847382449,-1.7132432374,4.269
1228196\C,-1.0270630412,-2.6824631036,4.7853027095\C,-1.9168980153,-3.
3452440549,3.9522875329\C,-1.9616611443,-3.0346743359,2.6053536307\H,0
.4444398769,-0.6551735795,2.5228756522\H,0.4997165146,-1.1817782786,4.
9122473431\H,-0.9973904938,-2.9192640008,5.838904622\H,-2.5826020692,-
4.0957485442,4.3521754606\H,-2.6725726442,-3.5392398429,1.9664413177\P

,-1.2102905704,-1.6993734388,0.2995283214\C,-1.3761375855,0.9505206686
,-0.7668332063\H,-2.1784986106,1.0175141827,-0.0353908023\C,-0.5290140
155,-0.2562622568,-0.4316828582\C,0.8199554754,-0.0389836815,-0.602836
9577\H,1.1873052514,0.5938292011,-1.3977041985\C,1.8473587978,-0.94157
74694,-0.0627788539\O,1.6243298525,-1.8439678198,0.7111945467\O,3.0462
594026,-0.6411395261,-0.5209030883\C,4.1419582158,-1.3663035832,0.0156
307949\H,4.129079581,-1.2530424718,1.0996241\H,4.0119509312,-2.4249680
208,-0.2017279232\C,5.4096151644,-0.8336406341,-0.5783420142\C,6.52186
19438,-1.6629777241,-0.6468047377\C,5.5065493335,0.4798475281,-1.02142
69584\C,7.7245255528,-1.1879081833,-1.1417454494\H,6.4477319876,-2.689
6136602,-0.3127410313\C,6.7099229096,0.9485165366,-1.5244158202\H,4.64
47560095,1.1309235462,-0.9877032679\C,7.8203718926,0.1219270324,-1.583
3755375\H,8.5823364026,-1.842992175,-1.1905161919\H,6.7751938704,1.969
3163353,-1.8715744635\H,8.7552539607,0.4947571482,-1.9761873137\C,-3.0
098678431,-1.577781947,0.0581074555\C,-3.6461366565,-2.1538841726,-1.0
345730852\C,-3.7635693698,-0.8607804769,0.9873300517\C,-5.0145546161,-
2.0113562859,-1.1991260608\H,-3.0802614086,-2.7191959653,-1.7581702153
\C,-5.1282961111,-0.7129003404,0.8148639664\H,-3.28497819,-0.429989131
6,1.8551764218\C,-5.7554770361,-1.2898634002,-0.2793331027\H,-5.498689
5389,-2.4645422238,-2.0510438381\H,-5.702805262,-0.1557969388,1.539997
8826\H,-6.8219494074,-1.1801030827,-0.4103139921\C,-0.6480794979,-3.16
01325718,-0.6161546531\C,-0.5440483262,-3.0661379471,-2.0022223973\C,-
0.3264637699,-4.3540098824,0.018704097\C,-0.1467608298,-4.1654631409,-
2.7443645915\H,-0.7646712259,-2.1309223317,-2.4973614024\C,0.075191781
9,-5.4480360557,-0.7287897407\H,-0.3635719031,-4.4240669953,1.09426402
48\C,0.1610554716,-5.357570315,-2.1086277771\H,-0.0691734943,-4.085523
6413,-3.8184515174\H,0.3325156689,-6.3699067418,-0.2287547086\H,0.4778
047132,-6.2125363439,-2.6876892956\C,-0.4619968073,2.2248621031,-0.658
2427608\H,0.0204520478,2.327890798,-1.6314191417\C,-1.229746514,3.5162
522703,-0.4516185981\C,-2.3848239161,3.6315956005,0.312214579\C,-0.713
6570547,4.6730730762,-1.0321379366\C,-3.004687581,4.8598050731,0.49027
29143\H,-2.8227435384,2.7621920298,0.7802119756\C,-1.3278058275,5.8994
830218,-0.8574483294\H,0.191680204,4.6021132663,-1.6192633545\C,-2.480
8047074,5.9989283785,-0.0937686135\H,-3.9019859923,4.9221244998,1.0894
468402\H,-0.9054021524,6.7797786911,-1.3200707491\H,-2.9650067767,6.95
47957932,0.0433267805\C,0.6731875407,2.0766324311,0.3051353918\C,0.645
9982198,2.0808793777,1.7333814598\C,2.017977644,2.4031226109,-0.068274
3496\C,1.9732473495,2.2821119514,2.1441719882\C,-0.3341858093,1.932138
9402,2.7069262985\C,2.3571419139,2.3175177082,3.4593600861\C,0.0348687
341,1.9738975017,4.0476517795\H,-1.370505626,1.8050545493,2.4333666023
\C,1.358593655,2.1548775682,4.4205237636\H,3.3888672639,2.4795223974,3
.7308013671\H,-0.7231476069,1.8675039036,4.8102275884\H,1.621905878,2.
1892754484,5.4675853981\O,2.7951533936,2.4502898534,1.0815261556\O,2.5
235288304,2.5511427907,-1.1581013212\C,-2.0356616191,0.9110536647,-2.1
299078277\C,-3.3830566412,1.216151466,-2.2710250927\C,-1.3087767447,0.
5944844877,-3.275457772\C,-3.9919687781,1.2027128087,-3.5168266265\H,-

3.9648597249,1.4655488803,-1.3952491456\C,-1.9129893052,0.5776915563,-
4.5203544564\H,-0.2557227727,0.3625465709,-3.1954477328\C,-3.260435607
9,0.8817286773,-4.646683165\H,-5.041322908,1.4462605264,-3.6011412057\
H,-1.3279645896,0.3338435984,-5.3955697179\H,-3.7320694056,0.872878257
,-5.6184838786\\Version=EM64L-G09RevA.01\State=1-A\HF=-2570.3826213\RM
SD=7.103e-09\RMSF=2.347e-06\Dipole=-2.7721798,-3.3383601,0.4713022\Qua
drupole=3.1345642,-5.0462791,1.9117149,-4.9609663,-0.6989157,1.860223\
PG=C01 [X(C50H39O4P1)]\\@

γ-INT2'

1\1\GINC-SHI_03\FOpt\RmPWPW91\6-31G(d)\C50H39O4P1\YIN\21-Jan-2014\0\\#
p opt 6-31g(d) iop(3/76=0572004280) mpwpw91\\pph3_s22_gamma_step2\\0,1
\C,-0.0421059814,-0.1064356293,-0.0002305619\C,-0.075704416,-0.2818645
23,1.3763928968\C,1.1002261696,-0.342827012,2.107575996\C,2.3225204723
,-0.2216629914,1.468188956\C,2.3679816817,-0.0381977654,0.0941590952\C
,1.1933691807,0.0131671211,-0.636658031\H,-1.0316246863,-0.3372422301,
1.870725653\H,1.0598948781,-0.4786417301,3.1785605281\H,3.2394844464,-
0.2691064479,2.0376546793\H,3.3188622688,0.0577407363,-0.4096376903\H,
1.2395601452,0.1415159692,-1.7085584471\P,-1.6211884546,-0.0588709994,
-0.954289888\C,-3.4621783993,-2.1231191233,-0.171324882\H,-2.917025585
7,-2.6273836776,0.6358499114\C,-3.0616492221,-0.6616741328,-0.31131625
49\C,-4.080271574,0.2202962235,0.3795675777\H,-4.7527323167,0.73038855
79,-0.3194258618\C,-3.6010319349,1.308035383,1.3078414949\O,-2.4673957
313,1.5539170024,1.6151477538\O,-4.6415056416,2.0052502734,1.764782955
3\C,-4.3445240517,3.0592797894,2.6633344754\H,-3.8289224149,2.65077577
42,3.5310524106\H,-3.6560804394,3.7487954207,2.1755843569\C,-5.6121541
901,3.7517386976,3.0646875344\C,-5.5546571729,4.6971960333,4.083046925
4\C,-6.8278513343,3.4957099137,2.4476326052\C,-6.6914408141,5.37983714
73,4.4764760442\H,-4.6116329476,4.8991774156,4.574089373\C,-7.96789352
44,4.1776987084,2.8467104174\H,-6.8881163129,2.7593869423,1.6620173446
\C,-7.9052110383,5.1205879984,3.8577871722\H,-6.6311061498,6.110938286
3,5.2696647442\H,-8.9095051598,3.9642057739,2.3622538518\H,-8.79570277
98,5.6488006084,4.1659079187\C,-1.0782686428,-0.9118939464,-2.47588918
68\C,-1.2958903226,-0.3590925591,-3.732932166\C,-0.4506901582,-2.15189
74775,-2.382548816\C,-0.9016254976,-1.0370484455,-4.8753422008\H,-1.76
30960266,0.6089694198,-3.8270613876\C,-0.072721468,-2.8348278554,-3.52
43220493\H,-0.2544109699,-2.5877864268,-1.4144470563\C,-0.295821527,-2
.2775381061,-4.7734815415\H,-1.0672905504,-0.5910749904,-5.8452184345\
H,0.3964837192,-3.8033184599,-3.4372812492\H,0.0062027528,-2.808296467
9,-5.6645694016\C,-1.8627854229,1.6851177981,-1.4015364199\C,-3.018874
5194,2.0346838878,-2.0967646306\C,-0.9395841296,2.6723202342,-1.076946
1515\C,-3.2425837306,3.3495681752,-2.4692614716\H,-3.7425665502,1.2699
845604,-2.340547609\C,-1.1655445241,3.9865881485,-1.4515462834\H,-0.05
36343615,2.4185331007,-0.5164486887\C,-2.3140549175,4.3269807547,-2.14
75023239\H,-4.1436547675,3.6097802372,-3.0047150584\H,-0.4447584495,4.
7475721523,-1.1909681473\H,-2.4889708667,5.3537237327,-2.4340590074\C,

-4.9410276949,-2.0088748933,0.2471400359\H,-5.4865690576,-1.6990915252
,-0.6460072251\C,-5.6189960864,-3.2388673235,0.7842237554\C,-5.0195844
036,-4.098371938,1.7004410316\C,-6.9145350866,-3.5266170561,0.36489269
29\C,-5.6935589189,-5.2094642014,2.178548892\H,-4.0182969935,-3.900646
8653,2.0506737612\C,-7.5939121835,-4.6341330488,0.844574784\H,-7.39648
23548,-2.8693197983,-0.34534402\C,-6.9836650124,-5.4819556928,1.753217
4735\H,-5.2078959386,-5.8637855442,2.8881169257\H,-8.5990425876,-4.835
0583763,0.5033978858\H,-7.5081250174,-6.349392992,2.1268414762\C,-4.97
80178405,-0.7686131,1.16490803\C,-4.6213690574,-0.9943701706,2.6002892
16\C,-6.4065591045,-0.2594950663,1.2937651634\C,-5.7400996299,-0.69144
47772,3.3571213655\C,-3.47002988,-1.4050464781,3.2434970366\C,-5.77844
71628,-0.7870031558,4.727507645\C,-3.474869397,-1.5126758755,4.6296336
519\H,-2.5799423603,-1.639857173,2.6806043389\C,-4.6153348889,-1.20978
62836,5.3586651574\H,-6.6726309752,-0.5389282675,5.2766963608\H,-2.581
1512317,-1.8339218433,5.1433435509\H,-4.6017229204,-1.298856267,6.4348
572128\O,-6.7945764671,-0.2768115963,2.5926920207\O,-7.1343895649,0.09
46199003,0.420689272\C,-3.3660349695,-3.0326460092,-1.3750558553\C,-2.
8717999752,-4.3227185192,-1.2352886813\C,-3.8383280156,-2.6390583012,-
2.6226975601\C,-2.8402071049,-5.1982491668,-2.3102897995\H,-2.51163486
46,-4.6495167379,-0.2690194795\C,-3.8090501782,-3.5066210816,-3.698766
2173\H,-4.2135782613,-1.6329043535,-2.7470403809\C,-3.3079610741,-4.79
19207542,-3.5473191685\H,-2.452717489,-6.1983559793,-2.1773989435\H,-4
.1744736002,-3.179316985,-4.6615468172\H,-3.2850464528,-5.4694544592,-
4.3886612605\\Version=EM64L-G09RevA.01\State=1-A\HF=-2570.4236694\RMSD
=7.051e-09\RMSF=2.393e-06\Dipole=1.9120649,0.2486764,-0.1629908\Quadru
pole=-8.5831338,-0.3871146,8.9702484,-2.1843053,-1.2502857,-2.2959892\
PG=C01 [X(C50H39O4P1)]\\@

11. References

[1] Y.-Q. Jiang, Y.-L. Shi, M. Shi, *J. Am. Chem. Soc.* **2008**, *130*, 7202.

[2] (a) H. Xiao, Z. Chai, C.-W. Zheng, Y.-Q. Yang, W. Liu, J.-K. Zhang, G. Zhao, *Angew. Chem. Int. Ed.* **2010**, *49*, 4467; (b) X. Han, Y. Wang, F. Zhong, Y. Lu, *J. Am. Chem. Soc.* **2011**, *133*, 1726.

[3] S.-F. Zhu, Y. Yang, L.-X. Wang, B. Liu, Q.-L. Zhou, *Org. Lett.* **2005**, *7*, 2333.

[4] (a) R. W. Lange, H.-J. Hansen, *Org. Synth.* **1984**, *62*, 202; (b) B. T. B. Hue, J. Dijkink, S. Kuiper, S. Schaik, J. H. Maarseveen, H. Hiemstra, *Eur. J. Org. Chem.* **2006**, 127; (c) S. Castellano, P. De Leon, H. Fiji, S. Kinderman, O. Kwon, F. Tamanoi, M. Watanabe, *J. Am. Chem. Soc.* **2007**, *129*, 5843.

[5] (a) M. Msaddek, M. Rammah, K. Ciamala, J. Vebrel, B. Laude, *Synthesis* **1997**, 1495.

[6] A. Voituriez, N. Pinto, M. Neel, P. Retailleau, A. Marinetti, *Chem. Eur. J.* **2010**, *16*, 12541.

[7] T. Cleary, T. Rawalpally, N. Kennedy, F. Chavez, *Tetrahedron Lett.* **2010**, *51*, 1533.
